# Dampening of ISGylation of RIG-I by ADAP regulates type I interferon response of macrophages to RNA virus infection

**DOI:** 10.1371/journal.ppat.1012230

**Published:** 2024-05-22

**Authors:** Yan Wang, Haixia Feng, Xiao Li, Yina Ruan, Yueping Guo, Xinxing Cui, Pengchao Zhang, Yanli Li, Xinning Wang, Xingran Wang, Luxin Wei, Yulan Yi, Lifeng Zhang, Xiaodong Yang, Hebin Liu

**Affiliations:** 1 Institutes of Biology and Medical Sciences (IBMS), Soochow University, Suzhou, Jiangsu Province, China; 2 Department of Biological Sciences, Xi’an Jiaotong-Liverpool University, Suzhou, Jiangsu Province, China; 3 Children’s Hospital, Zhejiang University School of Medicine, Hangzhou, Zhejiang Province, China; 4 Department of Veterinary Medicine, Zhejiang A&F University, Hangzhou, Zhejiang Province, China; 5 Department of General Surgery, The Fourth Affiliated Hospital of Soochow University, Suzhou, China; 6 Department of Gastrointestinal Surgery, The Second Affiliated Hospital of Soochow University, Suzhou, China; University of Arkansas for Medical Sciences, UNITED STATES

## Abstract

While macrophage is one of the major type I interferon (IFN-I) producers in multiple tissues during viral infections, it also serves as an important target cell for many RNA viruses. However, the regulatory mechanism for the IFN-I response of macrophages to respond to a viral challenge is not fully understood. Here we report ADAP, an immune adaptor protein, is indispensable for the induction of the IFN-I response of macrophages to RNA virus infections via an inhibition of the conjugation of ubiquitin-like ISG15 (ISGylation) to RIG-I. Loss of ADAP increases RNA virus replication in macrophages, accompanied with a decrease in LPS-induced *IFN-β* and *ISG15* mRNA expression and an impairment in the RNA virus-induced phosphorylation of IRF3 and TBK1. Moreover, using *Adap*^*-/-*^ mice, we show ADAP deficiency strongly increases the susceptibility of macrophages to RNA-virus infection *in vivo*. Mechanically, ADAP selectively interacts and functionally cooperates with RIG-I but not MDA5 in the activation of IFN-β transcription. Loss of ADAP results in an enhancement of ISGylation of RIG-I, whereas overexpression of ADAP exhibits the opposite effect *in vitro*, indicating ADAP is detrimental to the RNA virus-induced ISGylation of RIG-I. Together, our data demonstrate a novel antagonistic activity of ADAP in the cell-intrinsic control of RIG-I ISGylation, which is indispensable for initiating and sustaining the IFN-I response of macrophages to RNA virus infections and replication.

## Author summary

Macrophages are the major type I interferons (IFNs-I) producers upon virus infections, but they are also important target cells for many RNA viruses. Understanding how the IFN-I response of macrophages to RNA virus infection and replication is vital for the development of targeted therapies to mitigate severe viral diseases. To this end, the present study portrays a novel regulatory mechanism whereby ADAP exhibits a virus-suppressive function via dampening of RIG-I ISGylation in the induction of the IFN-I response of macrophages to RNA virus infections. Loss of ADAP dampens RIG-I-type I IFN signaling and renders macrophages more susceptible to virus infection. Therefore, this study provides a new insight into how the permissiveness of macrophages to RNA viruses can be controlled. Additionally, ADAP-RIG-I module can potentially serve as a novel target for therapeutic intervention against diseases caused by RNA viruses.

## Introduction

The macrophage, a versatile sentinel immune cell present in most tissues, plays a central role in both innate and adaptive immune defense against viral infections. Meanwhile, macrophages themselves are also important target cells for many RNA viruses including IAVs, Mayaro virus (MAYV), HIV-1, swine viruses and SARS-CoV-2, and are implicated in the pathogenesis of severe diseases caused by these viruses [1,2]. While the mechanism by which macrophage as an immune executive cell restricts viral replication has been well-elucidated, the molecular basis is not fully understood for macrophage as a permissive cell regarding the regulation of the response and permissiveness of macrophages to RNA virus infection and replication.

One of the primary responses of macrophages to viral infections is the production of antiviral type I interferons (IFNs) [3,4]. Infection and replication of these viruses in macrophages is limited through a type I IFN-dependent mechanism [5–7]. The biological response to the secreted type I IFNs by macrophages is mediated by its binding to specific receptor IFNAR to induce downstream numerous interferon-stimulated genes (ISGs) via the JAK/STAT pathway, which are major forces in controlling and restricting viral infections [8]. ISG15 is one of the most upregulated genes upon type I interferons treatment or infection by a wide range of viruses [9]. While some of ISGs have a direct antiviral activity, ISG15 serves as a small ubiquitin-like modifier whose conjugation to specific lysine residues of target proteins (ISGylation) plays a critical role in antiviral responses [10]. Similar to ubiquitination, the ISG15-mediated ISGylation is an enzymatic cascade process involving multiple modifying enzymes including the E1 activating enzyme UBE1L, the E2 conjugating enzyme UbcH8 and the E3 ligases Herc5, HHARI or Trim25 [10]. While RIG-I acts as the upstream activator of the expression of ISG15, it is also subject to ISGylation [11,12].

The retinoic acid-inducible gene I (RIG-I), a key cytoplasmic RNA helicase, is one of the major inducers of type I interferons following virus infection. RIG-I detects specific viral RNA in the cytosol, and is activated to recruit and interact with the mitochondrial adapter protein MAVS, leading to the activation of intracellular signaling cascades that activates IRF3 and NF-κB. Ultimately, these actions converge on the production of type I IFNs and proinflammatory cytokines [13,14]. Of note, essentially, RIG-I is subject to ISG15-mediated ISGylation, which negatively regulates RIG-I signaling pathway and thus reduces the production of type I IFNs [11,12].

ADAP (adhesion and degranulation-promoting adapter protein), also known as FYB (Fyn-binding protein), is a hematopoietic cell-specific immune adapter protein encoded by the *Fyb* gene [15]. ADAP facilitates the infection of RNA viruses such as HIV-1 and IAV in a T cell-dependent mechanism [16–18]. ADAP directly interacts with enzymes of the ubiquitin-like system such as the SUMO E2-binding enzyme Ubc9 [19]. Moreover, ADAP regulates the polarization and phagocytic ability of macrophages via interacting with STAT family members STAT3 and STAT1, respectively [20,21].

While macrophages are important target cells for a wide range of RNA viruses and serve as a site for the viral replication, it remains largely unclear how the RNA virus replication in macrophages is regulated, and what is the impact on the type I IFN-mediated antiviral response of macrophages to the viral infection and replication. Here we show ADAP negatively regulates ISGylation of RIG-I in macrophages, which is indispensable for the IFN-I response of macrophages to viral replication. Loss of ADAP strongly increases the susceptibility of macrophages to RNA virus infection *in vitro* and *in vivo*. Together, our data demonstrate ADAP exerts agonistic activity in the cell-intrinsic control of RIG-I activation for IFN production and signaling in macrophages in response to RNA virus infections.

## Results

### ADAP deficiency reduces type I IFN response of macrophages to RNA virus infection

**Fig 1:**
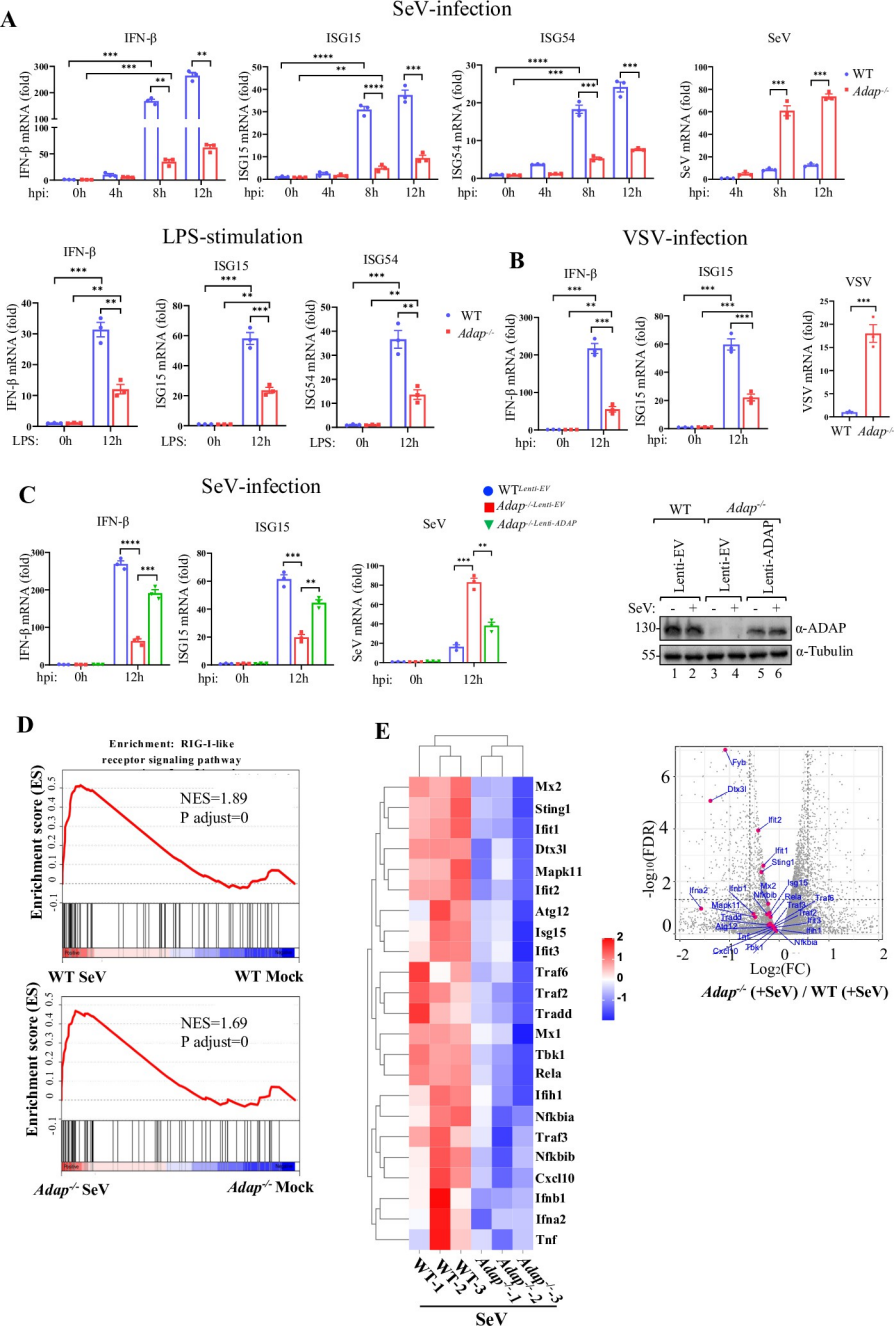
ADAP deficiency impairs the type I interferon response of macrophages to RNA virus infection. (A) WT and *Adap*^-/-^ iBMMs were either mock-infected or infected with SeV, or stimulated with LPS (1 μg/ml). At the indicated time post infection or stimulation, cells were harvested for total RNA isolation, and virus infection or LPS stimulation-induced expression of IFN-β, ISG15 or ISG54 was assayed by qRT-PCR. Data were shown as mean ± SEM (n  =  3). (B) Same experiment performed as in (A) using a different RNA virus VSV for the infection. (C) WT or ADAP knockdown RAW264.7 cells reconstituted via lentiviral transduction with ADAP or empty vector as a control for 48 h, as indicated, were either mock-infected or infected with SeV at an MOI of 3. cDNA was prepared from the cells at 12 h after SeV infection, and was subsequently subjected to qPCR analyses for *IFN-β* and *ISG15* genes. Western blot analysis was performed to verify the ADAP expression in WT RAW264.7, or ADAP knockdown RAW264.7 cells reconstituted with empty vector (lanes 3 and 4) or ADAP (lanes 5 and 6) for 12 h, under mock-infected or SeV infection conditions. Data are shown as the mean ± SEM for three independent experiments and fold induction is in reference to uninfected cells (**p < 0.01, ***p < 0.001, ****p < 0.0001). (D) RNA-seq analysis of peritoneal macrophages from WT and *Adap*^-/-^ mice following mock or SeV infection. GSEA enrichment plots of RIG-I-like receptor signaling pathway comparing SeV-infected peritoneal macrophages with mock-infected peritoneal macrophages from WT mice (upper panel) and from *Adap*^*-/-*^ mice (lower panel). Normalized enrichment score (NES) and the FDR q-value of each enrichment are shown. (E) Expression heatmap (left panel) and volcano plot (right panel) represent a number of differentially expressed genes (DEGs) related to type I IFN and anti-viral signaling between WT and *Adap*^-/-^ peritoneal macrophages in response to SeV infection.

While macrophages constitute a key source of the type I interferons (IFNs-I) upon viral infections, it is also an important target cell for many RNA viruses [22,23]. We previously showed that ADAP modulates inflammatory response of LPS-stimulated macrophages [20]. To investigate whether ADAP plays a role in the host responses of macrophages to viruses, quantitative PCR was performed to examine the changes in the mRNA expression of IFN-I, and ISG15 and ISG54, two major interferon-stimulated genes (ISGs) downstream IFN-I, in iBMMs upon LPS stimulation or RNA virus infection. As shown in Fig 1A, while both infection with Sendai virus (SeV), a model RNA virus that preferentially stimulation RIG-I signaling [24,25], and LPS stimulation induced a robust response in the increase of IFN-β, ISG15 and ISG54 mRNA in both wild-type (WT) and ADAP knock-out *(Adap*^*-/-*^) macrophages, the induced expression levels of these genes were significantly lower in *Adap*^*-/-*^ macrophages compared to that in WT macrophages, indicating ADAP is required for IFN-I response of macrophages to RNA virus infection or LPS stimulation. To further confirm this, the same assays as in Fig 1A were performed by using VSV, another reported RNA virus that stimulates RIG-I signaling [26,27]. As shown in Fig 1B, similar result was observed in the infection with VSV. Importantly, as shown in Fig 1C, the impairment of the SeV-induced IFN-β and ISG15 mRNA expression in ADAP knockdown macrophage cell line RAW264.7 cells could be largely restored by lentiviral-mediated reconstitution of ADAP. In line with this, while SeV mRNA was detected at much higher quantities in the ADAP knockdown macrophages relative to the WT cells, ADAP reconstitution significantly decreased this hyper-replication of SeV in ADAP knockdown macrophages. These data further support ADAP is required for IFN-I response of macrophages to RNA virus infection. To assess whether this was the case *in vivo*, RNA-Seq was performed on purified peritoneal macrophages from WT and *Adap*^*-/-*^ mice with or without challenges of SeV. Enrichment analysis (GSEA) of the RNA-Seq revealed that the RIG-I-like receptor signaling pathway was efficiently induced upon SeV infection in both WT and *Adap*^*-/-*^ macrophages (Fig 1D). In contrast, however, the expression of type I interferon *IFN-α*/*β* and the ISGs including *ISG15*, *IFIT1* and *IFIT2* were decreased in *Adap*^*-/-*^ macrophages compared with that in WT macrophages as shown in the expression heatmap and volcano plot (Fig 1E). These results point to that ADAP is required for type I IFN response of macrophages following the RNA virus infection.

### ADAP selectively interacts with RIG-I but not MDA5, which occurs mainly in the mitochondria but not lipid rafts

**Fig 2:**
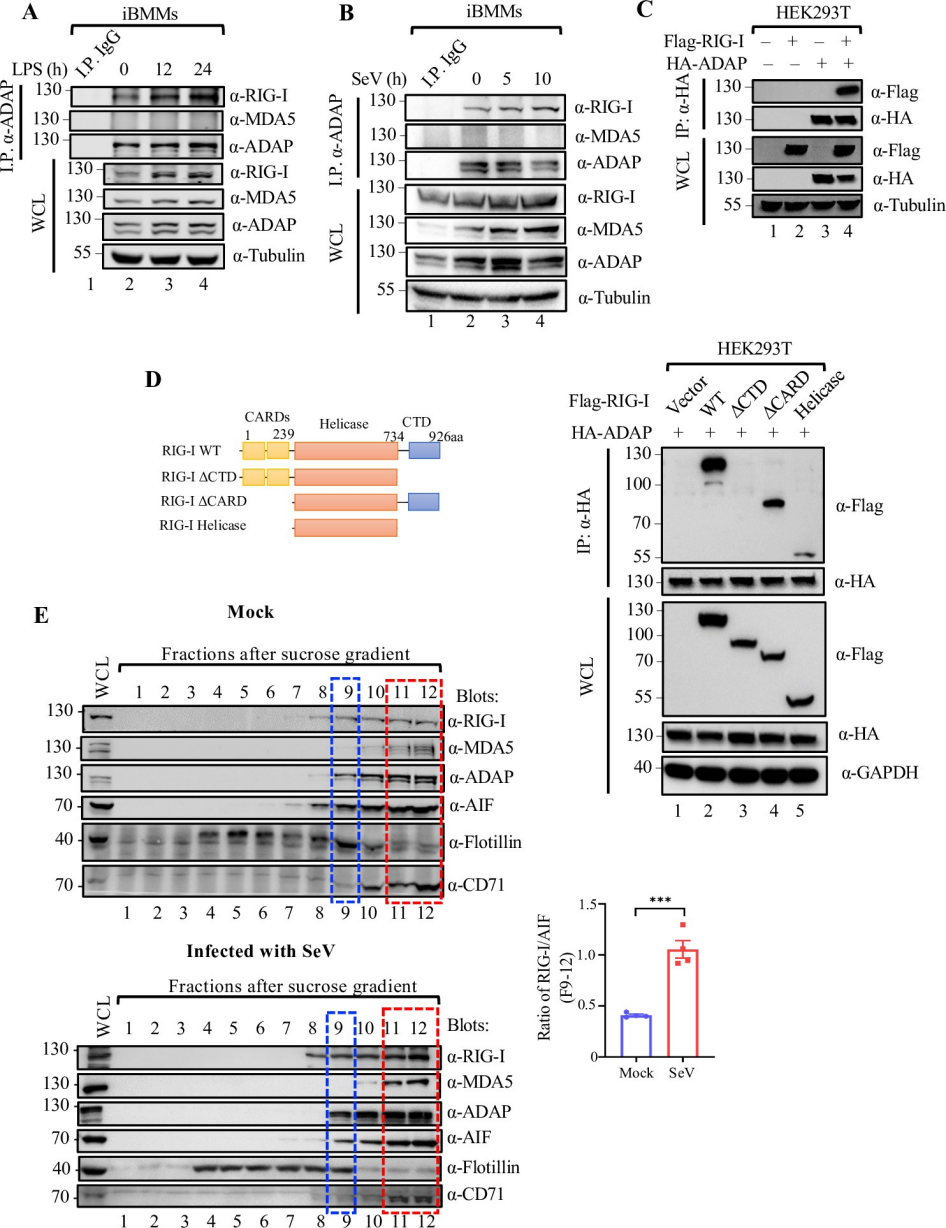
ADAP selectively interacts with the RIG-I but not MDA5, which occurs mainly in the mitochondria but not lipid rafts. (A) Whole-cell lysates (WCL) of iBMMs either mock-treated or stimulated with 1 μg/ml LPS for 0, 12 and 24 h were prepared and subjected to immunoprecipitation with an anti-ADAP antibody, and Western blotting was performed with antibodies as indicated. (B) The effect of SeV infection on interaction between RIG-I and ADAP in iBMMs. Cell lysates of iBMMs either mock-infected or infected with SeV for 0, 5 and 10 h were subjected to immunoprecipitation with an anti-ADAP antibody, and Western blotting was performed with antibodies as indicated. (C) RIG-I interacts with ADAP *in vitro*. Whole-cell lysates of HEK293T cells co-transfected with Flag-RIG-I and HA-ADAP constructs as indicated were prepared at 48 h post transfection and subjected to immunoprecipitation with an anti-HA antibody, followed by immunoblotting with an anti-Flag antibody. (D) Left panel: Schematic of RIG-I WT and truncated mutant constructs used in this study. Right panel: ADAP binds to CTD region of RIG-I. HEK293T cells were co-transfected with HA-ADAP and Flag-tagged RIG-I mutant constructs as indicated. Whole-cell lysates of the transfected cells were prepared at 48 h post transfection and subjected to immunoprecipitation with an anti-HA antibody, followed by immunoblotting with indicated antibodies. (E) Whole-cell lysates of mock-infected iBMMs (left upper panel) or SeV infected iBMMs (left lower panel) were prepared, and subjected to a discontinuous sucrose gradient (5%, 30% and 40%) ultracentrifugation. Equal volumes (∼400 μl) of fractions (F1 to F12) were collected from the top to bottom, and subjected to immunoblotting with indicated antibodies against ADAP, RIG-I, MDA5, AIF (a mitochondrial marker), Flotillin (a lipid rafts marker) and CD71 (a non-raft membrane marker) after fractionation. The bar graph showing the ratio of RIG-I relative to AIF enriched in the F9-12 fractions from the mock-infected and SeV-infected cells that was calculated based on the quantitation analysis of the RIG-I and AIF protein bands density (right panel).

RIG-I is a key cytoplasmic viral RNA sensor and is one of the major drivers of type I IFN during viral infections [28,29]. Given loss of ADAP leads to a decrease in the IFN-I induction in macrophages response to SeV infection, we set out to examine whether there is a potential functional interaction between RIG-I and ADAP. Anti-ADAP coimmunoprecipitation assays (co-IPs) was performed on protein lysates from iBMMs that were treated with or without either infection with SeV or LPS stimulation, followed by anti-RIG-I immunoblotting. Endogenous RIG-I was readily coimmunoprecipitated with ADAP-specific antibody, and, interestingly, the assembly of ADAP-RIG-I complex was enhanced upon LPS simulation or SeV infection (Fig 2A, lanes 2–4; Fig 2B, lanes 2–4). In contrast, no such interaction was observed between ADAP and MDA5, another primary viral RNA sensor (Fig 2A, lanes 2–4; Fig 2B, lanes 2–4), suggesting ADAP selectively interacts with RIG-I but not MDA5 in macrophages. Further, we validated ADAP-RIG-I interaction with overexpressed proteins in HEK293T cells where endogenous ADAP is absent. Flag-RIG-I was co-expressed with empty vector or HA-ADAP, followed by anti-HA co-immunoprecipitation and anti-Flag blotting. Ectopically expressed HA-tagged ADAP was coprecipitated with RIG-I in HEK293T cells (Fig 2C, lane 4), indicating a physical interaction of ADAP and RIG-I *in vitro*. To verify the interaction and determine the specific region of RIG-I responsible for the interaction, we constructed several Flag-tagged RIG-I truncated mutants (ΔCTD, ΔCARD and Heli) (Fig 2D, left panel) and assessed their abilities to bind to ADAP. As shown in Fig 2D, the wild-type RIG-I (lane 2) and the N-terminal CARD domain-deletion mutant RIG-I-ΔCARD containing aa 240–926 (lane 4) retained a comparable interaction activity with ADAP as full-length RIG-I. In contrast, the RIG-I C-terminal CTD deletion mutant RIG-I-ΔCTD containing aa 1–734 virtually abolished ability ADAP binding (lane 3), and the isolated helicase-domain of the RIG-I (aa 239–734) showed an approximate 60% decrease in binding with ADAP compared to that of wild-type RIG-I (lane 5 vs. lane 2), suggesting that the C-terminal CTD (aa 734–926) of RIG-I is critical for the interaction with ADAP.

**Fig 3:**
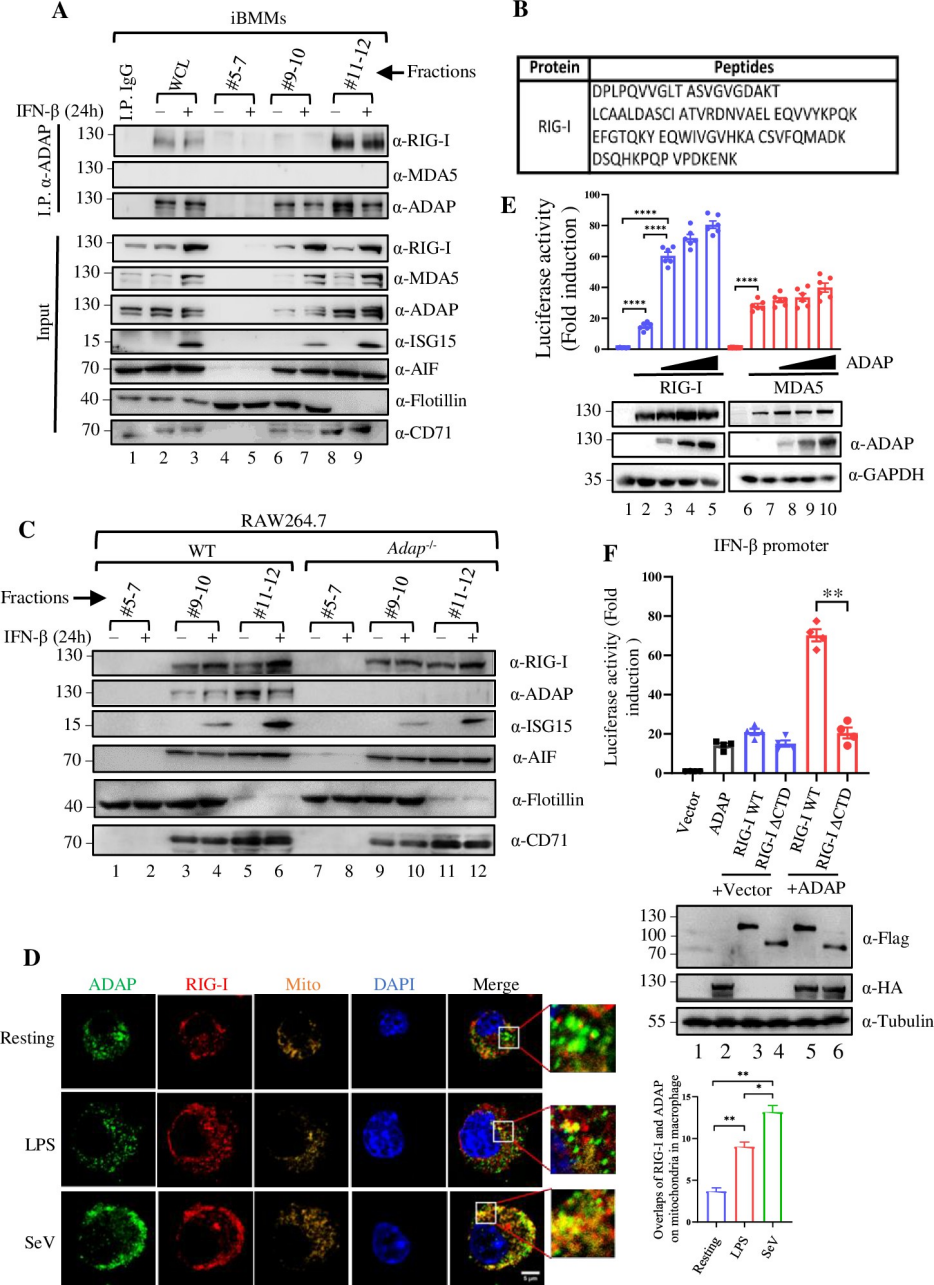
ADAP selectively interacts with the RIG-I but not MDA5, which occurs mainly in the mitochondria but not lipid rafts. (A) WT iBMMs were left unstimulated or stimulated with 50 ng/ml IFN-β for 24 h and subjected to sucrose gradient centrifugation. Fractions 5–7, 9–10 and 11–12 and subjected to immunoprecipitation with an anti-ADAP antibody, and immunoblot analysis with the indicated antibodies. (B) Fractions 11–12 of sucrose gradient from iBMMs were immunoprecipitated by an anti-ADAP antibody, followed by LC-MS/MS analysis. The sequence shown was the peptides of RIG-I identified by LC-MS/MS. (C) WT and ADAP knockdown RAW264.7 cells were either mock-treated or stimulated with 50 ng/ml IFN-β for 24 h, followed by discontinuous sucrose gradient centrifugation for subcellular fractionation. Fractions 5–7, 9–10 and 11–12 were subjected to immunoblot analysis with the indicated antibodies. (D) Confocal microscopic images of iBMMs that were either left untreated, stimulated with LPS or infected with SeV for 12 h, followed by staining with MitoTracker (orange), RIG-I (red), ADAP (green) and Dapi (blue). Scale bar = 5 μm. (E) Overexpression of ADAP potentiates activation of the IFN-β promoter mediated by RIG-I but not MDA5. Co-transfection of HEK293T cells with an IFN-β-promoter luciferase reporter plasmid, a renilla luciferase transfection control, and either a RIG-I plasmid (empty blue bars) or a MDA5 plasmid (empty red bars) together with an ADAP construct in increasing amounts (50, 200 and 400 ng of plasmid) or empty vector control (EV). 48 h post transfection, luciferase activity was measured normalized to renilla luciferase activity, and fold induction of luciferase activity compared to vector control was plotted in the bar chart on the upper panel. Data represent the mean ± SEM of six replicates. Lower panel showing the representative Western blot analysis of the whole cell lysates of the transfected cells with antibodies as indicated. (F) Same luciferase dual reporter assay performed as in (E) by co-transfection of HEK293T cells with a plasmid encoding RIG-I WT or ADAP-binding deficient mutant RIG-I ΔCTD together with an ADAP plasmid (empty red bars) or empty vector control (empty blue bars). Data represent the mean ± SEM of four replicates. **p < 0.01. Lower panel showing the representative Western blot analysis of the whole cell lysates of the transfected cells with antibodies as indicated.

To assess the subcellular localization of ADAP-RIG-I interaction, sucrose gradient sedimentation was performed. Cell extracts from iBMMs were ultracentrifuged in sucrose gradient, and the localization of ADAP and RIG-I in the specific cellular fractions from the gradient were determined and characterized by immunoblot using antibodies against ADAP and RIG-I, together with antibodies against mitochondrial marker AIF, lipid rafts marker Flotillin 1, and the non-raft membrane marker CD71 [30–32]. As shown in Fig 2E, the lipid rafts marker Flotillin 1 was observed mainly in fractions 4 to 9 (F4-9) of the gradient (row 5, lanes 4–9), whereas CD71 was observed mainly in F10-12 (row 6, lanes 10–12). The mitochondrial marker AIF appeared in F8-12 (row 4, lanes 8–12). ADAP was broadly dispersed in F9-12 of the gradient, among which a large portion of ADAP (≈ 60%) was with mitochondrial in F11-12 (indicated by the red dashed line box), and to a lesser extent with lipid raft fraction in F9 (the blue dashed line box). Interestingly, RIG-I was also mainly enriched in F11-12 (Fig 2E, upper panel, row 1, lanes 10–12). The gradient ultracentrifugation in the fractionation of RIG-I, ADAP and AIF was also performed with SeV-infected iBMMs (Fig 2E lower panel). Further, the RIG-I and AIF protein bands density was quantified, based on which the ratio of RIG-I relative to AIF enriched in the F9-12 fractions form mock and SeV-infected cells was analysed (Fig 2E, bar chart panel). The ratios of RIG-I/AIF in the F9-12 fractions (mainly in mitochondria) was significantly higher in cells upon SeV infection relative to that in mock-infected cells. Furthermore, different cellular fractions of the gradient containing ADAP and RIG-I were subjected to anti-ADAP co-immunoprecipitation, followed by anti-RIG-I immunoblotting. RIG-I was readily coimmunoprecipitated with ADAP in the combined F11-12 but not in F9-10 under conditions of both resting and IFN-β stimulation (Fig 3A, row 1, lanes 2–3, 8–9). In contrast, despite that MDA5 was also mainly detected in F10-12 (Fig 2E, row 2, lanes 10–12), MDA5 was not co-immunoprecipitated with ADAP from any fractions (Fig 3A, row 2). The presence of RIG-I in the Co-IP complex of ADAP from fractions F11-12 was further confirmed by LC-MS/MS with the urea-eluted proteins from anti-ADAP immunoprecipitates from F11-12 (Fig 3B). Moreover, although ADAP deficiency didn’t lead to a change in the distribution patterns of RIG-I and ISG15, the abundance of RIG-I and ISG15 was much lower under IFN-β stimulation in ADAP deficiency cells compared to WT cells (Fig 3C, lanes 9–12 vs. lanes 3–6). Moreover, the subcellular localization of RIG-I and ADAP in iBMMs was also examined by immunofluorescence assay. As shown in Fig 3D, a substantial portion of ADAP and RIG-I was co-localized in cytoplasm, most of which occurred at the mitochondria, which was increased upon LPS treatment or SeV infection. Finally, given the interaction of ADAP with RIG-I, we further examined whether there is a functional cooperation between them in the regulation of IFN-I transcription. As shown in Fig 3E, ADAP had a synergistic effect in a dose dependent manner with RIG-I but not MDA5 in the activation of IFN-β transcription, as measured by luciferase report assay. Interestingly, ADAP collaborates with WT RIG-I but not the ADAP-binding deficient RIG-I ΔCTD mutant in the activation of IFN-β promoter activity (Fig 3F).

### ADAP negatively regulates RIG-I ISGylation in macrophages in response to RNA virus infection

**Fig 4:**
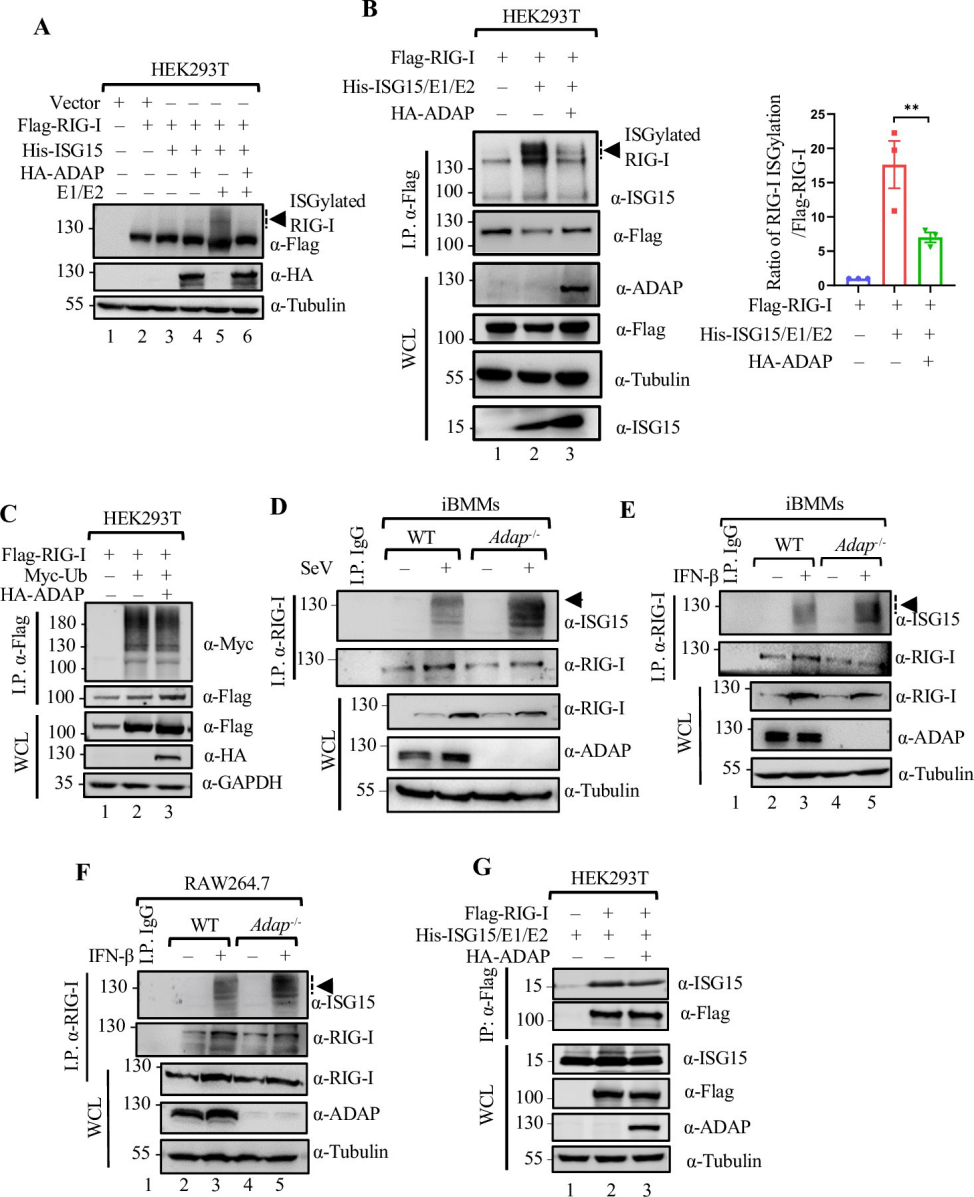
ADAP deficiency arguments RIG-I ISGylation in the virus-infected or IFN-β stimulated macrophages. (A, B) *In vitro* RIG-I ISGylation assay. HEK293T cells were co-transfected with Flag-RIG-I, ISGylation system plasmids (His-ISG15, HA-UBE1L, Myc-Ubc8H) and either empty plasmid or HA-ADAP as indicated. 48 h post transfection, ISGylation of RIG-I was determined by immunoblot analysis with the indicated antibodies (A), or immunoprecipitation with an anti-Flag antibody followed by immunoblotting with the indicated antibodies (B). Bars represent the mean ± SEMs of the three independent experiments (n = 3). **p < 0.01. (C) Co-transfection of HEK293T cells with Flag-RIG-I and or Myc-ubiquitin and either empty plasmid or HA-ADAP, 48 h post transfection, ubiquitination of RIG-I was determined by immunoprecipitation with an anti-Flag antibody followed by immunoblot with the indicated antibodies. (D) Whole cell lysates of WT and *Adap*^*-/-*^ iBMMs either mock treated or infected with SeV at an MOI of 3 for 12 h were prepared and subjected to immunoprecipitation with an anti-RIG-I antibody, followed by Western blot analysis with antibodies against ISG15 and RIG-I, as indicated. The input (10%) of the whole cell lysates were subjected to Western blot analysis with antibodies as indicated. (E, F) Analysis of ISGylation of endogenous RIG-I in macrophages upon IFN-β stimulation. RIG-I was immunoprecipitated from WT and *Adap*^-/-^ iBMMs (E) or from WT and ADAP knockdown RAW264.7 cells (F) with or without IFN-β stimulation for 24 h, followed by immunoblot with the indicated antibodies. (G) HEK293T cells were co-transfected with Flag-RIG-I, ISG15 and either empty plasmid or HA-ADAP. 48 h post transfection, the effect of ADAP on the interaction between RIG-I and ISG15 was determined by immunoprecipitation with an anti-Flag antibody followed by immunoblot with the indicated antibodies.

RIG-I is subject to ISG15-mediated ISGylation during RNA virus infection, which inhibits the type I IFN induction [11]. We thus assessed whether ADAP could affect ISG15 conjugation to RIG-I. First, we performed *in vitro* ISGylation assay with RIG-I. HEK293T cells were transfected with expression constructs encoding for Flag-RIG-I, His-ISG15, HA-UBE1L (E1 enzyme), Myc-Ubc8H (E2 enzyme), with or without co-transfection of HA-ADAP. Cell lysates from these transfected cells were subjected to Western blot analysis with antibodies against Flag and HA. As previously reported [11,33,34], higher molecular weight bands representing ISGylated RIG-I were detected in the cell lysates from cells co-transfected with RIG-I together with ISG15 and E1, E2 enzyme (Fig 4A, lanes 5 and 6). Interestingly, addition of HA-ADAP decreased the amount of the higher band of ISGylated RIG-I (Fig 4A, lane 6 vs. lane 5). Similar result was also observed with the enriched RIG-I in the *in vitro* ISGylation assay, wherein addition of HA-ADAP decreased the ISGylation of Flag-RIG-I that was enriched by anti-Flag immunoprecipitation (Fig 4B, lane 2 vs. lane 3). In contrast, an addition of ADAP did not cause difference in ubiquitination of RIG-I (Fig 4C, lane 2 vs. lane 3). Further, to verify whether this negative effect of ADAP on RIG-I ISGylation also occurred in macrophages *in vivo*, we assessed the effect of ADAP-deficiency on SeV infection-induced RIG-I ISGylation in macrophages. RIG-I was immunoprecipitated from cell extracts of WT or *Adap*^*-/-*^ iBMMs that were either mock-treated or infected with SeV, followed by blotting with anti-RIG-I or anti-ISG15. As shown in Fig 4D, the level of RIG-I ISGylation was significantly enhanced in both WT macrophages (lane 2 vs lane 1) and *Adap*^-/-^ macrophages (lane 4 vs lane 3) upon SeV infection, but in comparison, the level of increase was more profound in *Adap*^-/-^ cells (lane 4 vs lane 2), which is in congruent with the result that ADAP dampens SeV-induced RIG-I ISGylation. Furthermore, we assessed the effect of ADAP-deficiency on RIG-I ISGylation in WT or *Adap*^-/-^ iBMMs or RAW264.7 cells that were either mock-treated or stimulated with IFN-β. As shown in Fig 4E and 4F, despite a decreased level of RIG-I, the induced RIG-I ISGylation upon IFN-β treatment was substantially higher in *Adap*^*-/-*^ iBMMs or in ADAP knockdown RAW264.7 cells than that in WT cells (lanes 4 and 5 vs. lanes 2 and 3). Moreover, as reported [11], Co-IP assay showed that RIG-I interacts with ISG15, which was not affected by ectopic expression of ADAP (lane 2 vs lane 3, Fig 4G). Thus, ADAP selectively inhibits the ISGylation but not the ubiquitination of RIG-I without affecting the interaction of RIG-I-ISG15 in macrophages.

### ADAP is indispensable for the full activation of TBK1-IRF3 for IFN-β production downstream of RIG-I in macrophages

**Fig 5:**
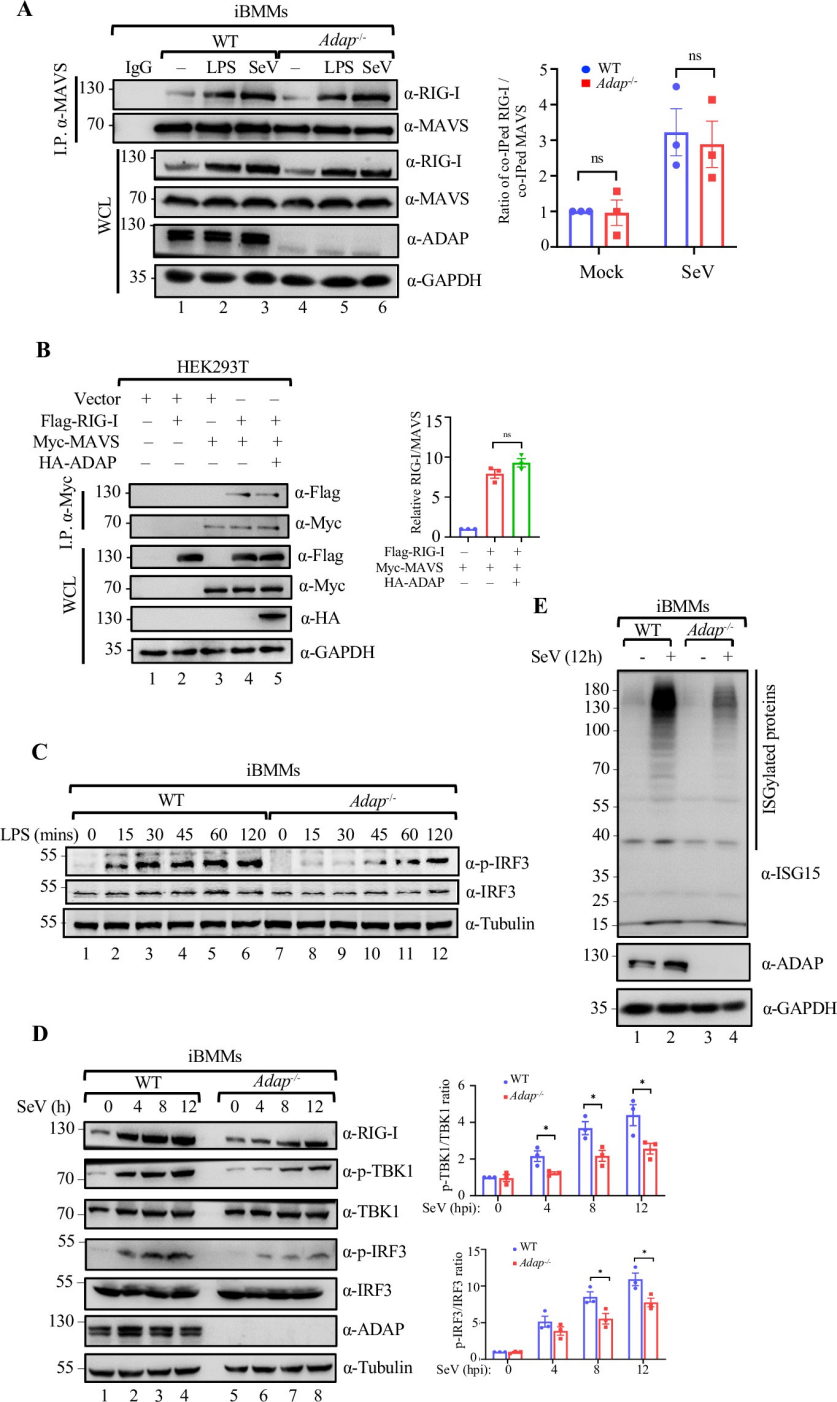
ADAP is indispensable for the full activation of TBK1-IRF3 for IFN-β production downstream of RIG-I in macrophages. (A) WT and *Adap*^-/-^ iBMMs were either left unstimulated or stimulated with 1 μg/ml LPS or infected with SeV for 12 h. Cell extracts were immunoprecipitated with an anti-MAVS antibody followed by immunoblot with the indicated antibodies. The ratio for co-IPed RIG-I/MAVS was calculated by dividing the relative density of RIG-I band by the relative density of the MAVS in the same lane. Bars represent the mean ± SEMs of the three independent experiments (n = 3). n.s., not significant (*p* > 0.05). (B) HEK293T cells were co-transfected with Flag-RIG-I, Myc-MAVS and/or HA-ADAP expression constructs as where indicated. Cells were harvested 48 h post transfection followed by immunoblot analysis with indicated antibodies. The ratio for co-IPed RIG-I/MAVS was calculated by dividing the relative density of RIG-I bands in lanes 3, 4 and 5 by the relative density of the MAVS in the same lane. Bars represent the mean ± SEMs of the three independent experiments (n = 3). (C) WT and *Adap*^-/-^ iBMMs were treated with 1 μg/ml LPS for the indicated times. Cell extracts were subjected to immunoblot analysis with the indicated antibodies. (D) Whole cell extracts of WT and *Adap*^-/-^ iBMMs infected with SeV for the indicated times were subjected to immunoblot analysis for total IRF3 and phospho-IRF3 (ser-396) or total TBK1 and phospho-TBK1 (ser-172) with the indicated antibodies. Bar chart showing densitometry analysis of phospho-IRF3 (ser-396), phospho-TBK1(ser-172) normalized to total IRF3 and total TBK1. Bars represent the mean ± SEMs of the three independent experiments (n = 3). *p < 0.05. (E) Loss of ADAP led to a substantial decrease in the global ISGylation level induced by RNA virus infection in macrophages. WT and *Adap*^-/-^ iBMMs were either mock-infected or infected with SeV for 12 h, followed by immunoblotting with the indicated antibodies.

When RIG-I is activated by viral RNAs, the CARDs from multiple RIG-I assemble and interact with MAVS, leading to downstream signaling activation [29,35,36]. To address whether ADAP-RIG-I interaction affects the RIG-I binding to MAVS, we performed anti-MAVS coimmunoprecipitation in WT and ADAP deficient iBMMs with or without LPS treatment or SeV infection, followed by immunoblot analyses probing with anti-RIG-I. As shown in Fig 5A, in agreement with the previous report [37,38], RIG-I presented in the Co-IP complex of MAVS from iBMMs, and the level of co-IPed RIG-I with MAVS was significantly increased upon SeV infection. In contrast, no significant difference was observed with the level of co-IPed RIG-I by the anti-MAVS antibody between WT and *Adap*^*-/-*^ macrophages under both resting (Fig 5A, lane 2 vs. lane 5) and infection with SeV infection (Fig 5A, lane 4 vs. lane 7). Further, *in vitro* pull-down assay showed overexpression of ADAP hardly altered the amount of the precipitated Flag-RIG-I by Myc-MAVS (Fig 5B, lane 5 vs. lane 4).

We next assessed the effect of ADAP deficiency on the activation of IRF3 and TBK1, the key downstream signaling molecules of RIG-I in macrophages upon LPS stimulation or viral infection [13,39,40]. While LPS stimulation induced Ser396 phosphorylation of IRF3 in both ADAP deficient and WT macrophages (Fig 5C), the level of IRF3 phosphorylation was remarkedly decreased with slower kinetics in ADAP deficient cells as compared with that in WT cells (Fig 5C, lanes 7–12 vs. lanes 1–6). Similarly, SeV infection induced the phosphorylation of IRF3 and TBK1 in both WT and ADAP deficient cells, but IRF3 and TBK1 phosphorylation level was substantially lower when ADAP was absent (Fig 5D, lanes 5–8 vs. lanes 1–4). In line with the impaired IRF3 signaling axis upon ADAP depletion, the level of global ISGylation in response to SeV infection was significantly lower in the *Adap*^*-/-*^ macrophages than that in WT cells (Fig 5E, lane 4 vs lane 2). Together, these data suggest that while ADAP does not compete with MAVS for the binding with RIG-I, the ADAP-RIG-I complex is needed for full activation of signaling molecules IRF3 and TBK1 for IFN-β production in RIG-I signaling pathway.

### Loss of ADAP enhances RNA virus replication in macrophages

**Fig 6:**
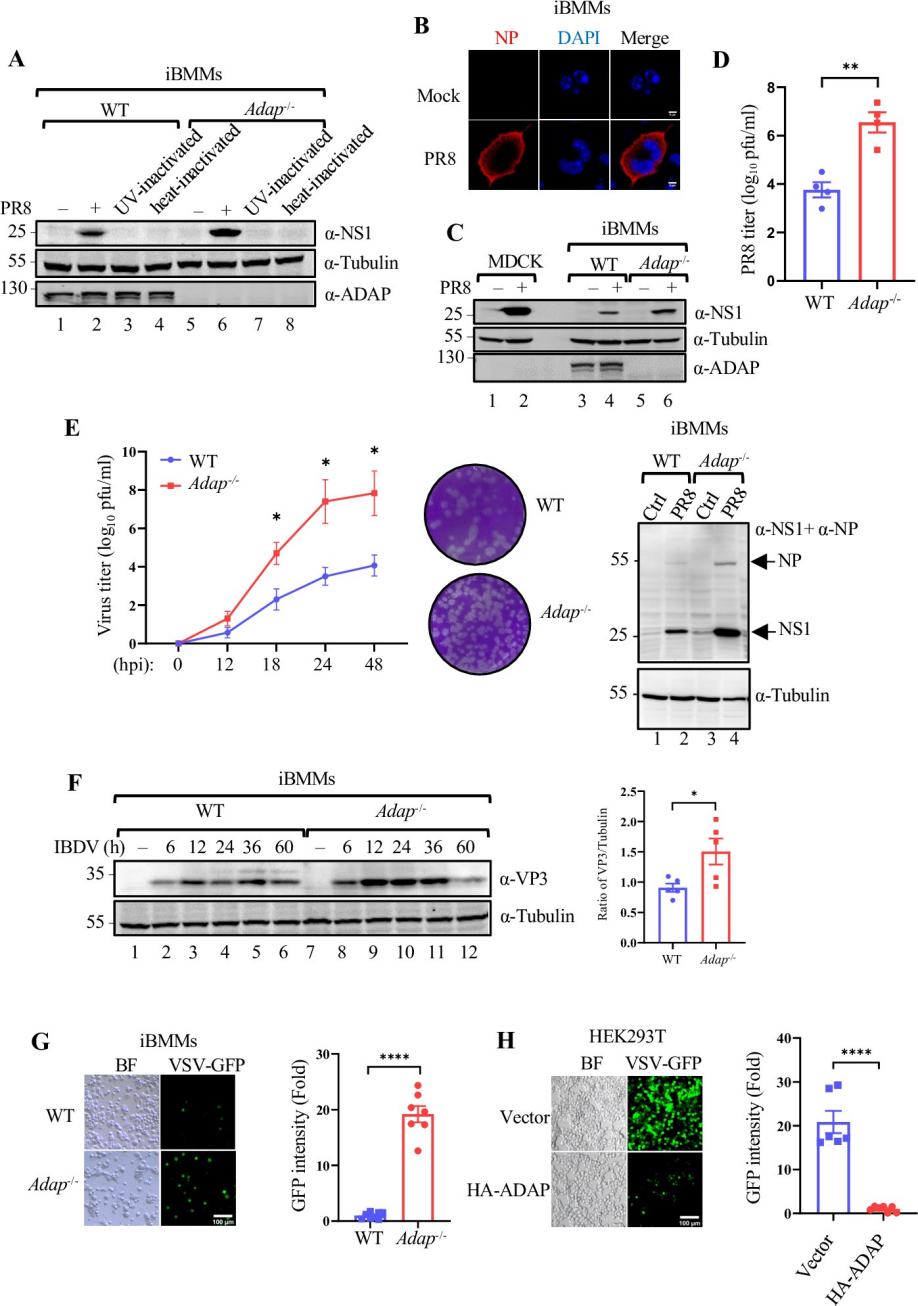
Loss of ADAP enhances RNA virus replication in macrophages. (A) Cell lysates of WT and *Adap*^-/-^ iBMMs infected with either live PR8 or UV-inactivated or heat-inactivated PR8 for 24 h were subjected to immunoblot analysis with the indicated antibodies. (B) Confocal microscopic images of iBMMs that were either mock-infected or infected with PR8 for 24 h, followed by immunostaining with an anti-NP antibody (red) and staining with DAPI (blue); scale bar = 5 μm. (C) Cell lysates of iBMMs either mock-infected or infected with IAV-PR8 were prepared at 24 h post viral infection and subjected to immunoblot analysis with the indicated antibodies. MDCK cells served as a positive control for PR8 infection. (D) WT and *Adap*^-/-^ iBMMs were infected with PR8 for 24 h. Virus titers in the supernatants of infected iBMMs were measured by virus plaque assay on the MDCK cells in 12-cell plates. (E) The PR8 replication between in WT and in *Adap*^*-/-*^ iBMMs over a time course of 48 h post infection (hpi). WT or *Adap*^*-/-*^ iBMMs were infected with PR8 (MOI of 3) for various time points (0, 12, 18, 24 and 48 h). Plaque assays were performed by applying the precleared supernatants from the PR8 infected cells to infect monolayers of MDCK cells as described in Method. The viral plaques were plotted (left panel). Data are representative of the mean and SEM of 3 replicates. Representative images of plaques formed on MDCK cells inoculated the supernatants from WT or *Adap*^*-/-*^ iBMMs that were infected with PR8 (MOI of 3) at the time point of 18 hpi (middle panel). Representative immunoblotting of cell lysates of WT and *Adap*^*-/-*^ iBMMs either mock-infected or infected with PR8 at time point of 24 hpi with antibodies against IAV NP and NS1 (mixed) (right panel). Tubulin served as a loading control. (F) Cell lysates of WT and *Adap*^-/-^ iBMMs infected by IBDV for the indicated times were subjected to immunoblot analysis with the antibody against IBDV VP3. Tubulin served as a loading control. Bar chart showing the densitometry analysis of VP3 bands normalized to Tubulin between WT and *Adap*^-/-^ iBMMs with IBDV infection for various time points. *p < 0.05. (G) Confocal microscopic images of WT and *Adap*^-/-^ iBMMs that were infected with VSV-GFP for 12 h. Scale bar = 100 μm. The intensity of GFP fluorescence were analyzed by ImageJ. (H) Confocal microscopic images of HEK293T cells transfected with either empty vector or HA-ADAP that were infected with VSV-GFP for 12 h. Scale bar = 100 μm. The intensity of GFP fluorescence were analyzed by ImageJ. Bars represent the mean ± SEMs of the six independent experiments (n = 6). ****p < 0.0001.

The inhibitory effect of ADAP on IFN-I induction via modulating the ISGylation of RIG-I prompted us to assess whether ADAP could affect the susceptibility of macrophages to RNA virus infection. iBMMs were used for infection with PR8, a vaccine strain of H1N1 influenza virus. The virus infection and replication in iBMMs was monitored by Western blot analysis with antibodies against the viral proteins. The IAV viral protein NS1 was detected in the iBMMs at 24 h post infection with live IAV-PR8 (Fig 6A, lanes 2), but not in the iBMMs that were inoculated with the either UV or heat inactivated IAV (Fig 6A, lanes 3, 4, 7 and 8). The replication of PR8 in iBMMs was further confirmed by immunofluorescence microscopy using NP specific antibody (Fig 6B). Of note, interestingly, the replication of PR8 in *Adap*^*-/-*^ iBMMs was significantly higher compared with that in WT iBMMs as determined by Western blot analysis with anti-IAV viral protein NS1 (Fig 6A, lane 6 vs. lane 2; Fig 6C, lane 6 vs. lane 4), as well as by plaque assay for virus titers (Fig 6D). Further, plaque assays were performed with the supernatants from the PR8 infected iBMMs to evaluate the kinetics of PR8 replication in WT and *Adap*^*-/-*^ iBMMs, showing that ADAP deficiency led to a significant increase in the viral replication at 18, 24 and 48 hrs post infection relative to that in WT cells (left panel of Fig 6E). The direct PR8 replication in WT and *Adap*^*-/-*^ iBMMs at the time point of 24 hpi was verified by Western blot analysis using anti-NP and anti-NS1 antibodies (right panel of Fig 6E). This was also the case for other RNA viruses whose replication was significantly higher in *Adap*^*-/-*^ iBMMs than in WT iBMMs, including: IBDV, a dsRNA model virus, as detected by Western blot analysis with anti-viral protein VP3 (Fig 6F, lanes 8–12 vs. lanes 2–6), VSV (engineered with GFP integration), as measured by the fluorescence intensity and abundance (Fig 6G). On the contrary, ectopic expression of ADAP remarkedly decreased the VSV-GFP replications in HEK293T cells (Fig 6H).

Together, these data suggest ADAP is a detrimental host protein for the replication of RNA viruses in macrophages, and ADAP deficiency renders macrophages more susceptible to RNA virus infection and replication.

### Loss of ADAP renders macrophages more susceptible to RNA viruses *in vivo*

**Fig 7:**
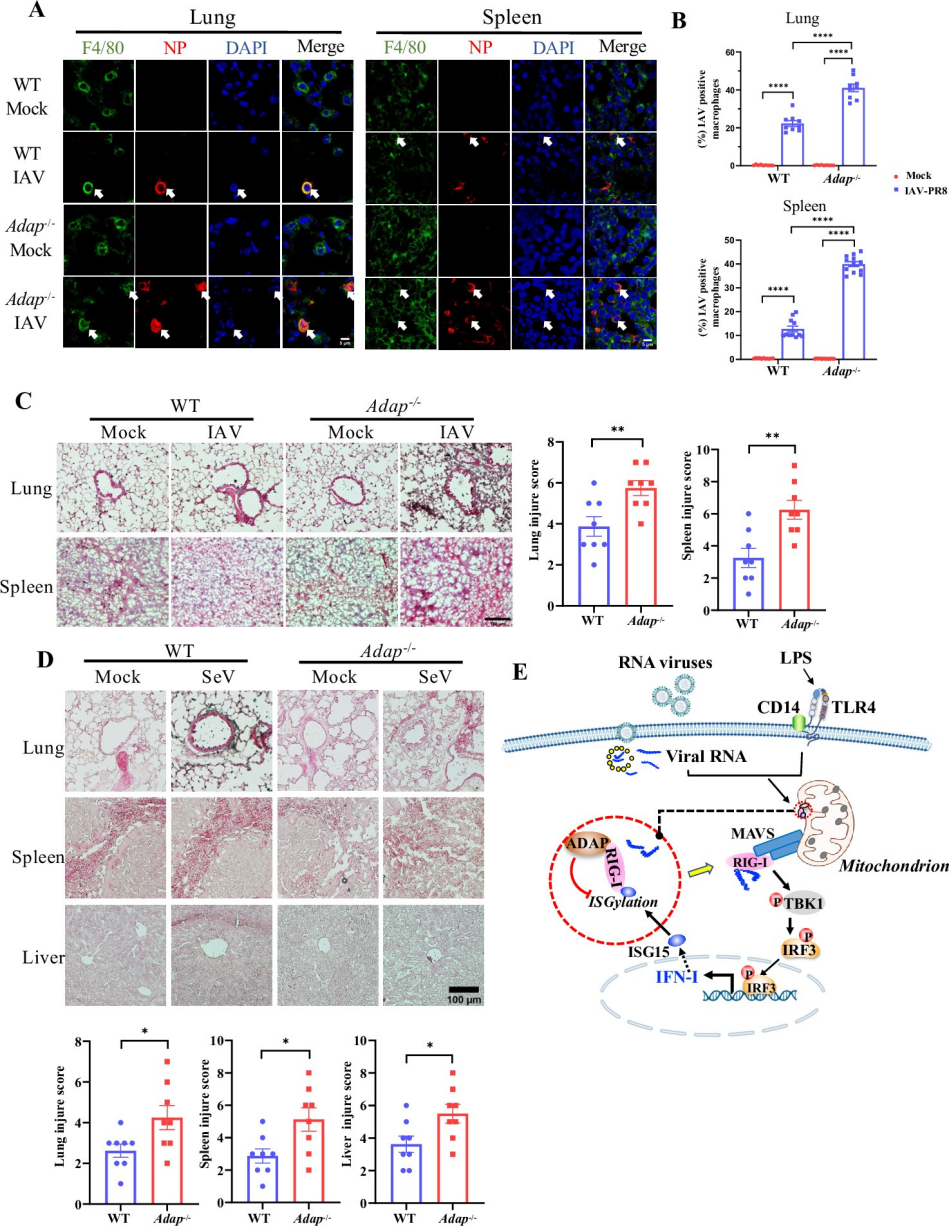
ADAP deficiency renders macrophages more susceptible to RNA infections *in vivo*. (A) Confocal microscopic images of mouse lung and spleen tissues from WT and *Adap*^-/-^ mice that were either mock infected or infected with IAV-PR8. Seven days post infection, lung and spleen tissues were fixed and stained with F4/80 (green), NP protein (red) and DAPI nuclear dye (blue). The scale bar = 5 μm. (B) The percentage of IAV positive macrophages in the lung (upper panel) and spleen (lower panel). Bars represent the mean ± SEMs of at least three independent experiments. **** *p* < 0.0001. (C) H&E staining of the lungs and spleens from WT and *Adap*^-/-^ mice with or without IAV infection. Scale bar = 100 μm. Bars represent the mean ± SEMs of eight independent experiments (n = 8). **p < 0.01. (D) H&E staining of the lungs, spleens and livers from WT and *Adap*^-/-^ mice with or without SeV infection. Scale bar = 100 μm. Bars represent the mean ± SEMs of eight independent experiments (n = 8). *p < 0.05. (E) Schematic model for the role of ADAP in regulating type I IFN response of macrophages in response to RNA virus infection by control of the ISGylation of RIG-I. The binding of ADAP to RIG-I potentiates the activation of RIG-I via preventing RIG-I from ISGylation, and this accounts for the increase in the levels of type I IFNs and ISG15. As such the macrophages are in an anti-viral state. On the contrary, when ADAP is underexpressed or deficient in macrophages, the restraint of ADAP on RIG-I ISGylation is partially removed, resulting in an inhibition of RIG-I signaling. As a consequence, the viral infection induced the production of type I IFN and its stimulated ISG15 is robustly reduced, causing an excessive replication of viruses in macrophages.

We next set out to examine whether this scenario also occurs *in vivo*. WT and *Adap*^*-/-*^ mice were either mock-infected or infected with PR8 strain of IAV. 7 days post infection, tissue sections from lung and spleen of the infected WT and *Adap*^*-/-*^ mice were then double-immunostained with anti-NP and antibody against F4/80, a pan macrophage marker for the virus antigen and macrophages, respectively. As shown in Fig 7A and 7B, upon IAV-PR8 infection, the percentage of macrophages positively stained for both F4/80 and NP protein were robustly higher (approximately 2-fold) in both lung and spleen from *Adap*^*-/-*^ mice compared to that from WT mice, confirming that macrophages from both lung and spleen of *Adap*^*-/-*^ mice are more susceptible to IAV infection. This was further supported by in-depth pathological analysis of the IAV-PR8 infected mice, which were performed in samples obtained at 7 dpi. Hematoxylin and eosin (H&E) staining revealed that the alveolar wall thickening and edema in the lungs following IAV infection were more severe in *Adap*^*-/-*^ mice than in WT mice (Fig 7C, upper panels). IAV infection also caused more severe necrotic foci in spleen, as indicated by the less clear borders of white pulp, red pulp and marginal zone in *Adap*^-/-^ mice compared to WT mice (Fig 7C, lower panels). Similar observation was attained with the SeV infection that resulted in more severe tissue damage in all lungs, spleens and livers of *Adap*^-/-^ mice than that of WT mice (Fig 7D). Thus, ADAP deficiency rendered macrophages more susceptible to RNA virus infection and replication *in vivo*.

## Discussion

Macrophages normally contribute to anti-viral immune responses, and the response of macrophages to virus infection is of critical importance to disease outcome. Meanwhile, macrophages are also important target cells permissive to infection with many RNA viruses [23,41]. However, the detailed characterization of how the viral replication in macrophages is regulated and how this directs macrophage antiviral response is not entirely understood. To this end, this study portrays a novel mechanism regulating virus replication in macrophages whereby ADAP exerts an agonistic activity in the type I IFN response of macrophages to RNA virus infection via cell-intrinsic control of RIG-I ISGylation. Our findings demonstrate ADAP selectively interacts with RIG-I but not MDA5, and the association is inducible upon viral infections in macrophages. ADAP suppresses ISGylation of RIG-I, and a loss of ADAP increases ISGylation of RIG-I, accompanied by a decrease in the production of LPS-induced type I IFN and ISG15. Using *Adap*^-/-^ mice, ADAP deficiency strongly increases the susceptibility of macrophages to RNA-virus infection *in vivo*.

Macrophages are the primary producer of type I IFNs, and the antiviral response of macrophages to RNA virus infection is strongly associated with high levels of IFNs [3,4]. ADAP deficiency substantially decreased the production of IFN-I as well as the ISG15 in macrophages upon SeV and VSV infection (Fig 1A–1C). Given that the key cytoplasmic dsRNA senser RIG-I is the major driver for IFN-I, the response of macrophages to RNA virus infection with an altered RIG-I signaling pathway would be anticipated upon ADAP depletion. Indeed, we showed ADAP selectively interacts with RIG-I but not MDA5, and, essentially, this interaction is inducible upon RNA virus infection, and mainly occurs in the mitochondria, suggesting that a functional interplay between ADAP and RIG-I in macrophages response to RNA virus infection.

While in most cases, ISG15 mediated ISGylation plays a key role in the host’s innate anti-viral immunity, our data showing an increased level of ISGylation of RIG-I caused by ADAP-deficiency seems an exception, which decreased the production of IFN-I. This is in line with the previous study showing ISGylation contributes to the decline in RIG-I signaling for the production of IFN-I in macrophages during RNA virus infection [42], namely, instead of promoting RIG-I activation, ISG15 conjugation of RIG-I negatively regulates RIG-I-mediated antiviral signaling [11]. Our data suggest the potential involvement of ADAP in the functional regulation of RIG-I for IFN-I signaling through controlling the ISGylation of RIG-I. *Adap*^*-/-*^ macrophages display an enhanced RIG-I ISGylation (Fig 4D–4F), whereas ectopic expression of ADAP reduced the RIG-I ISGylation *in vitro* (Fig 4A and 4B), indicating ADAP acts as an inhibitor for RIG-I ISGylation in macrophages. In contrast, the ubiquitination of RIG-I was hardly affected in the absence of ADAP. This is in line with the concept that ADAP functions as an intracellular immune checkpoint molecule to restrain the phagocytic ability of splenic macrophages [21]. Indeed, as expected, *Adap*^*-/-*^ macrophages exhibits higher level of RIG-I ISGylation, but a lower level of type I-IFNs and a reduced downstream TBK1 and IRF3 activation compared to the WT macrophages (Fig 5C and 5D). As a consequence, not only the iBMMs and RAW264.7 but also the primary lung macrophages became highly susceptible to the infection with RNA viruses such as IAV, SeV and VSV when ADAP was depleted or down-regulated. Thus, ADAP functions as an anti-viral factor in a type I IFN-dependent manner via dampening the ISGylation of RIG-I in macrophages.

Interestingly, in resting macrophages, RIG-I expression at both mRNA and protein levels was unaffected upon ADAP depletion, whereas in SeV infected macrophages, ADAP deficiency led to a significant decrease of RIG-I expression at protein level but not at the mRNA level (S1 Fig). Given that unlike ubiquitylation, ISGylation usually does not appear to directly target proteins for proteasome-mediated degradation [9,10], one possible explanation is that RIG-I stability may require its association with ADAP in a pattern similar to SKAP1, a bona fide ADAP interacting partner, whereby loss of ADAP causes SKAP1 degradation [43]. The observation that the assembly of ADAP-RIG-I complex can be induced by SeV infection (Fig 2B) indicates RIG-I stability could be more dependent on ADAP in macrophages in the context of viral infection than in resting context, contributing to explain why ADAP deficiency causes a significant decrease in RIG-I protein in SeV-infected macrophages but not in the resting macrophages. However, it remains to be seen exactly how the ADAP suppresses the ISGylation of RIG-I in the ADAP-RIG-I complex and whether or not the increased level of ISGylation of RIG-I influences its stability. The most likely explanation is that the binding of ADAP to RIG-I could confer RIG-I in a conformation unfavorable for ISGylation of RIG-I and or detrimental to the recruitment of ISGylation enzymes to the vicinity of RIG-I. Similar model could be found between Dax1 and ESRRB, whereby Dax1 binds to and functions as a negative regulator of Esrrb [44]. Investigation of such assumptions in detail would be of interest for future study.

ISGylation of RIG-I inhibits the expression of type I IFN and its mediated downstream signaling upon infection with RNA viruses [11]. In line with this, ADAP deficiency results in an increase in RIG-I ISGylation (Fig 4D–4F), thus leading to a decline in the production of type I IFN and ISG15 (Fig 1A–1C). Given that type I IFNs are key mediators in the anti-viral immunity of macrophages, the inhibitory effect of ADAP on the initial ISGylation of RIG-I is critical in the determination of permissiveness of macrophages to viruses. Indeed, using knockout mice, ADAP deficiency markedly increased viral burden in macrophages, pointing to a loss of ADAP renders the macrophages more permissive to RNA viruses. Together, our study is to our knowledge the first to demonstrate ADAP as an essential host macrophage permissive factor for RNA-virus replication in macrophages by regulation of RIG-I ISGylation whose alterations influence its downstream type I IFN signaling, and subsequently the permissiveness of macrophages to RNA viruses.

Suppressing the synthesis of type I IFNs is one of the key strategies for viruses to evade the host’s innate immunity [4]. For example, host’s IFN-β1 transcription can be inhibited by several SARS-CoV-2 proteins including NSP1, NSP3, NSP5, and ORF3a [45,46]. Our data demonstrate that repression of IFN-I signaling by ADAP deficiency allows for excess virus replication in macrophages *in vitro*, and loss of ADAP aggravates viral replication in macrophages *in vivo*, suggesting ameliorating viral replication in macrophages can be achieved via targeting the ADAP-RIG-I module.

In summary, our study identifies a novel functional interplay between ADAP and RIG-I in the control of RNA virus replication in macrophages in a model, wherein ADAP sustains the expression of type I IFN and it stimulated ISG15 via inhibiting the ISGylation of RIG-I (Fig 7E). Under-expression or loss of ADAP reduces type I IFN and its related antiviral gene expression, and renders macrophages more susceptible to RNA viruses.

## Material and methods

### Ethics statement

All animal experiments involved in this study were approved by the Ethics Committee of Soochow University (ECSU) (approval No. SUDA20231113A04) and carried out in accordance with the approved guidelines.

### Plasmids and transfection

The mammalian expressing plasmid pSRα-HA-ADAP has been described previously [19]. Mutants of RIG-I were generated by PCR and confirmed by DNA sequencing. pCAGGS-FLAG-DDX58, pCDNA3.1–6×His-ISG15 and CAGGS-HA-hUBE1Lwere purchased from Miaoling Bio. pCMV3-C-Myc-Ube2L6 was purchased from Sino Biological. Transfection of HEK293T cells were performed using Hieff Trans Liposomal transfection regent according to the manufactory’s instructions (Yeasen Biotechnology). Transient transfection of RAW264.7 cells was performed as described previously [20]. Briefly, 1×10^7^ RAW264.7 cells suspended in Opti-MEM medium (Life Technologies) were mixed with 10 μg of pSRα-HA-ADAP and electroporated by exponential pulse at 250 V and 950 mF with Gene Pulser Xcell (Bio-Rad Laboratories). Followed by electroporation, cells were recovered in room temperature for 10 min and cultured in DMEM supplemented with 10% FBS.

### Antibodies and reagents

The antibodies and reagents used in this study were commercially obtained from different sources, which were listed as follows: mouse anti-Influenza A NP, mouse anti-Influenza A NS1 and mouse anti-ISG15 (Santa Cruz); rabbit anti-F4/80 antibody and mouse anti-β-Tubulin (Abcam); rabbit anti-ISG15, rabbit anti-RIG-I, rabbit anti-MAVS, rabbit anti-MDA5, Alexa Fluor 488-cunjugated anti-Rabbit IgG (H+L), F(ab’)2 Fragment and anti-Mouse IgG (H+L), F(ab’)2 Fragment (Alexa Fluor 647 Conjugate), anti-rabbit IgG (H+L) (DyLight 680 Conjugate), anti-rabbit IgG (H+L) (DyLight 800 4X PEG Conjugate) and anti-mouse IgG (H+L) (DyLight 680 Conjugate) (CST); rabbit anti-RIG-I, mouse anti-Flag tag, mouse anti-AIF, rabbit anti-CD71, rabbit anti-Flotillin 1 and mouse anti-GAPDH (Proteintech); rabbit anti-ADAP (Millipore); mouse anti-ADAP (BD); mouse anti-HA tag (Sigma-Aldrich); IFN-β Protein (Sino Biology); LPS derived from Escherichia coli strain O111:B4 (Sigma-Aldrich); poly (I:C) (Selleck Chemicals). Mouse anti-viral protein (VP3) of IBDV polyclonal antibody was generated from the sera of mice by immunization with purified IBDV.

### Mice

The *Adap*^-/-^ mice was generated as previously described [47], which was a gift from C.E. Rudd (University of Cambridge, Cambridge, U.K.). Wild type (WT) C57BL/6 mice were obtained from GemPharmatech (Nanjing, China). Mice were bred under specific pathogen-free conditions at Animal Centre of Soochow University. Sex and age matched mice between 6 and 8 weeks were used.

### Cells and viruses

Immortalized bone marrow-derived macrophages (iBMMs) were generated as previously described [48]. Briefly, bone marrow cells were isolated and cultured in J2-infected RPMI 1640 medium containing 10% FBS (Serana Europe, Pessin, Germany), 100 U/ml penicillin-streptomycin (Beyotime), 100ng/ml M-CSF (Peprotech) for 18h. Adherent cells were separated and incubated in J2-infected RPMI 1640 complete medium supplemented polybrene and M-CSF for additional 6 days. Following that, cells were cultured in RPMI1640 complete medium supplemented with 20% L929 cell culture supernatant and propagated for more than 20 passages until immortalization. Mouse primary bone marrow-derived macrophages (BMDMs) were prepared as previously described [21]. RAW264.7, HEK293T, MDCK and A549 cell lines were obtained from American Type Culture Collection (Manassas, VA) and maintained in DMEM supplemented with 10% FBS and 100 U/ml penicillin-streptomycin.

The H1N1 vaccine strain A/PR/8/1934 was obtained from American Type Culture Collection (Manassas, VA) and grown in MDCK cells. The IBDV attenuated strain HZ2 that was adapted to growth in DF-1 cells as previously described [49]. Sendai virus (SeV) kindly provided by Prof. Jianfeng Dai (Soochow University) [50], was grown in embryonated chicken eggs. VSV-GFP was grown in Vero cells. C57BL/6 mice were infected with PR8 or DMEM or SeV by nasal drip 7 days before sacrificed. Viral infections (IAV vaccine strain PR8 strain, SeV and IBDV) were performed in accordance with biosafety regulations, using biosafety level 2 containment laboratories approved for such use by the local authorities. The PR8 viral titration assay was reported previously [51]. Briefly, MDCK cells are infected with a series of diluted viruses. After 1 h, the medium was removed and replaced with a low melting point agarose layer, and cultured at 37°C according to the plaque results MOI.

### RNA-sequencing analysis

Peritoneal macrophages of WT and *Adap*^-/-^ mice were left uninfected or infected with SeV 12 h. Total RNA was extracted by TRIzol reagent (Sigma-Aldrich), and the integrity and quantity of RNA were examined with an Agilent Bioanalyzer 2100 (Agilent Technologies). A sequencing library was prepared using the NEBNext Ultra RNA Library Prep Kit for Illumina (New England Biolabs) according to the manufacturer’s instructions. Quality of library was determined using DNA 1000 assay kit (Agilent Technologies). RNA-seq was performed at Guangzhou Genedenove Corp.

### Confocal microscopy

Tissues or cells were fixed with 4% paraformaldehyde. Tissues were further cryo-sectioned post 10%, 20% and 30% sucrose dehydration by Cryostat Microtome (Leica, CM1950). Tissues or cells were permeabilized with 0.2% Triton X-100 in PBS 30 min and blocked with 5% goat serum for 1 h, followed by staining with indicated primary and secondary antibodies and DAPI. Tissues and cells were mounted with anti-fade mounting medium (Beyotime) and observed with a Zeiss confocal microscope (LSM800, Zeiss). The fluorescence intensity profile of the indicated proteins was processed using Image J. Mitochondria staining was performed according to the manufacturer’s instructions (Invitrogen, M7510). Briefly, cells were incubated in RPMI1640 containing 50 nM MitoTracker Orange CMTMRos for 30 min, washed twice and performed normal staining process as mentioned above.

### Hematoxylin and eosin (H&E) staining

H&E staining was performed using H&E staining kit according to the manufacturer’s instructions (Beyotime, C0105). Briefly, cryo-sections were stained by Hematoxylin for 8 min followed by 10 min running water wash, and then stained by Eosin for 1 min. Sections were sequentially washed with 70%, 80%, 90% and 100% ethanol and hyalinized by Xylene for 5 min, and finally mounted by mounting media (Beyotime, P0126). Images were required with upright microscope (Elipse Ni, Nikon). The H&E score of lesions in lung were assessed based on the criterium of the extent of denatured and collapsed epithelial cells, degeneration of alveoli pneumocytes, infiltration of inflammatory cells, edema, hemorrhage, exudation and expansion of parenchymal wall, each item was scored for 0, normal; 1, mild; 2, moderate; 3, marked. The H&E score of lesions in spleen of viral-infected animals and mock-infected controls was assessed based on the severity and abundance of lesions with large necrotic areas and disorganised histologic appearance in a range from 0 to 5 (0 = none/minimal; 1 = mild; 2 = moderate; 3 = severe; 4 = markedly severe). Liver lesions were assessed in accordance with the criteria based on cellular morphology of mouse liver lesions [52]. The main lesions observed were classified and eventually scored (0: none/minimal; 1: mild; 2: moderate; 3: severe) as: foci of necrosis, portal, and lobular inflammation/immune cells infiltration, anisocytosis, and anisokaryosis. The cumulative H&E scores represent the total score per mice.

### Sucrose-gradient centrifugation

Sucrose-gradient centrifugation was performed for subcellular fractionation as described in [53] with minor modifications. Briefly, Cells were harvested and lysed in lysis buffer (1% Triton X-100 [v/v] in 25 mM Tris-HCl [pH 8.0], 140 mM NaCl, 1 mM EDTA, 1 mM PMSF, and 1 mM Na_3_VO_4_ and 0.1% protease inhibitor mixture solution [Roche]) at 4°C 30 min. The lysate was adjusted to 1.8 ml of 40% (v/v) sucrose/lysate mixture, and then layered 2.2 ml 30% and 2.2 ml 5% sucrose on the top. Samples were centrifuged at 368,000 g for 18 h at 4°C using an SW55Ti rotor (Beckman, Optima L-80 XP Ultracentrifuge). Fractions of 0.4 ml were collected from the top of the gradient and analyzed by immunoprecipitation and Western blotting.

### Immunoprecipitation and immunoblotting

Cell lysis, immunoprecipitation, and detection were performed as described previously with minor modifications. Briefly, cells were lysed in cell lysis buffer (1% Triton X-100 [v/v] in 20 mM Tris-HCl [pH 8.3], 150 mM NaCl, and 0.1% protease inhibitor mixture solution [Roche]) at 4°C 30 min. For immunoprecipitation, cell lysate was incubated with indicated antibodies or IgG isotype control at 4°C 1 h, followed by the addition of 25 μl protein G Sepharose bead slurry (GE Healthcare) at 4°C overnight. Beads were washed with lysis buffer three times and boiled in sample buffer for dissociating proteins. Proteins were resolved by SDS-PAGE and transferred to nitrocellulose membranes. Membranes were blocked with 5% milk and incubated with indicated primary antibodies and secondary antibodies. Protein expression was detected with Odyssey Imaging Systems (LI-COR) or enhanced chemiluminescence (ECL) reagents (New Cell & Molecular Biotech, P10300).

### Quantitative real-time PCR

Total RNA was extracted from tissues or cells using Trizol Reagent (Sigma-Aldrich) according to the manufacturer’s instructions. The first-strand cDNA was synthesized using Hifair II 1^st^ Strand cDNA Synthesis kit (Yeasen Biotechnology), and quantitative real-time PCR was carried out using Hieff Quantitative PCR SYBR Green Master Mix (Yeasen Biotechnology) on QuantStudio Design and Analysis System (Applied Biosystems, Foster City, CA). Relative expression levels were calculated by a standard ΔΔC_T_ method and normalized to the housekeeping gene. Primers were synthesized by Sangon Biotech (Shanghai, China), and primer sequences are available on request.

### MS-LC/MS

Cell lysates and precipitates were prepared as described in immunoprecipitation and immunoblotting. Proteins of interest were dissociated from beads by 8 M urea, followed by reduction, alkylation and digestion according to standard protocols [54]. Tryptic extracts were collected, lyophilized and resuspended in 0.1% formic acid and tested on Linear ion trap Orbitrap combined mass spectrometer (LTQ Orbitrap Elite ETD) (ThermoFishser) for liquid chromatography–tandem mass spectrometry (LC-MS/MS) analysis.

### Plaque assay

Plaque assay was performed by applying supernatants from IAV-PR8 infected WT or *Adap*^*-/-*^ iBMMs in different times (0 h, 12 h, 18 h, 24 h, 48 h) to infect monolayers of MDCK cells seeded in 12-well plates. The MDCK cells were inoculated with the supernatants from IAV-PR8 infected WT or *Adap*^*-/-*^ iBMMs in serum-free DMEM. After 1 h incubation at 37°C, discard the supernatants and overlay immobilizing medium using a 1:1 mix of 2×DMEM with 2% FBS and 2 μg/ml TPCK and low melting temperature agarose. After addition of the overlay, the plate was incubated in 37°C incubator. After 72 h post incubation, cells were fixed with 4% paraformaldehyde for 1 h and stained with 1% crystal violet for 15 min. Plaque numbers were recorded after rinsing the plates with deionized water.

### Transfection and luciferase reporter assays

HEK293T cells plated in 24-well plates were cotransfected with reporter plasmid IFN-β-luc (125 ng), renilla luciferase plasmid pRL-TK (25 ng), together with vector alone or ADAP、RIG-I and MDA5. The transfections were performed by using Lipofectamine 3000 (Yeasen). 48 h later, cells were lysed and the luciferase activity was determined using a dual-luciferase reporter assay system (Promega) according to the manufacturer’s instructions. The data were determined by normalization of the firefly luciferase activities to the renilla luciferase activities.

### Lentiviral transduction

ADAP knockdown and reconstitution RAW264.7 cells were established by lentivirus infection. The cDNA of shRNAs or full-length mouse ADAP were encoded by psPAX2 and pMD2.G into HEK293T cells and were delivered to target cells by lentiviral transduction in the presence of 8 μg/ml polybrene. Cells were selected with puromycin for at least 3 weeks prior to experimental use [21].

### Statistical analysis

All data were analyzed with Prism 8 (GraphPad Software). Differences between two group means were analyzed using an unpaired Student t-test or the Mann–Whitney U test for nonparametric data. A one-way or two-way ANOVA followed by correction for multiple comparisons was used to compare more than two groups. Wherever indicated, *p* values are as follows: *p < 0.05, **p < 0.01, ***p < 0.001, ****p < 0.0001. Data are shown as mean ± SEM.

## Supporting information

S1 Fig The effect of ADAP deficiency on RIG-I expression at both mRNA and protein levels in resting macrophages and in SeV infected macrophages. (A) The RIG-I bands from both WT and *Adap*^*-/*-^ macrophages under conditions of resting (n = 6) and SeV infection (n = 3) were quantified across multiple independent experiments including those in Figs 4 and 5. Bars represent the mean ± SEMs of at least three independent experiments. n.s., not significant (*p* > 0.05). *p < 0.05. (B) RNA-seq data showed ADAP deficiency did not affect RIG-I mRNA level in mock and SeV infected macrophages. The total RNA isolated from WT and *Adap*^-/-^ mice (n = 3) peritoneal microphages that were infected with SeV, or mock-infected was subjected to RNA-sequencing. Expression heatmap (lower panel) represent count value of *RIG-I* gene between WT and *Adap*^-/-^ peritoneal macrophages in response to SeV infection. (C) qRT-PCR analysis of expression levels of RIG-I with the total RNA isolated from WT and *Adap*^-/-^ mouse BMDMs that were infected with SeV, or mock-uninfected. Relative values of qPCR data were normalized to the HPRT expression. The fold change was normalized to that of uninfected WT mice. Bars represent the mean ± SEMs of the three independent experiments (n = 3).
(TIF)

S1 File The raw data of IF and HE in this study. (ZIP)

S1 Data The raw data of Western blot and the underlying numerical data and statistical analysis in this study. (XLSX)

S1 Table The mass spectrometry proteomics data for Fig 3B. (XLSX)
