# Supplementary figures and images for: Dampening of ISGylation of RIG-I by ADAP regulates type I interferon response of macrophages to RNA virus infection (part 1 of 2)
Source: PLoS Pathog. 2024 May 22;20(5):e1012230. doi: 10.1371/journal.ppat.1012230 (PMC11111093; doi:10.1371/journal.ppat.1012230)

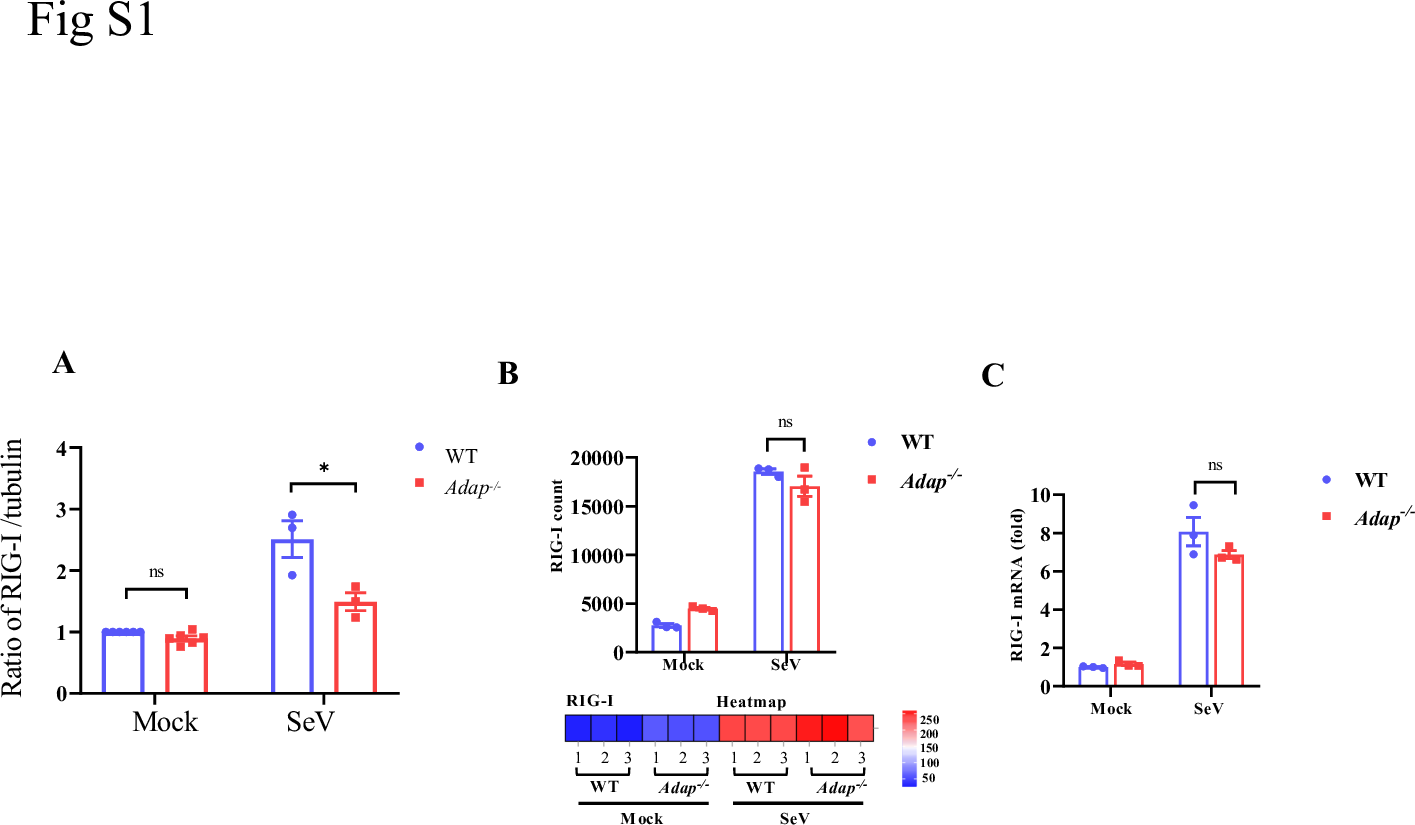

Supplement: S1 Fig — (A) The RIG-I bands from both WT and Adap-/- macrophages under conditions of resting (n = 6) and SeV infection (n = 3) were quantified across multiple independent experiments including those in Figs 4 and 5. Bars represent the mean ± SEMs of at least three independent experiments. n.s., not significant (p > 0.05). *p < 0.05. (B) RNA-seq data showed ADAP deficiency did not affect RIG-I mRNA level in mock and SeV infected macrophages. The total RNA isolated from WT and Adap-/- mice (n = 3) peritoneal microphages that were infected with SeV, or mock-infected was subjected to RNA-sequencing. Expression heatmap (lower panel) represent count value of RIG-I gene between WT and Adap-/- peritoneal macrophages in response to SeV infection. (C) qRT-PCR analysis of expression levels of RIG-I with the total RNA isolated from WT and Adap-/- mouse BMDMs that were infected with SeV, or mock-uninfected. Relative values of qPCR data were normalized to the HPRT expression. The fold change was normalized to that of uninfected WT mice. Bars represent the mean ± SEMs of the three independent experiments (n = 3). (TIF) [file ppat.1012230.s001.tif]

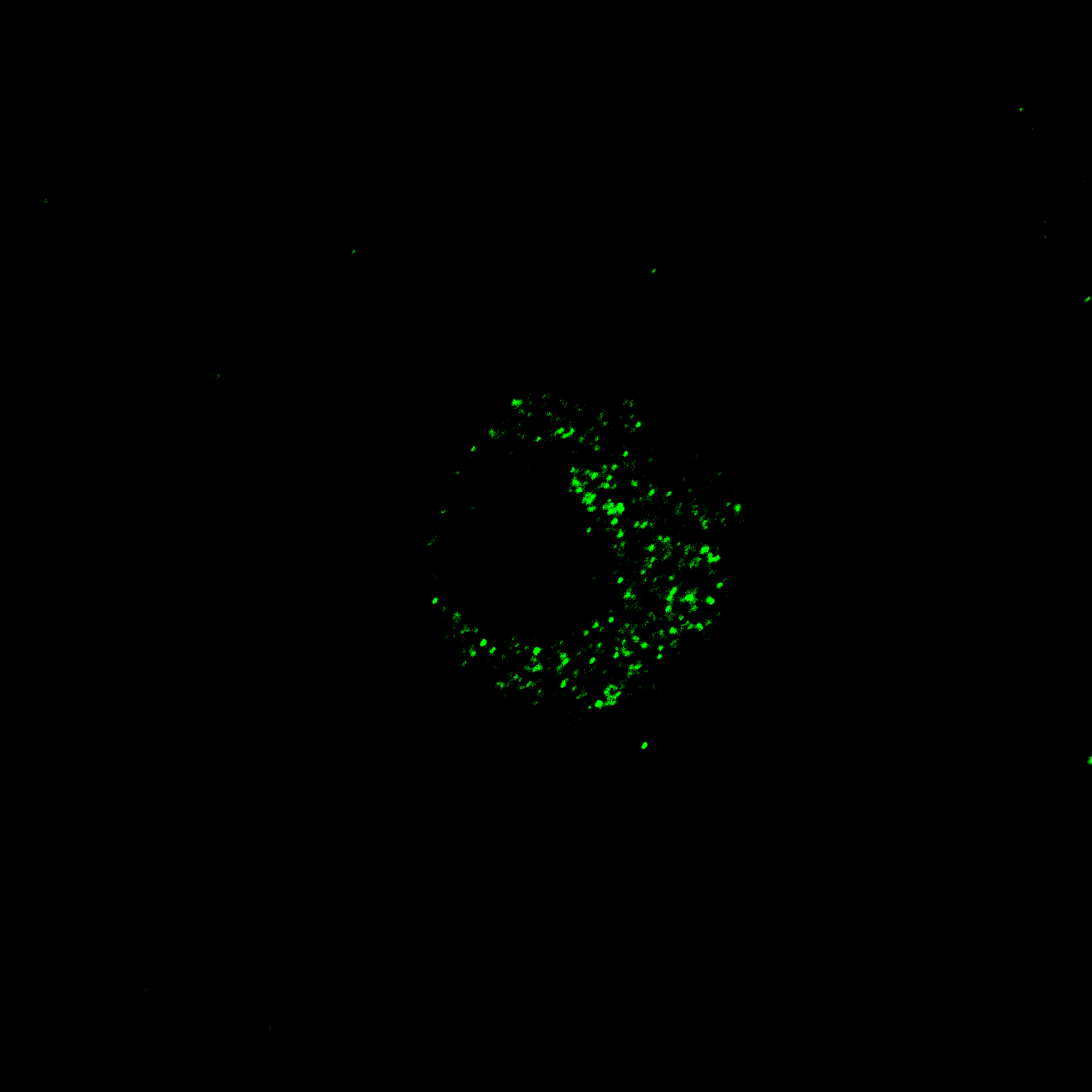

Supplement: S1 File — (ZIP) [file ppat.1012230.s002.zip › S1_File/Fig_3D/LPS/LPS-ADAP-1.tif]

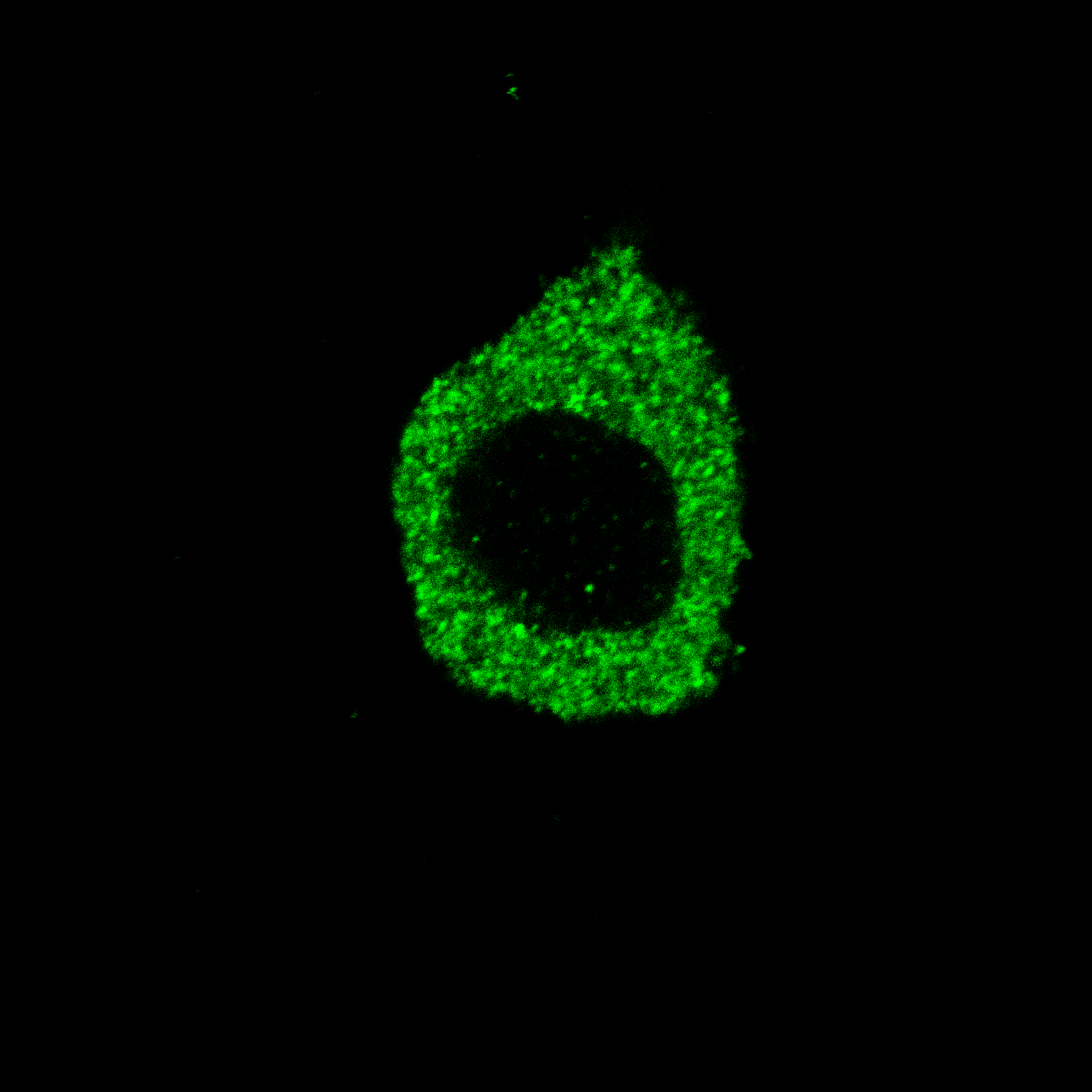

Supplement: S1 File — (ZIP) [file ppat.1012230.s002.zip › S1_File/Fig_3D/LPS/LPS-ADAP-2.tif]

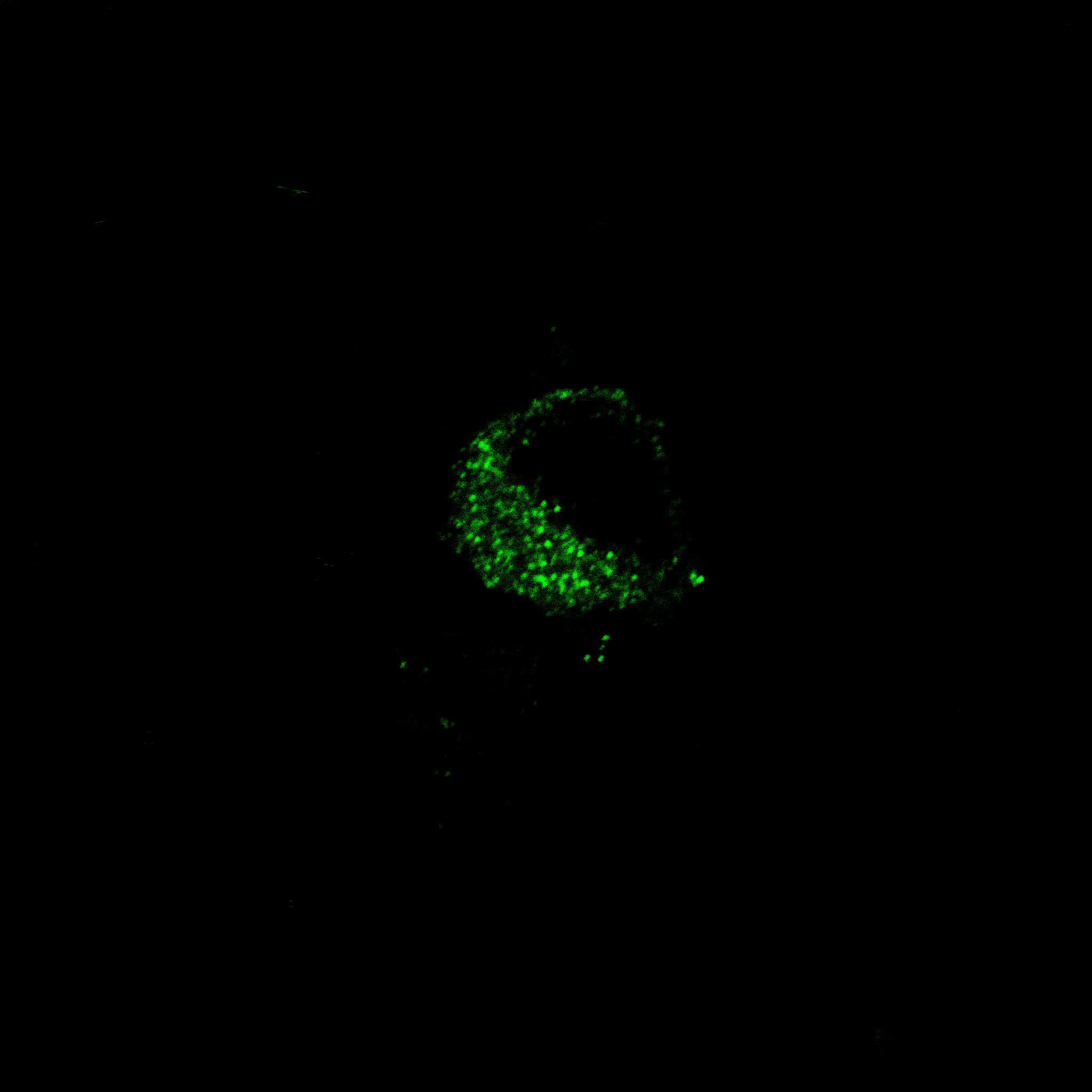

Supplement: S1 File — (ZIP) [file ppat.1012230.s002.zip › S1_File/Fig_3D/LPS/LPS-ADAP-3.tif]

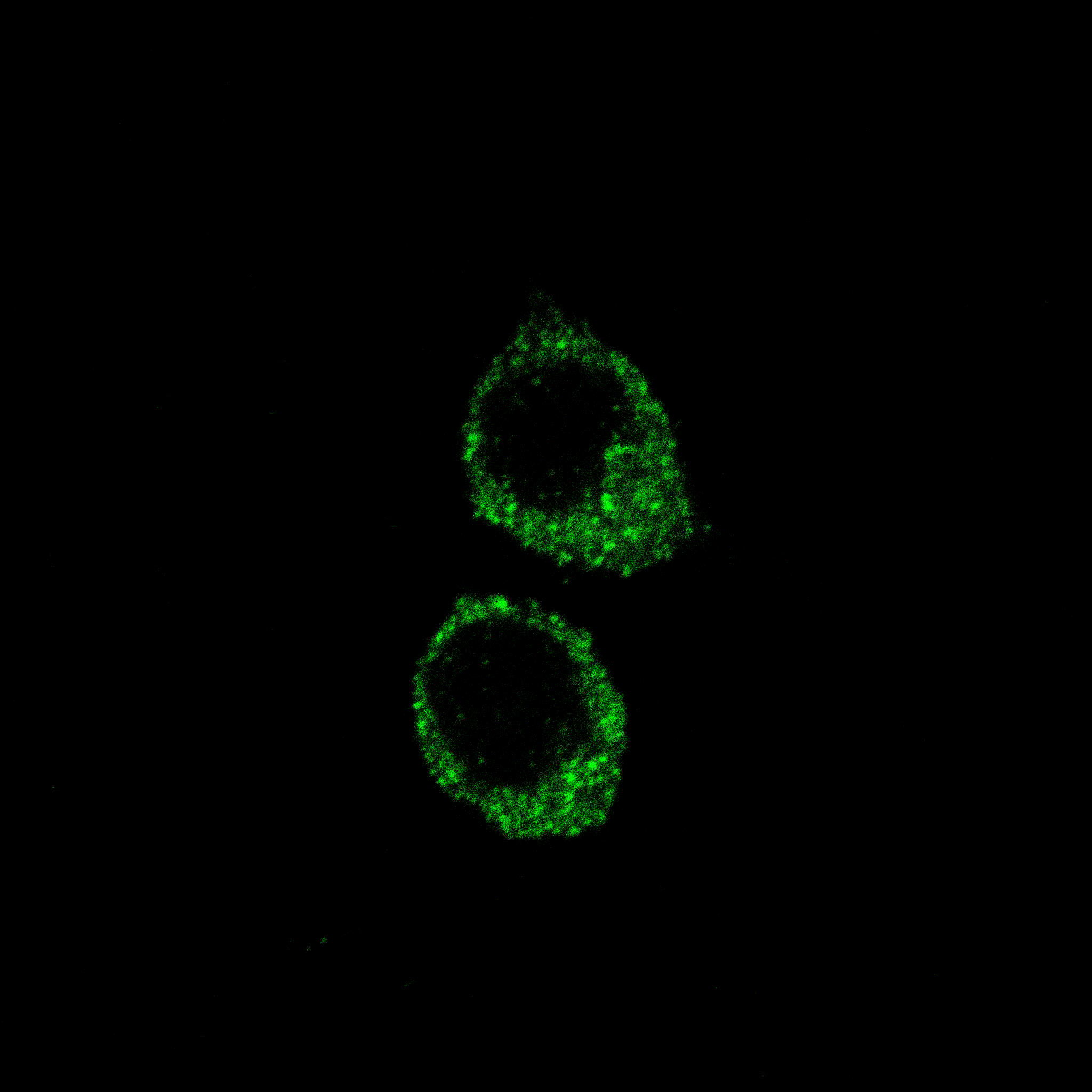

Supplement: S1 File — (ZIP) [file ppat.1012230.s002.zip › S1_File/Fig_3D/LPS/LPS-ADAP-4.tif]

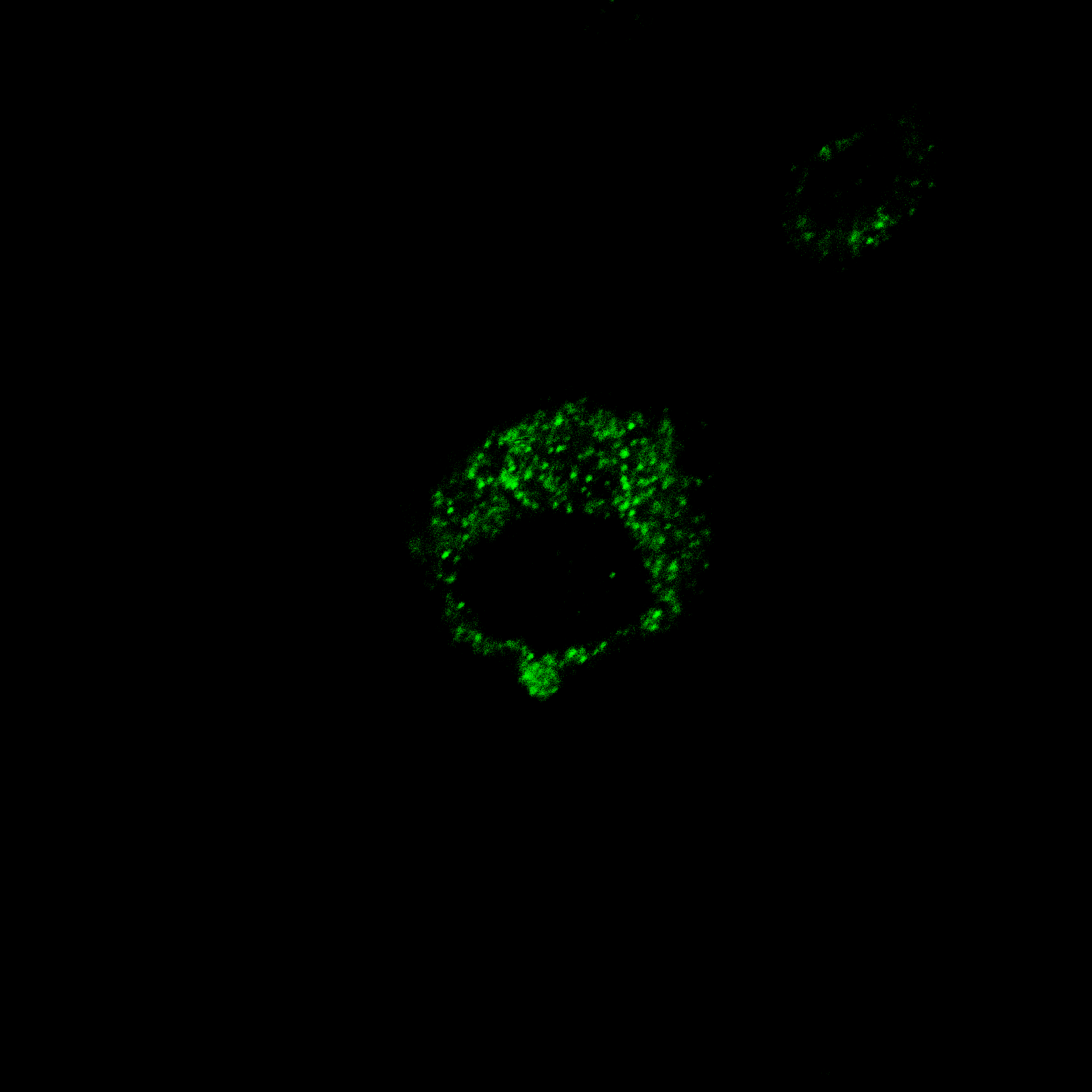

Supplement: S1 File — (ZIP) [file ppat.1012230.s002.zip › S1_File/Fig_3D/LPS/LPS-ADAP-5.tif]

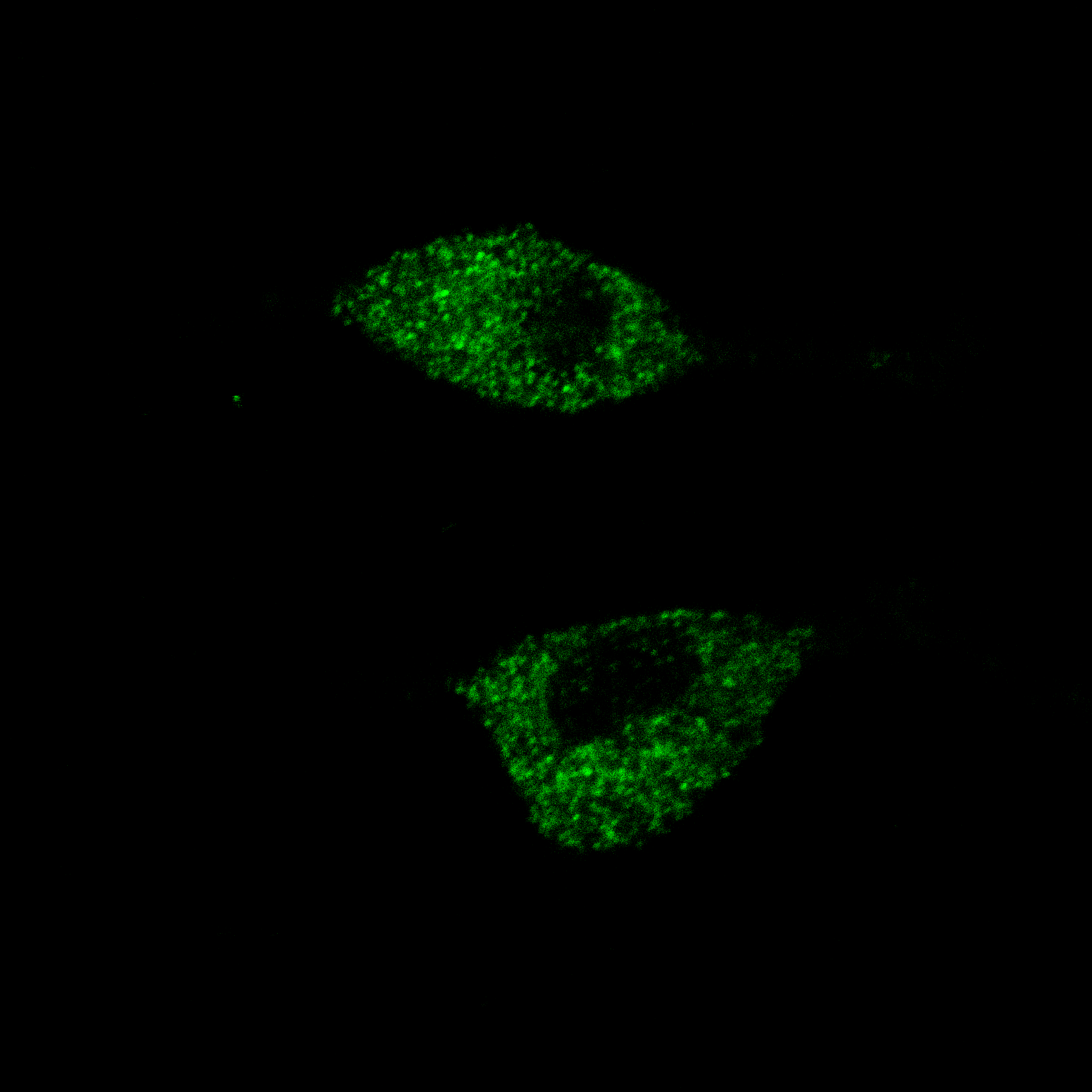

Supplement: S1 File — (ZIP) [file ppat.1012230.s002.zip › S1_File/Fig_3D/LPS/LPS-ADAP-6.tif]

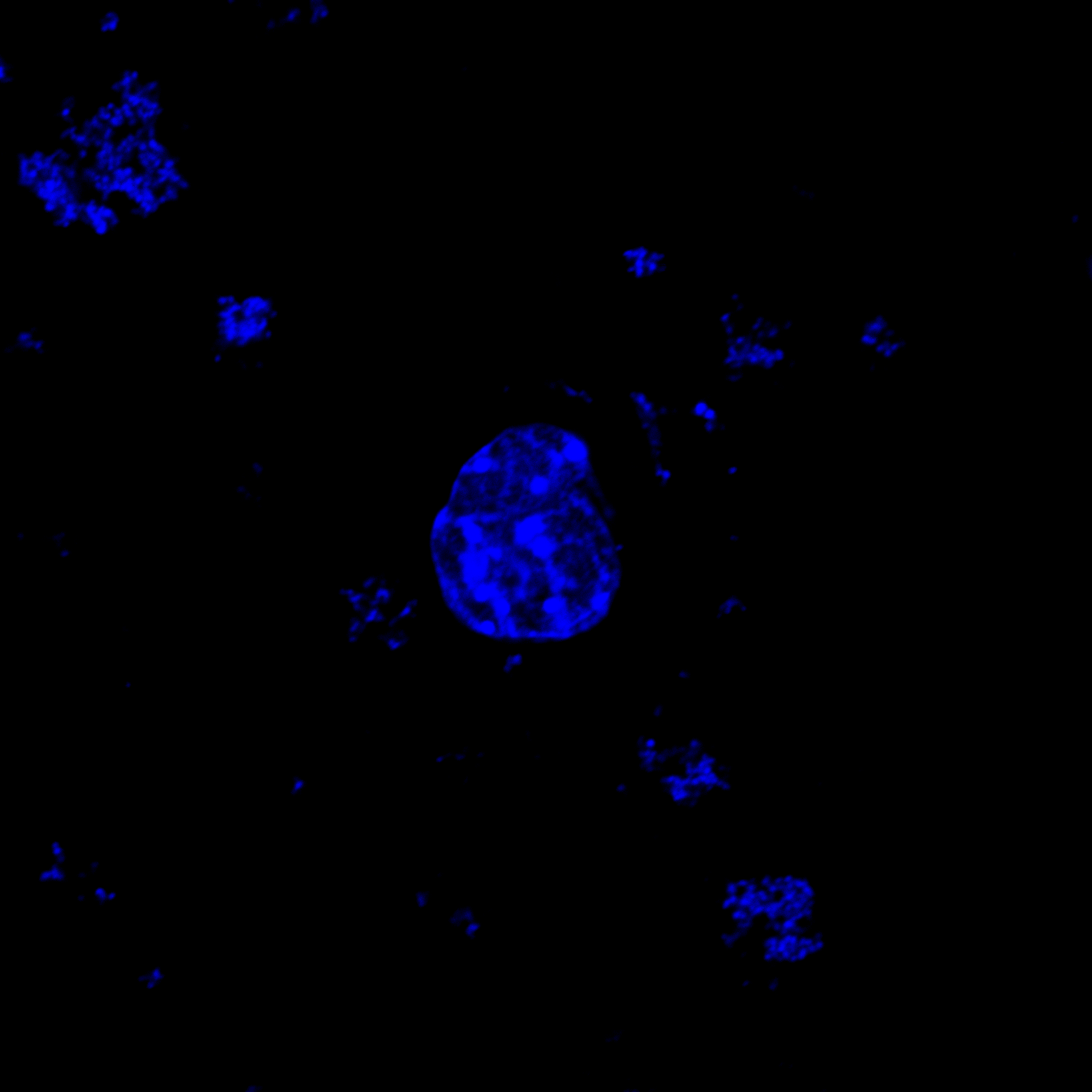

Supplement: S1 File — (ZIP) [file ppat.1012230.s002.zip › S1_File/Fig_3D/LPS/LPS-DAPI-1.tif]

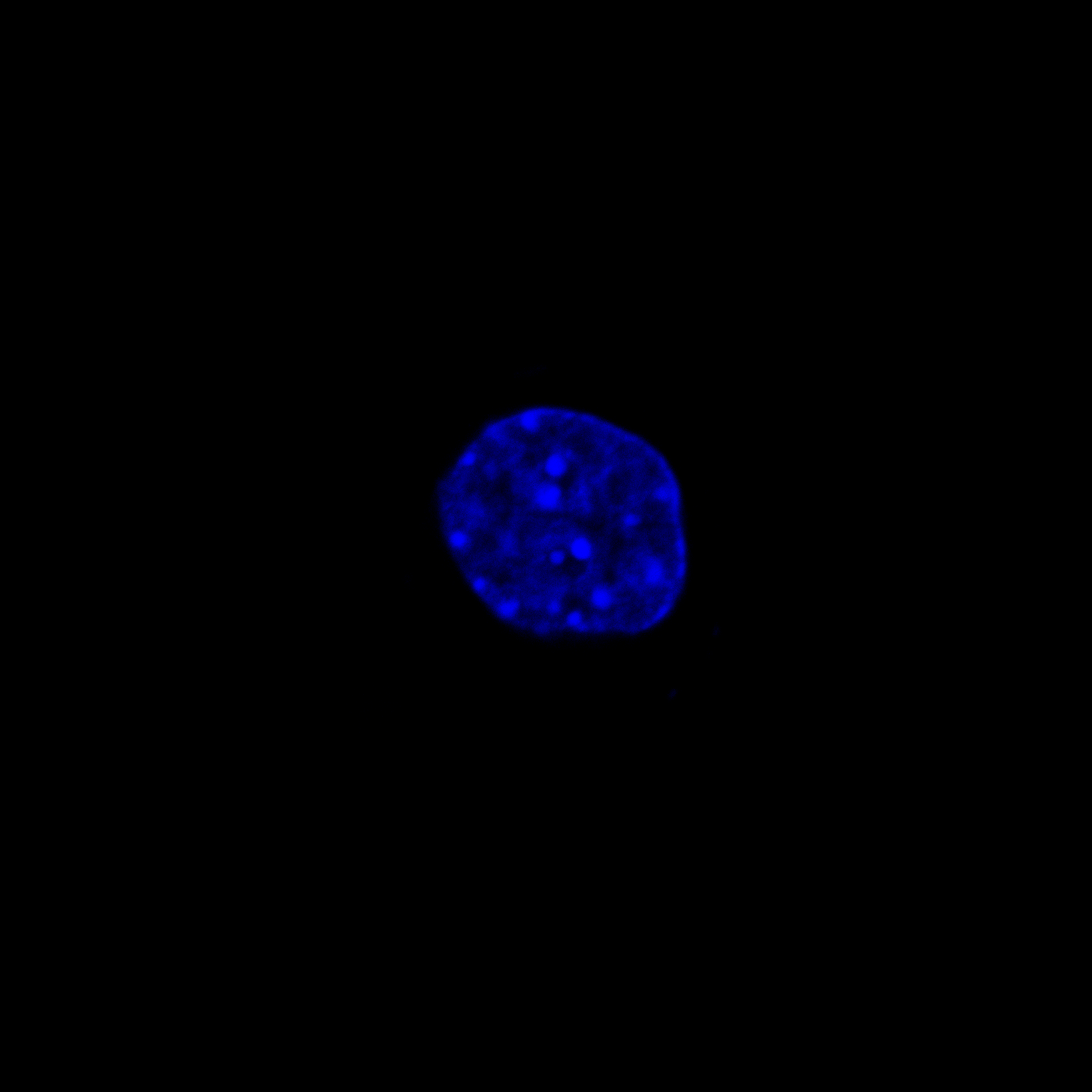

Supplement: S1 File — (ZIP) [file ppat.1012230.s002.zip › S1_File/Fig_3D/LPS/LPS-DAPI-2.tif]

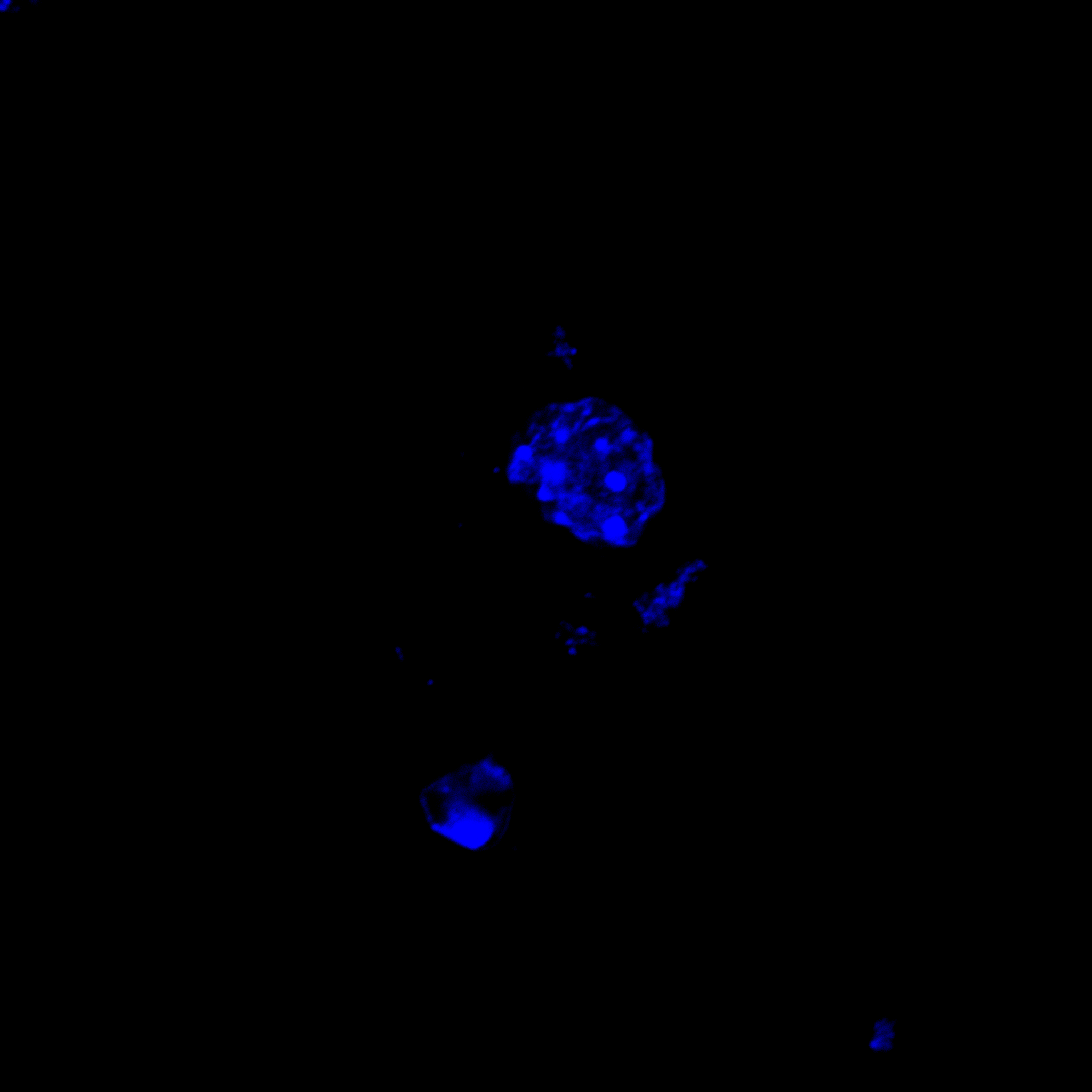

Supplement: S1 File — (ZIP) [file ppat.1012230.s002.zip › S1_File/Fig_3D/LPS/LPS-DAPI-3.tif]

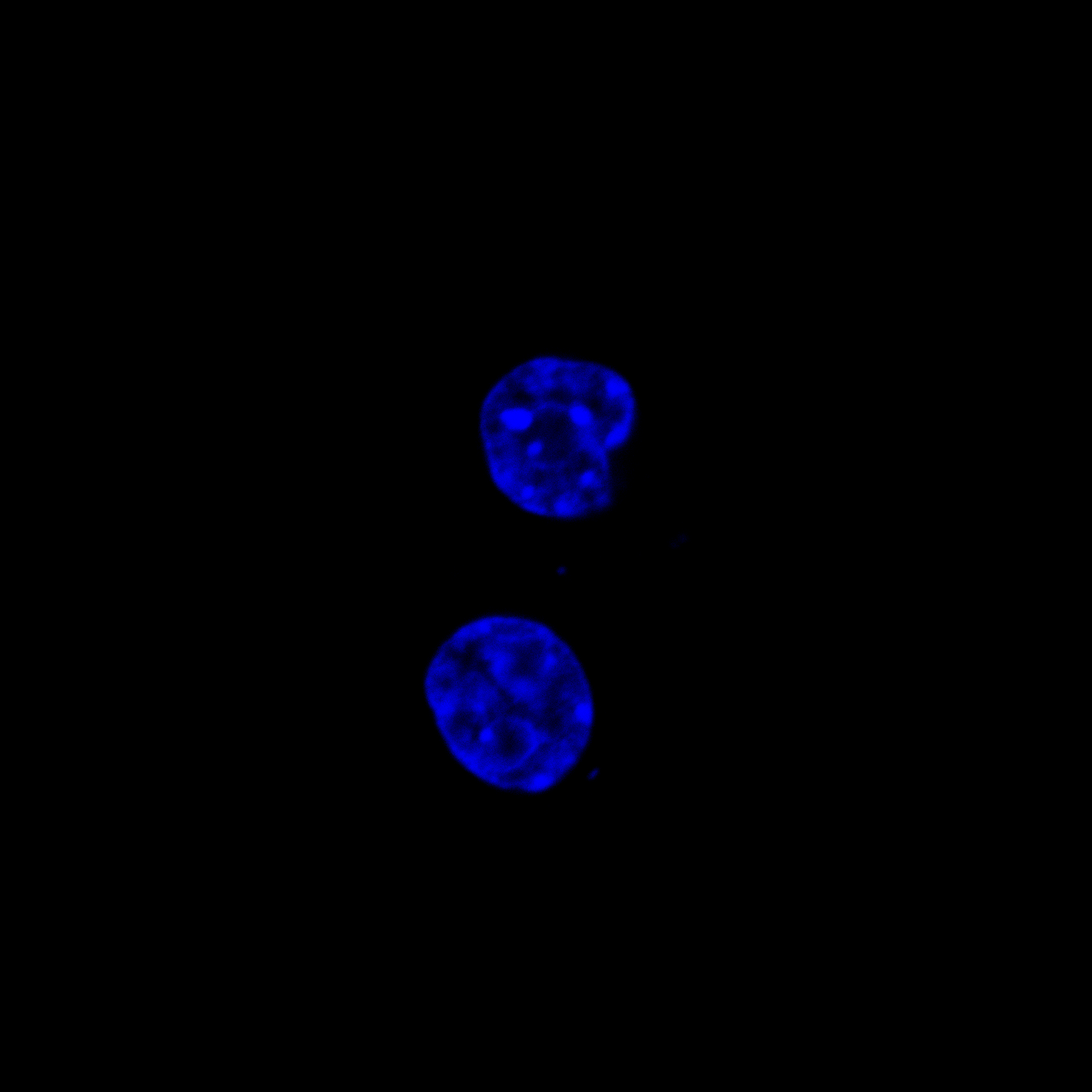

Supplement: S1 File — (ZIP) [file ppat.1012230.s002.zip › S1_File/Fig_3D/LPS/LPS-DAPI-4.tif]

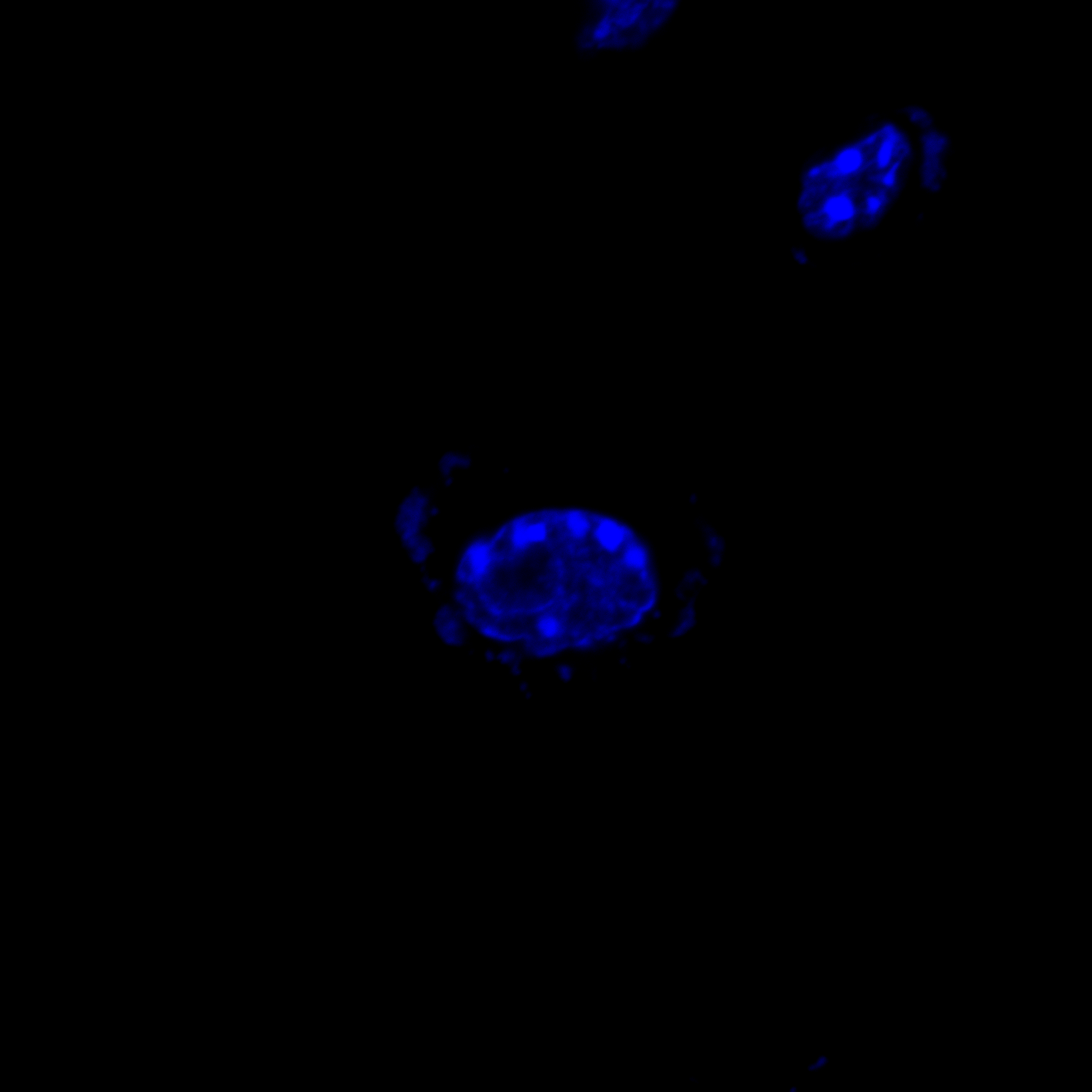

Supplement: S1 File — (ZIP) [file ppat.1012230.s002.zip › S1_File/Fig_3D/LPS/LPS-DAPI-5.tif]

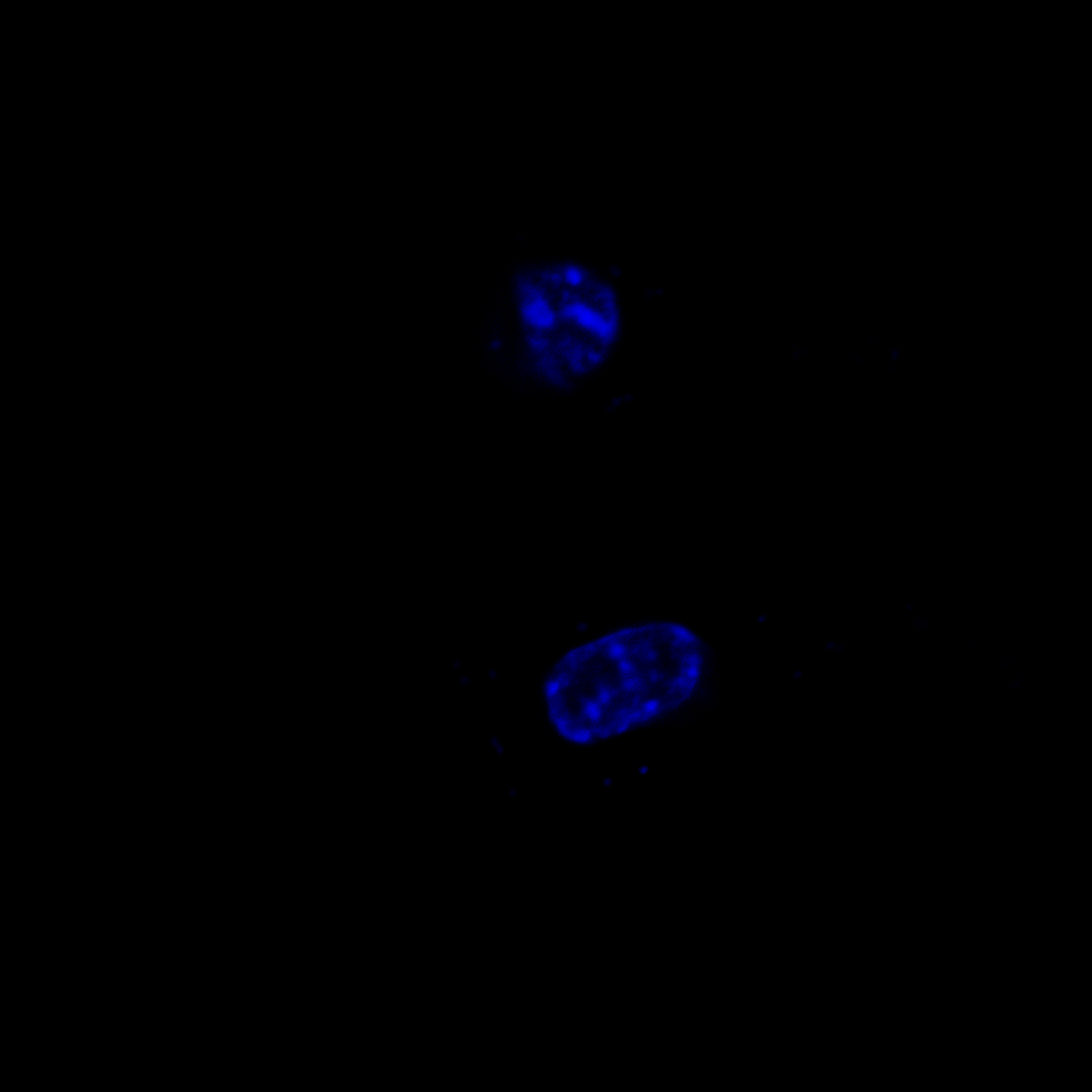

Supplement: S1 File — (ZIP) [file ppat.1012230.s002.zip › S1_File/Fig_3D/LPS/LPS-DAPI-6.tif]

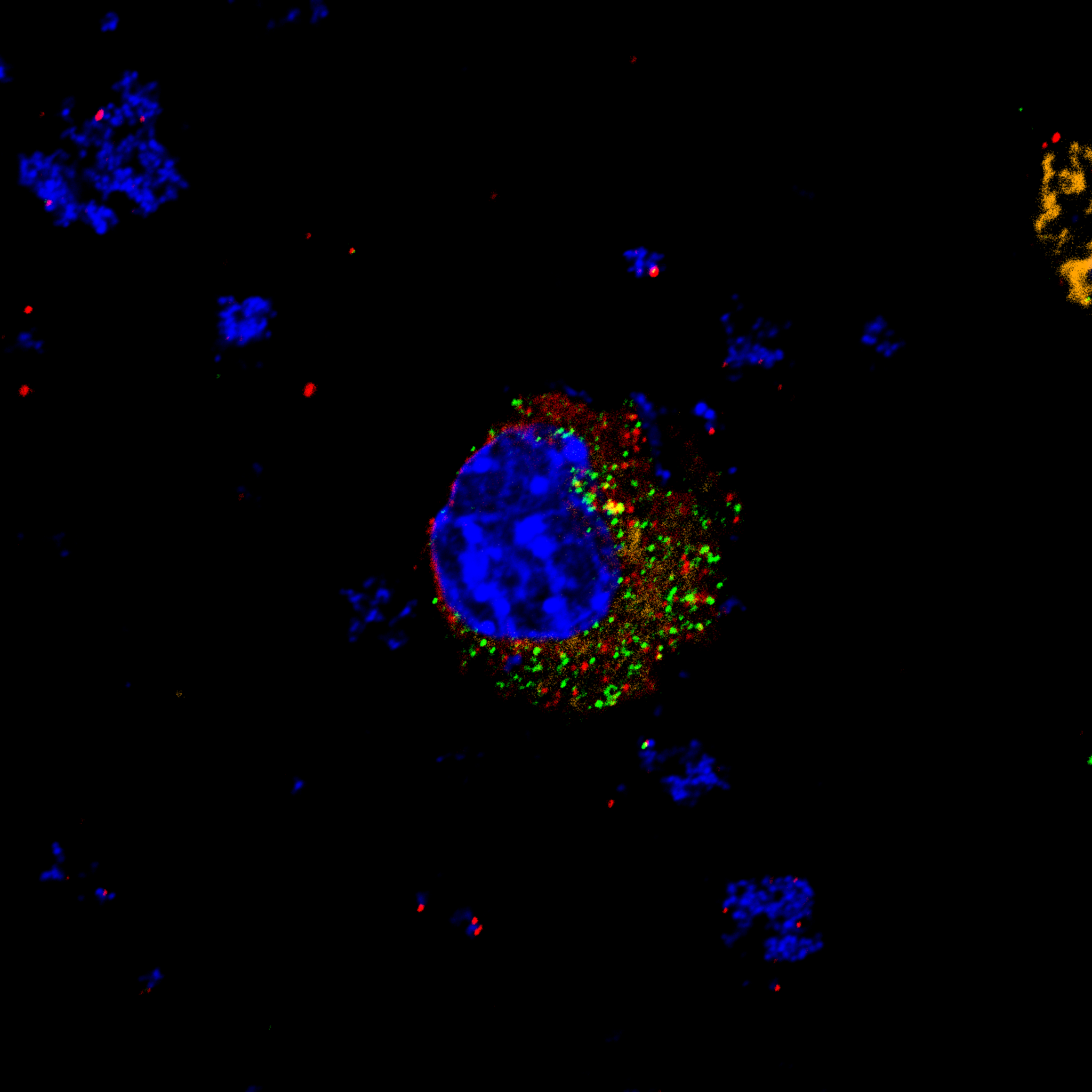

Supplement: S1 File — (ZIP) [file ppat.1012230.s002.zip › S1_File/Fig_3D/LPS/LPS-merge-1.tif]

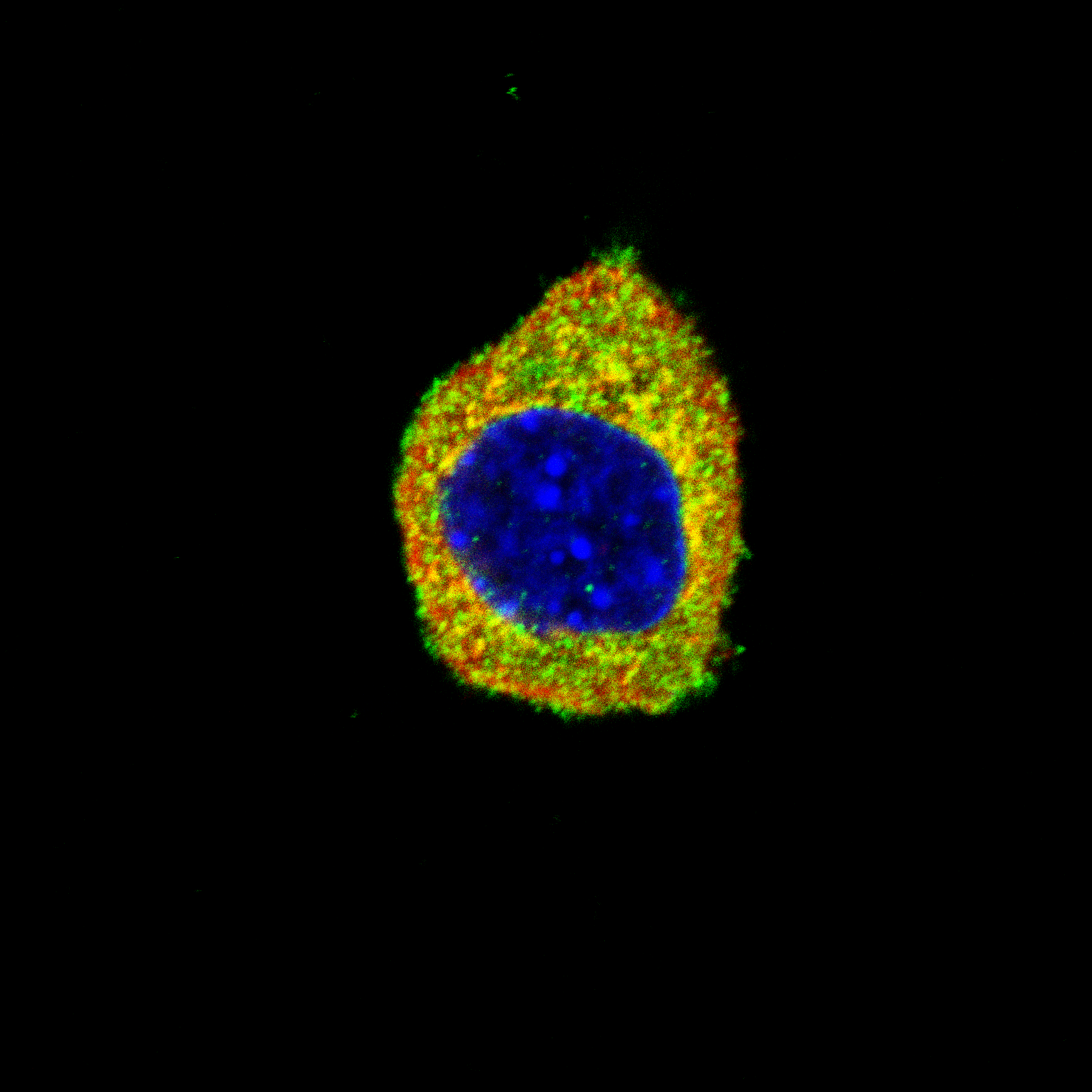

Supplement: S1 File — (ZIP) [file ppat.1012230.s002.zip › S1_File/Fig_3D/LPS/LPS-merge-2.tif]

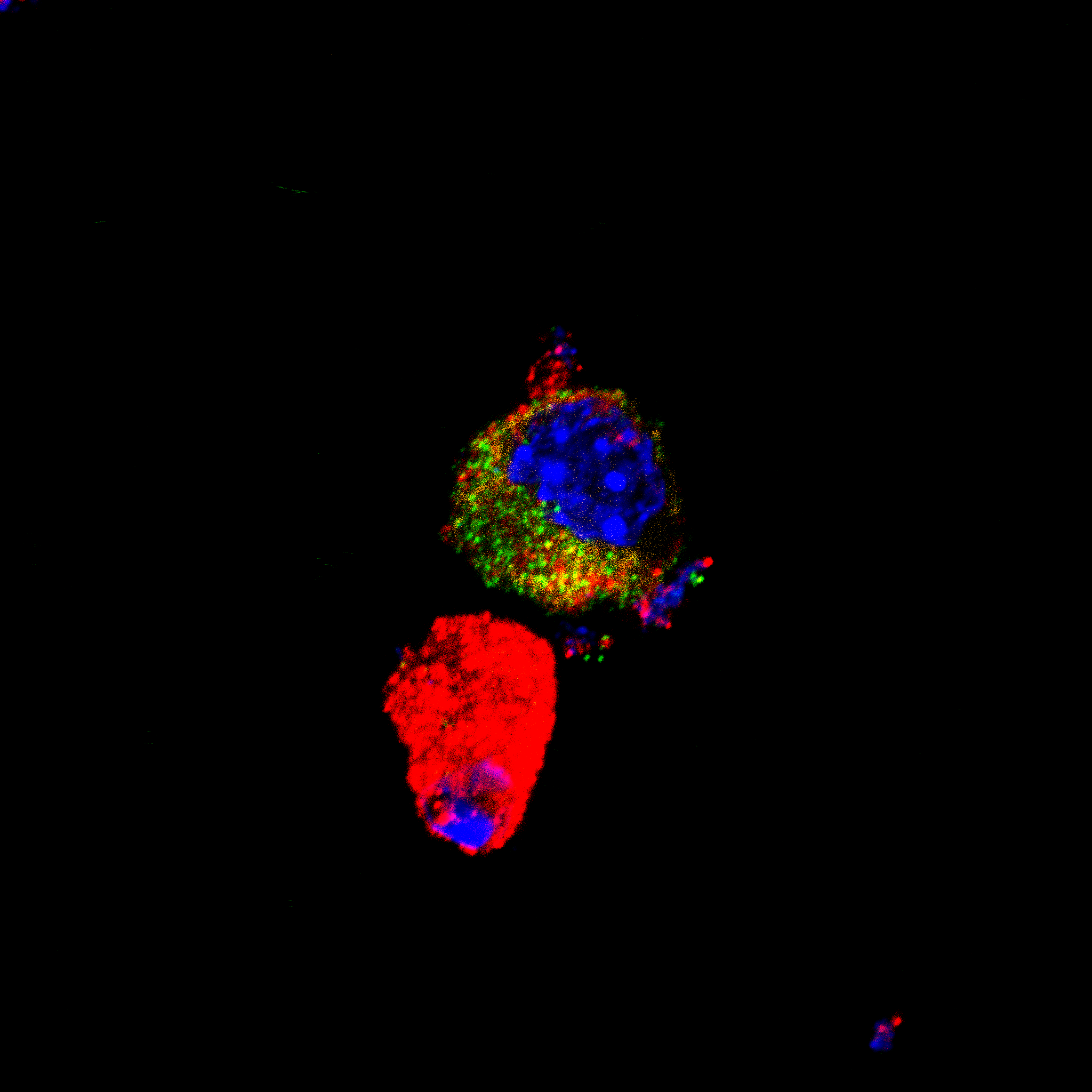

Supplement: S1 File — (ZIP) [file ppat.1012230.s002.zip › S1_File/Fig_3D/LPS/LPS-merge-3.tif]

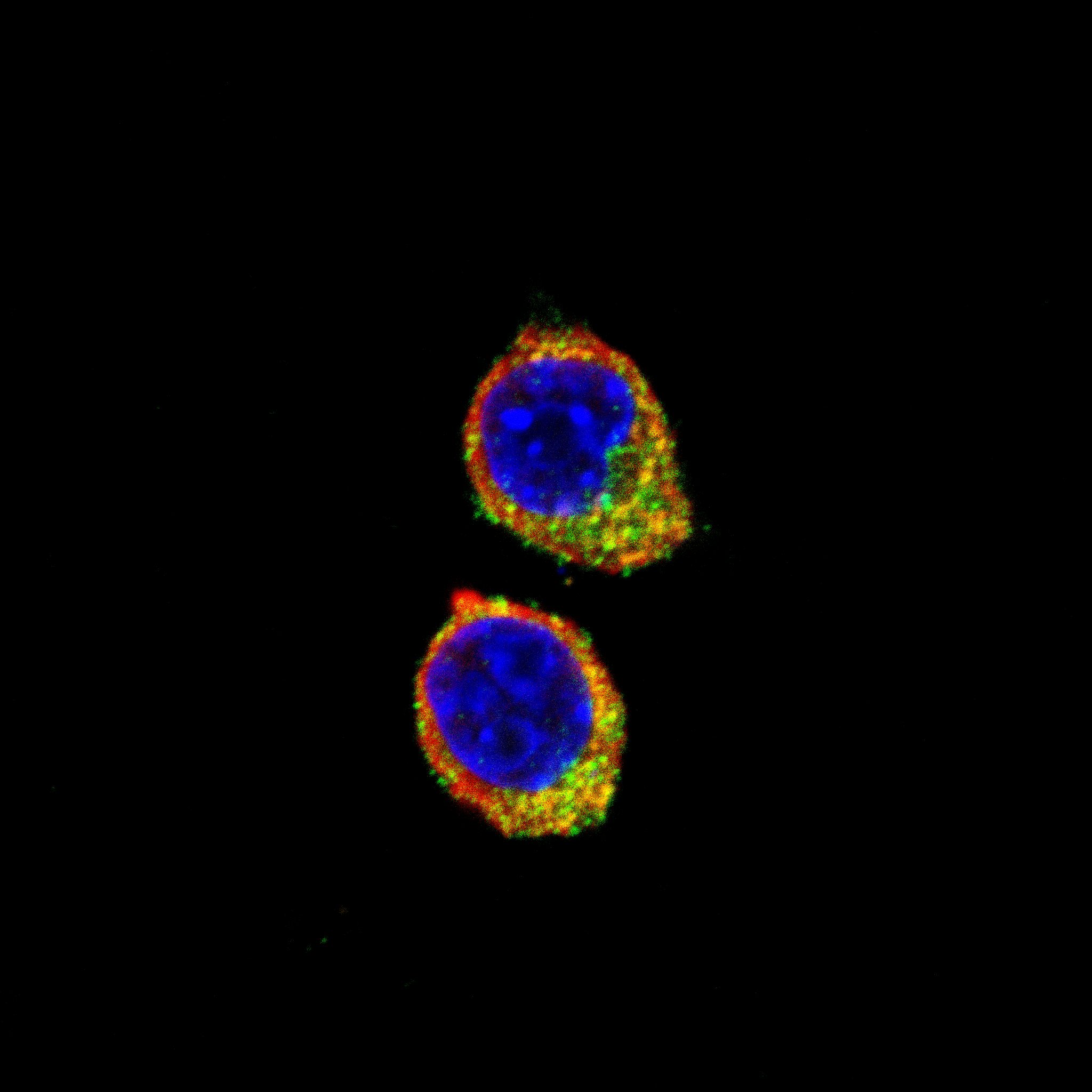

Supplement: S1 File — (ZIP) [file ppat.1012230.s002.zip › S1_File/Fig_3D/LPS/LPS-merge-4.tif]

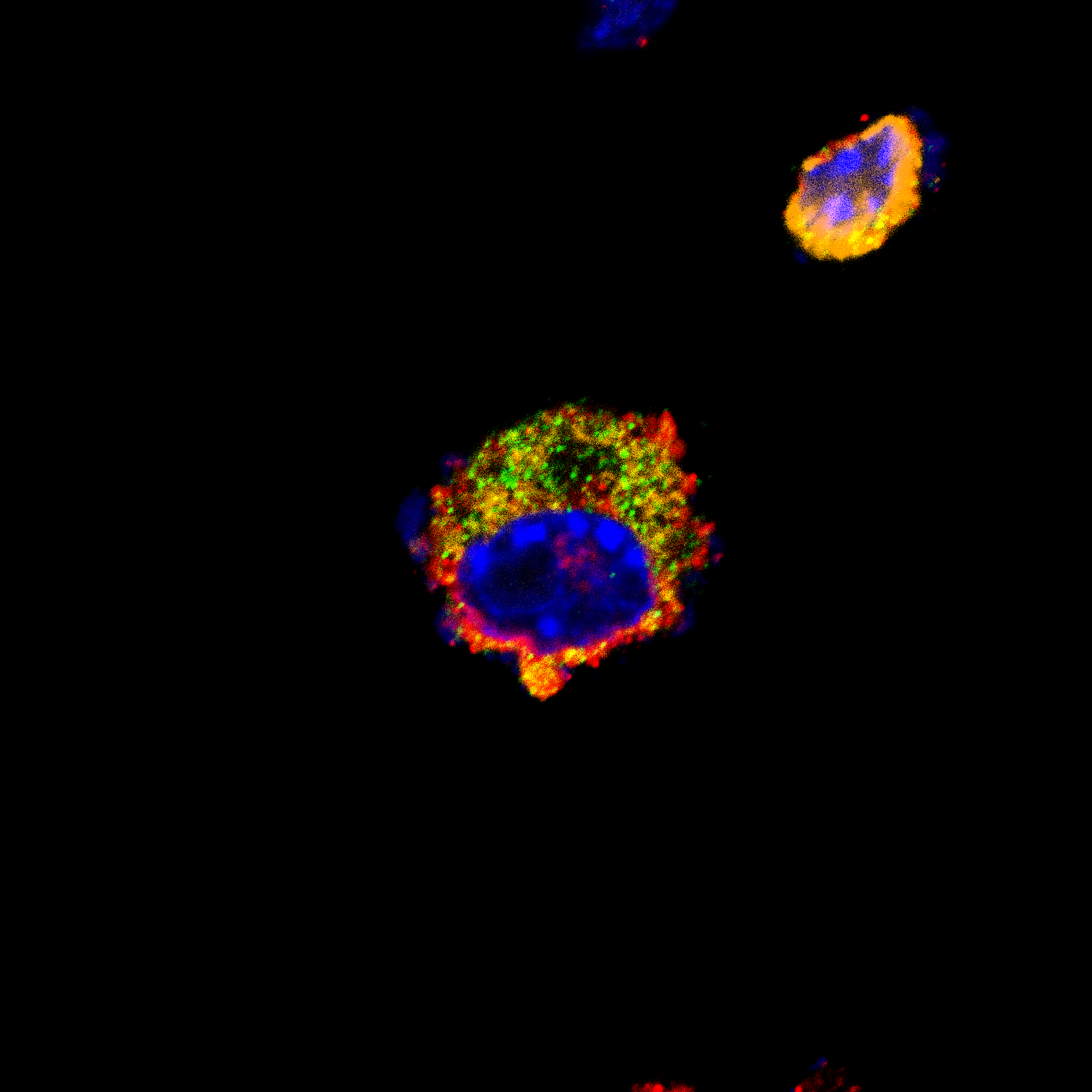

Supplement: S1 File — (ZIP) [file ppat.1012230.s002.zip › S1_File/Fig_3D/LPS/LPS-merge-5.tif]

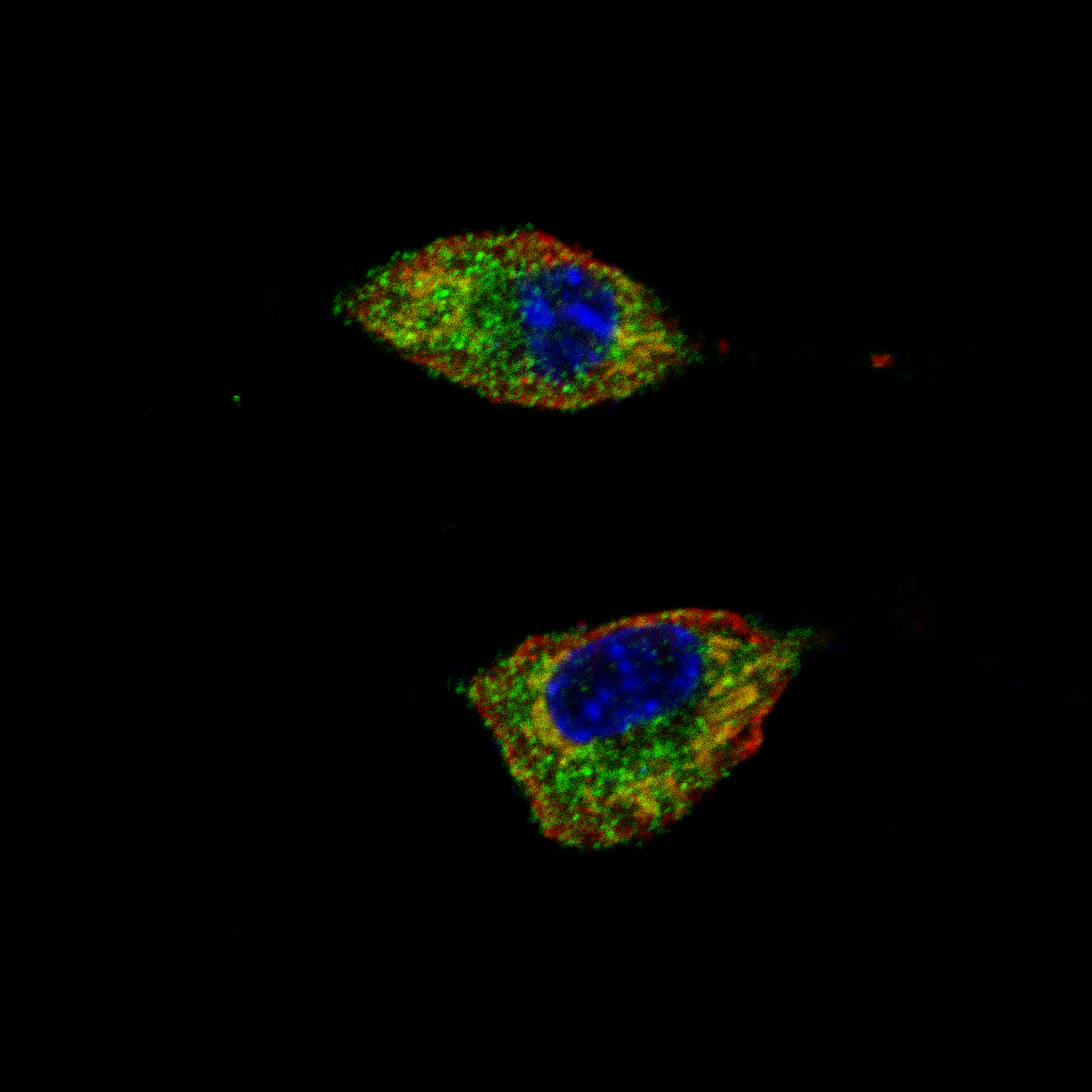

Supplement: S1 File — (ZIP) [file ppat.1012230.s002.zip › S1_File/Fig_3D/LPS/LPS-merge-6.tif]

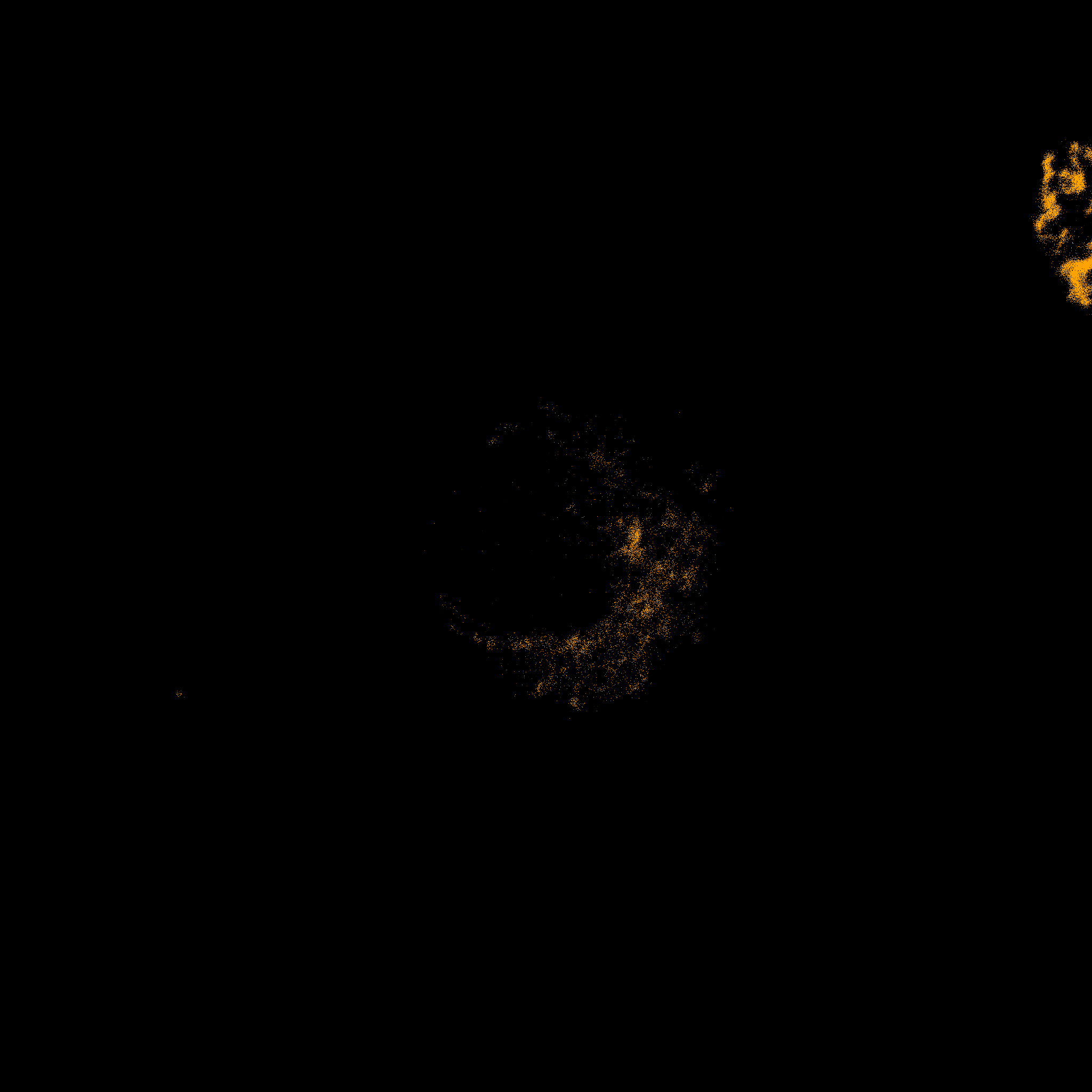

Supplement: S1 File — (ZIP) [file ppat.1012230.s002.zip › S1_File/Fig_3D/LPS/LPS-Mito-1.tif]

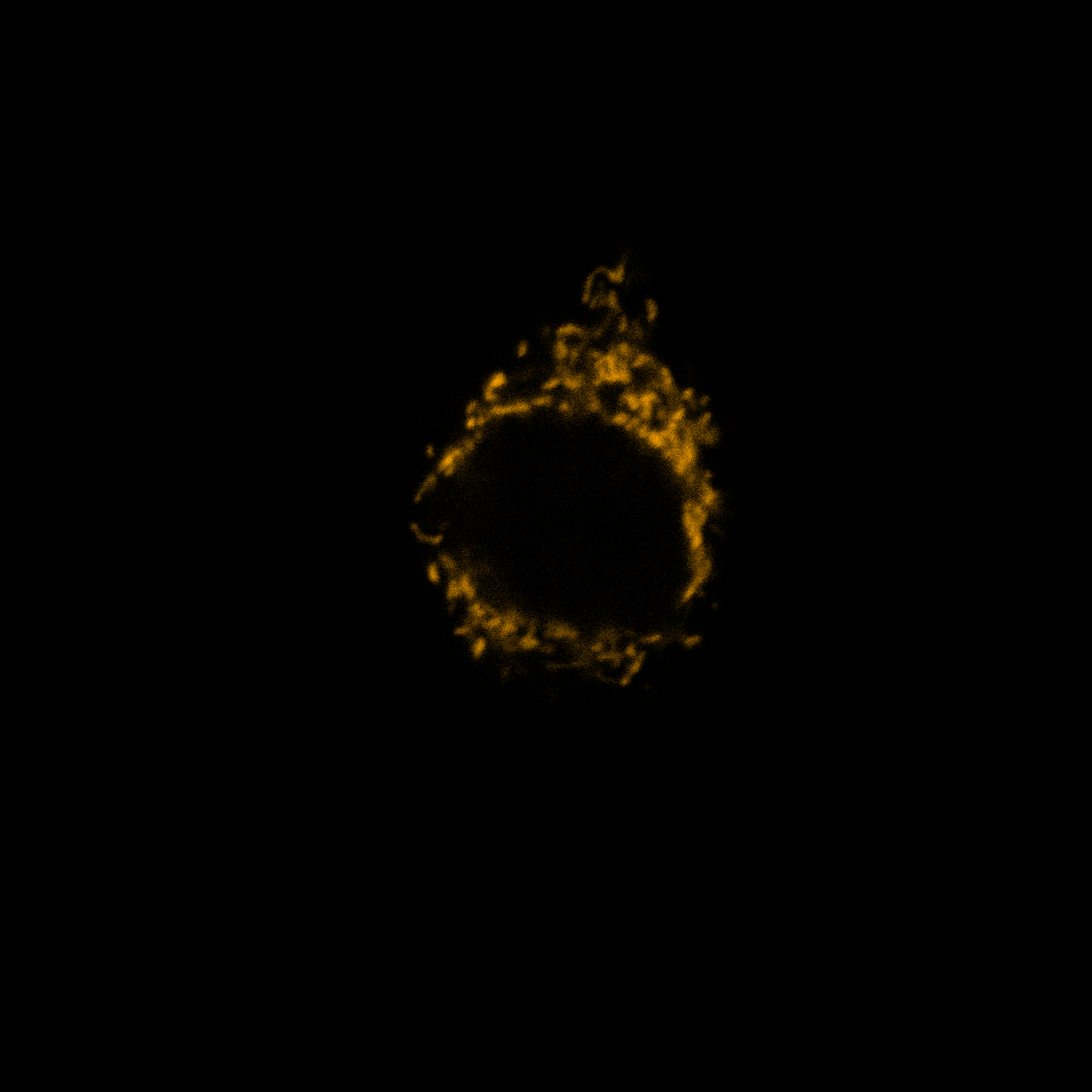

Supplement: S1 File — (ZIP) [file ppat.1012230.s002.zip › S1_File/Fig_3D/LPS/LPS-Mito-2.tif]

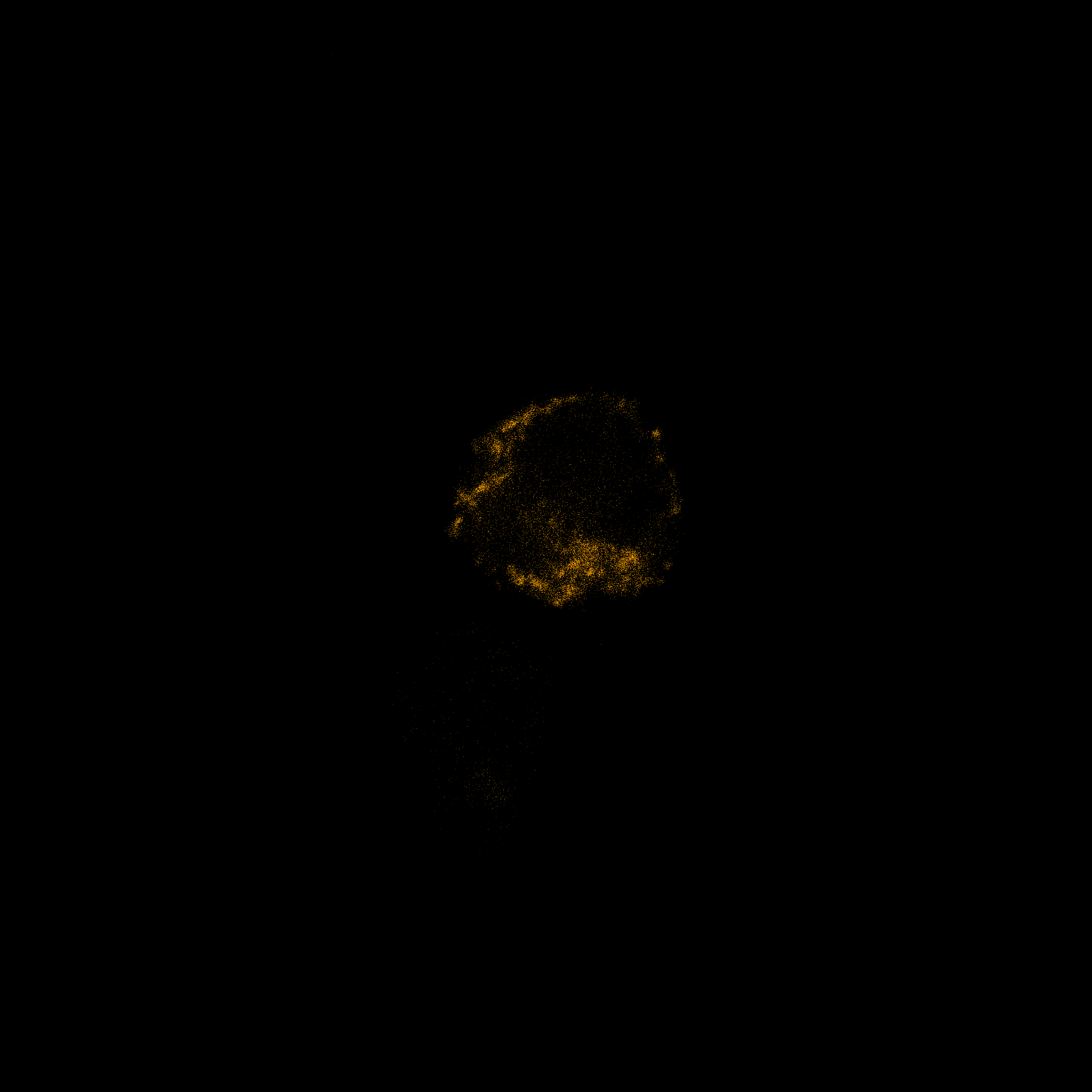

Supplement: S1 File — (ZIP) [file ppat.1012230.s002.zip › S1_File/Fig_3D/LPS/LPS-Mito-3.tif]

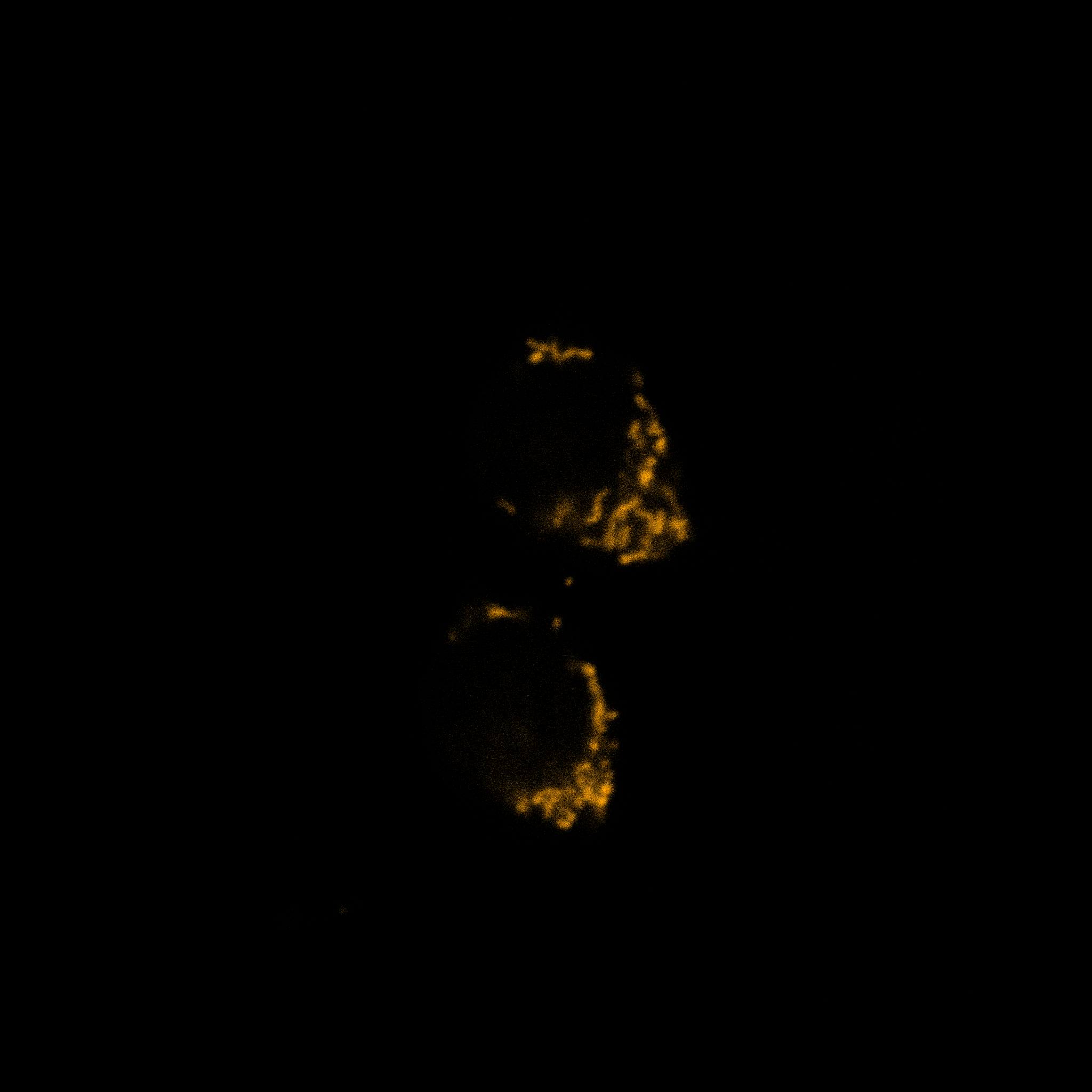

Supplement: S1 File — (ZIP) [file ppat.1012230.s002.zip › S1_File/Fig_3D/LPS/LPS-Mito-4.tif]

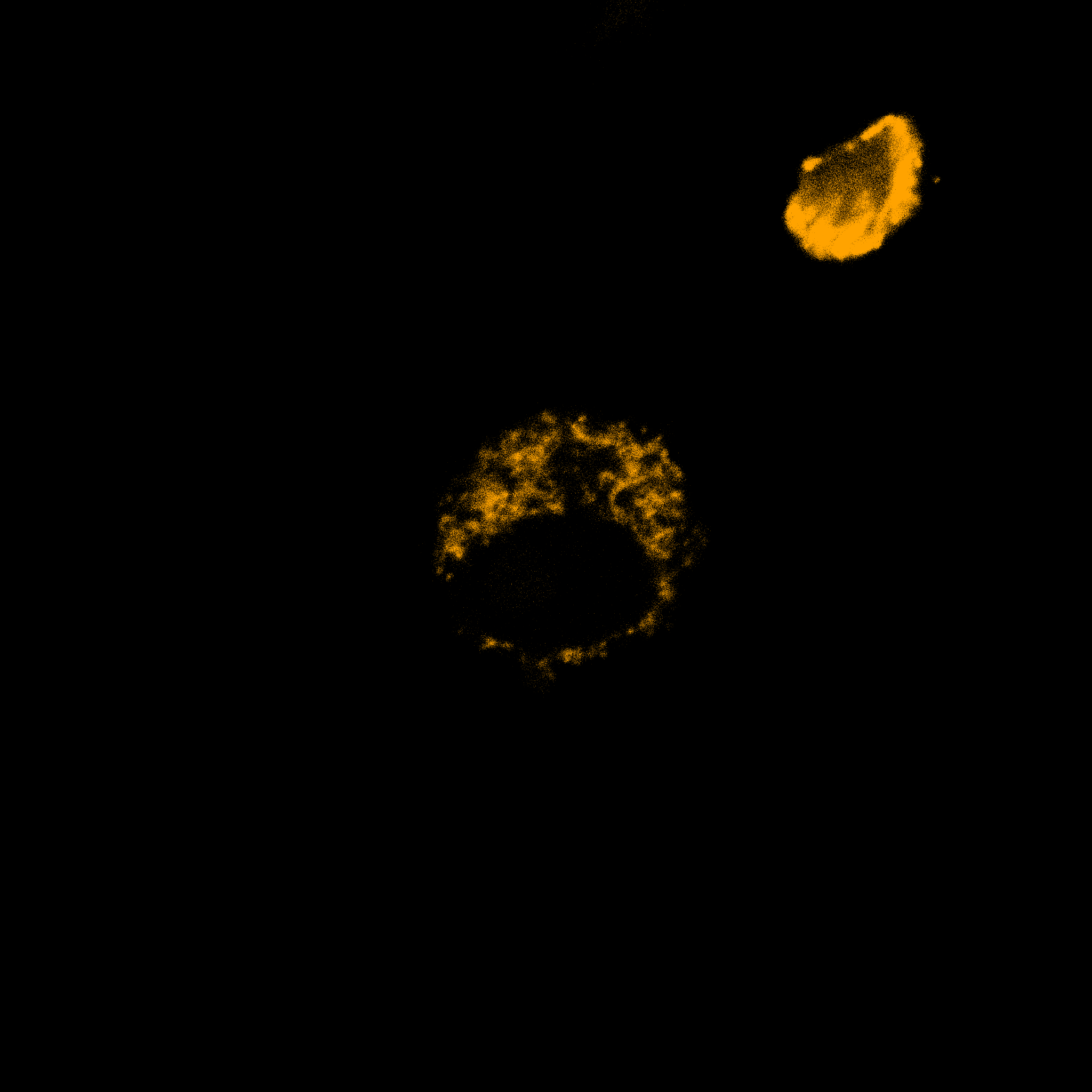

Supplement: S1 File — (ZIP) [file ppat.1012230.s002.zip › S1_File/Fig_3D/LPS/LPS-Mito-5.tif]

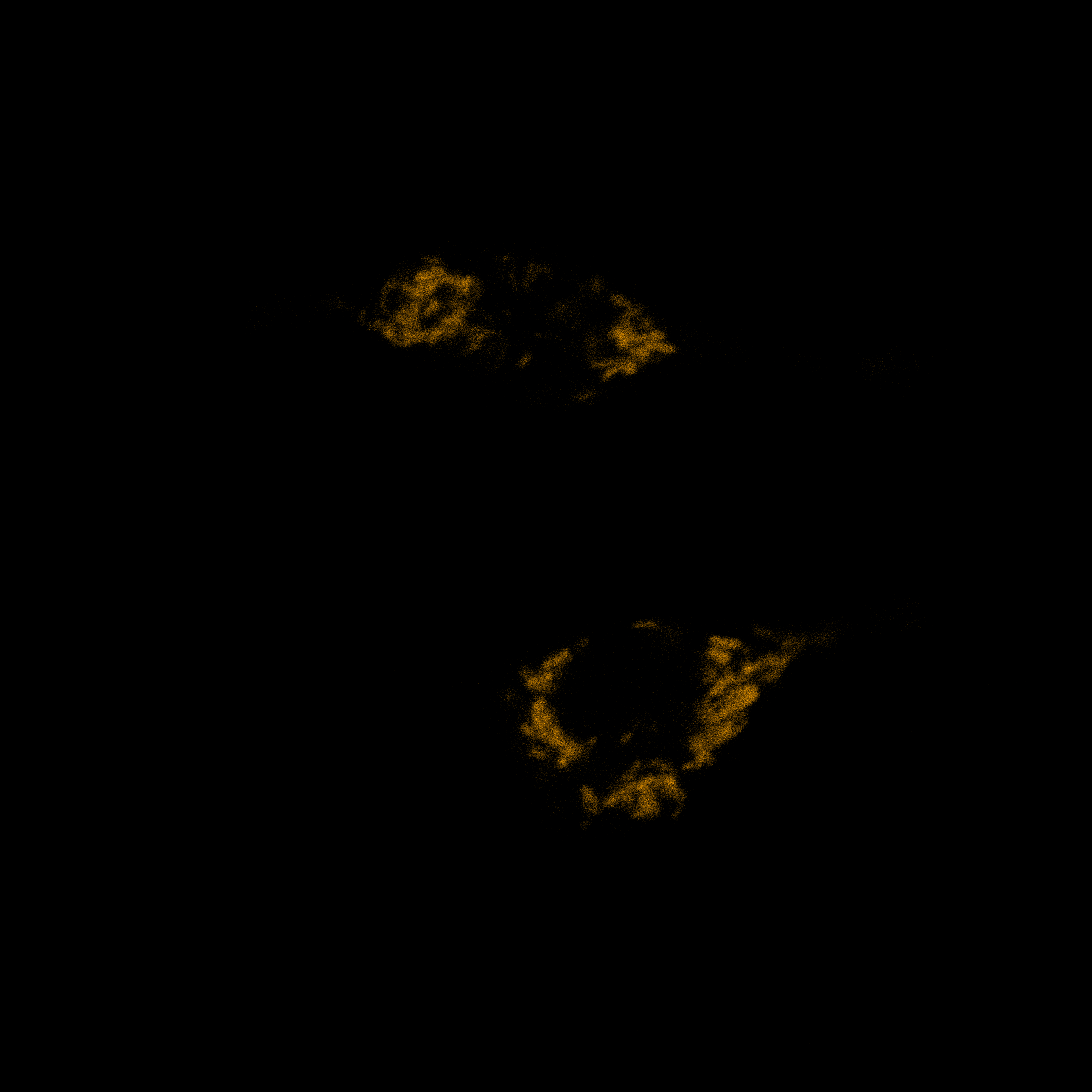

Supplement: S1 File — (ZIP) [file ppat.1012230.s002.zip › S1_File/Fig_3D/LPS/LPS-Mito-6.tif]

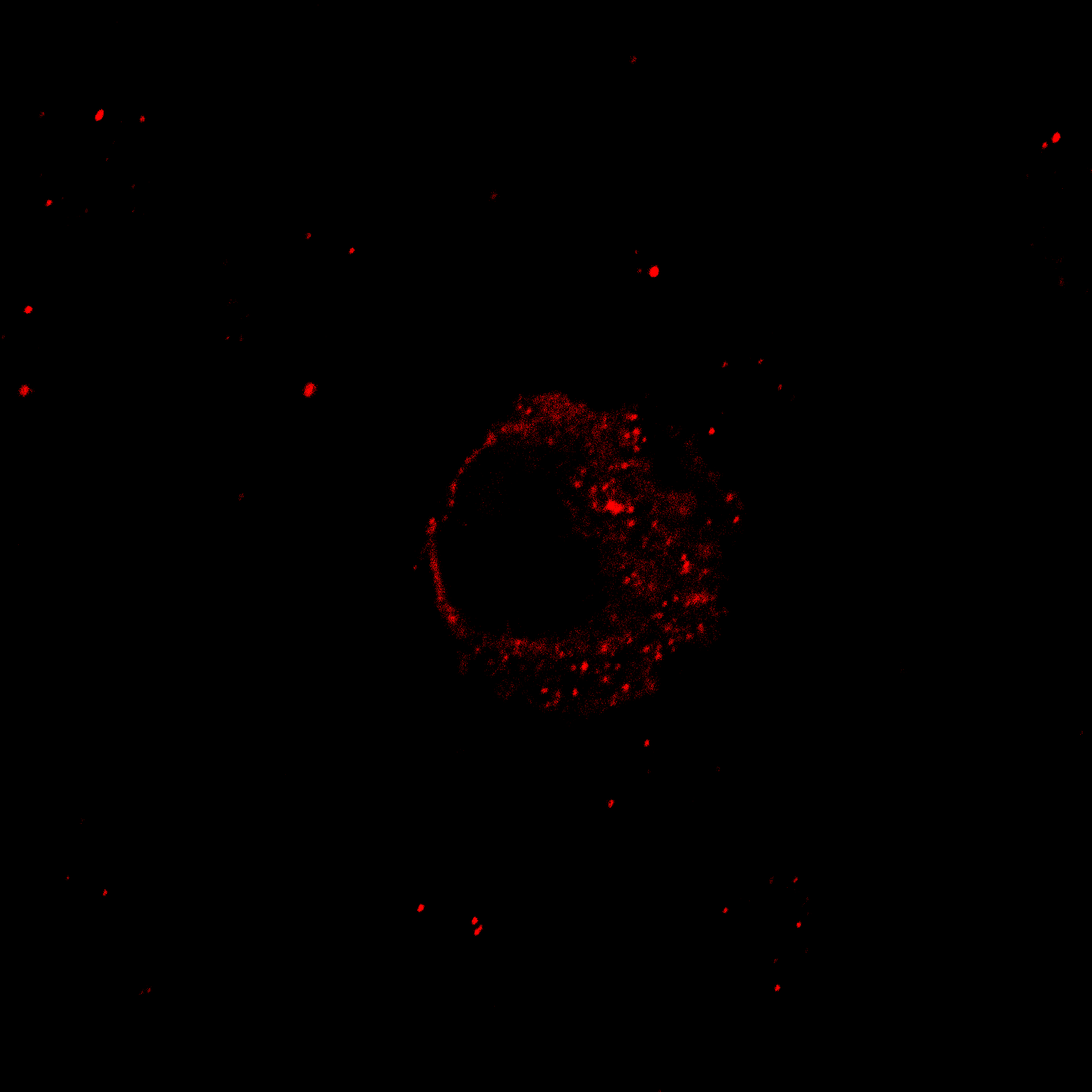

Supplement: S1 File — (ZIP) [file ppat.1012230.s002.zip › S1_File/Fig_3D/LPS/LPS-RIG-I-1.tif]

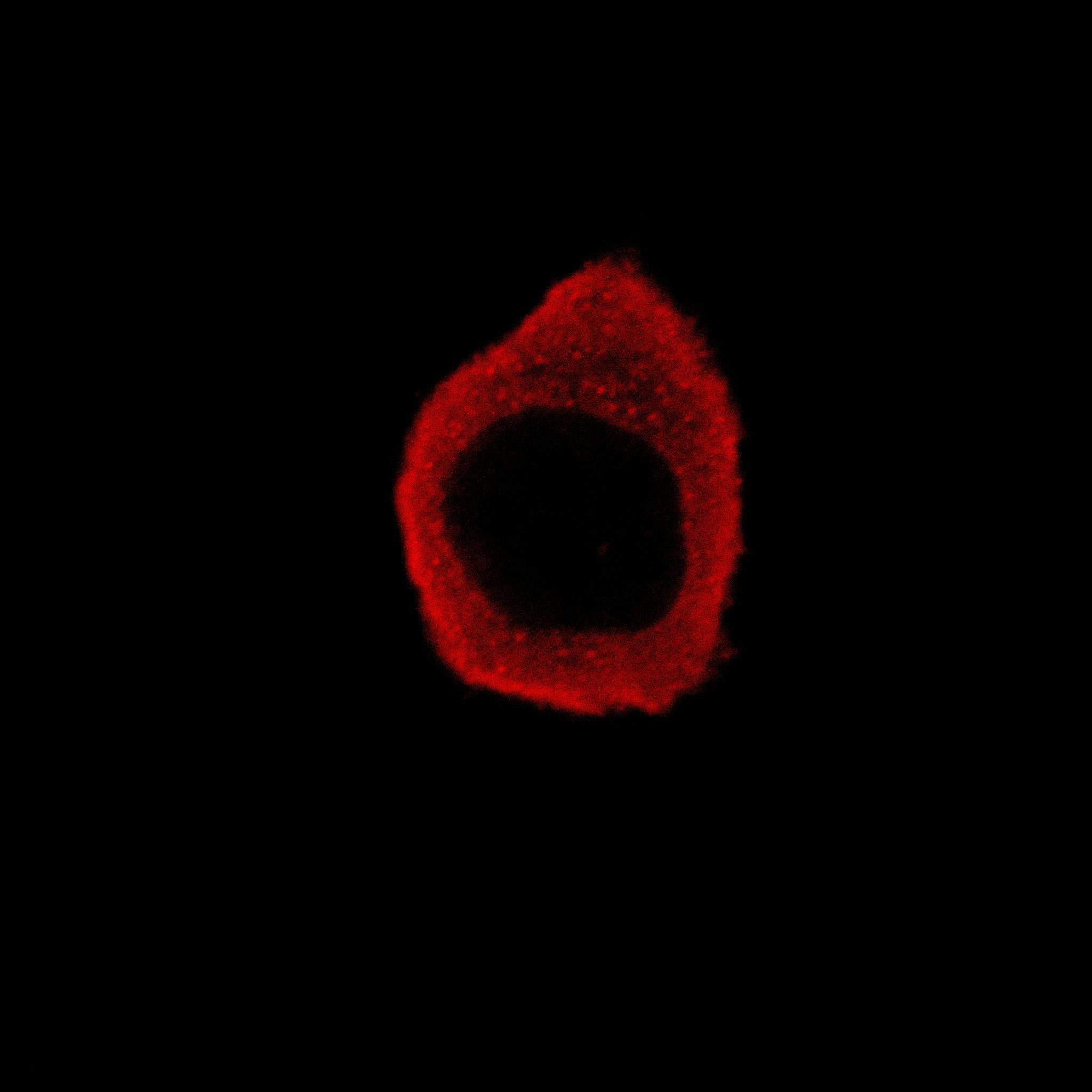

Supplement: S1 File — (ZIP) [file ppat.1012230.s002.zip › S1_File/Fig_3D/LPS/LPS-RIG-I-2.tif]

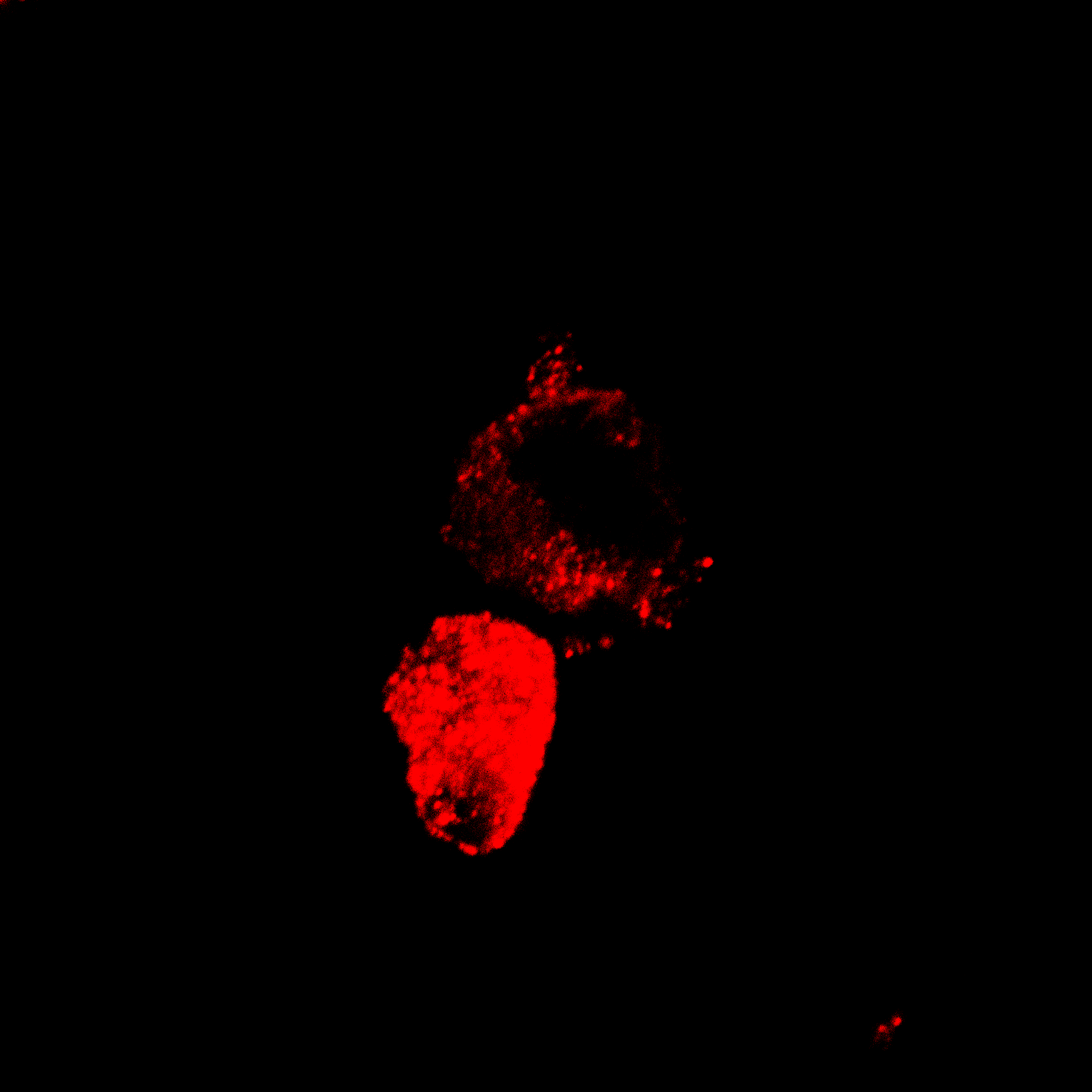

Supplement: S1 File — (ZIP) [file ppat.1012230.s002.zip › S1_File/Fig_3D/LPS/LPS-RIG-I-3.tif]

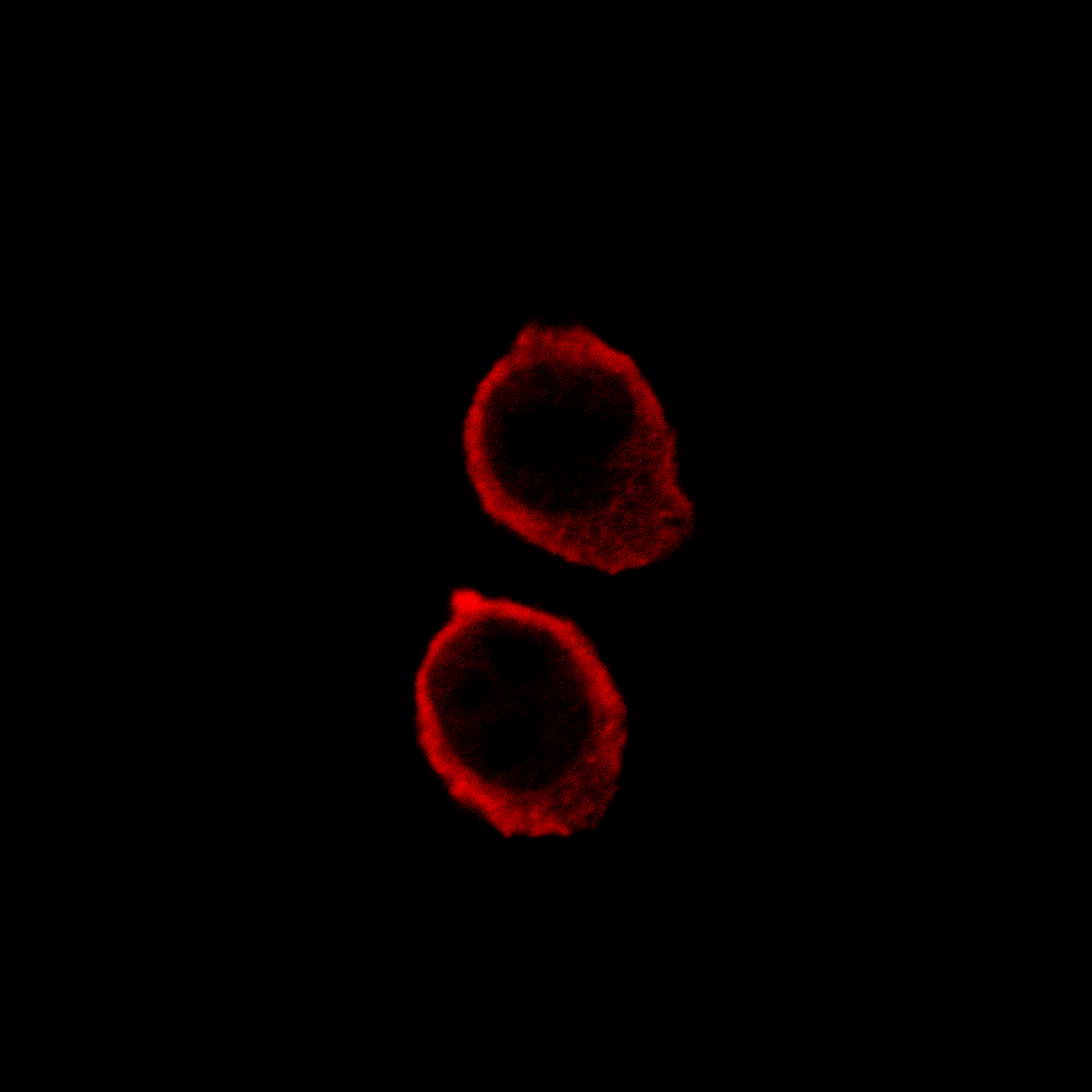

Supplement: S1 File — (ZIP) [file ppat.1012230.s002.zip › S1_File/Fig_3D/LPS/LPS-RIG-I-4.tif]

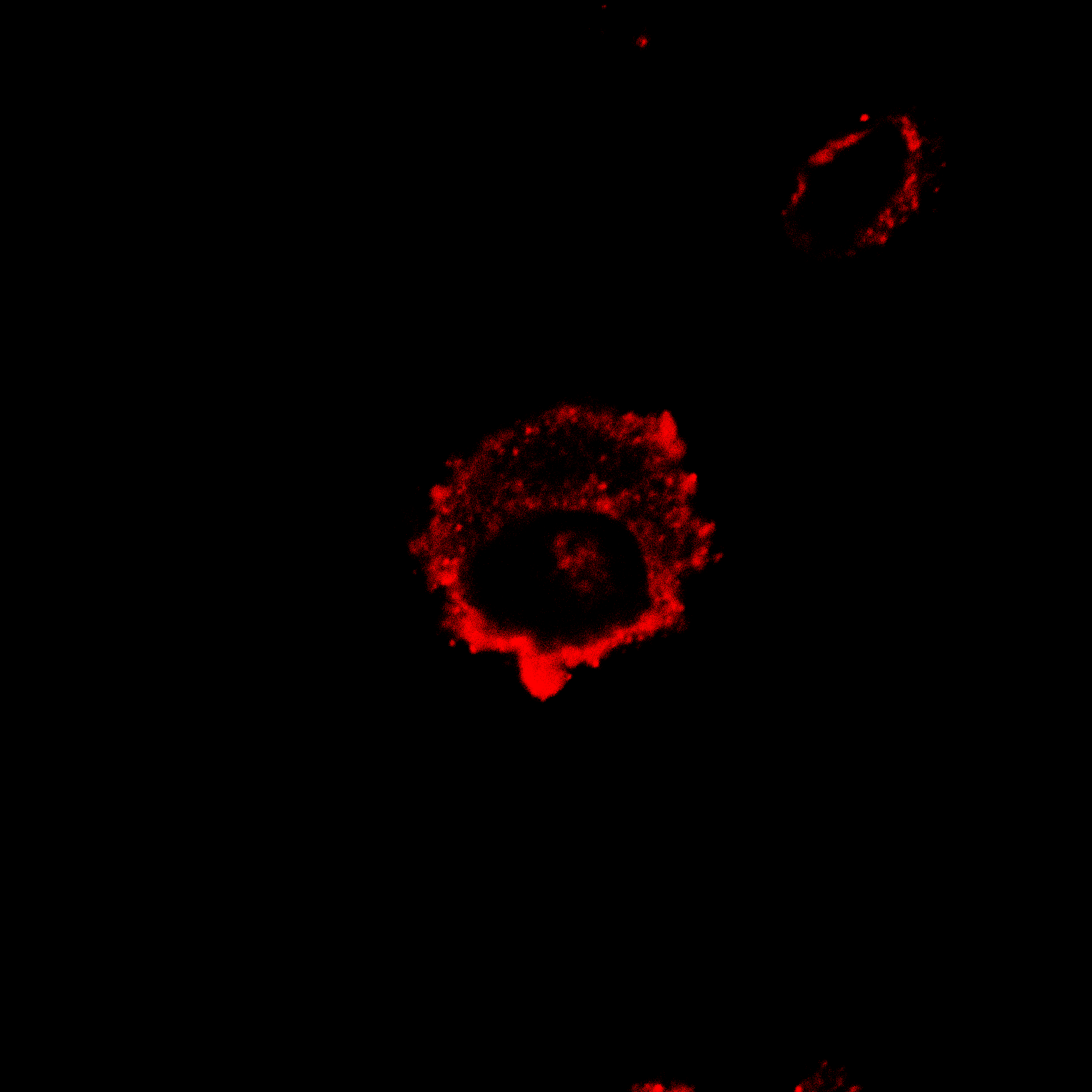

Supplement: S1 File — (ZIP) [file ppat.1012230.s002.zip › S1_File/Fig_3D/LPS/LPS-RIG-I-5.tif]

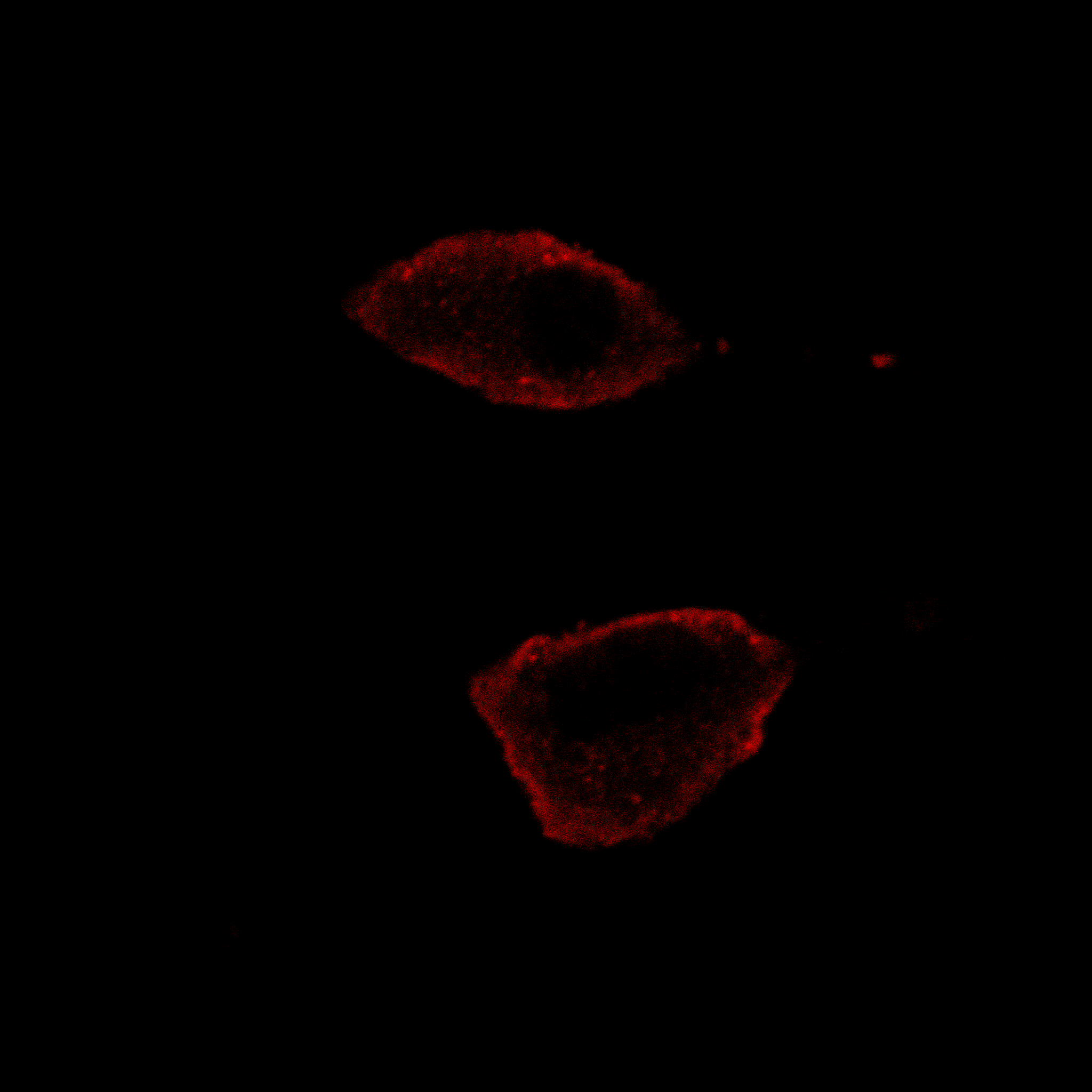

Supplement: S1 File — (ZIP) [file ppat.1012230.s002.zip › S1_File/Fig_3D/LPS/LPS-RIG-I-6.tif]

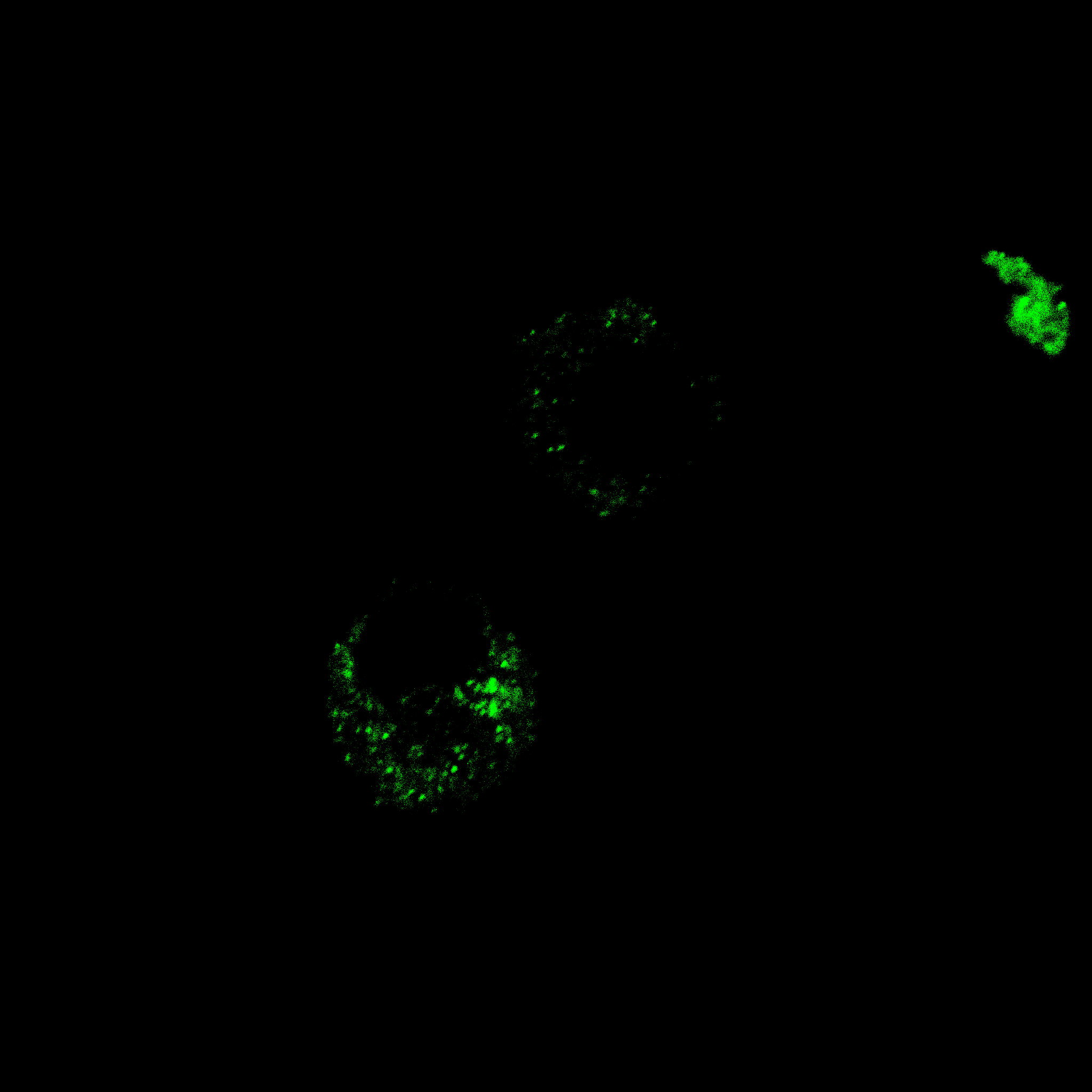

Supplement: S1 File — (ZIP) [file ppat.1012230.s002.zip › S1_File/Fig_3D/Resting/Resting-ADAP-1.tif]

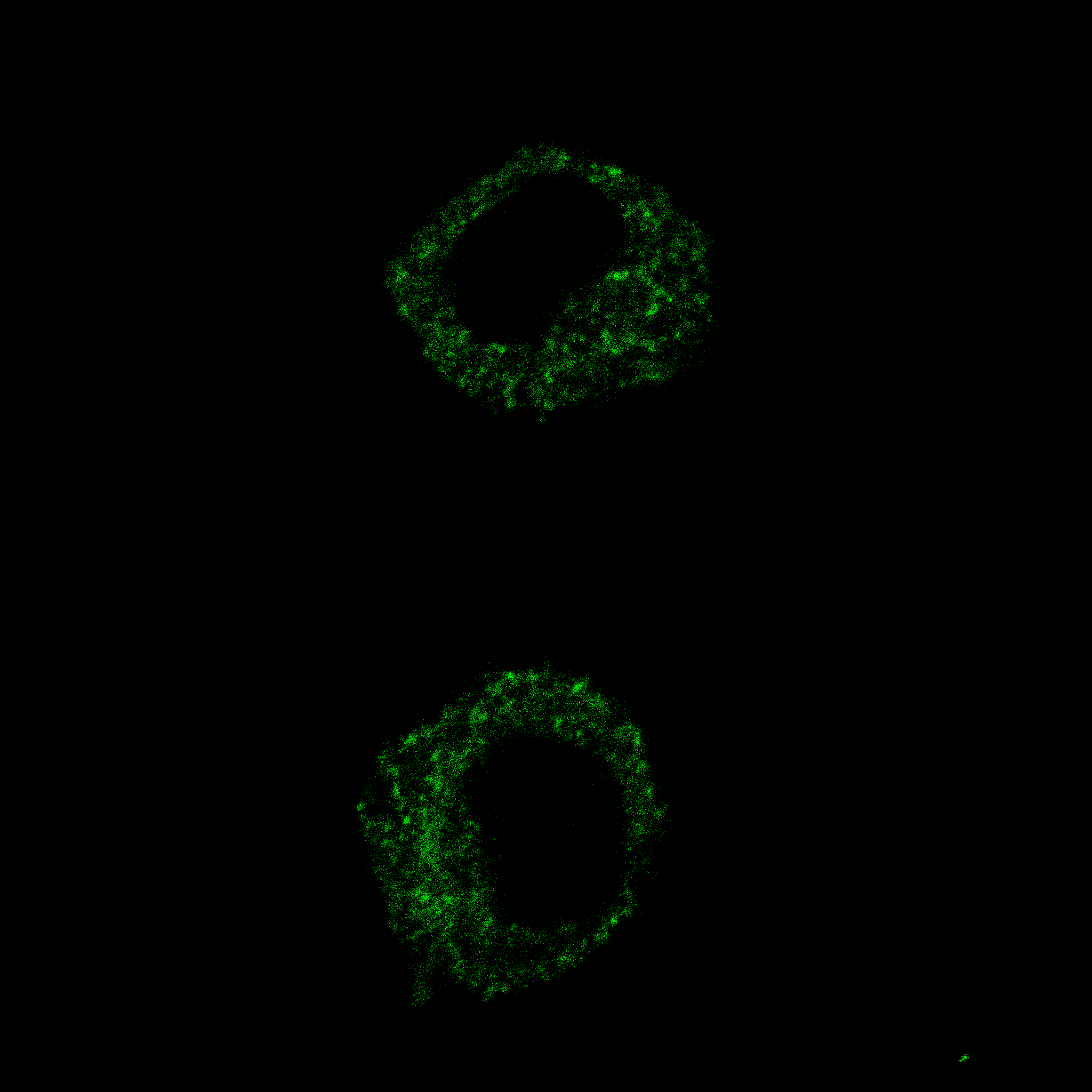

Supplement: S1 File — (ZIP) [file ppat.1012230.s002.zip › S1_File/Fig_3D/Resting/Resting-ADAP-2.tif]

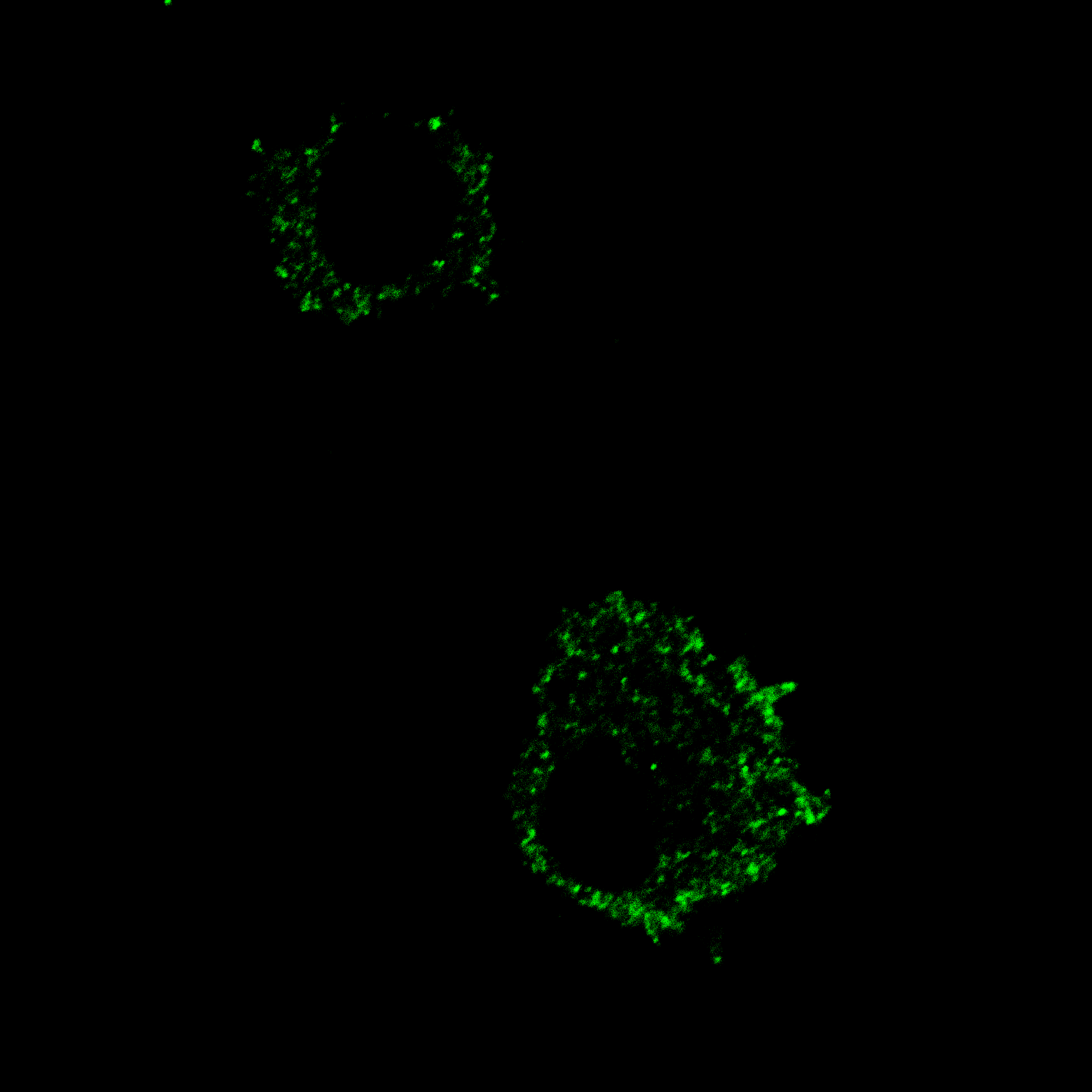

Supplement: S1 File — (ZIP) [file ppat.1012230.s002.zip › S1_File/Fig_3D/Resting/Resting-ADAP-3.tif]

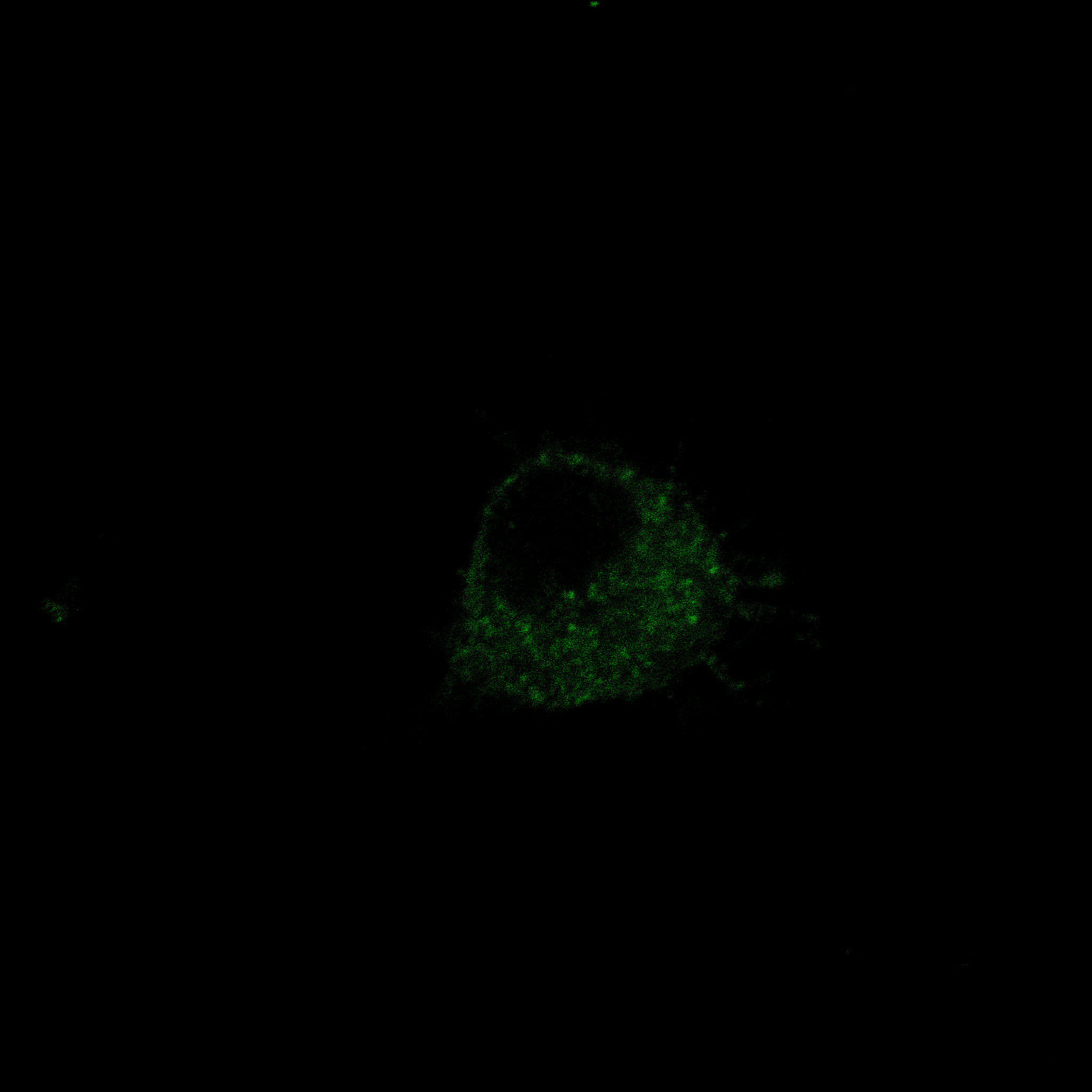

Supplement: S1 File — (ZIP) [file ppat.1012230.s002.zip › S1_File/Fig_3D/Resting/Resting-ADAP-4.tif]

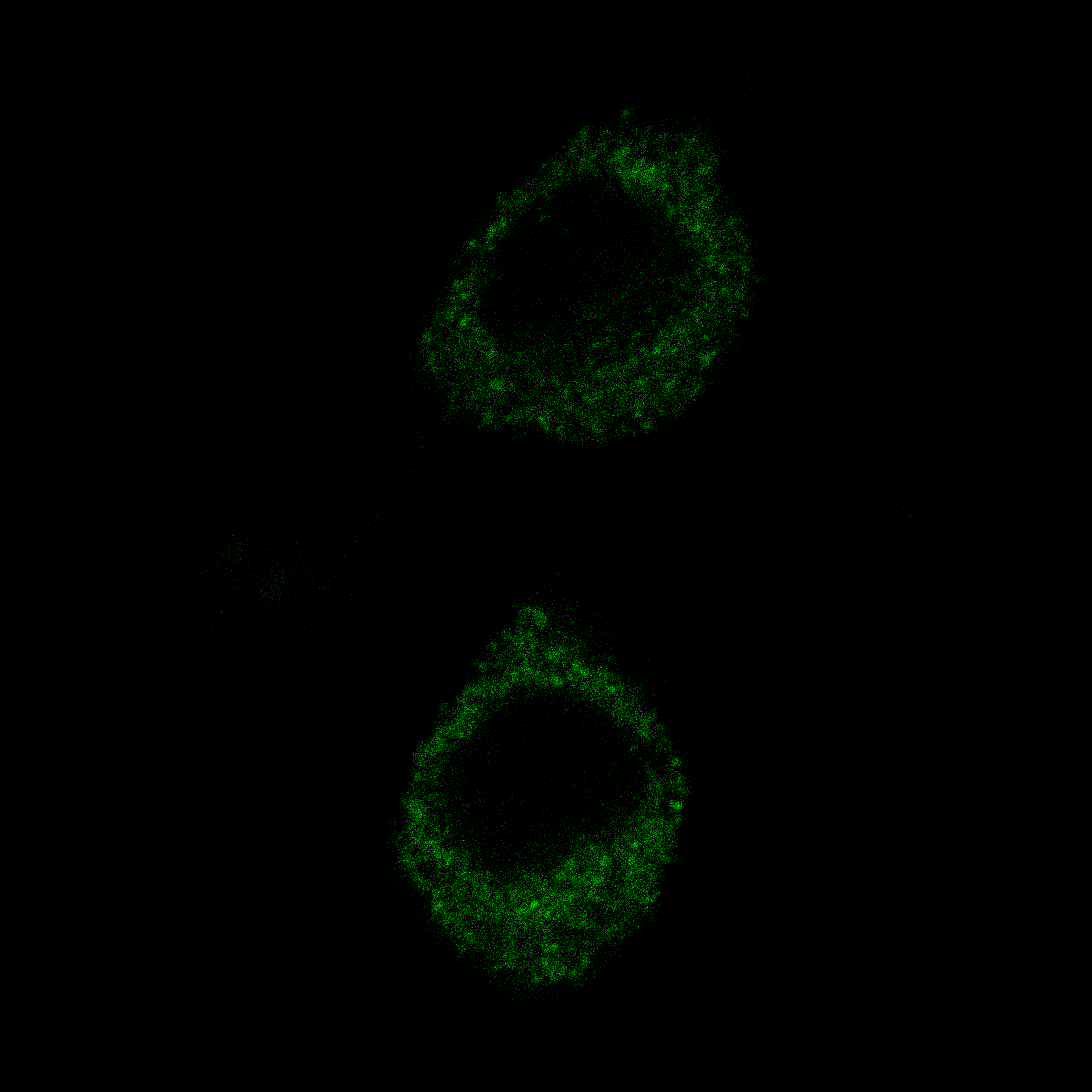

Supplement: S1 File — (ZIP) [file ppat.1012230.s002.zip › S1_File/Fig_3D/Resting/Resting-ADAP-5.tif]

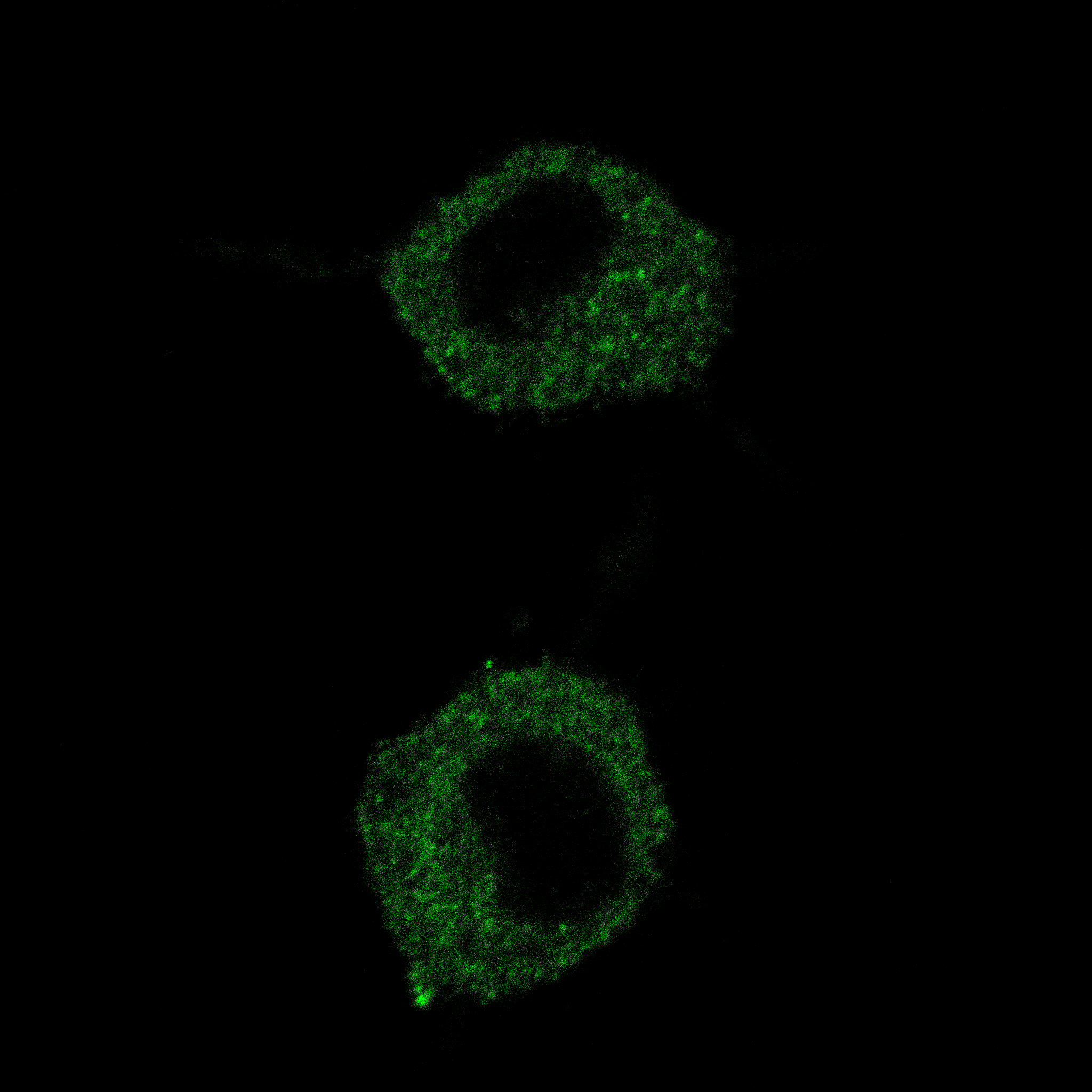

Supplement: S1 File — (ZIP) [file ppat.1012230.s002.zip › S1_File/Fig_3D/Resting/Resting-ADAP-6.tif]

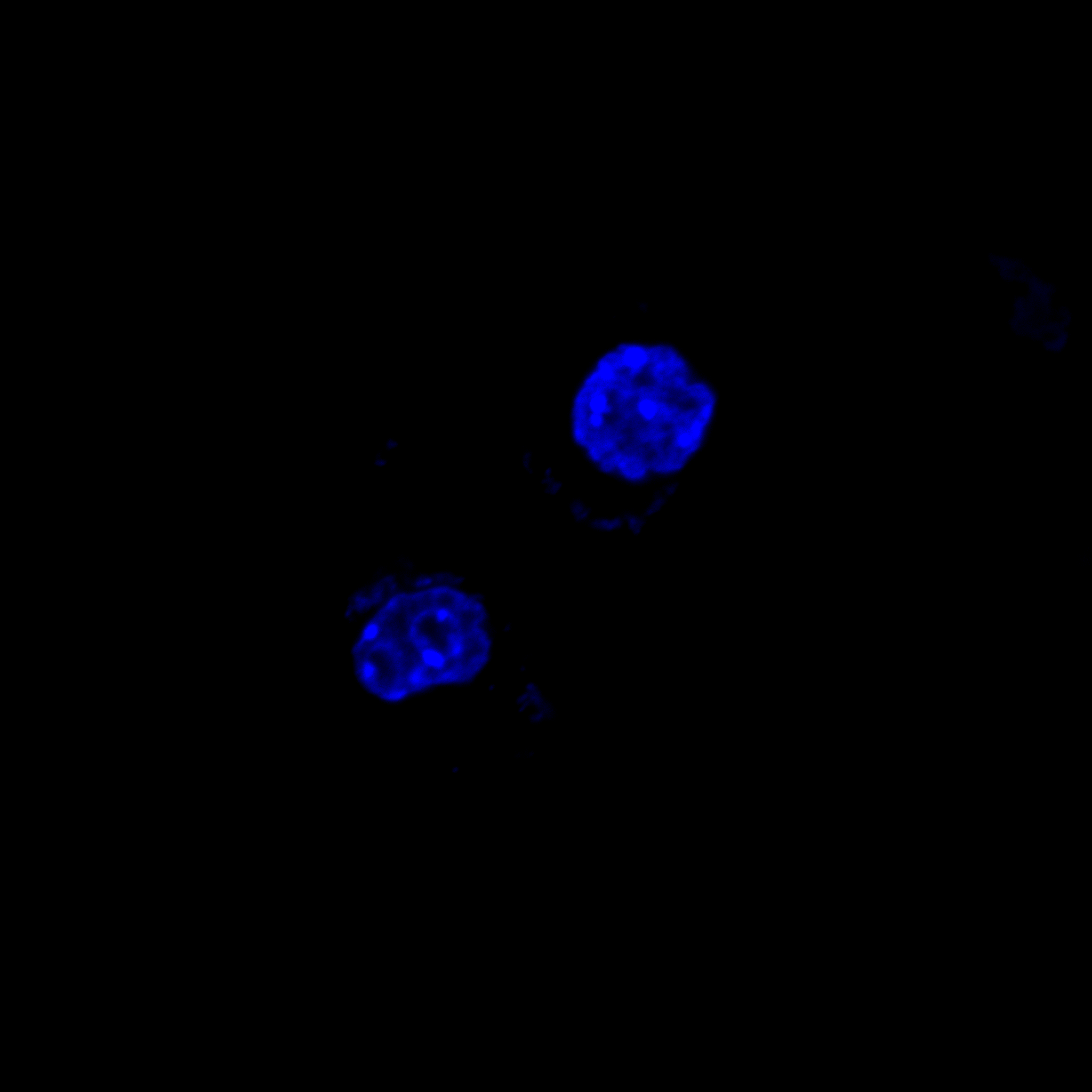

Supplement: S1 File — (ZIP) [file ppat.1012230.s002.zip › S1_File/Fig_3D/Resting/Resting-DAPI-1.tif]

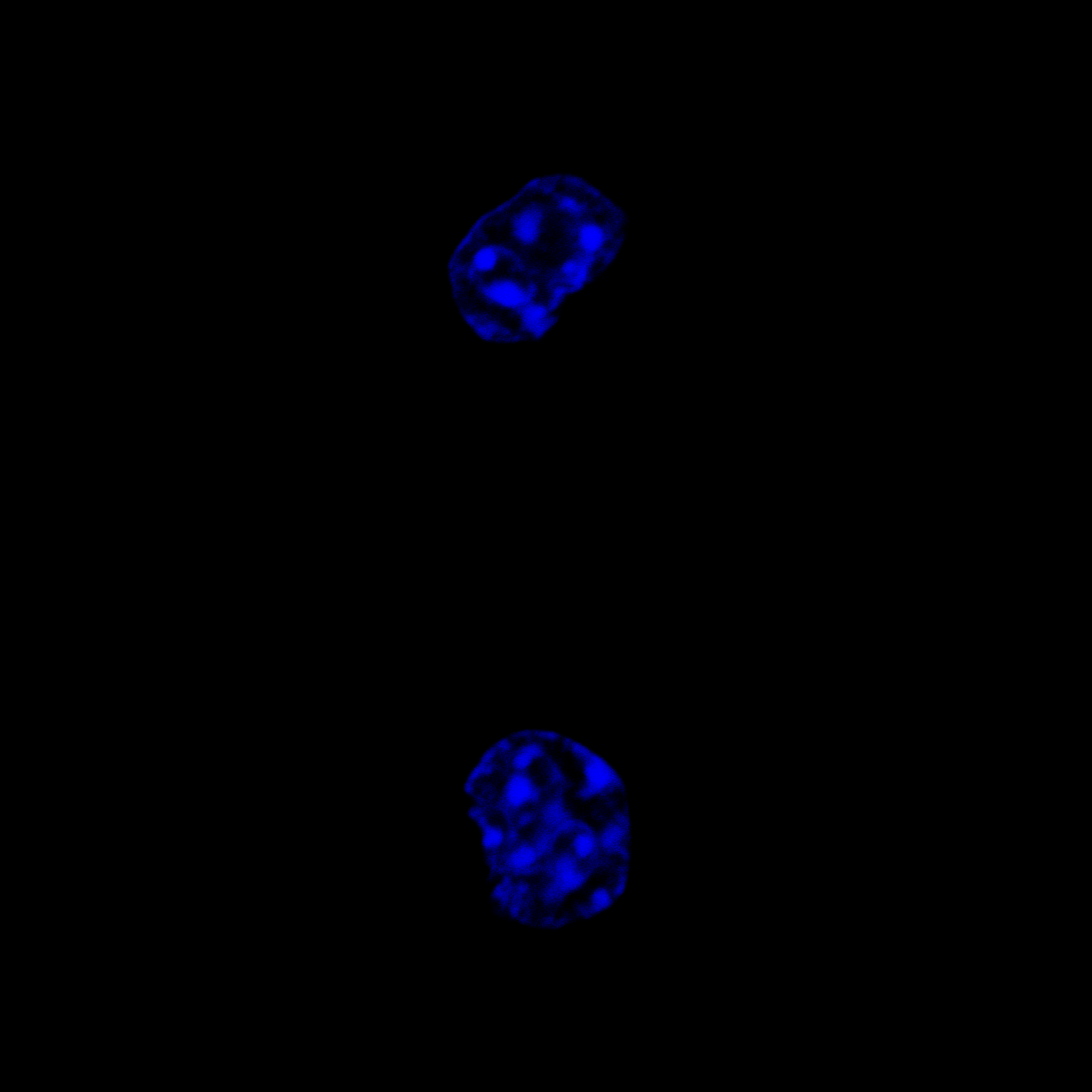

Supplement: S1 File — (ZIP) [file ppat.1012230.s002.zip › S1_File/Fig_3D/Resting/Resting-DAPI-2.tif]

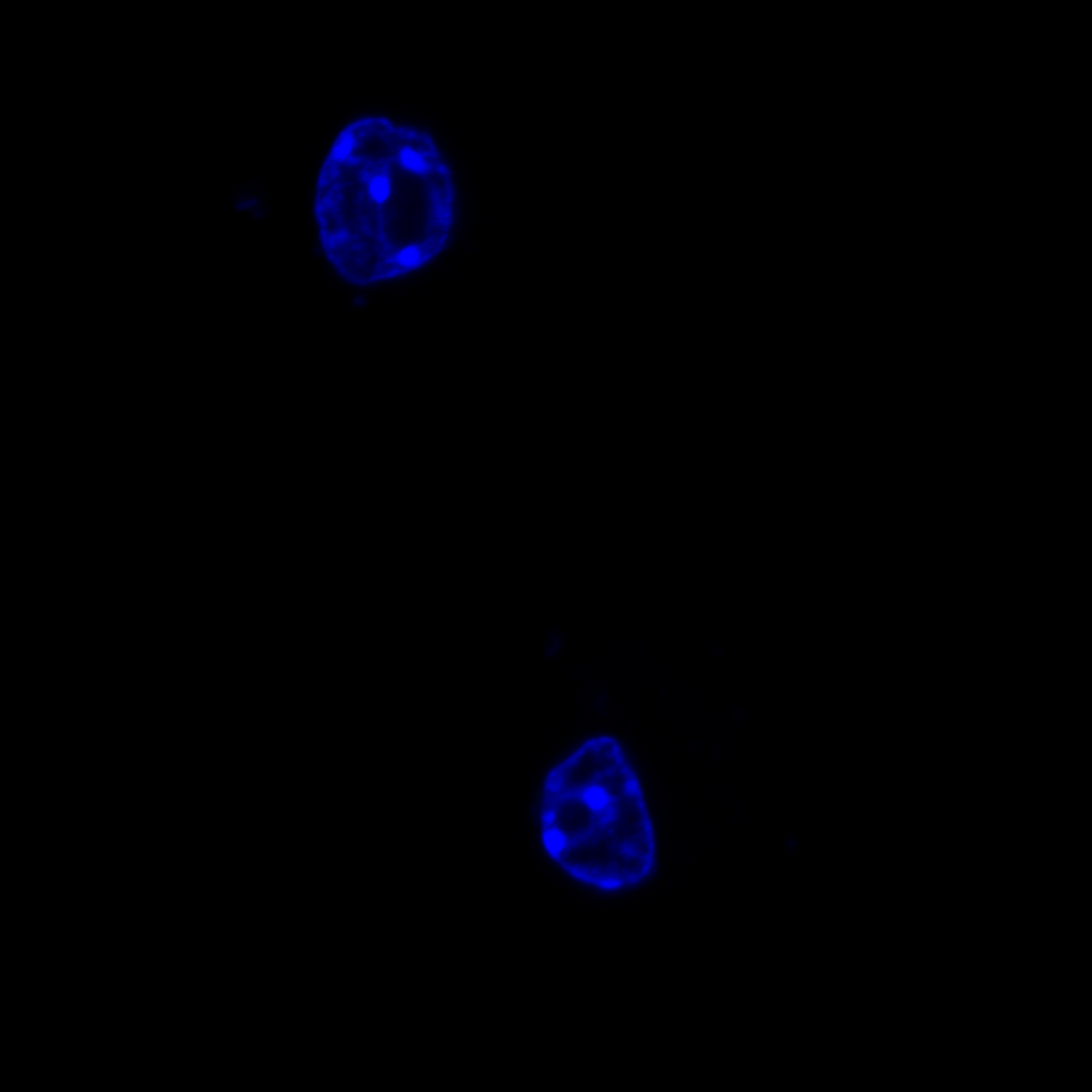

Supplement: S1 File — (ZIP) [file ppat.1012230.s002.zip › S1_File/Fig_3D/Resting/Resting-DAPI-3.tif]

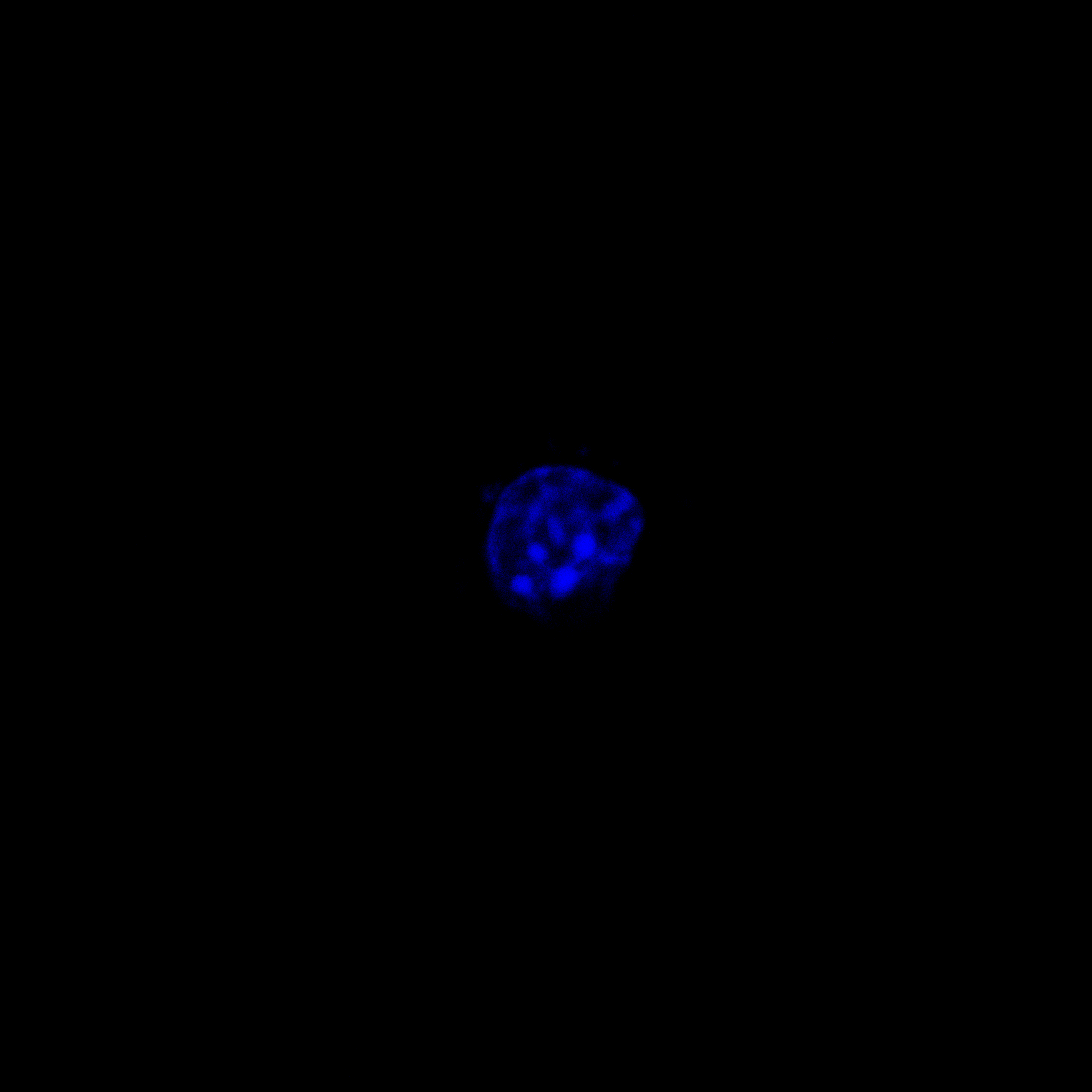

Supplement: S1 File — (ZIP) [file ppat.1012230.s002.zip › S1_File/Fig_3D/Resting/Resting-DAPI-4.tif]

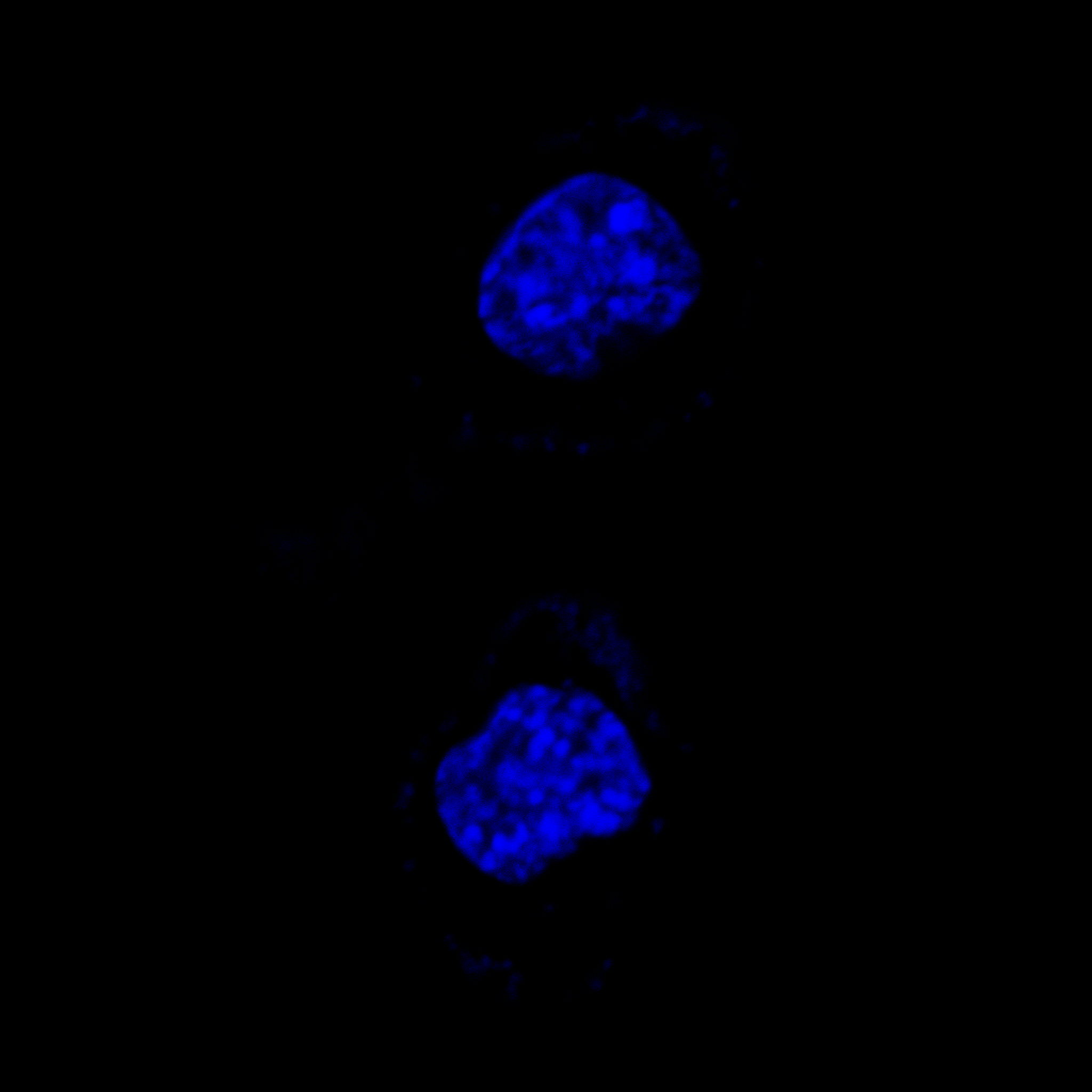

Supplement: S1 File — (ZIP) [file ppat.1012230.s002.zip › S1_File/Fig_3D/Resting/Resting-DAPI-5.tif]

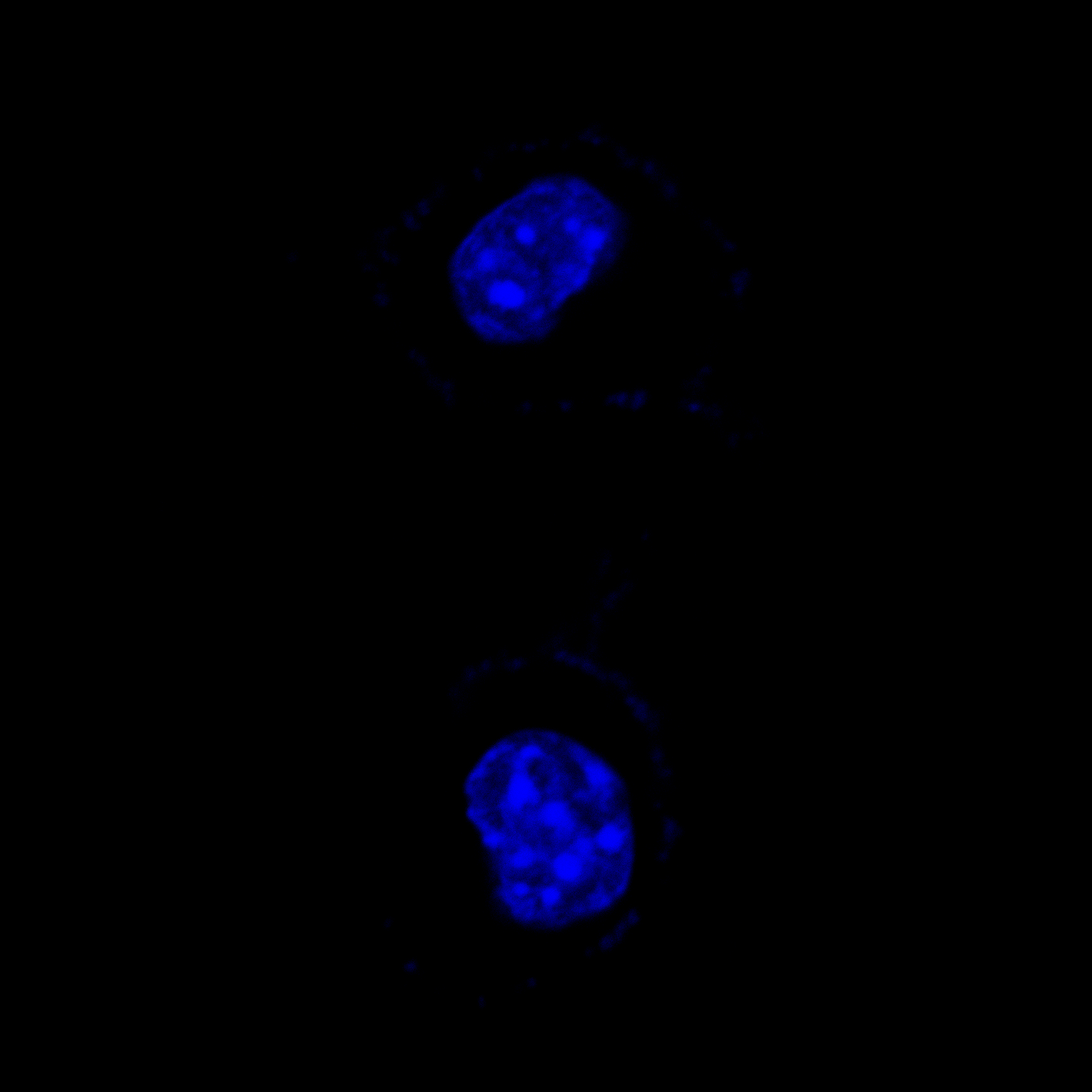

Supplement: S1 File — (ZIP) [file ppat.1012230.s002.zip › S1_File/Fig_3D/Resting/Resting-DAPI-6.tif]

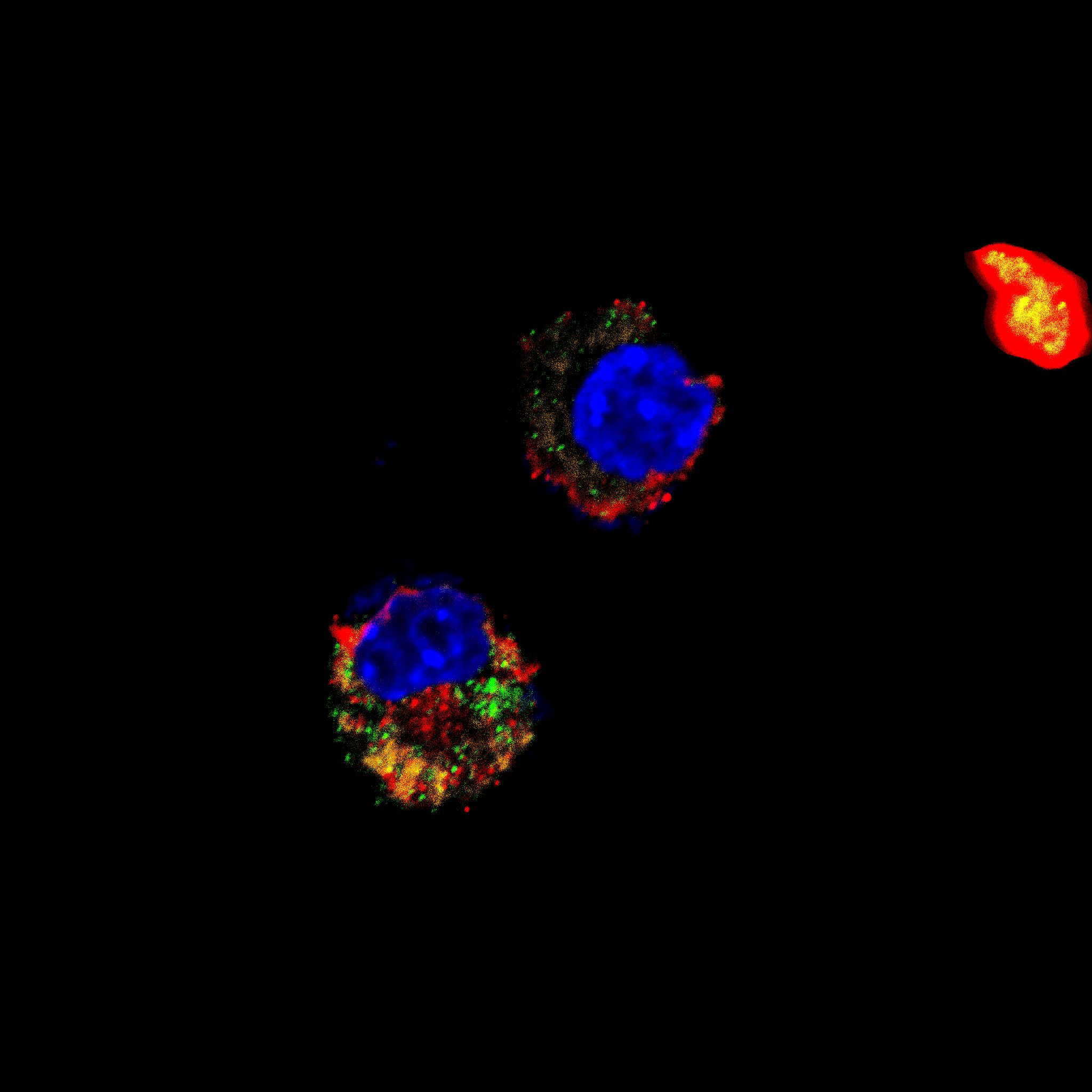

Supplement: S1 File — (ZIP) [file ppat.1012230.s002.zip › S1_File/Fig_3D/Resting/Resting-merge-1.tif]

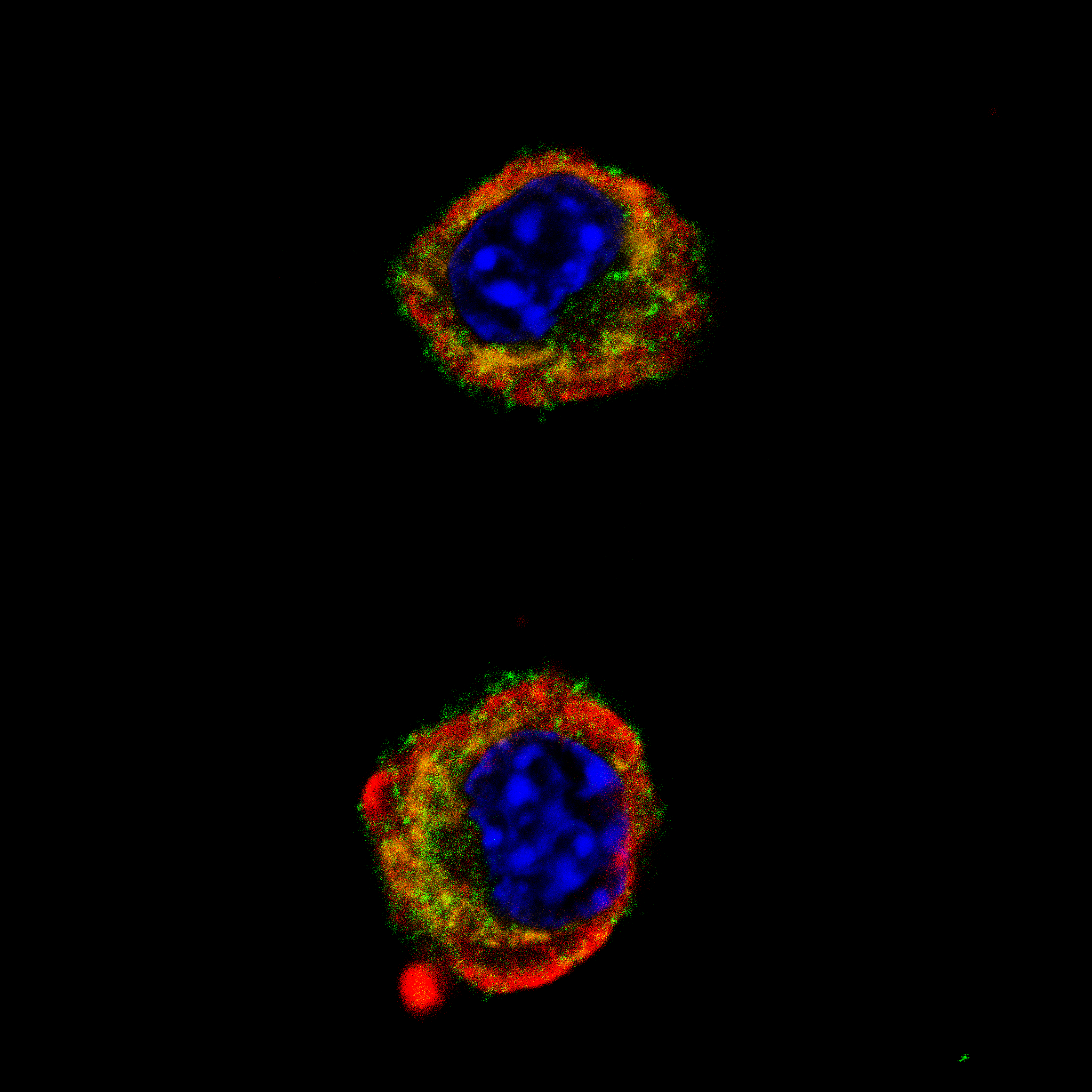

Supplement: S1 File — (ZIP) [file ppat.1012230.s002.zip › S1_File/Fig_3D/Resting/Resting-merge-2.tif]

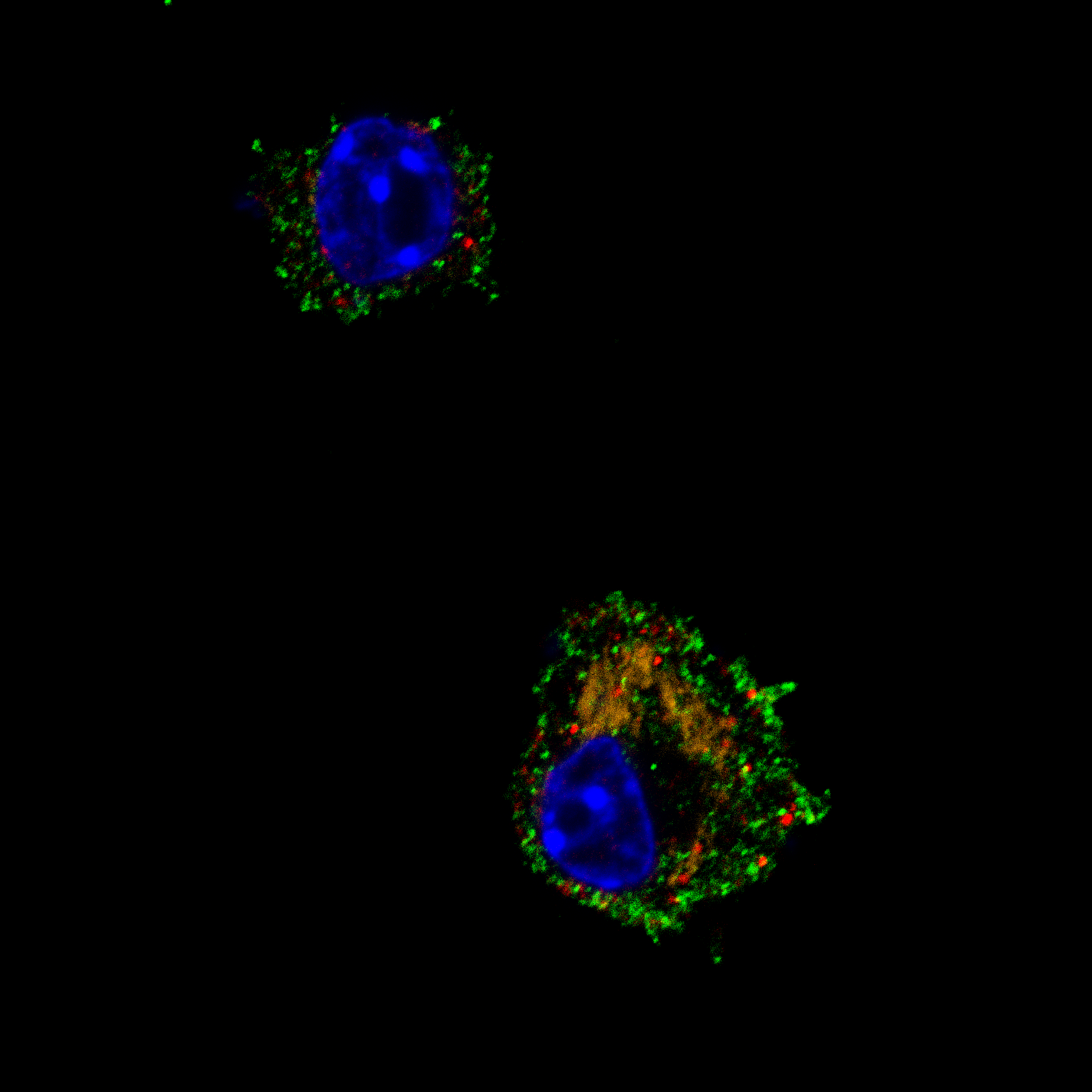

Supplement: S1 File — (ZIP) [file ppat.1012230.s002.zip › S1_File/Fig_3D/Resting/Resting-merge-3.tif]

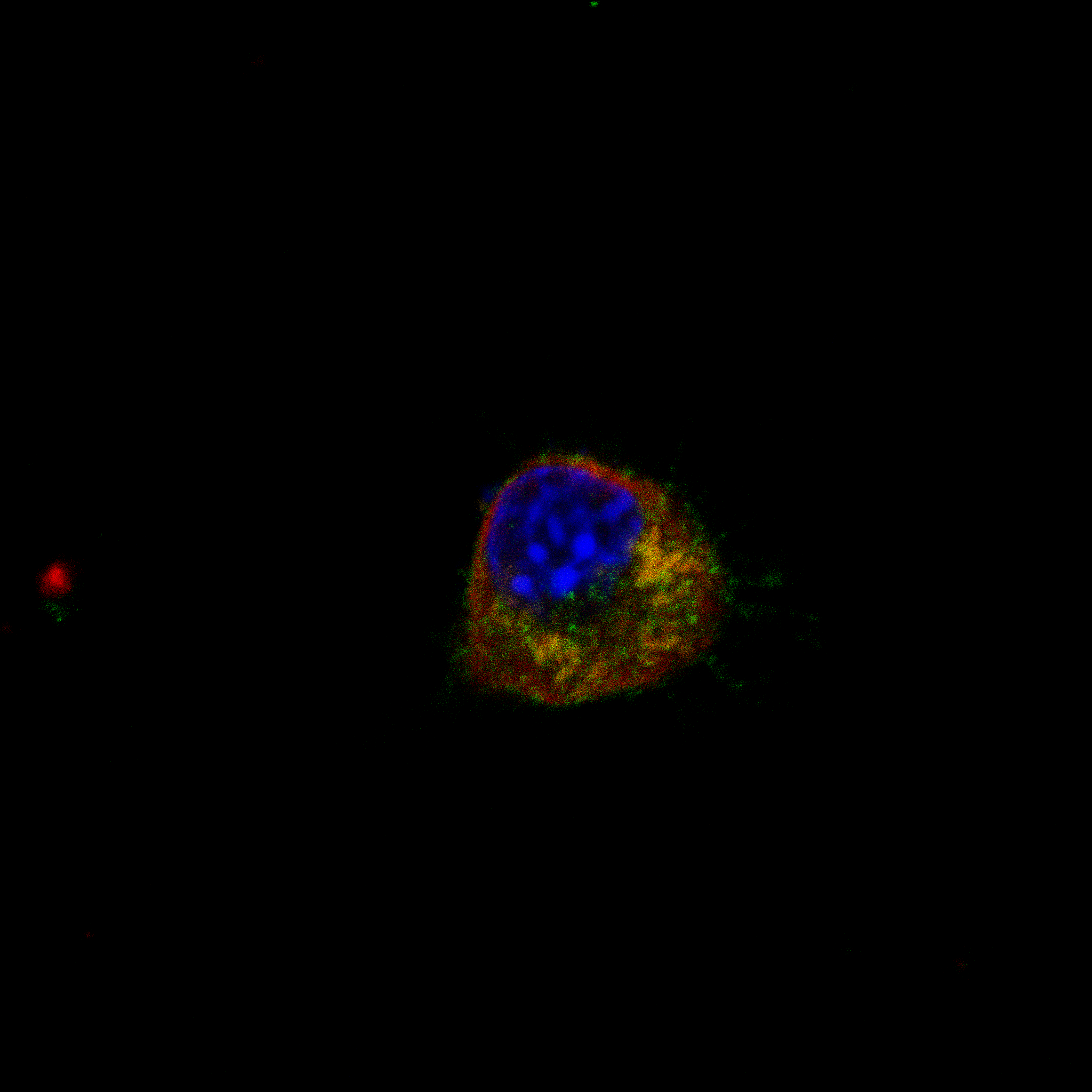

Supplement: S1 File — (ZIP) [file ppat.1012230.s002.zip › S1_File/Fig_3D/Resting/Resting-merge-4.tif]

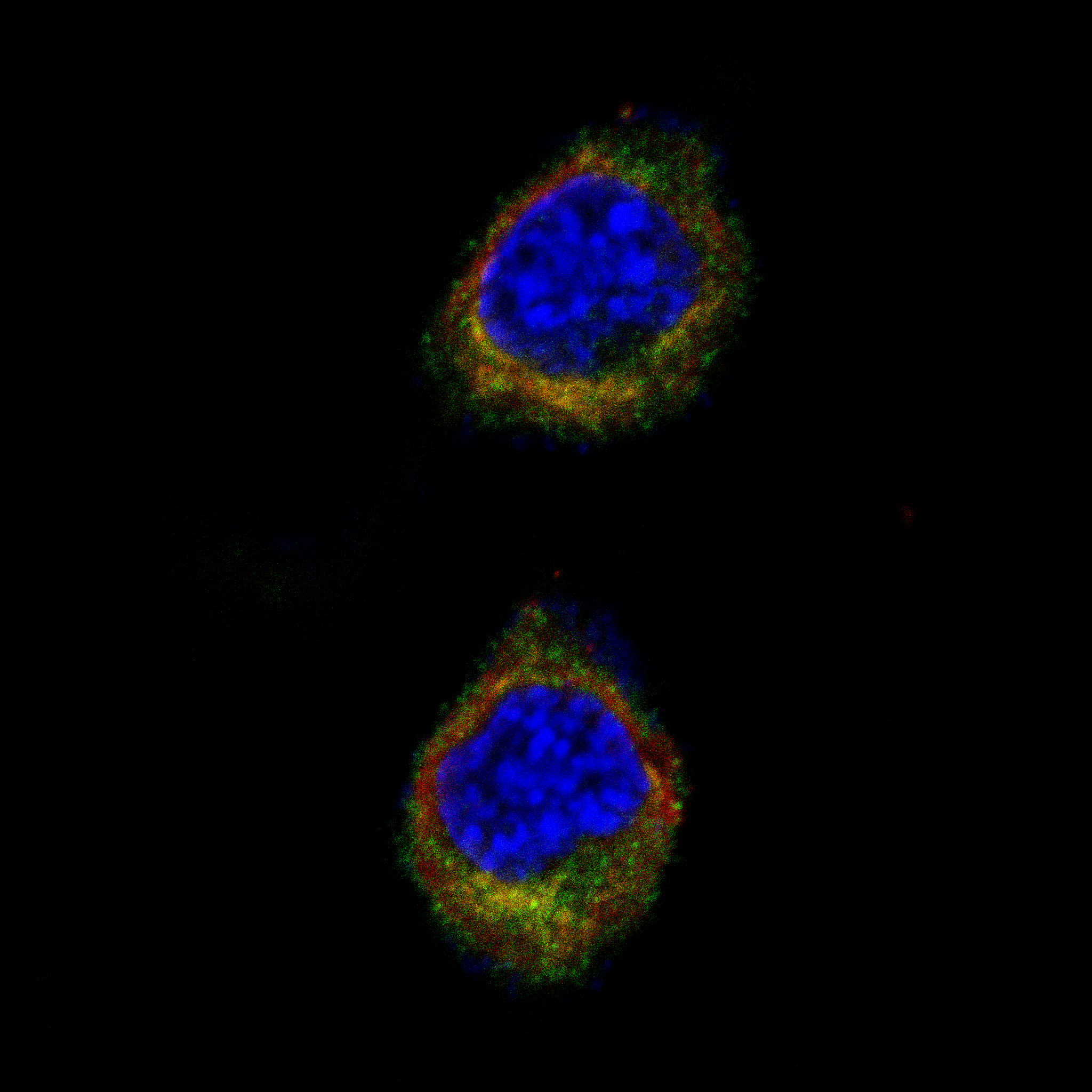

Supplement: S1 File — (ZIP) [file ppat.1012230.s002.zip › S1_File/Fig_3D/Resting/Resting-merge-5.tif]

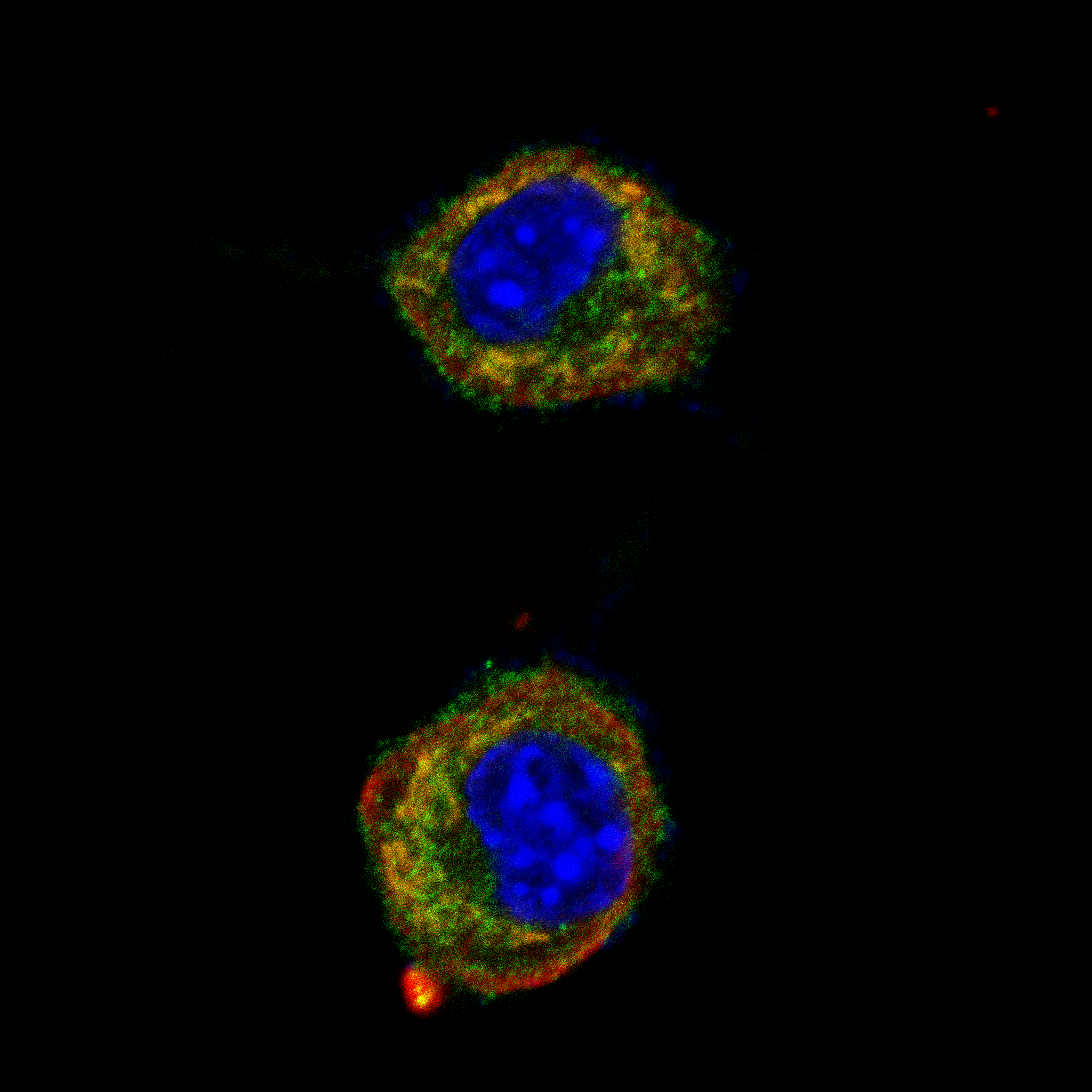

Supplement: S1 File — (ZIP) [file ppat.1012230.s002.zip › S1_File/Fig_3D/Resting/Resting-merge-6.tif]

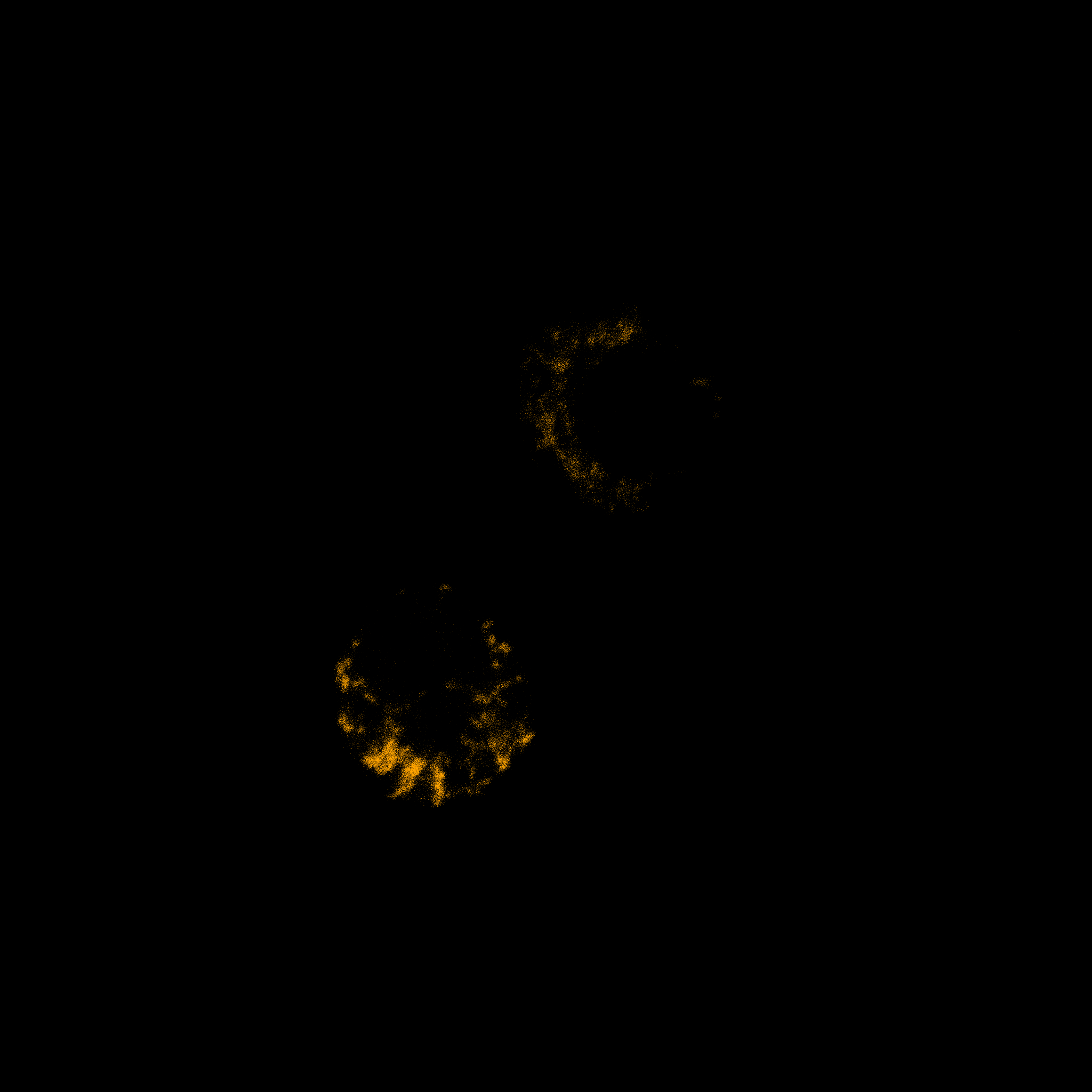

Supplement: S1 File — (ZIP) [file ppat.1012230.s002.zip › S1_File/Fig_3D/Resting/Resting-Mito-1.tif]

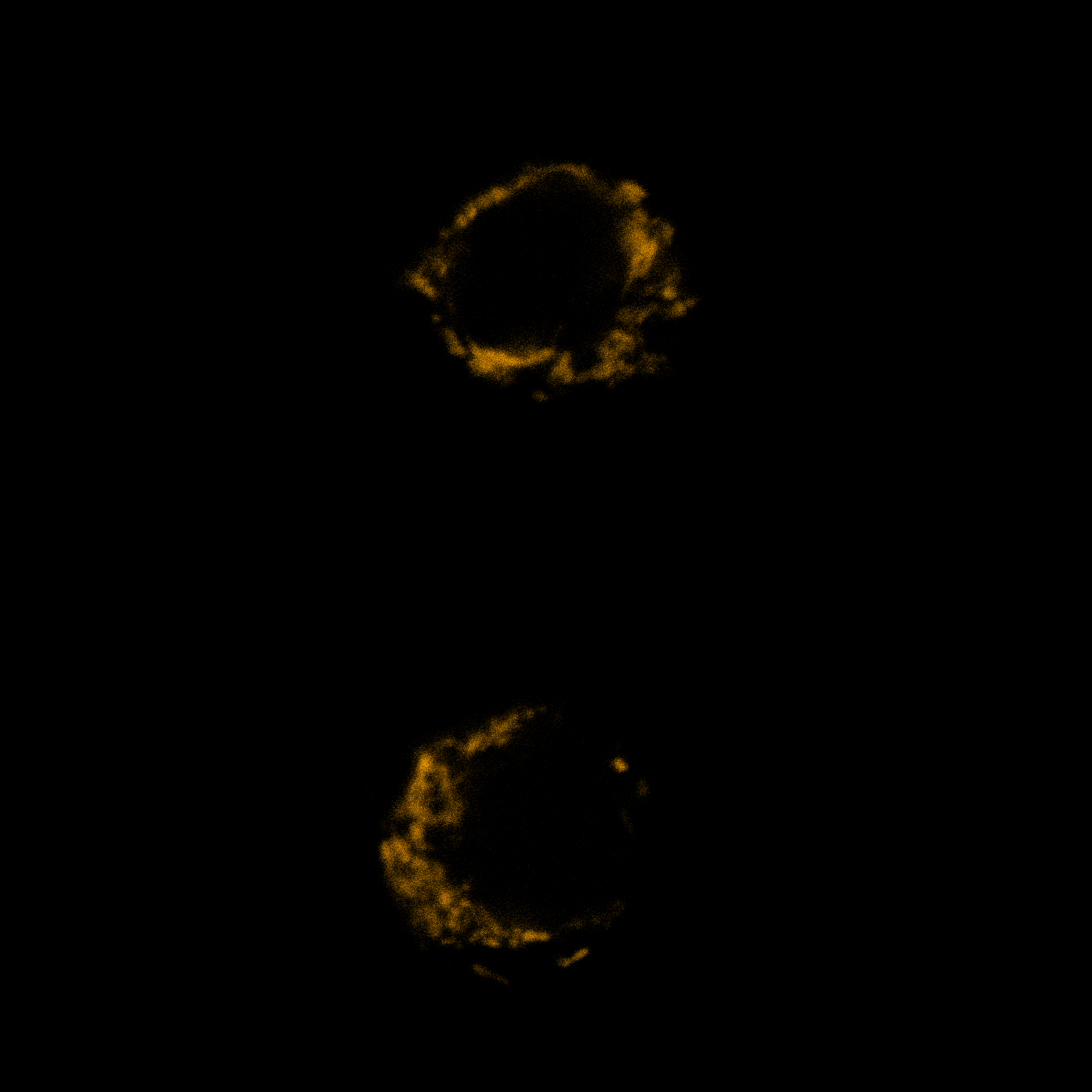

Supplement: S1 File — (ZIP) [file ppat.1012230.s002.zip › S1_File/Fig_3D/Resting/Resting-Mito-2.tif]

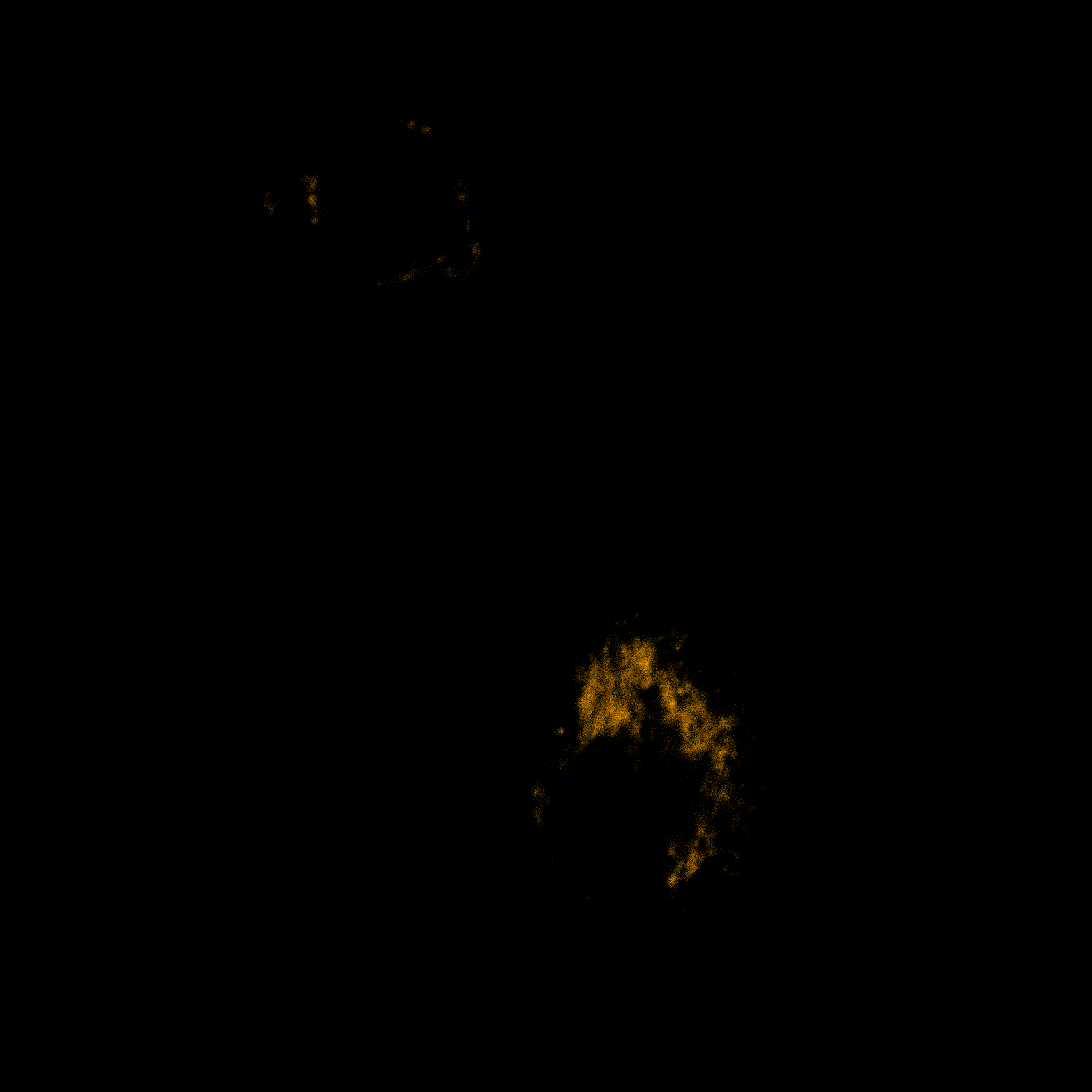

Supplement: S1 File — (ZIP) [file ppat.1012230.s002.zip › S1_File/Fig_3D/Resting/Resting-Mito-3.tif]

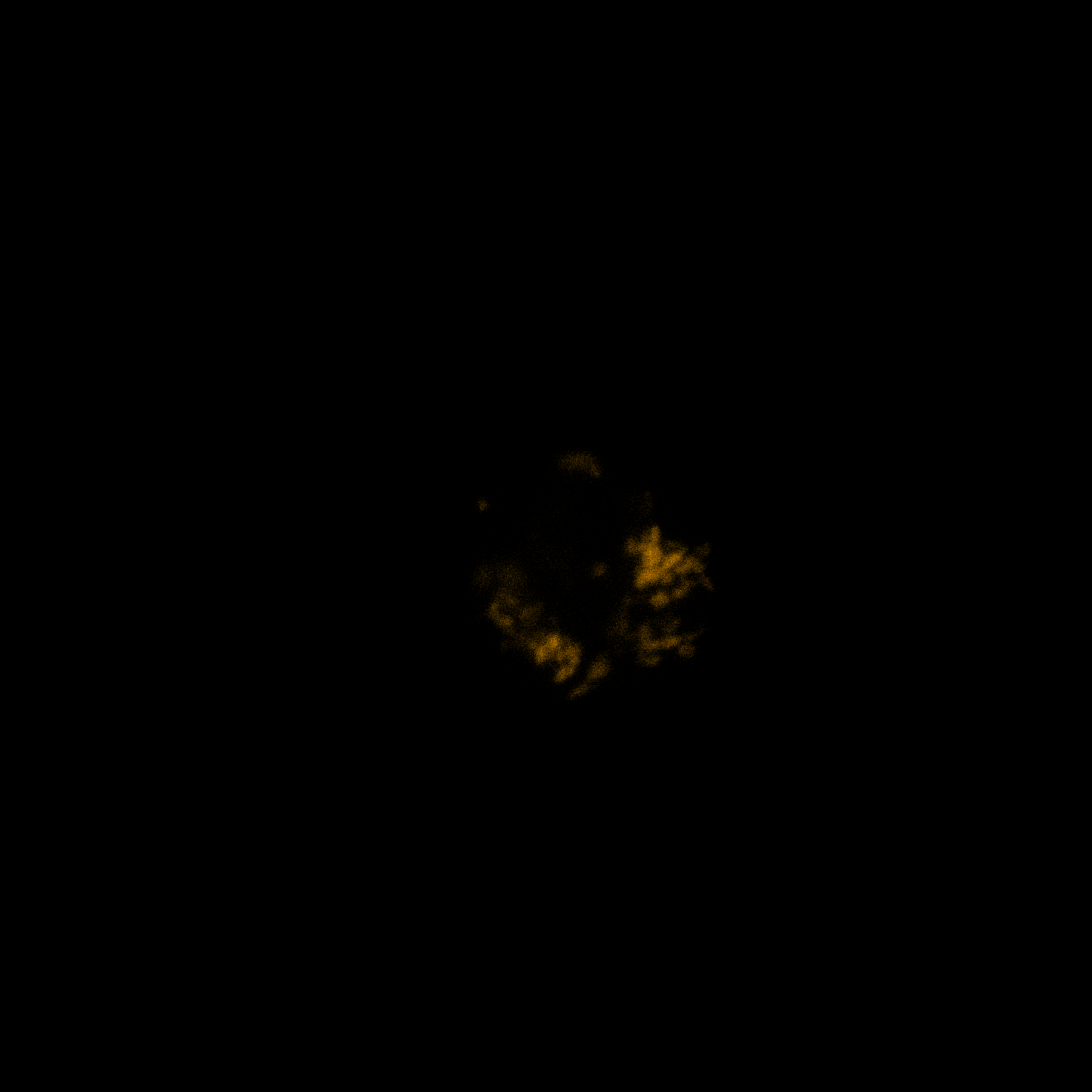

Supplement: S1 File — (ZIP) [file ppat.1012230.s002.zip › S1_File/Fig_3D/Resting/Resting-Mito-4.tif]

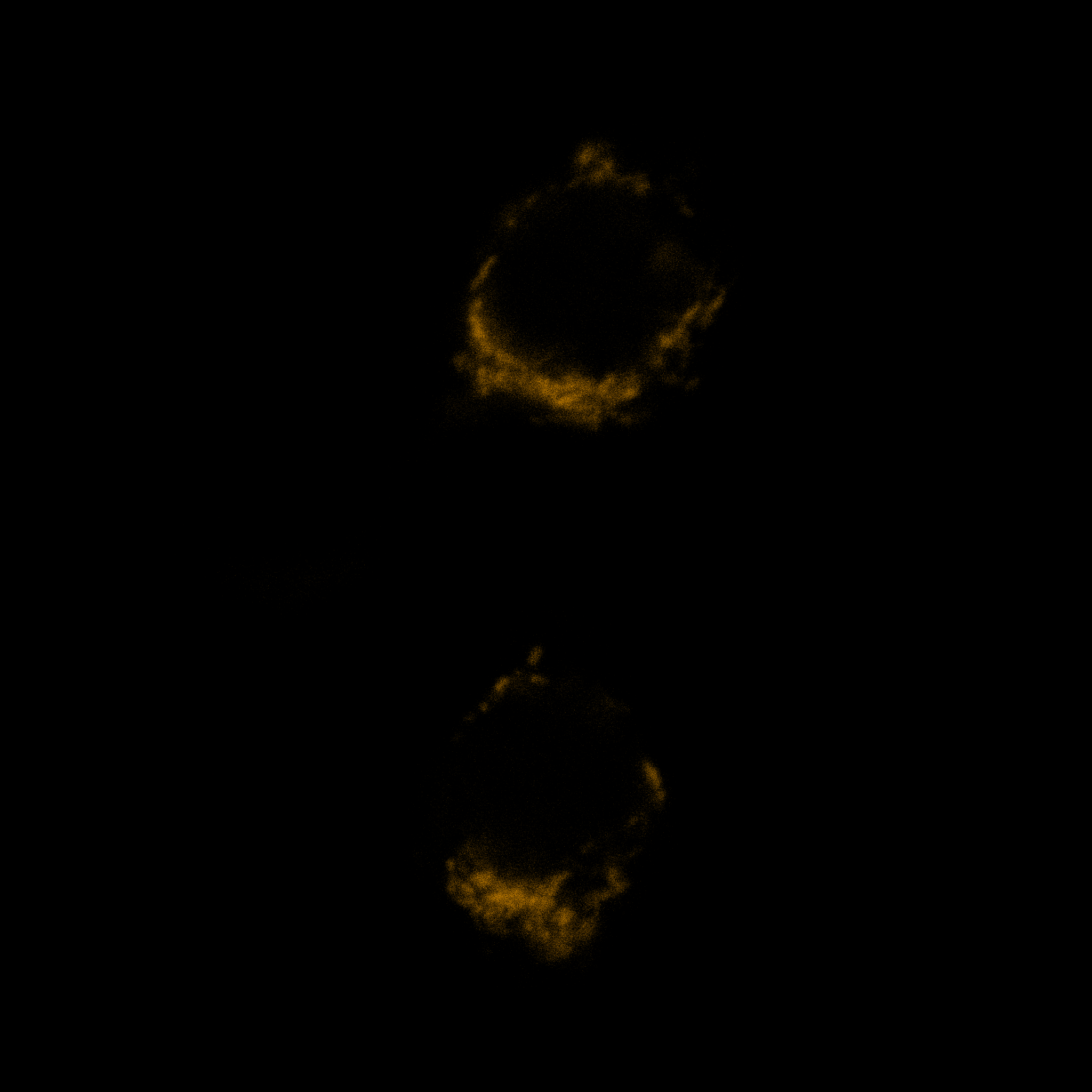

Supplement: S1 File — (ZIP) [file ppat.1012230.s002.zip › S1_File/Fig_3D/Resting/Resting-Mito-5.tif]

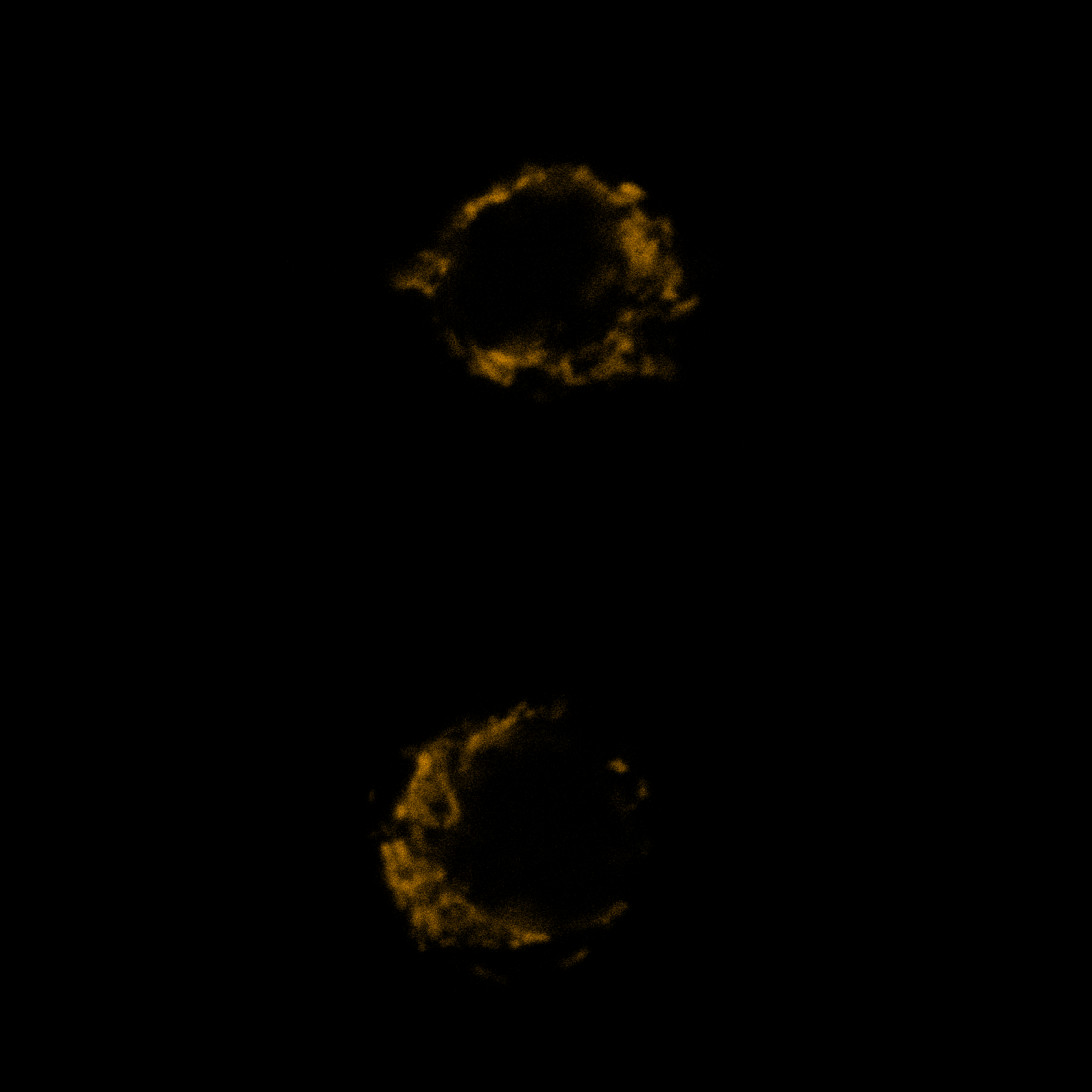

Supplement: S1 File — (ZIP) [file ppat.1012230.s002.zip › S1_File/Fig_3D/Resting/Resting-Mito-6.tif]

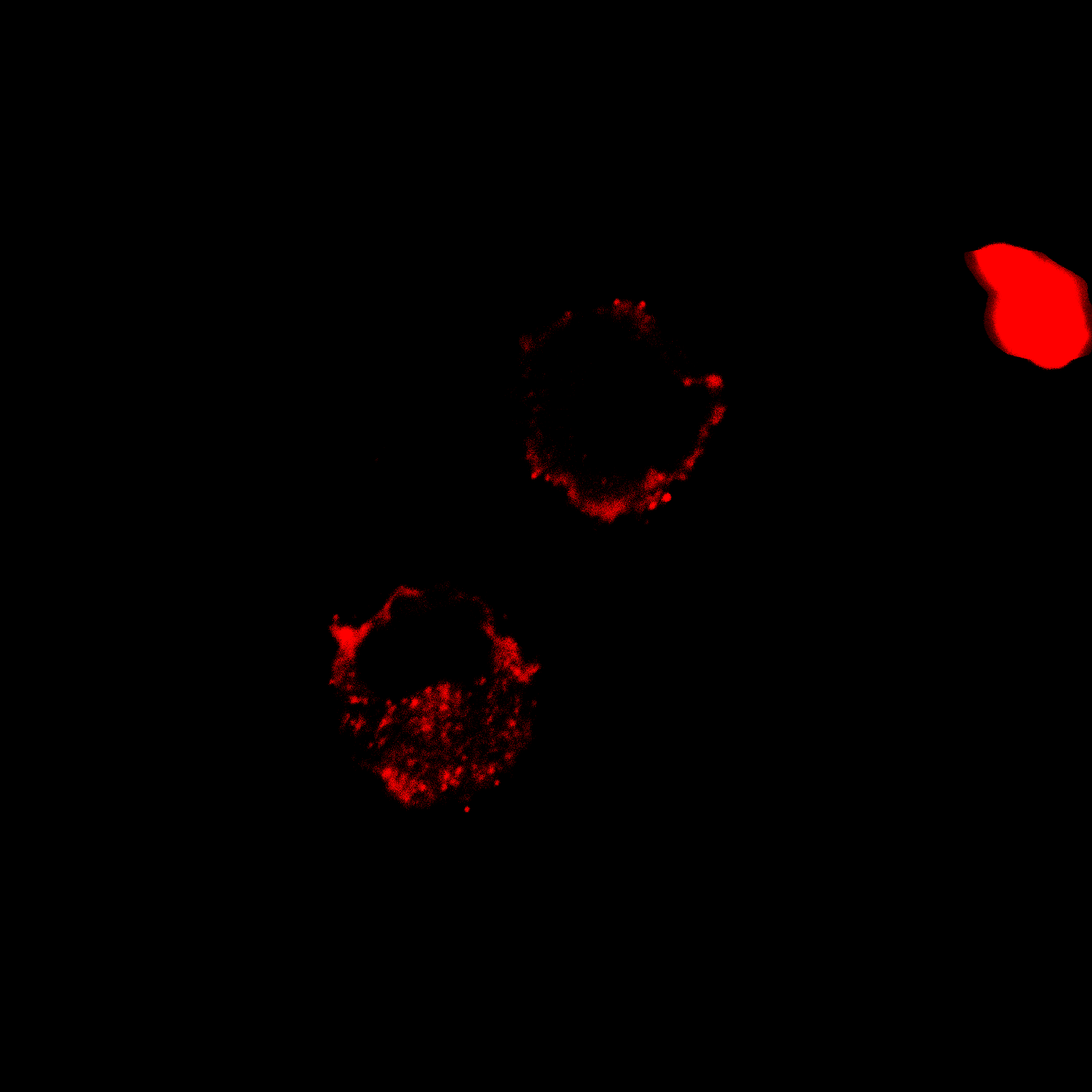

Supplement: S1 File — (ZIP) [file ppat.1012230.s002.zip › S1_File/Fig_3D/Resting/Resting-RIG-I-1.tif]

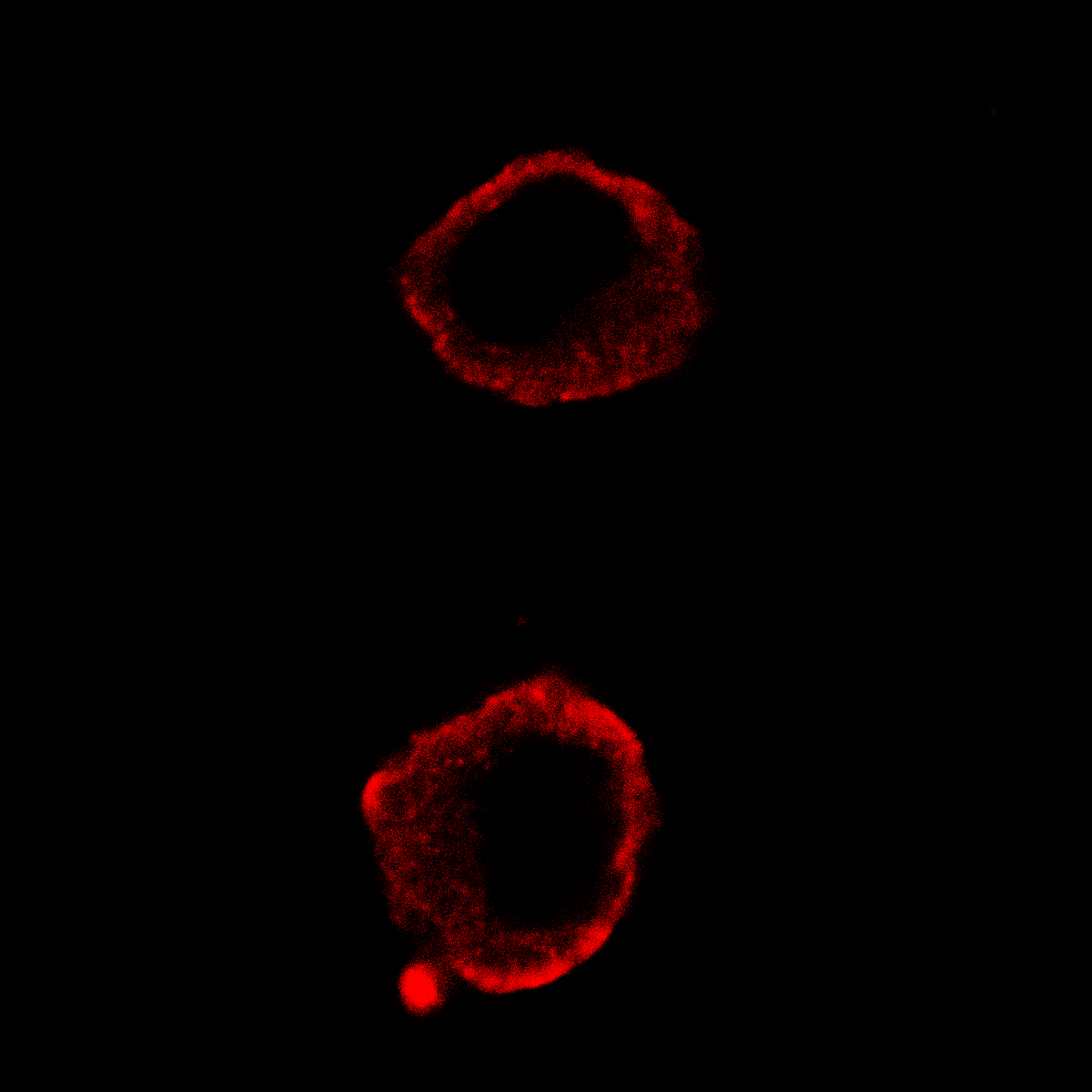

Supplement: S1 File — (ZIP) [file ppat.1012230.s002.zip › S1_File/Fig_3D/Resting/Resting-RIG-I-2.tif]

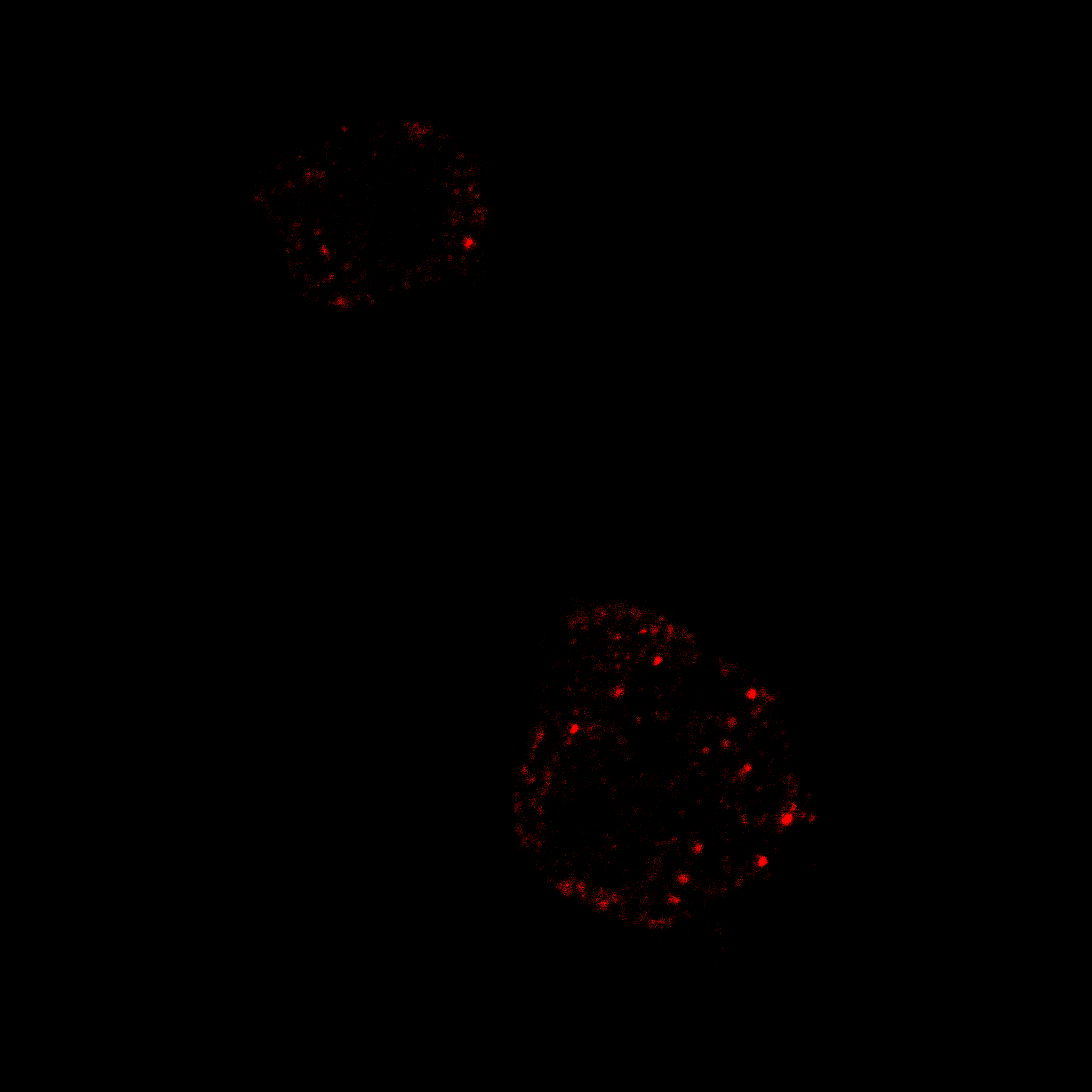

Supplement: S1 File — (ZIP) [file ppat.1012230.s002.zip › S1_File/Fig_3D/Resting/Resting-RIG-I-3.tif]

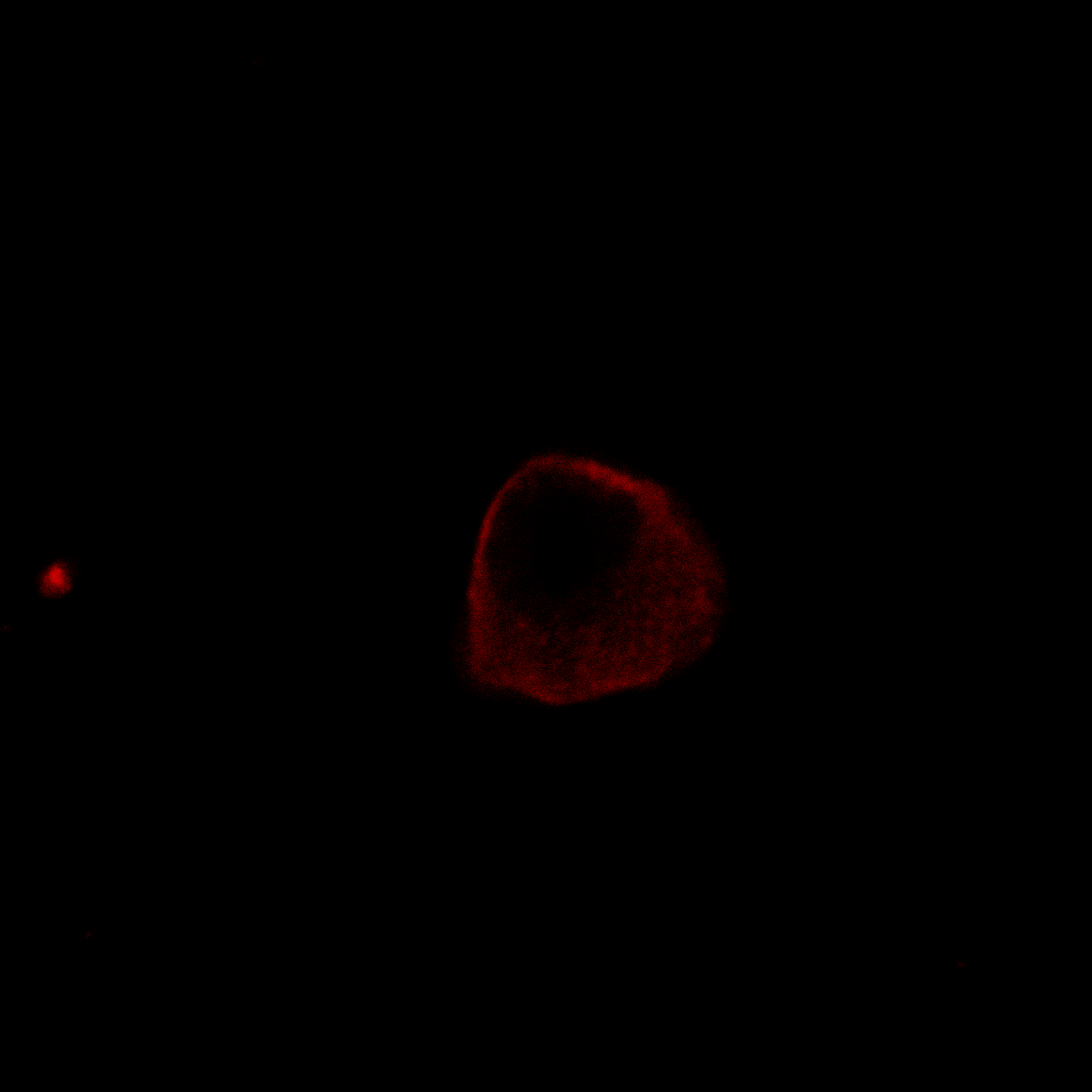

Supplement: S1 File — (ZIP) [file ppat.1012230.s002.zip › S1_File/Fig_3D/Resting/Resting-RIG-I-4.tif]

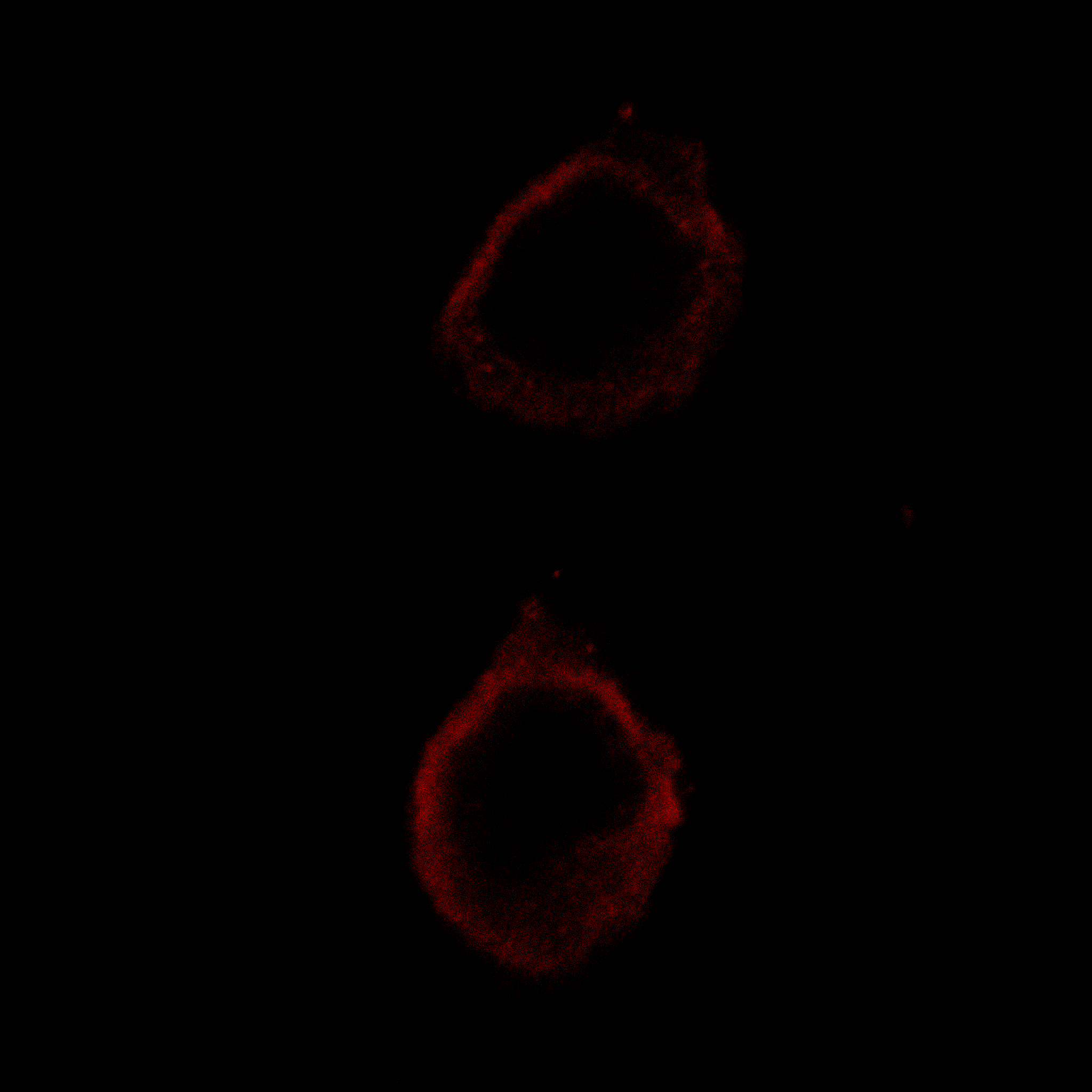

Supplement: S1 File — (ZIP) [file ppat.1012230.s002.zip › S1_File/Fig_3D/Resting/Resting-RIG-I-5.tif]

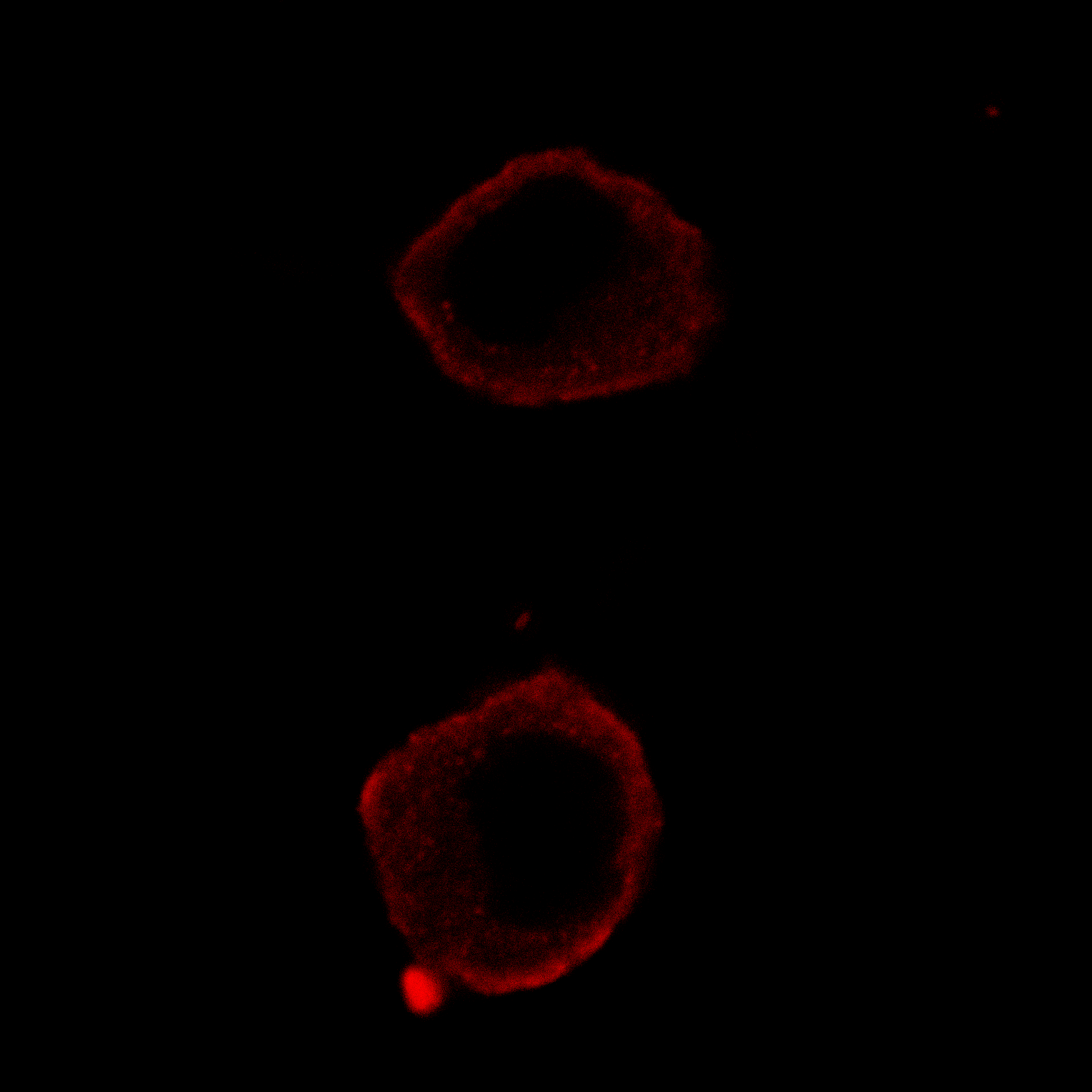

Supplement: S1 File — (ZIP) [file ppat.1012230.s002.zip › S1_File/Fig_3D/Resting/Resting-RIG-I-6.tif]

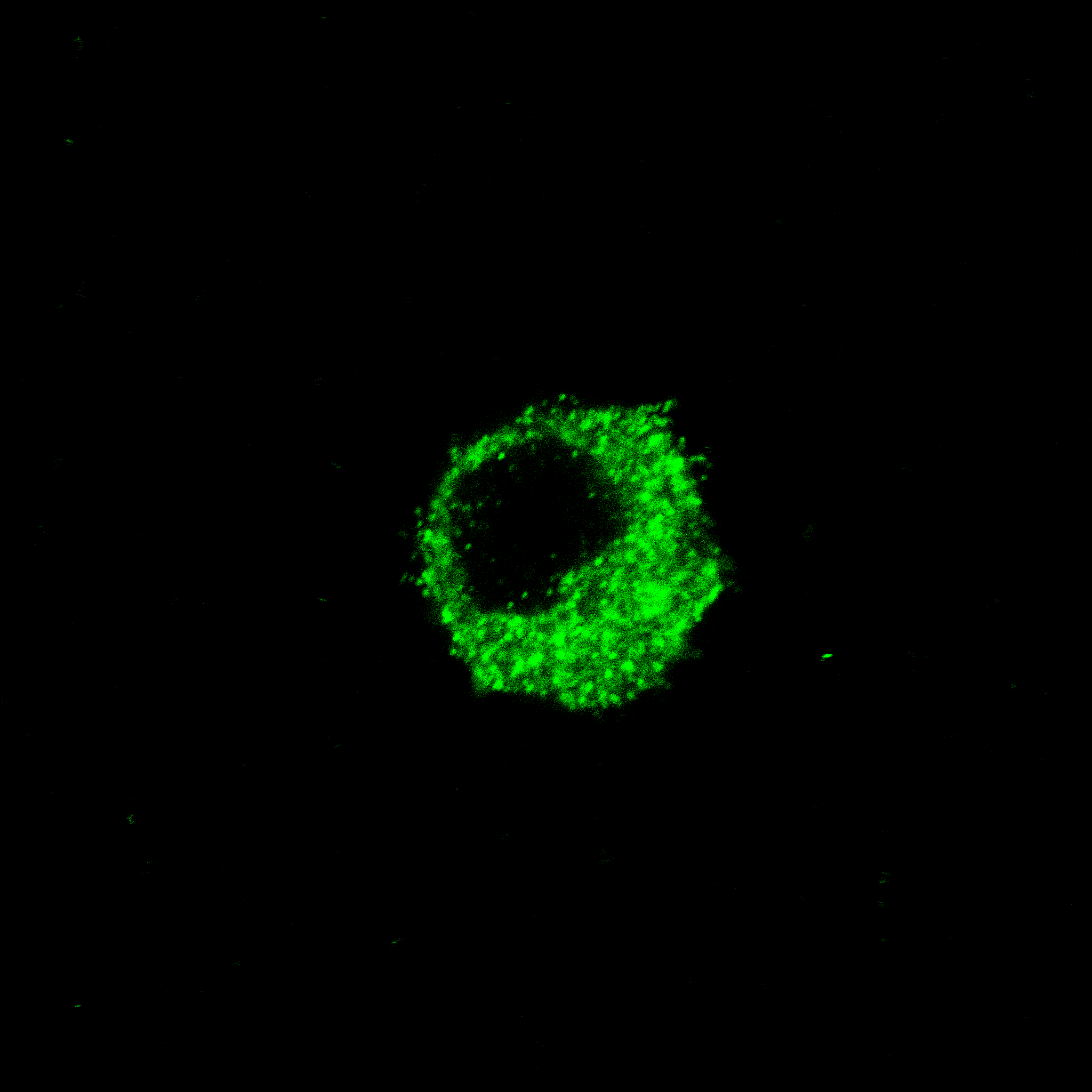

Supplement: S1 File — (ZIP) [file ppat.1012230.s002.zip › S1_File/Fig_3D/SeV/SeV-ADAP-1.tif]

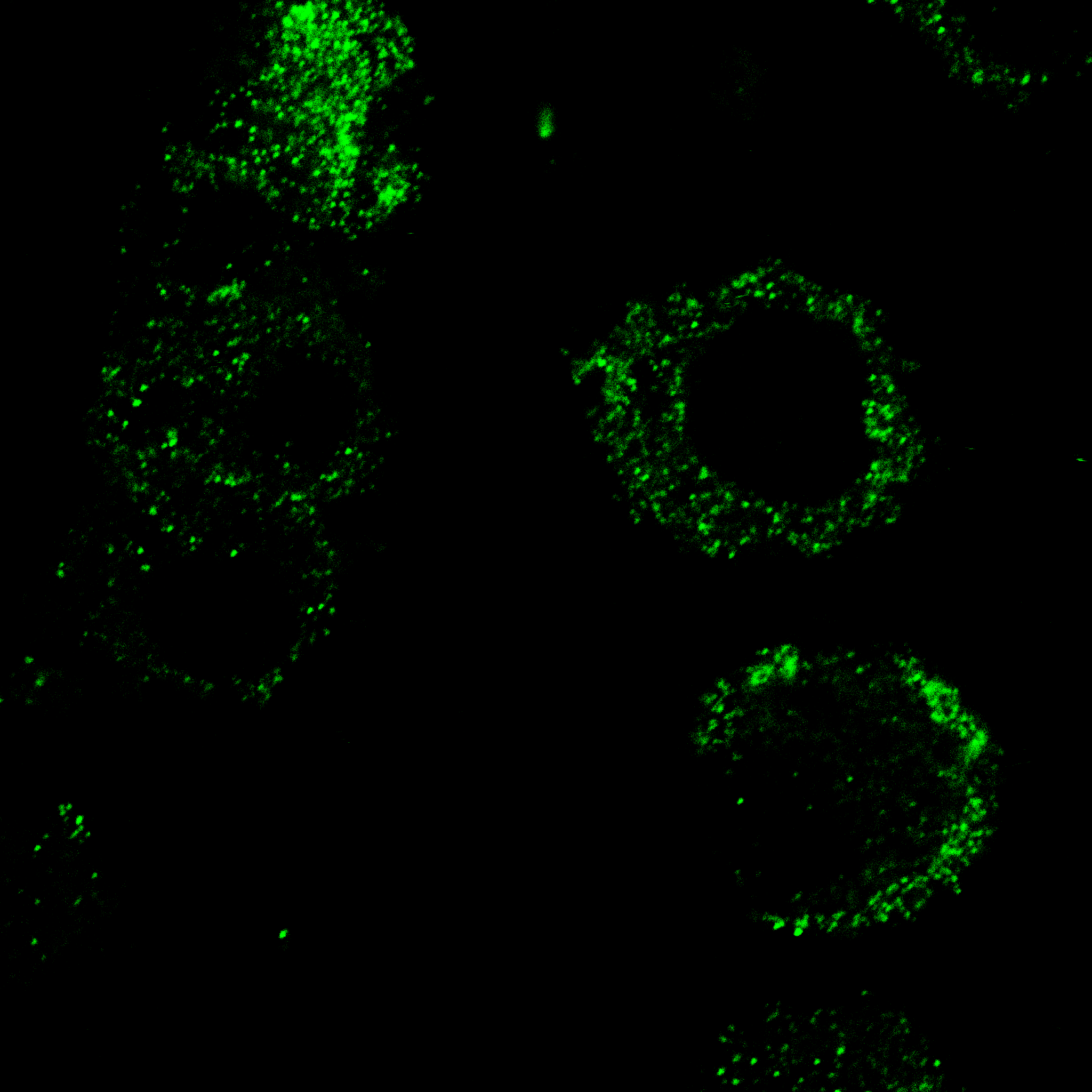

Supplement: S1 File — (ZIP) [file ppat.1012230.s002.zip › S1_File/Fig_3D/SeV/SeV-ADAP-2.tif]

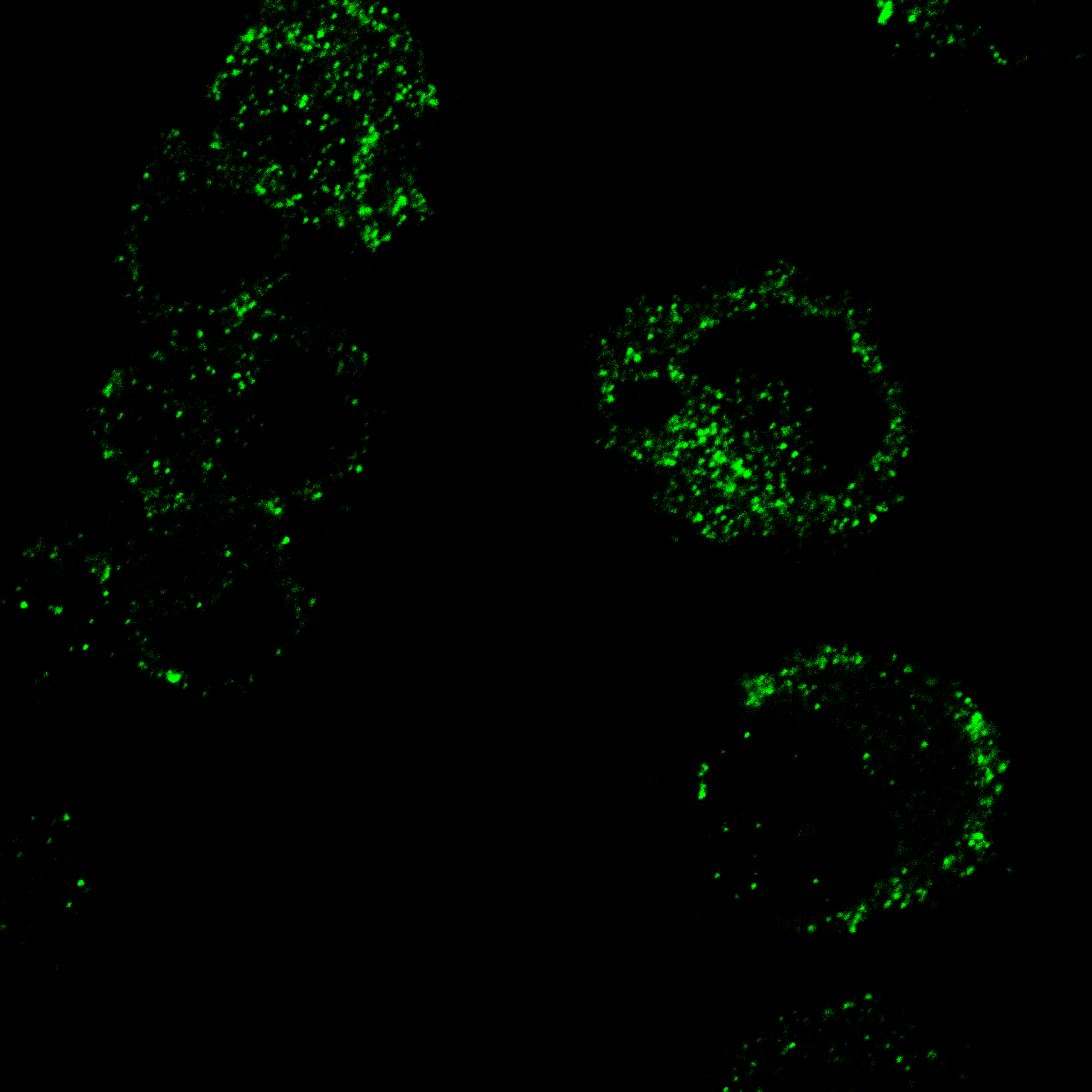

Supplement: S1 File — (ZIP) [file ppat.1012230.s002.zip › S1_File/Fig_3D/SeV/SeV-ADAP-3.tif]

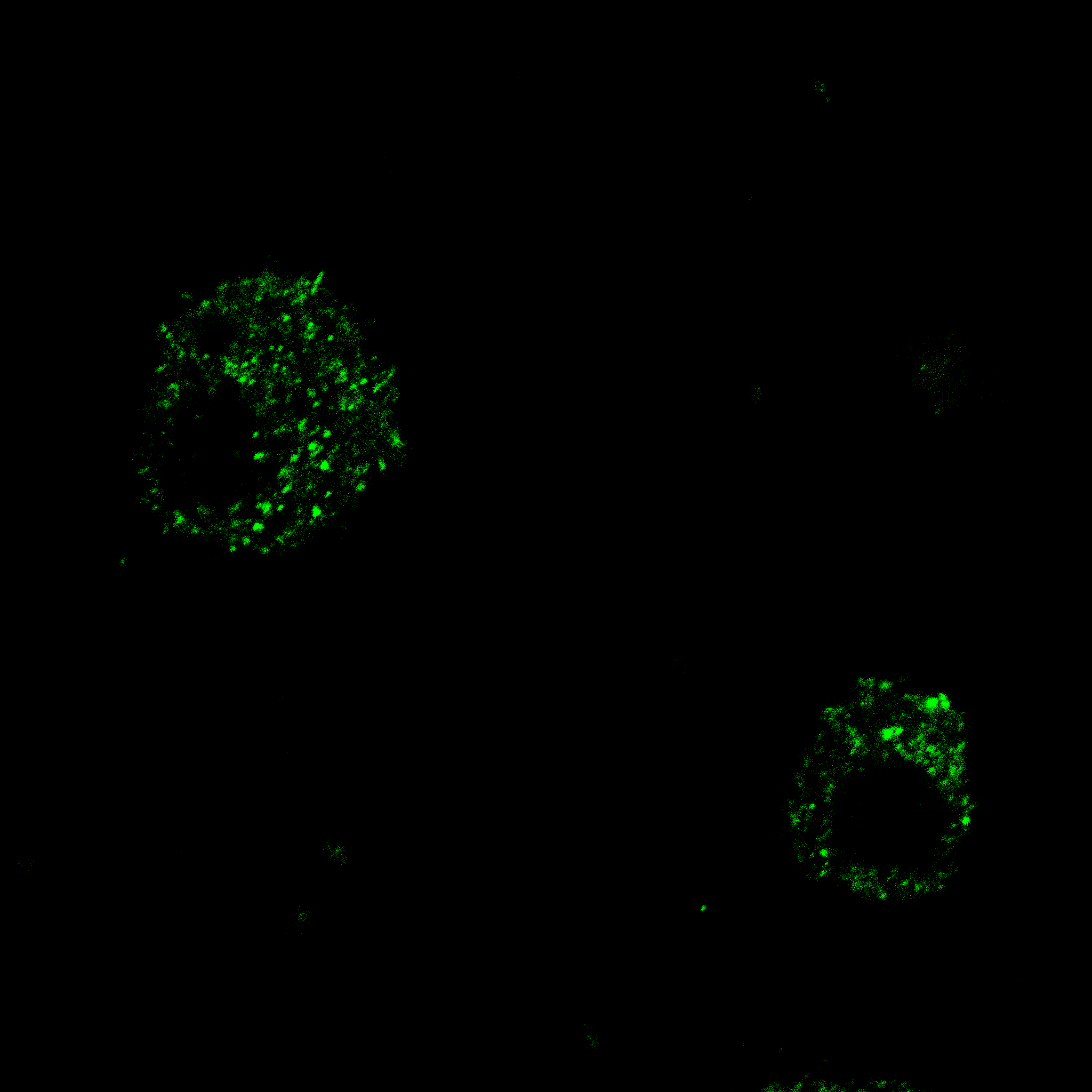

Supplement: S1 File — (ZIP) [file ppat.1012230.s002.zip › S1_File/Fig_3D/SeV/SeV-ADAP-4.tif]

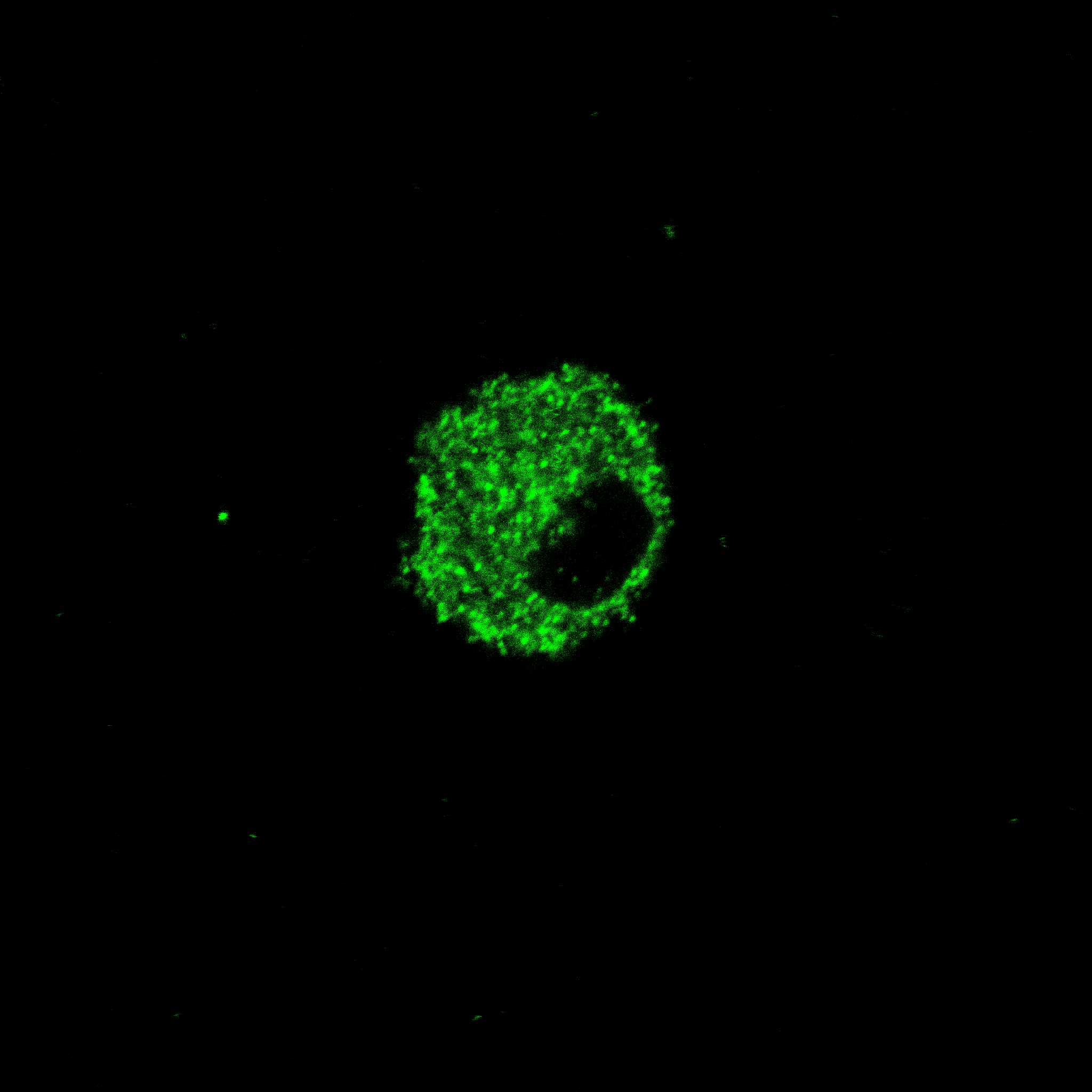

Supplement: S1 File — (ZIP) [file ppat.1012230.s002.zip › S1_File/Fig_3D/SeV/SeV-ADAP-5.tif]

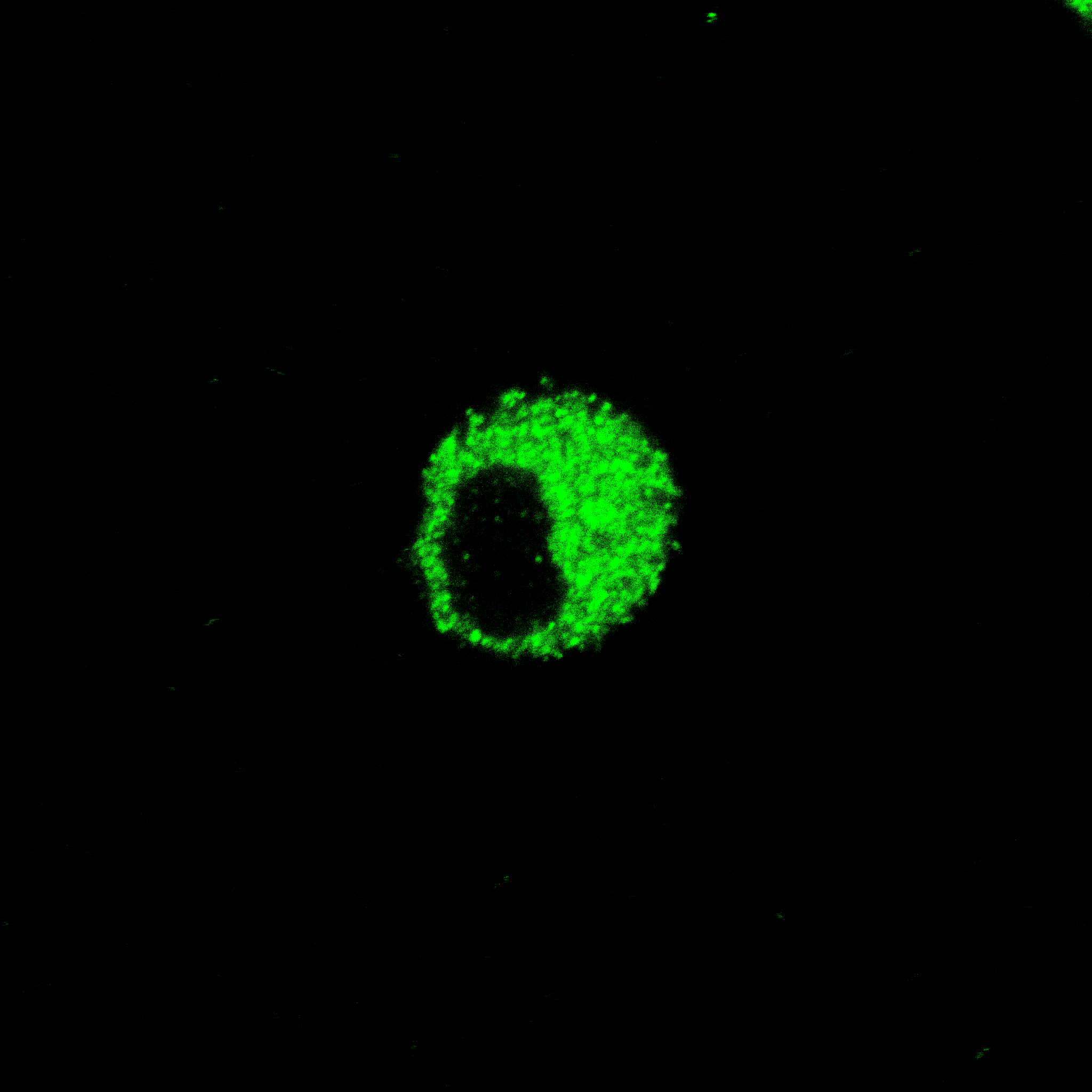

Supplement: S1 File — (ZIP) [file ppat.1012230.s002.zip › S1_File/Fig_3D/SeV/SeV-ADAP-6.tif]

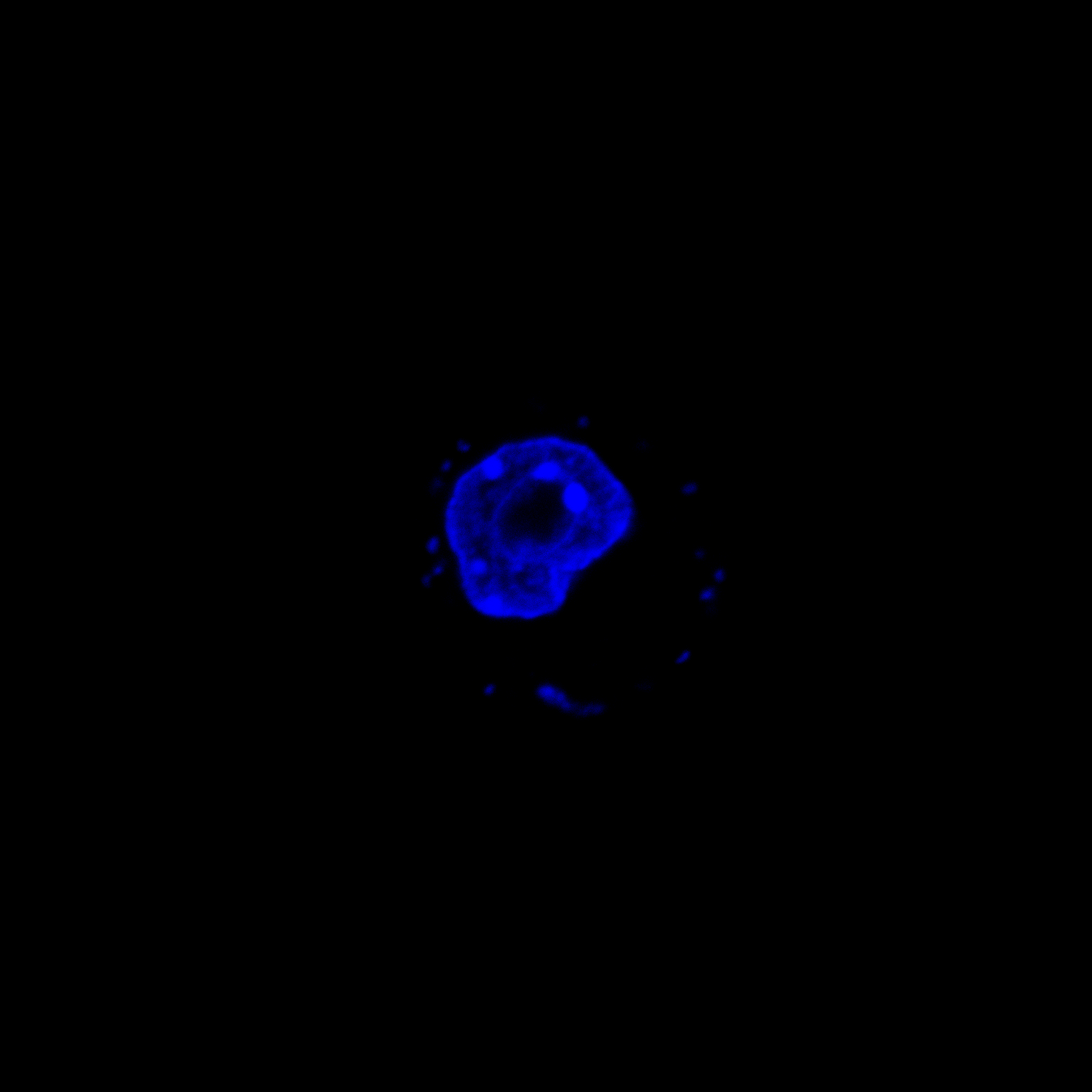

Supplement: S1 File — (ZIP) [file ppat.1012230.s002.zip › S1_File/Fig_3D/SeV/SeV-DAPI-1.tif]

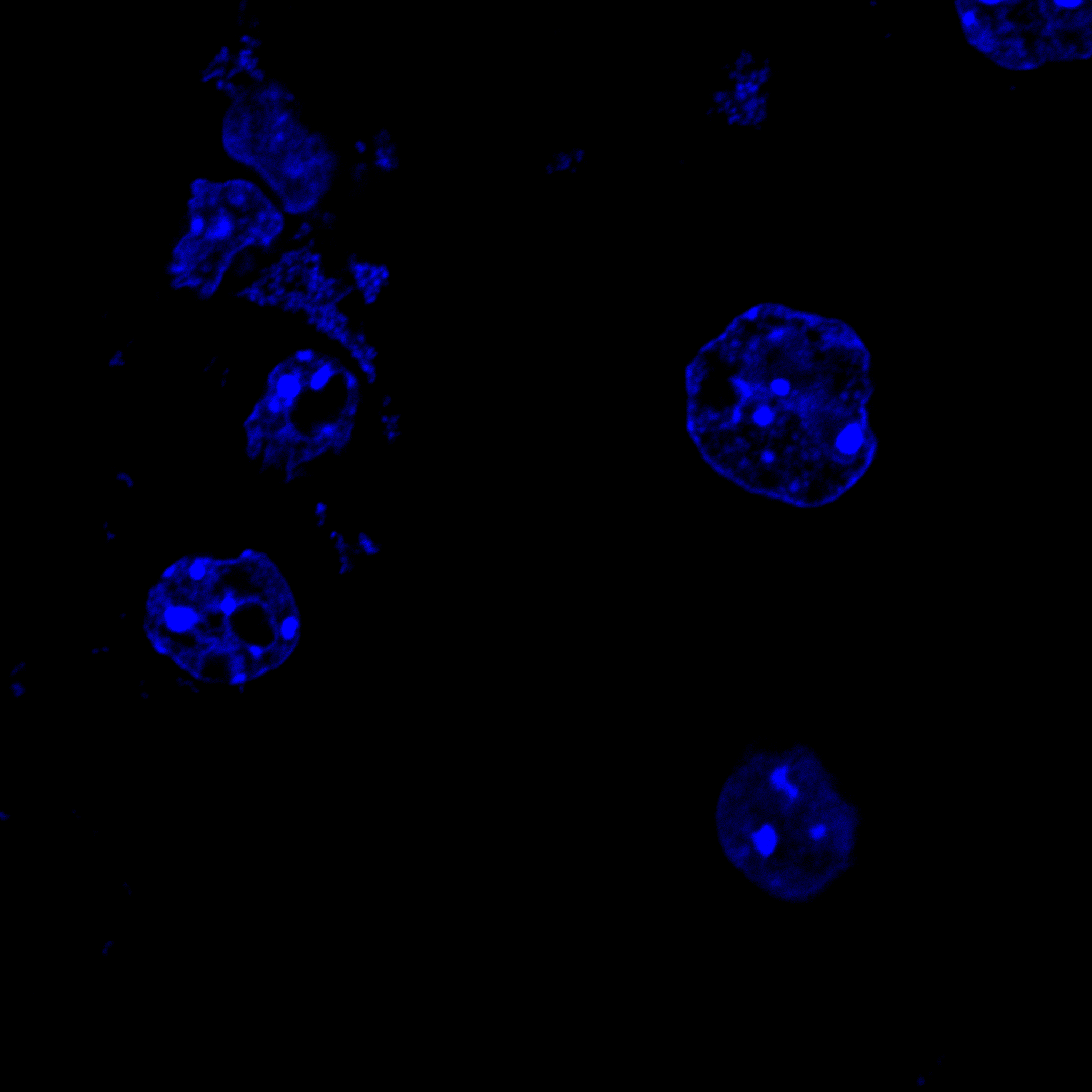

Supplement: S1 File — (ZIP) [file ppat.1012230.s002.zip › S1_File/Fig_3D/SeV/SeV-DAPI-2.tif]

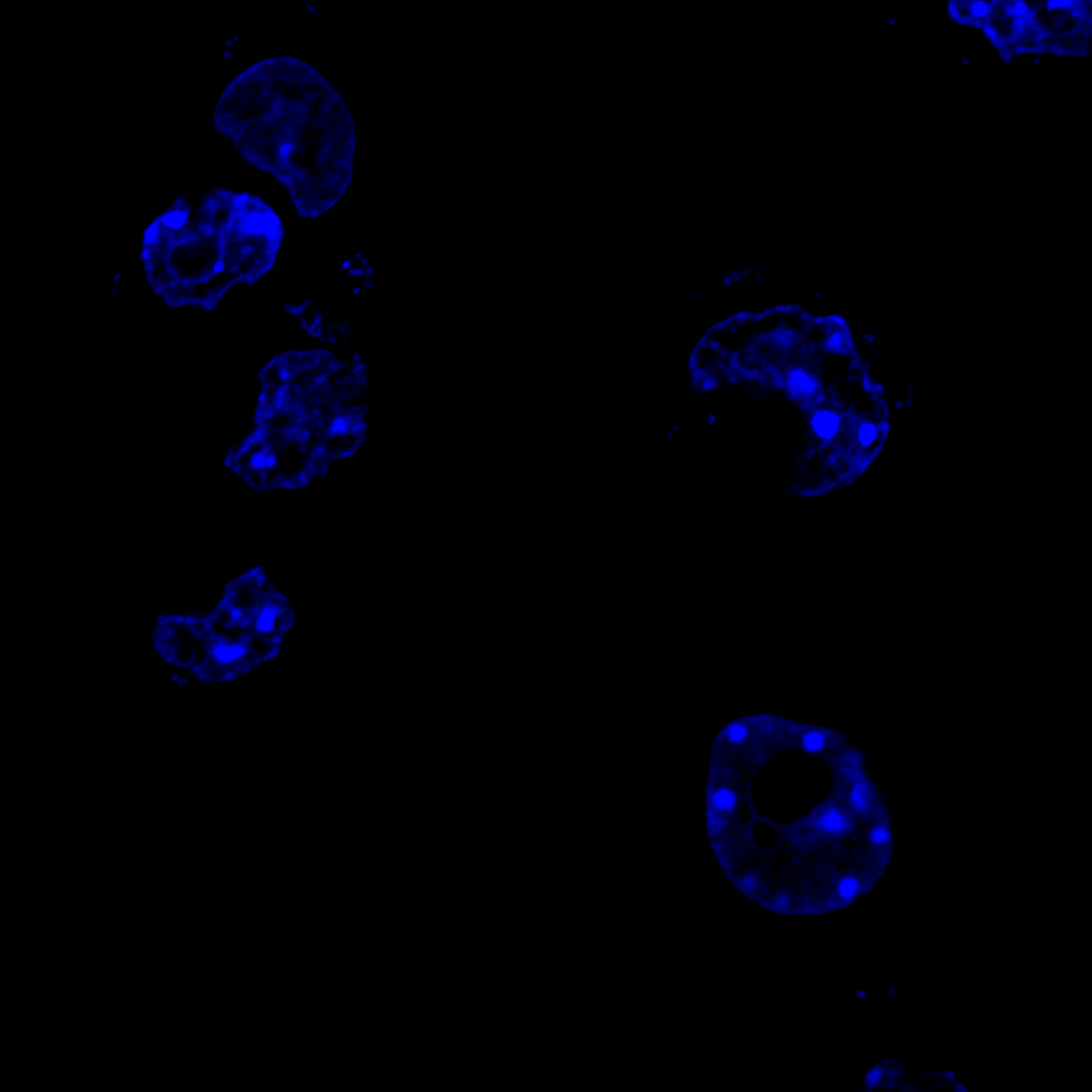

Supplement: S1 File — (ZIP) [file ppat.1012230.s002.zip › S1_File/Fig_3D/SeV/SeV-DAPI-3.tif]

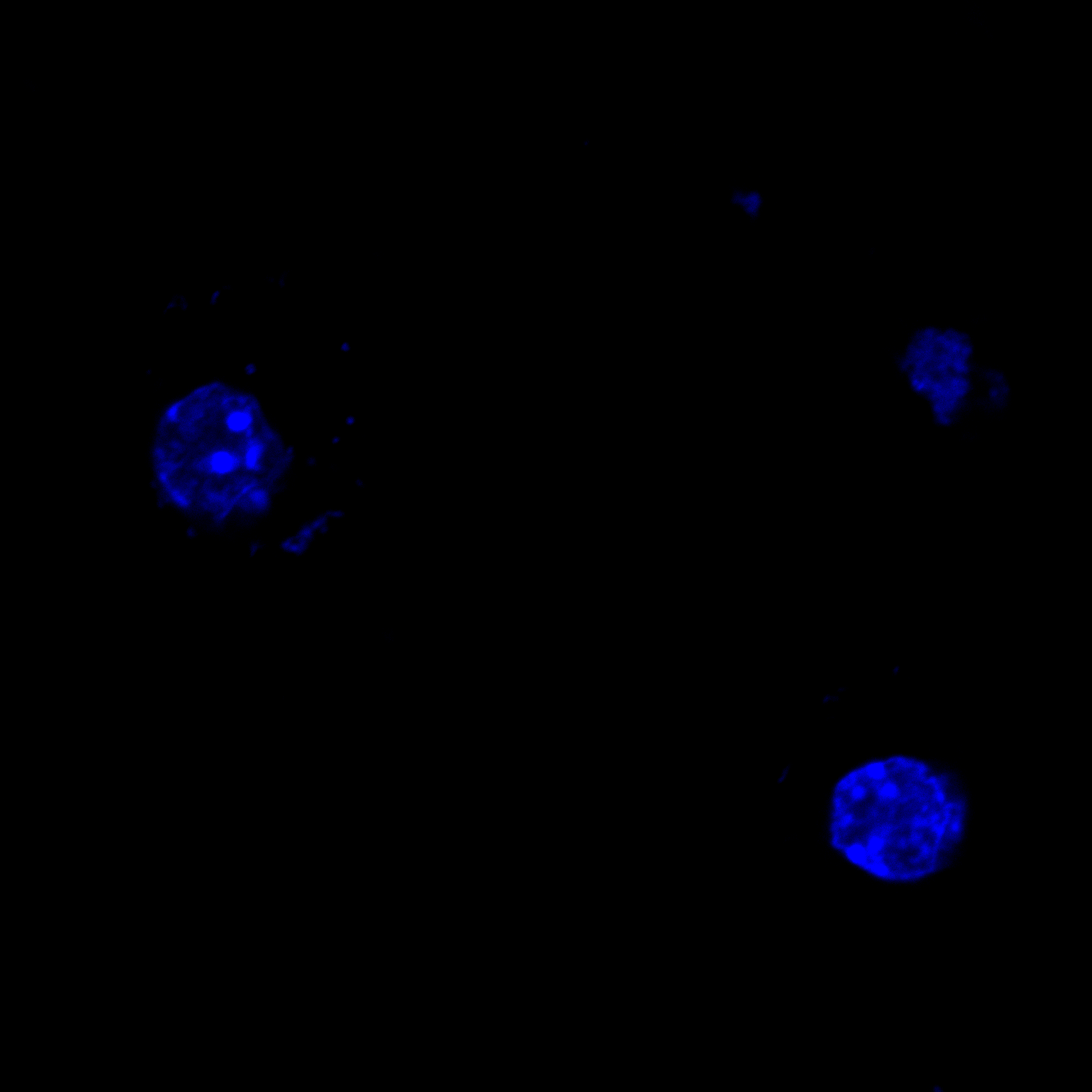

Supplement: S1 File — (ZIP) [file ppat.1012230.s002.zip › S1_File/Fig_3D/SeV/SeV-DAPI-4.tif]

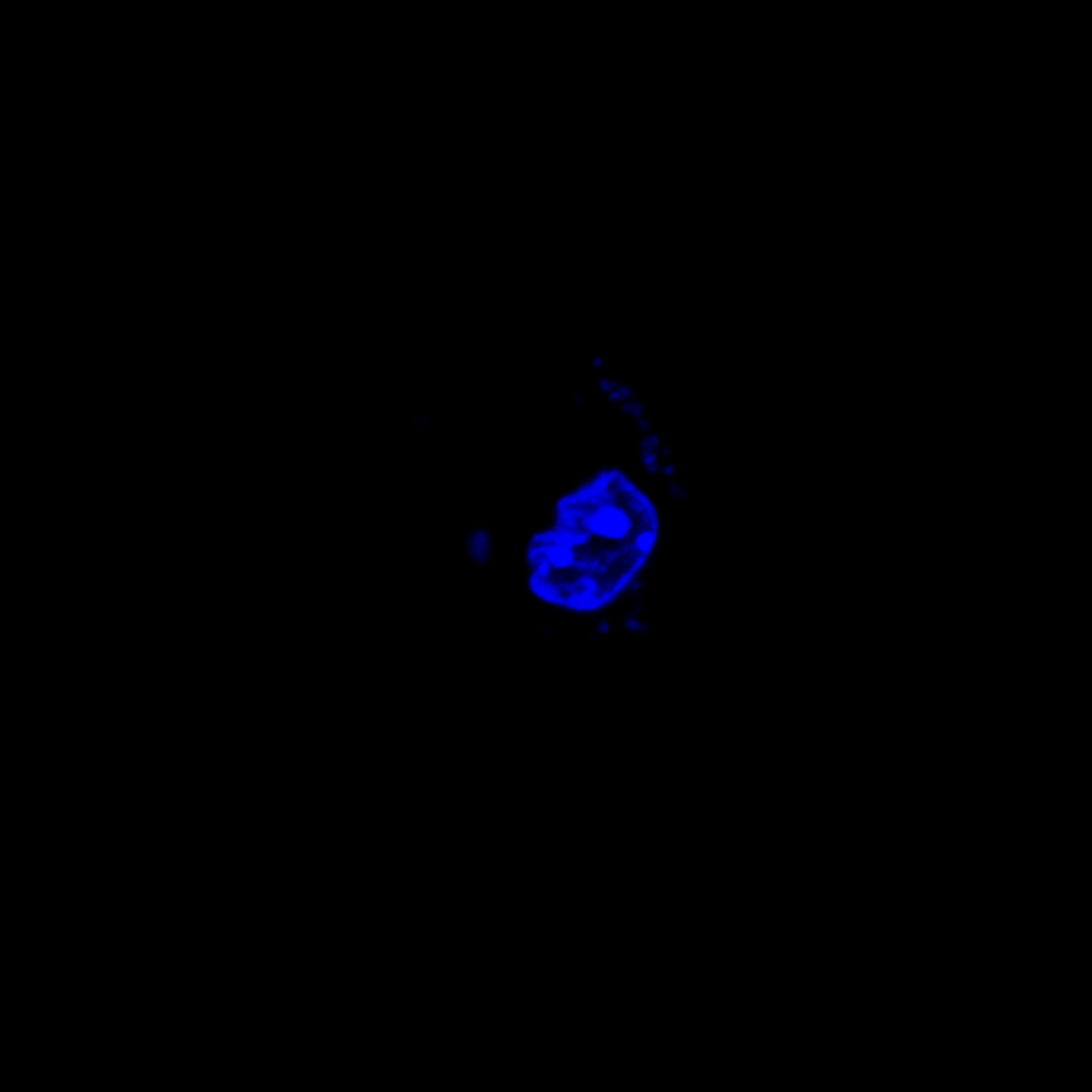

Supplement: S1 File — (ZIP) [file ppat.1012230.s002.zip › S1_File/Fig_3D/SeV/SeV-DAPI-5.tif]

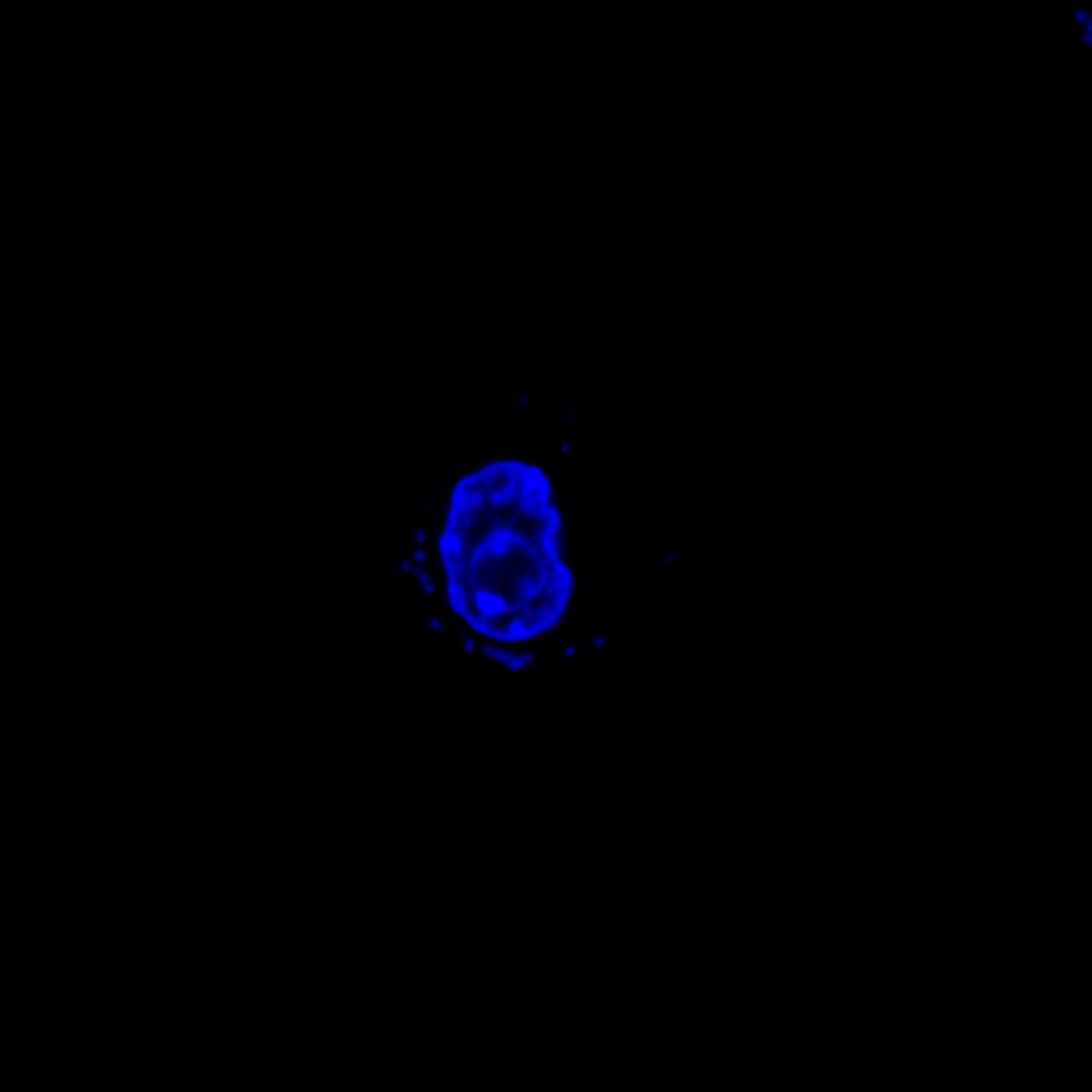

Supplement: S1 File — (ZIP) [file ppat.1012230.s002.zip › S1_File/Fig_3D/SeV/SeV-DAPI-6.tif]

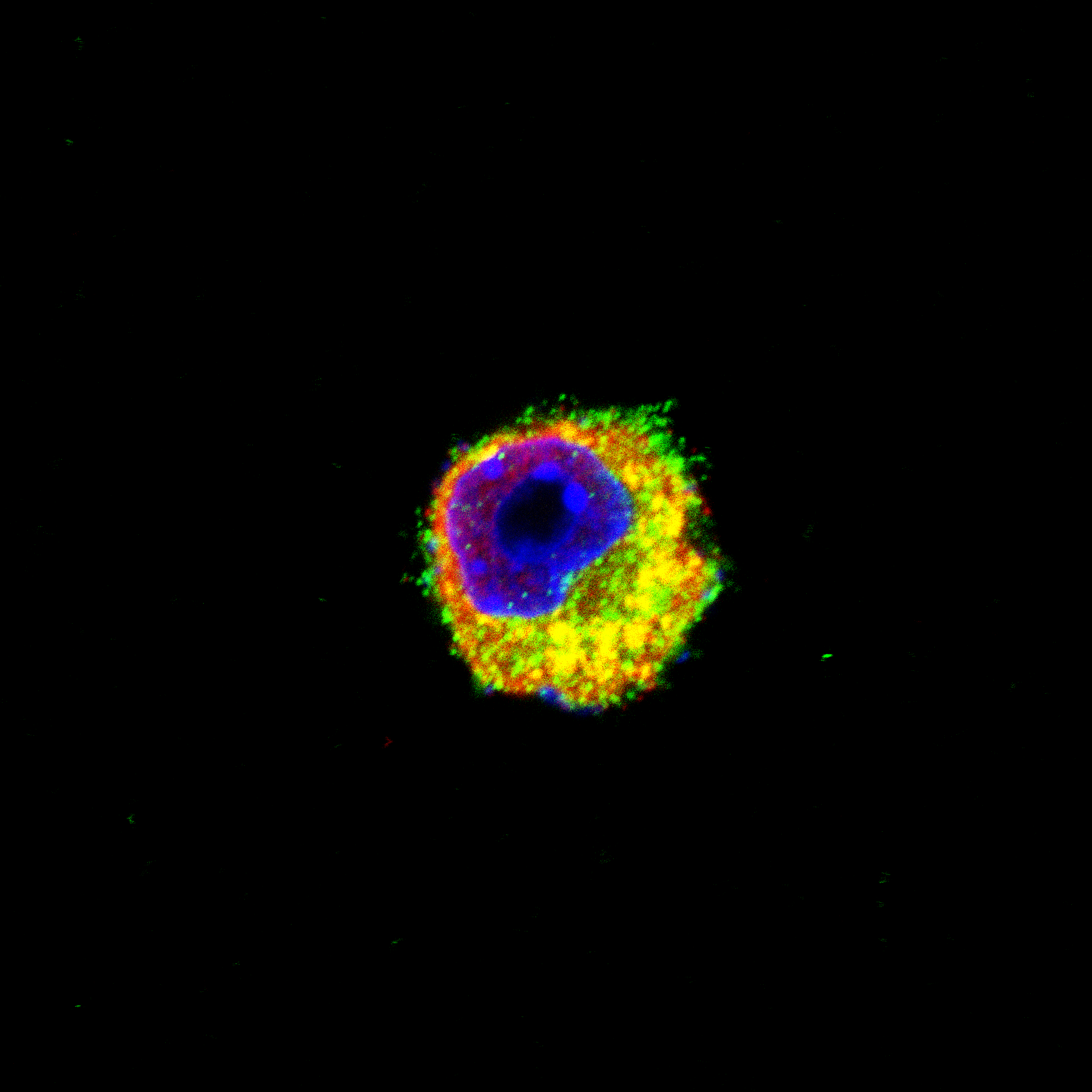

Supplement: S1 File — (ZIP) [file ppat.1012230.s002.zip › S1_File/Fig_3D/SeV/SeV-merge-1.tif]

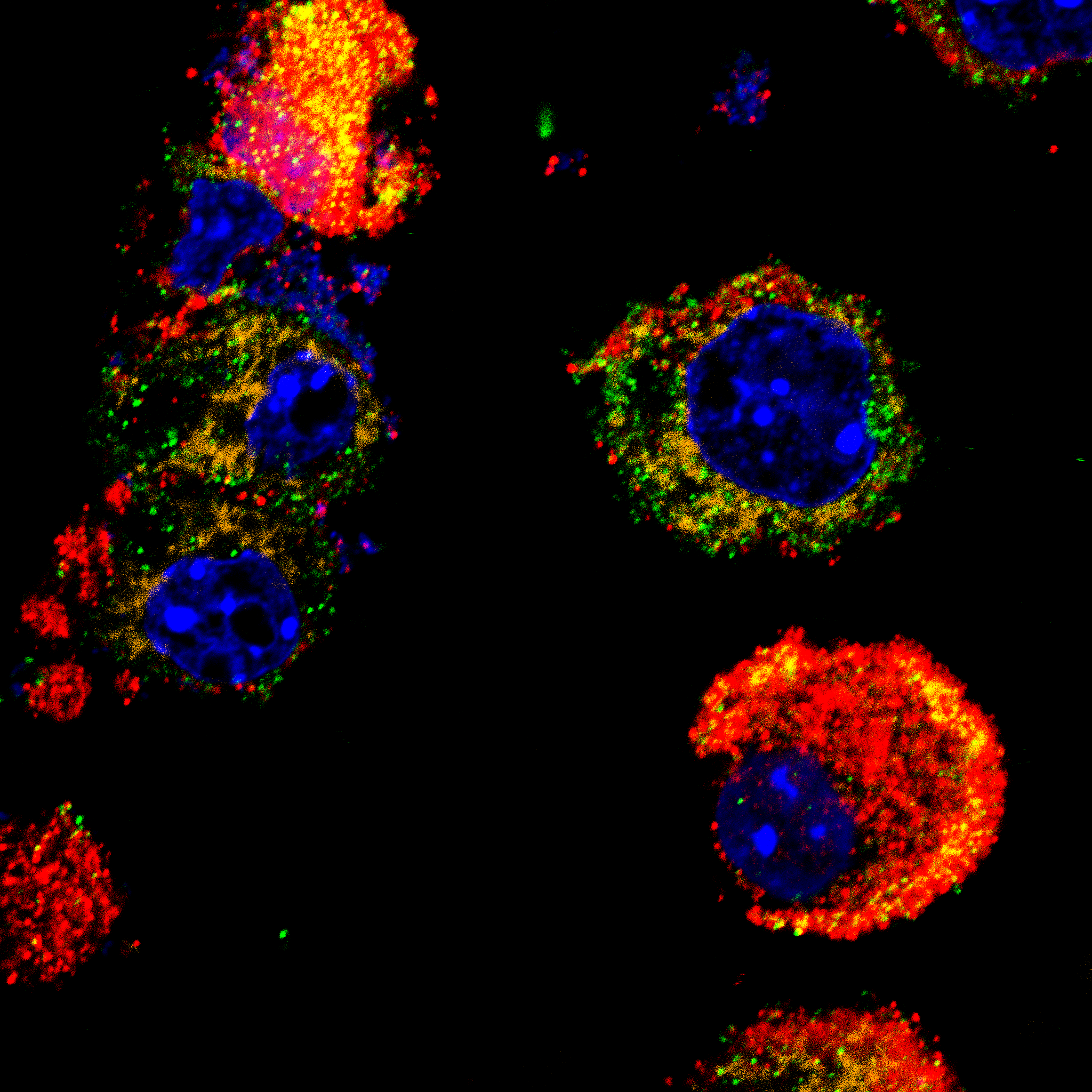

Supplement: S1 File — (ZIP) [file ppat.1012230.s002.zip › S1_File/Fig_3D/SeV/SeV-merge-2.tif]

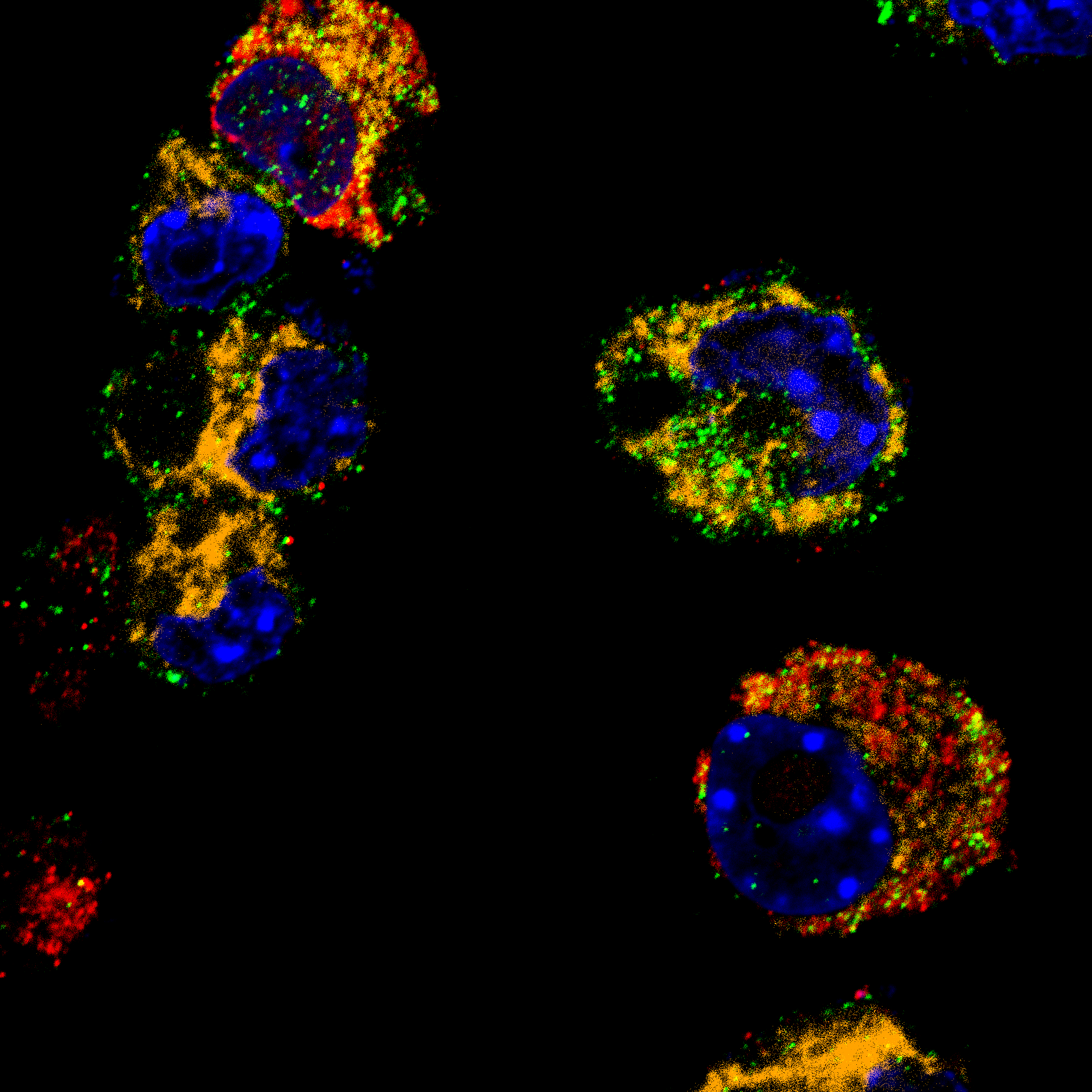

Supplement: S1 File — (ZIP) [file ppat.1012230.s002.zip › S1_File/Fig_3D/SeV/SeV-merge-3.tif]

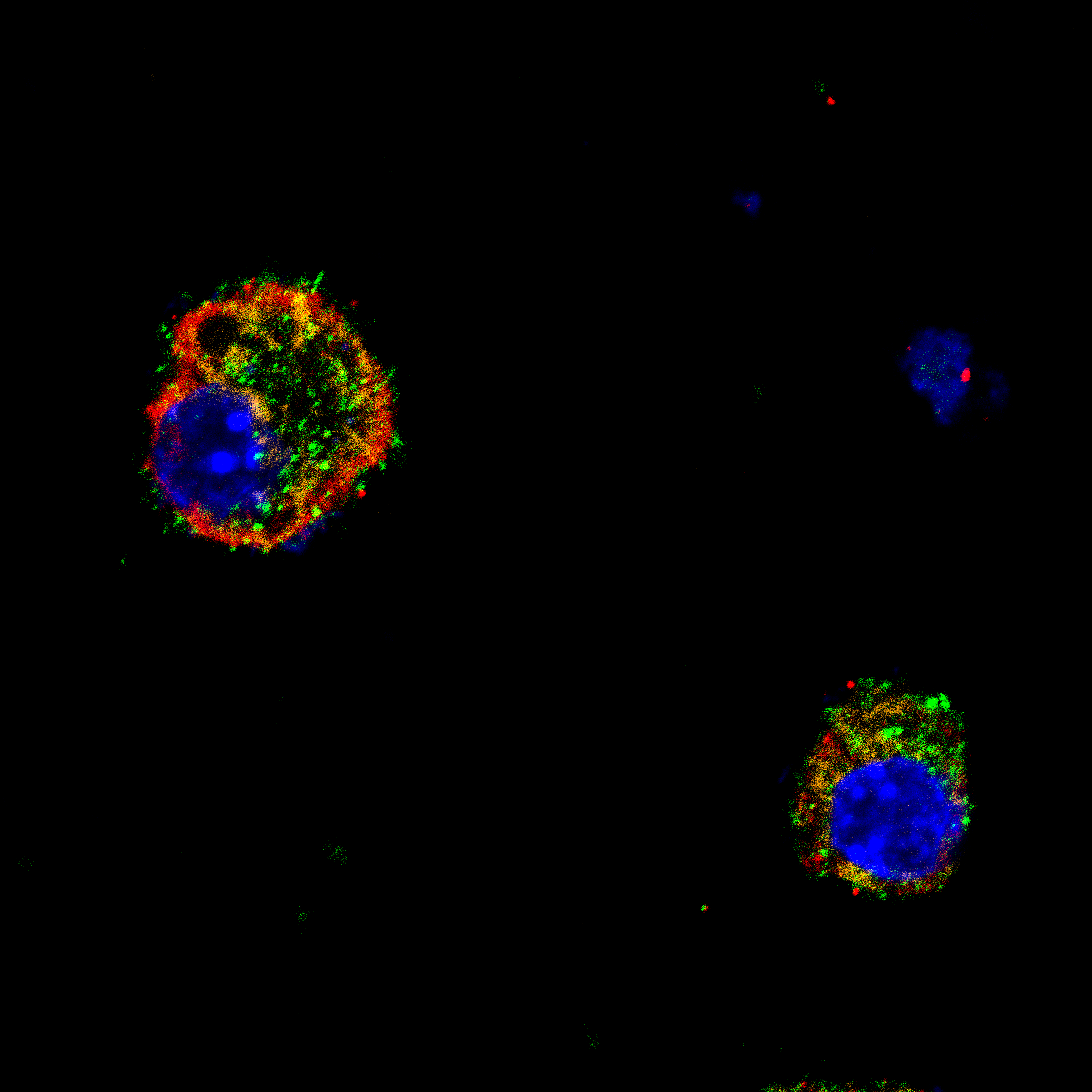

Supplement: S1 File — (ZIP) [file ppat.1012230.s002.zip › S1_File/Fig_3D/SeV/SeV-merge-4.tif]

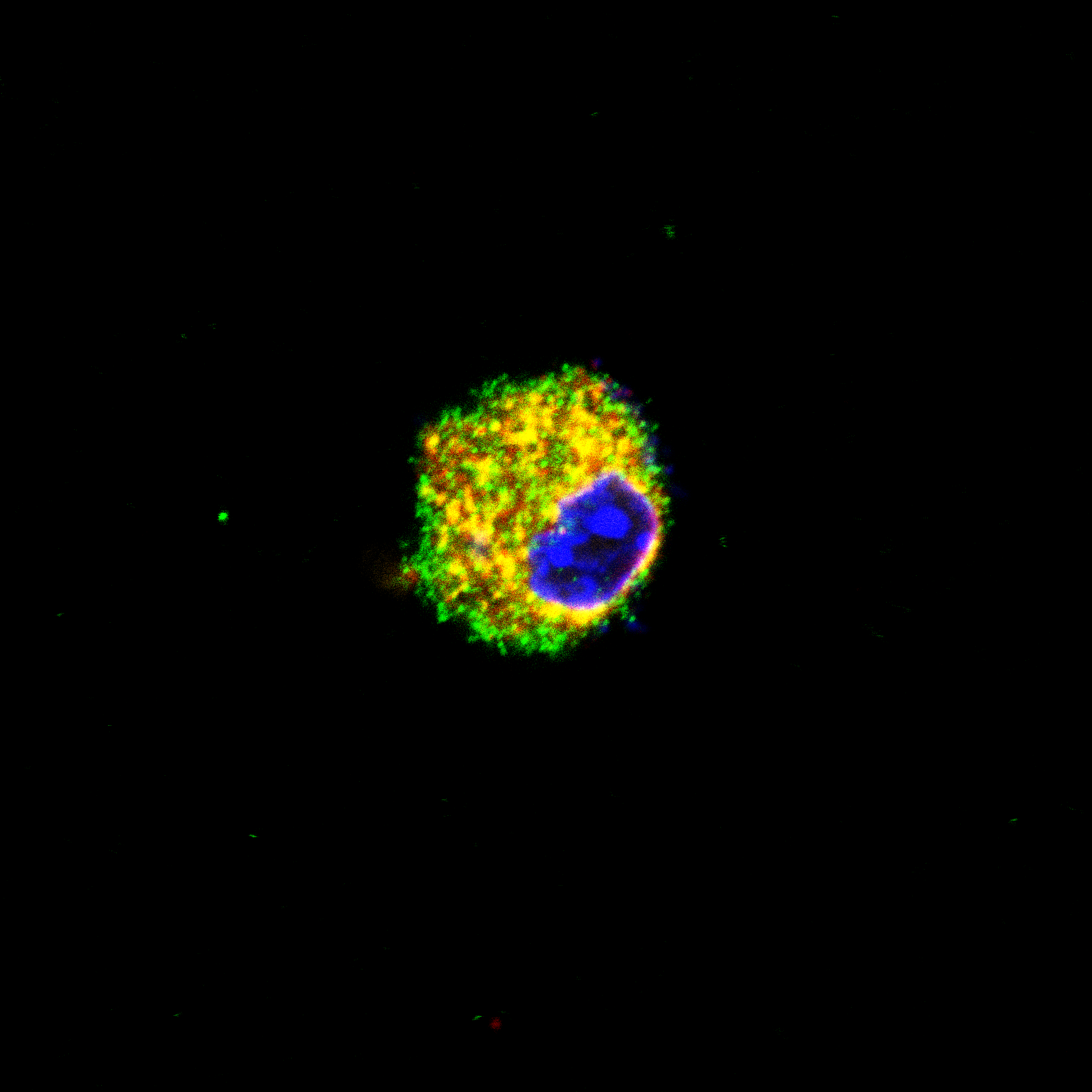

Supplement: S1 File — (ZIP) [file ppat.1012230.s002.zip › S1_File/Fig_3D/SeV/SeV-merge-5.tif]

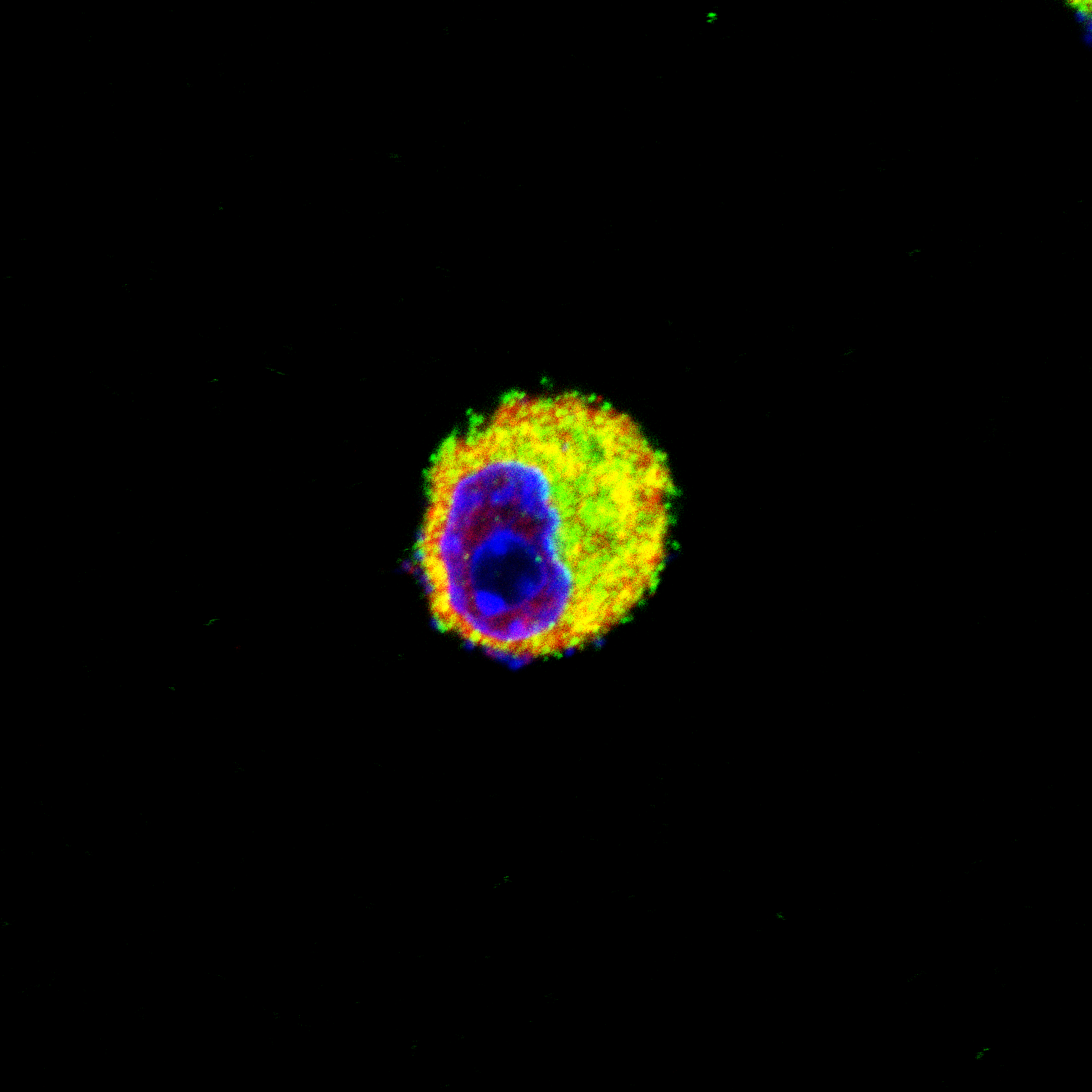

Supplement: S1 File — (ZIP) [file ppat.1012230.s002.zip › S1_File/Fig_3D/SeV/SeV-merge-6.tif]

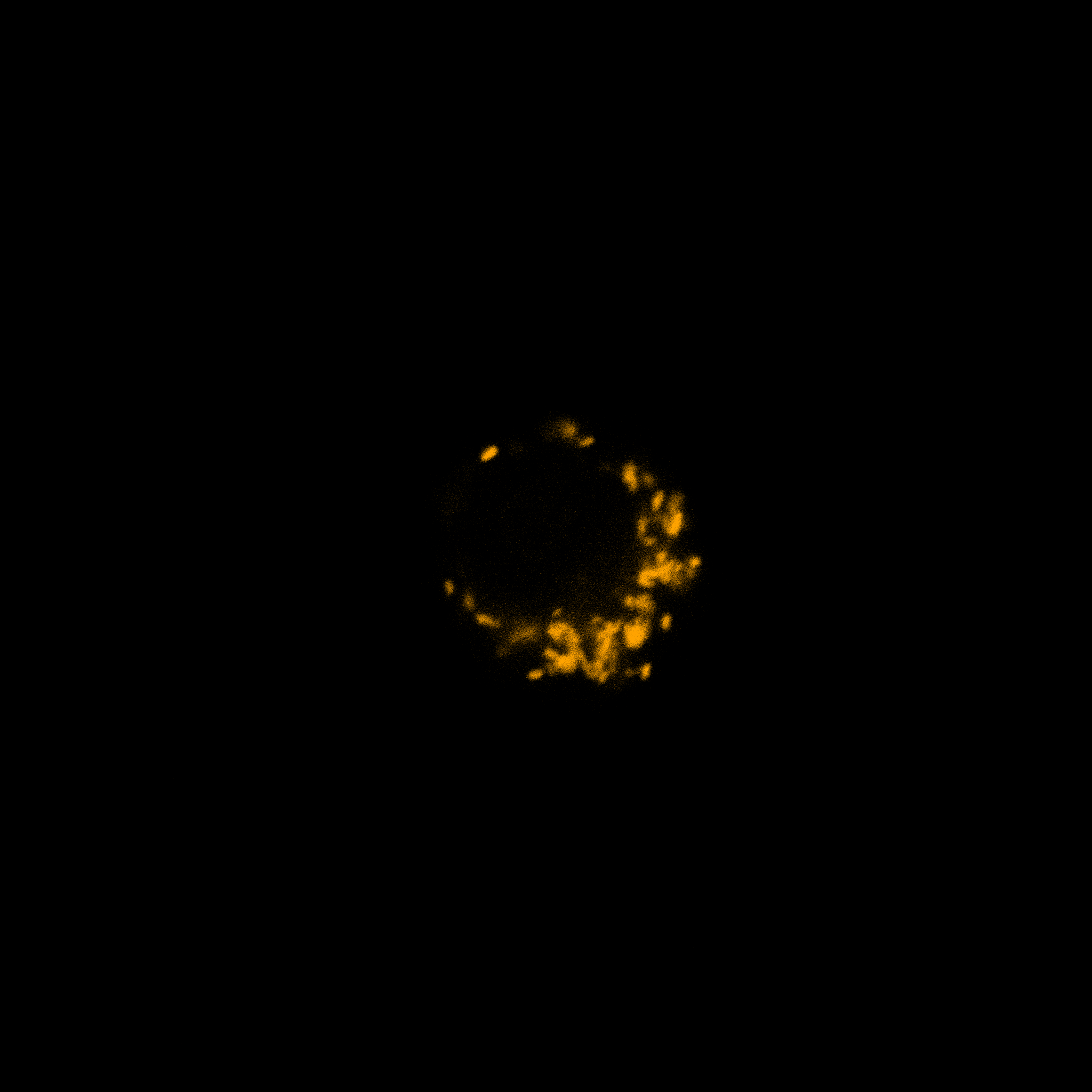

Supplement: S1 File — (ZIP) [file ppat.1012230.s002.zip › S1_File/Fig_3D/SeV/SeV-Mito-1.tif]

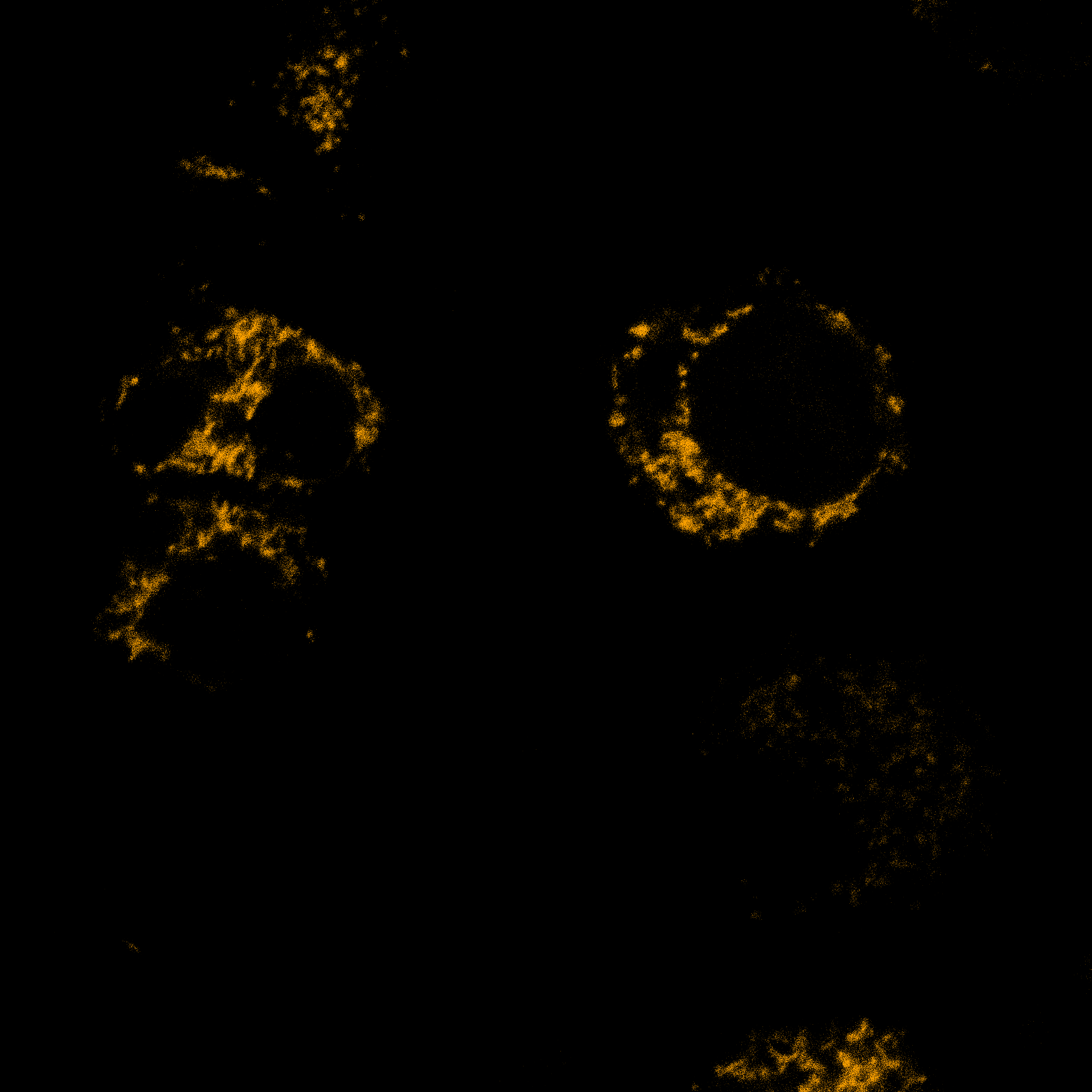

Supplement: S1 File — (ZIP) [file ppat.1012230.s002.zip › S1_File/Fig_3D/SeV/SeV-Mito-2.tif]

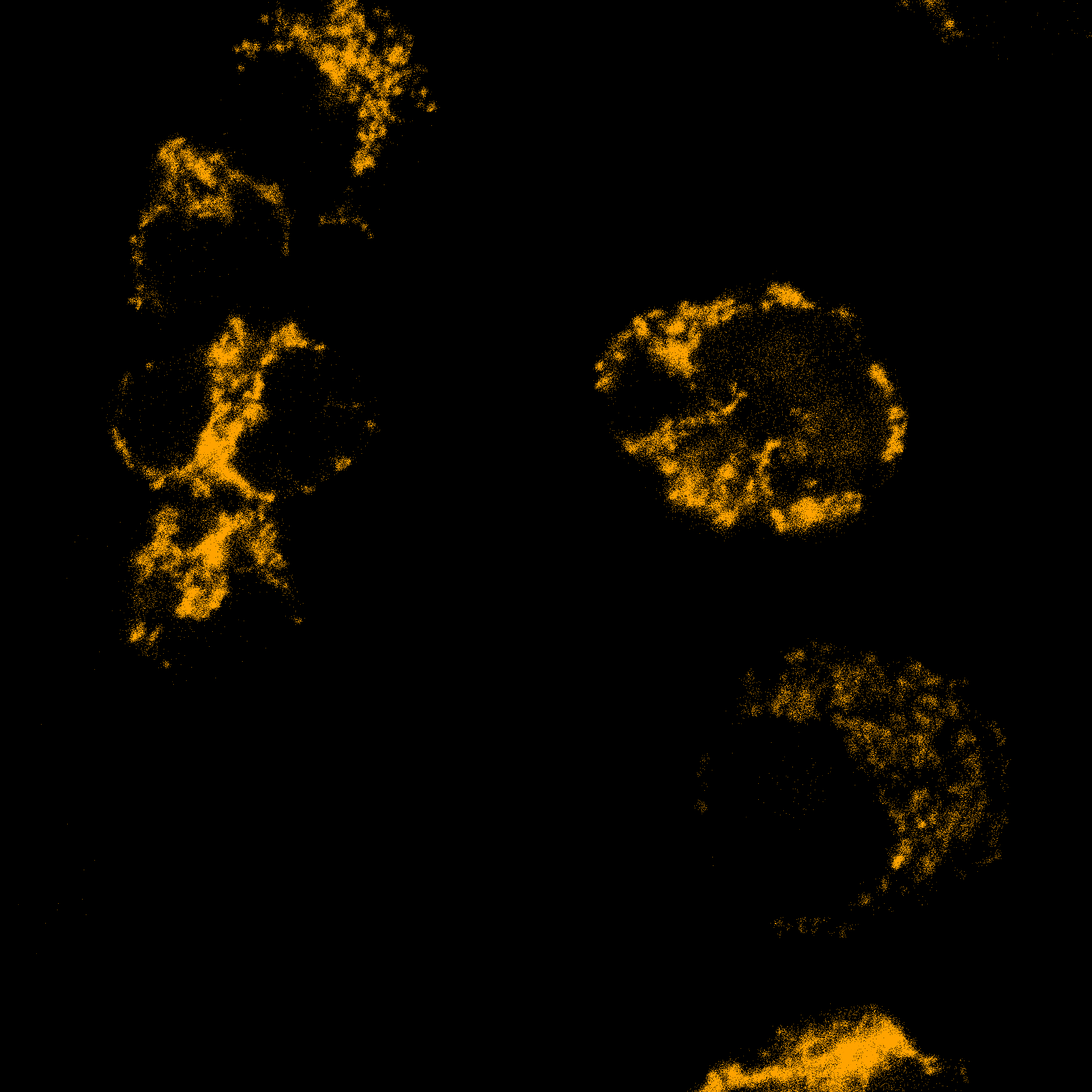

Supplement: S1 File — (ZIP) [file ppat.1012230.s002.zip › S1_File/Fig_3D/SeV/SeV-Mito-3.tif]

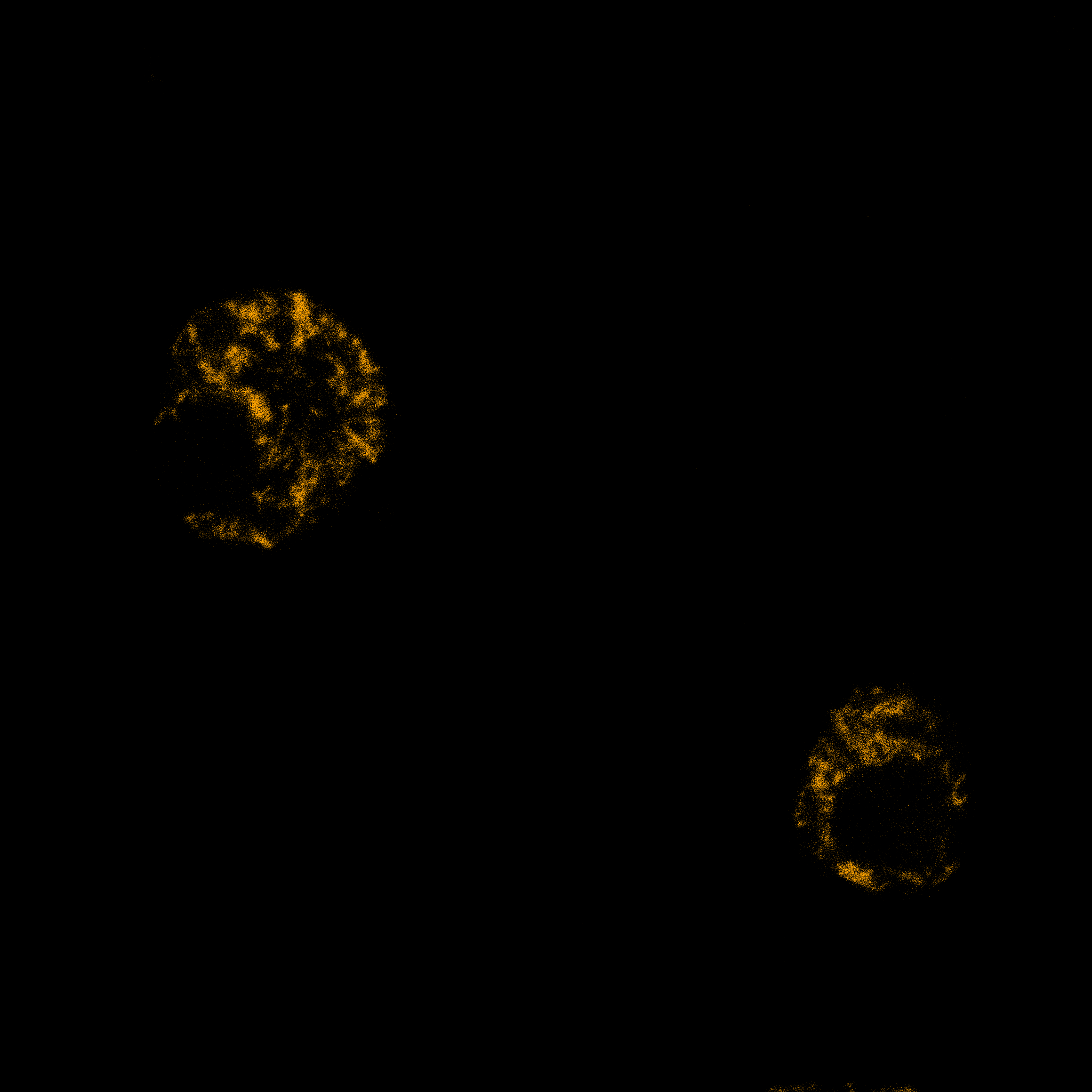

Supplement: S1 File — (ZIP) [file ppat.1012230.s002.zip › S1_File/Fig_3D/SeV/SeV-Mito-4.tif]

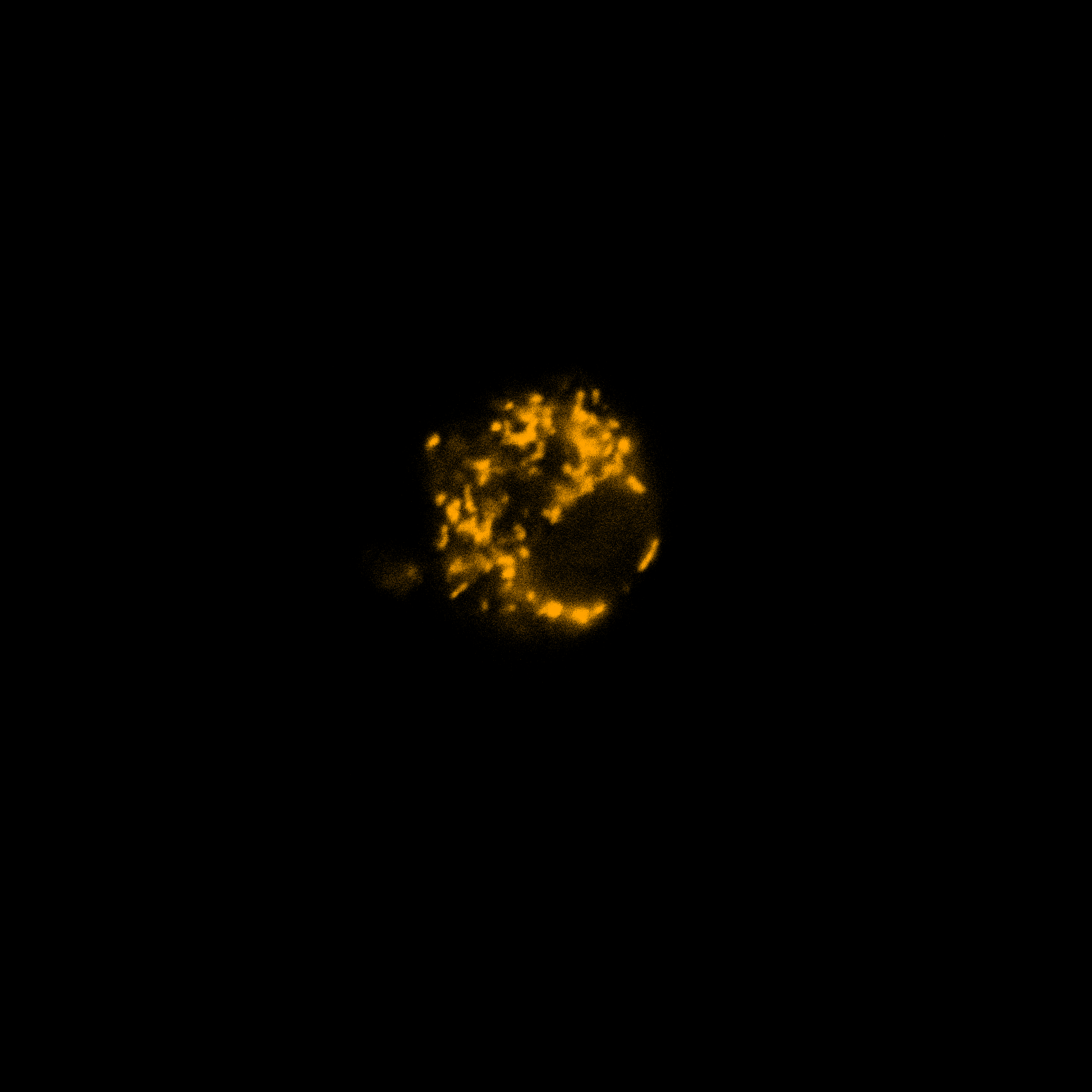

Supplement: S1 File — (ZIP) [file ppat.1012230.s002.zip › S1_File/Fig_3D/SeV/SeV-Mito-5.tif]

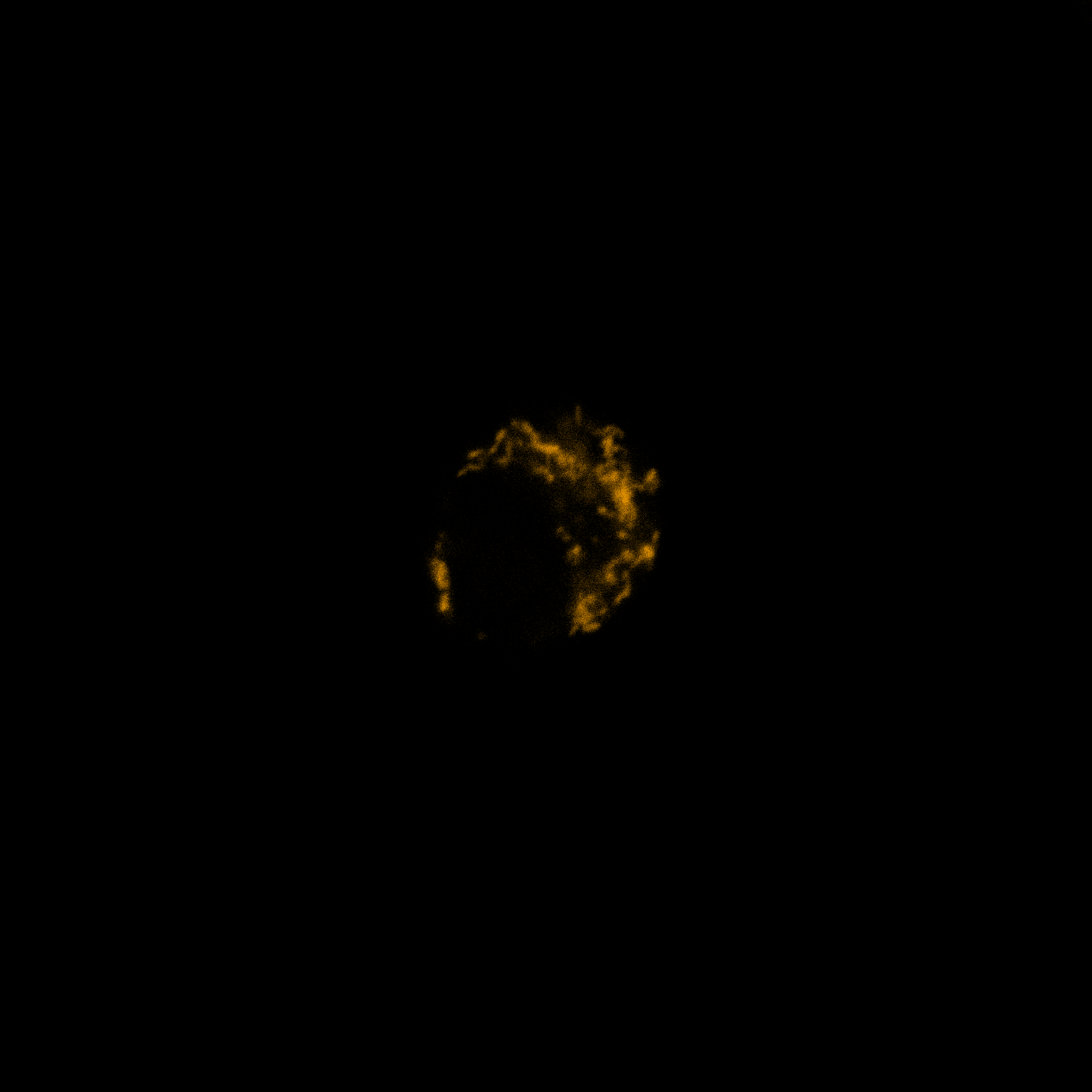

Supplement: S1 File — (ZIP) [file ppat.1012230.s002.zip › S1_File/Fig_3D/SeV/SeV-Mito-6.tif]

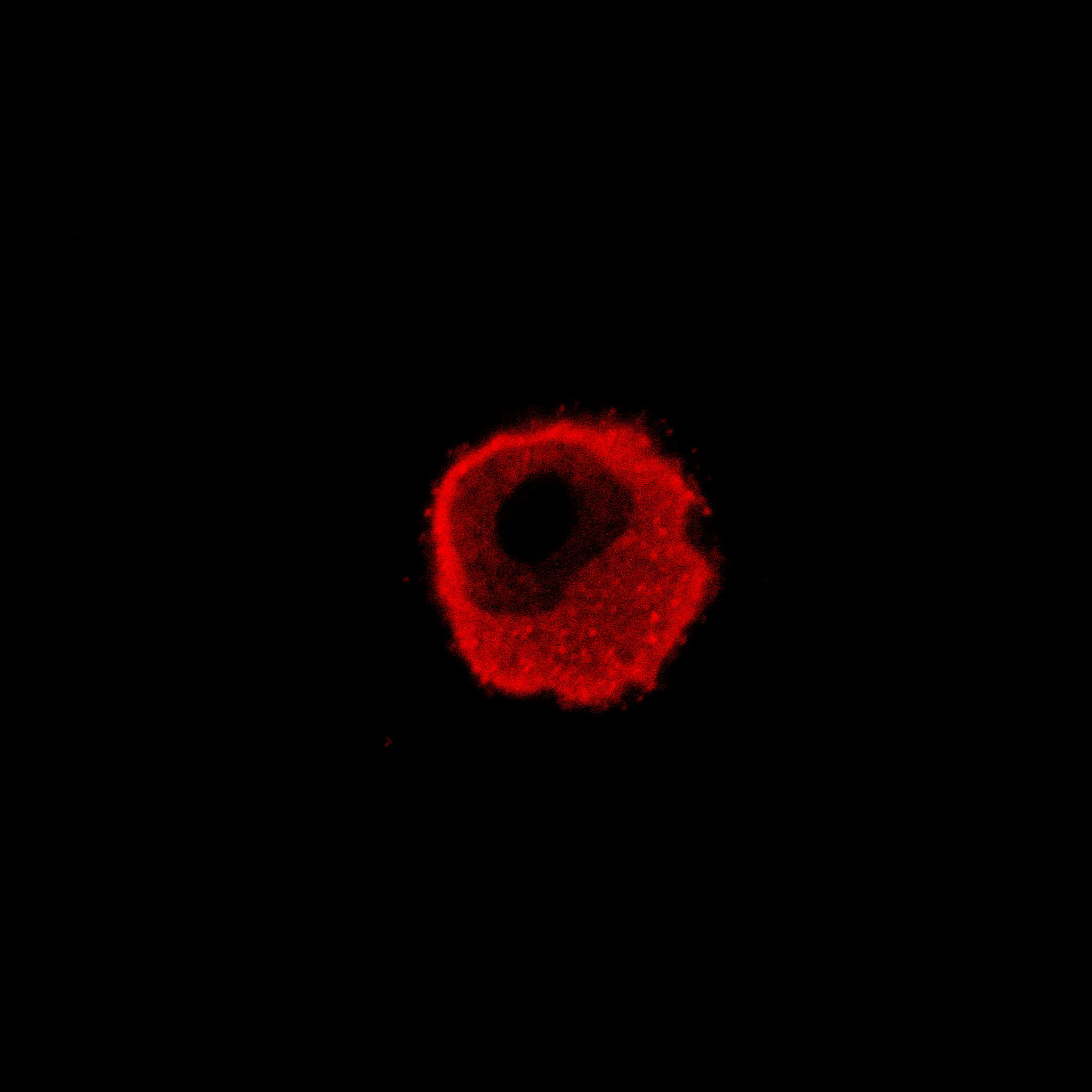

Supplement: S1 File — (ZIP) [file ppat.1012230.s002.zip › S1_File/Fig_3D/SeV/SeV-RIG-I-1.tif]

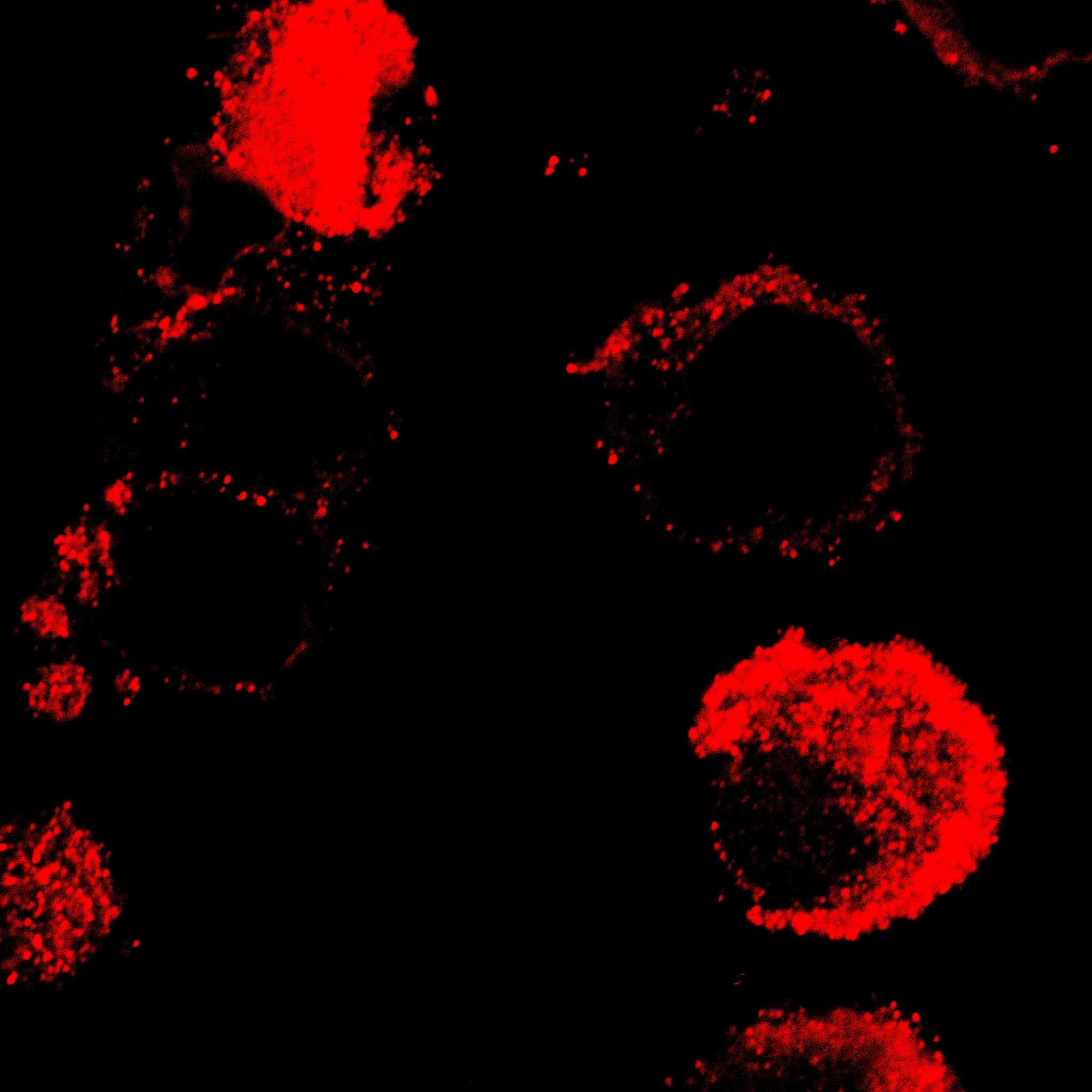

Supplement: S1 File — (ZIP) [file ppat.1012230.s002.zip › S1_File/Fig_3D/SeV/SeV-RIG-I-2.tif]

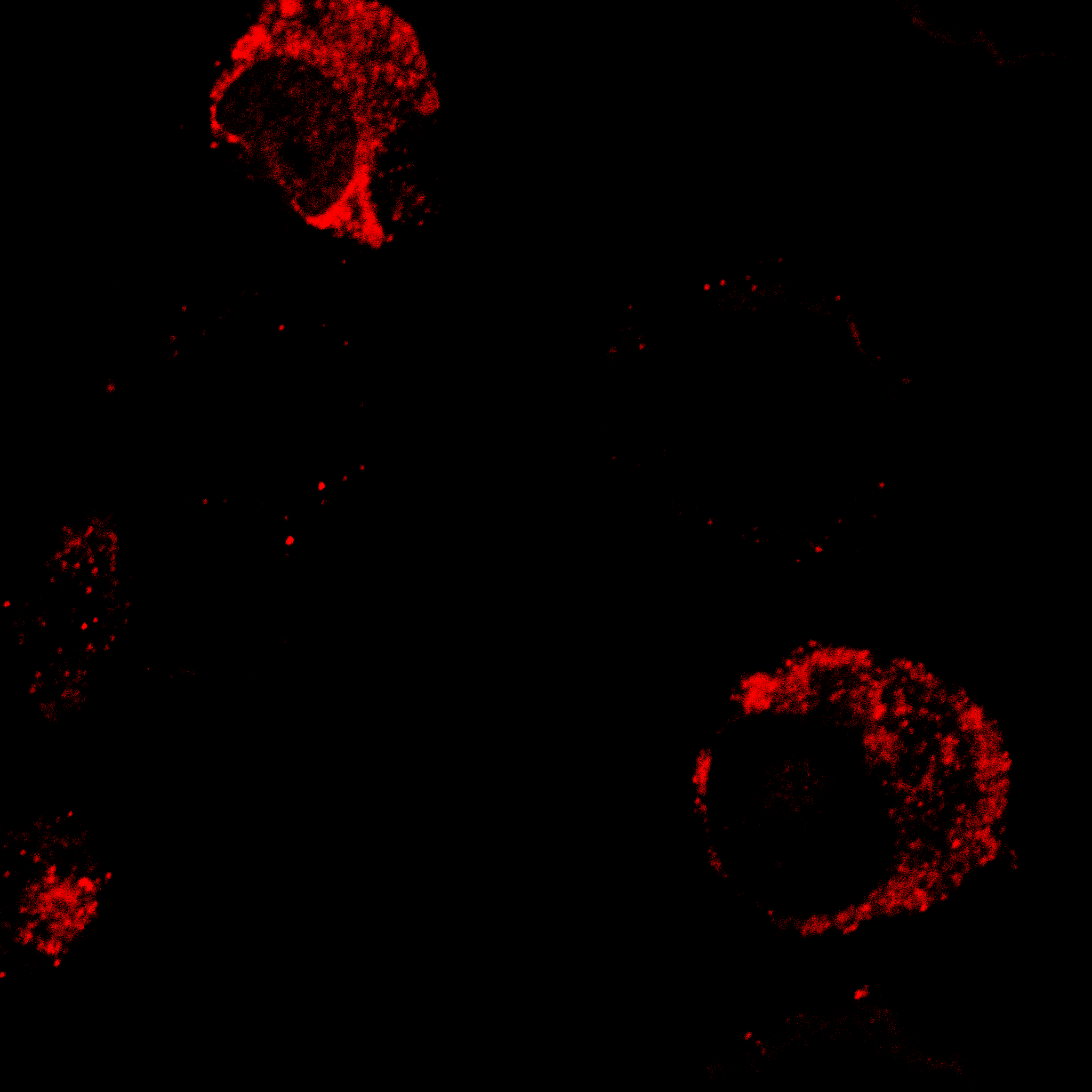

Supplement: S1 File — (ZIP) [file ppat.1012230.s002.zip › S1_File/Fig_3D/SeV/SeV-RIG-I-3.tif]

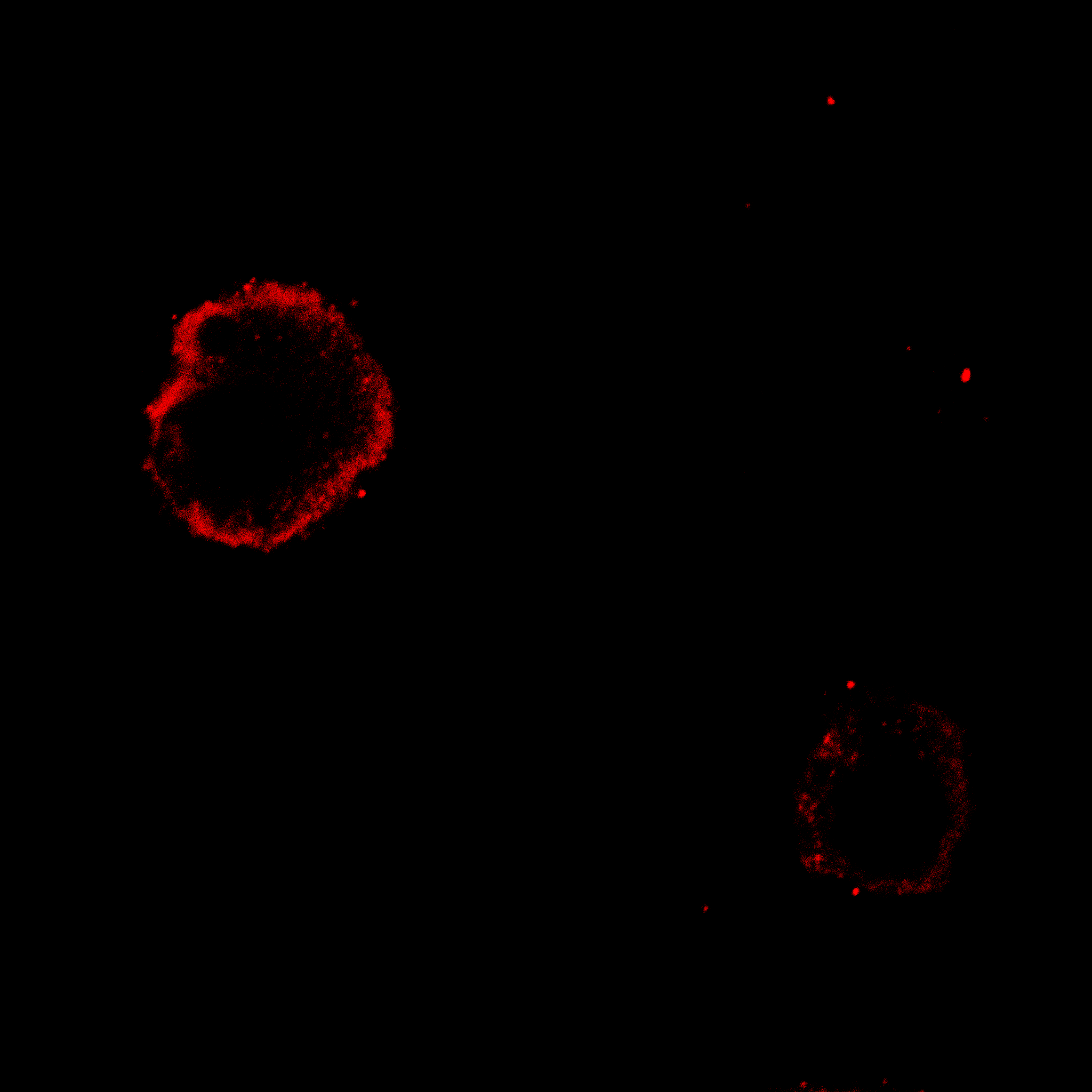

Supplement: S1 File — (ZIP) [file ppat.1012230.s002.zip › S1_File/Fig_3D/SeV/SeV-RIG-I-4.tif]

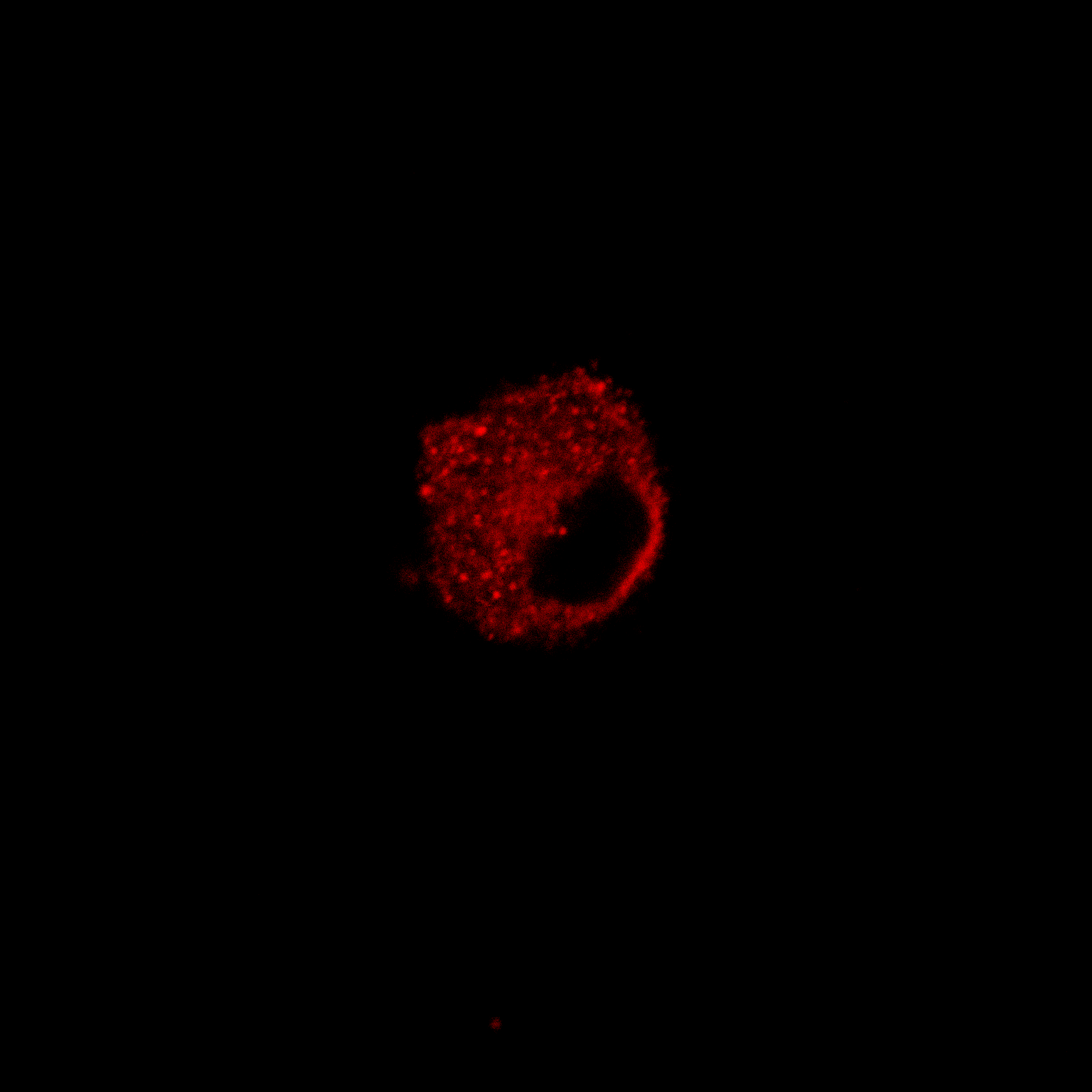

Supplement: S1 File — (ZIP) [file ppat.1012230.s002.zip › S1_File/Fig_3D/SeV/SeV-RIG-I-5.tif]

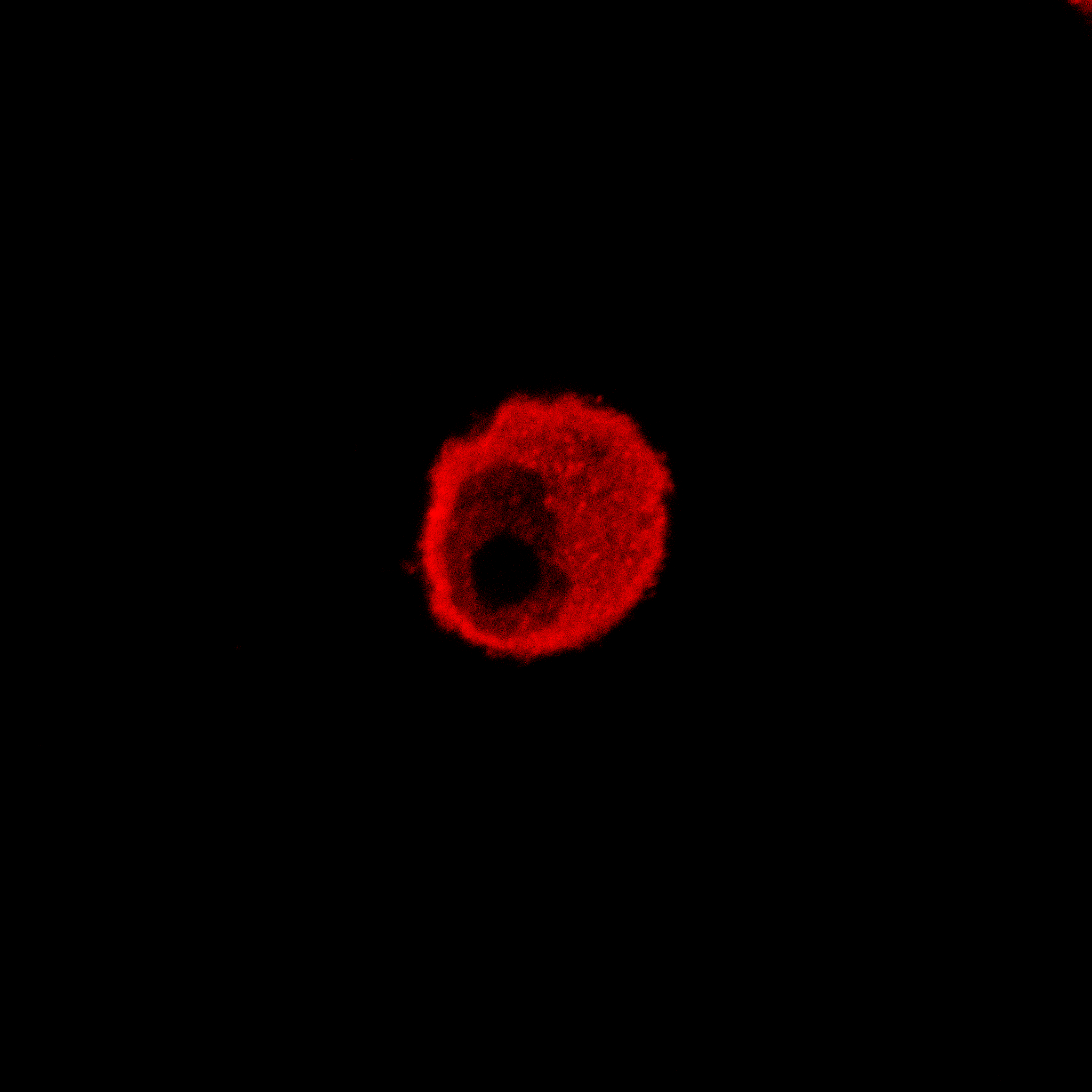

Supplement: S1 File — (ZIP) [file ppat.1012230.s002.zip › S1_File/Fig_3D/SeV/SeV-RIG-I-6.tif]

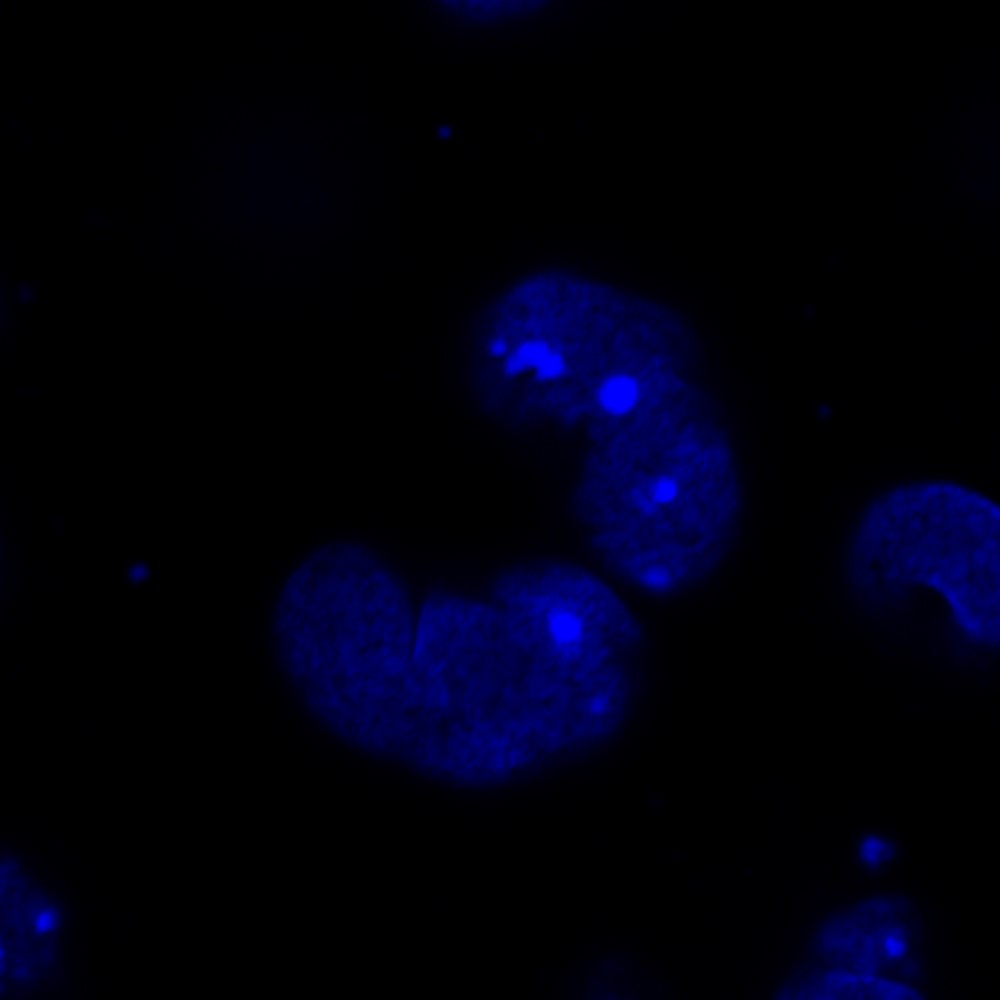

Supplement: S1 File — (ZIP) [file ppat.1012230.s002.zip › S1_File/Fig_6B/WT-IAV-DAPI.jpg]

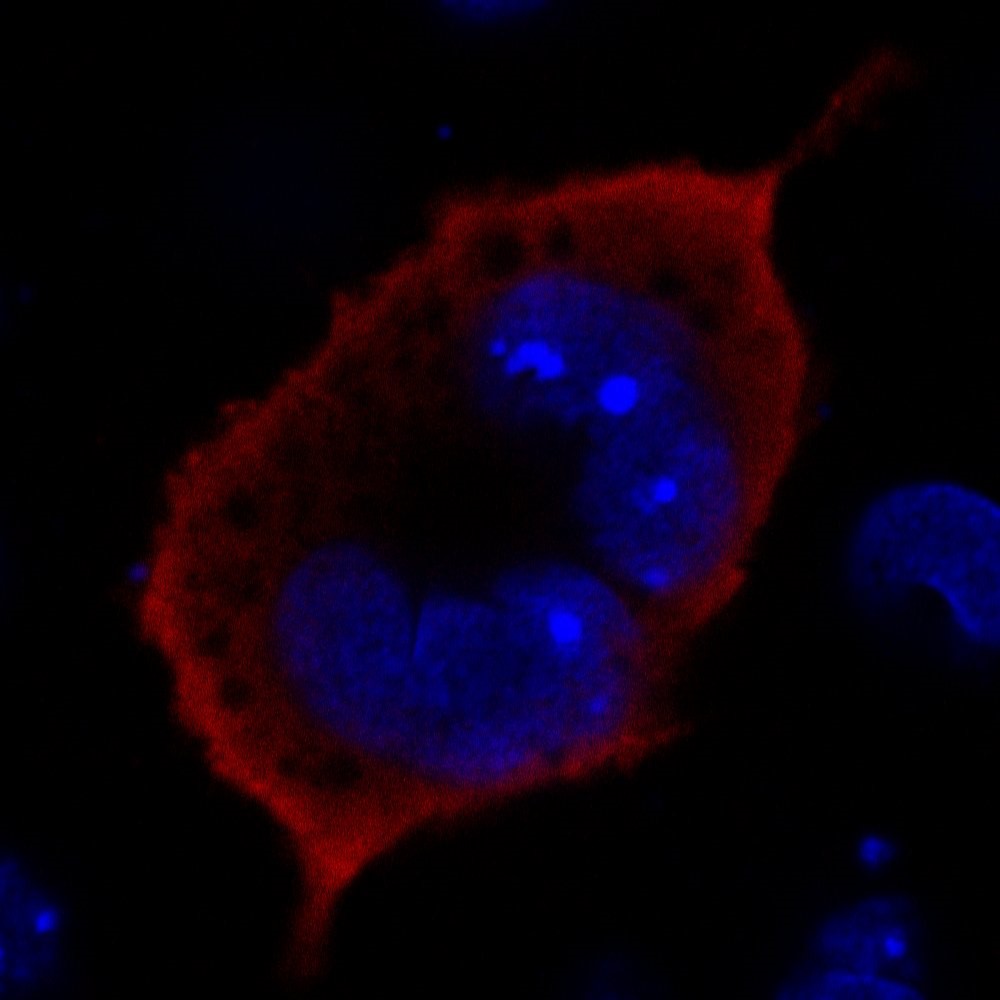

Supplement: S1 File — (ZIP) [file ppat.1012230.s002.zip › S1_File/Fig_6B/WT-IAV-merge.jpg]

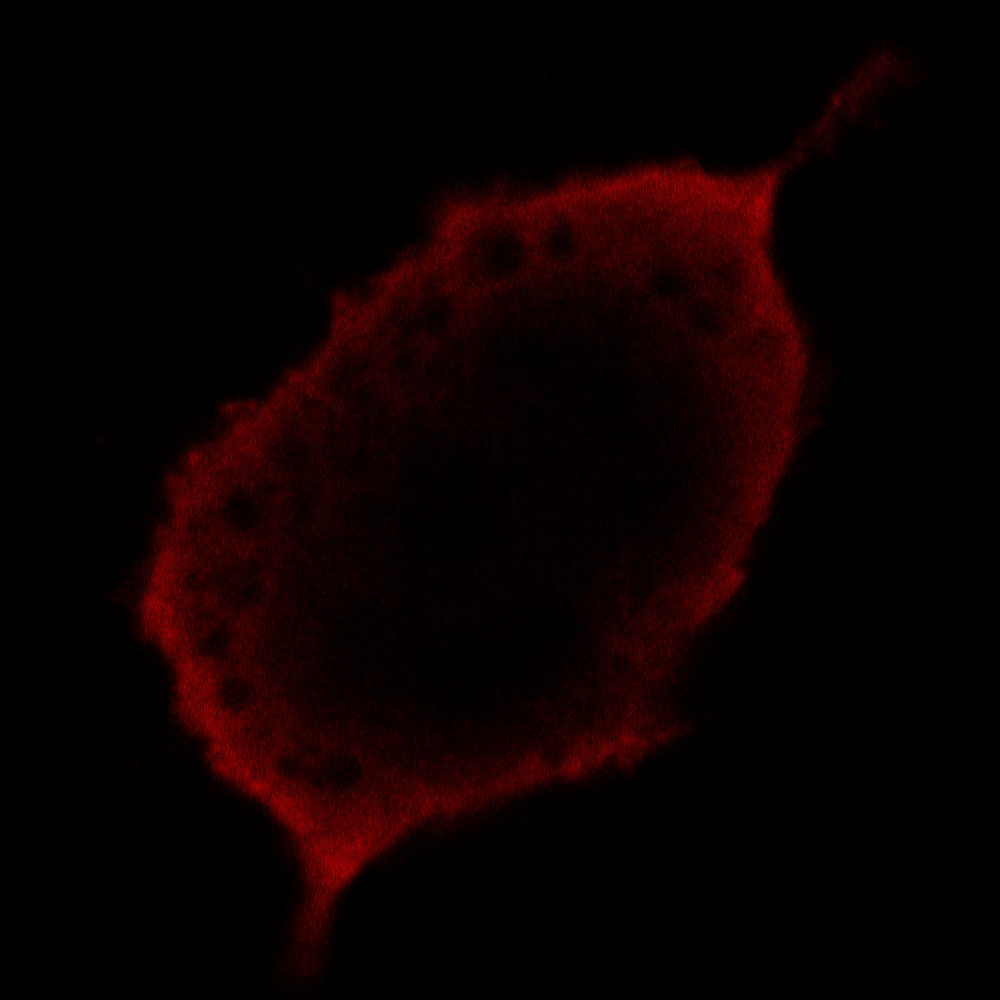

Supplement: S1 File — (ZIP) [file ppat.1012230.s002.zip › S1_File/Fig_6B/WT-IAV-NP.jpg]

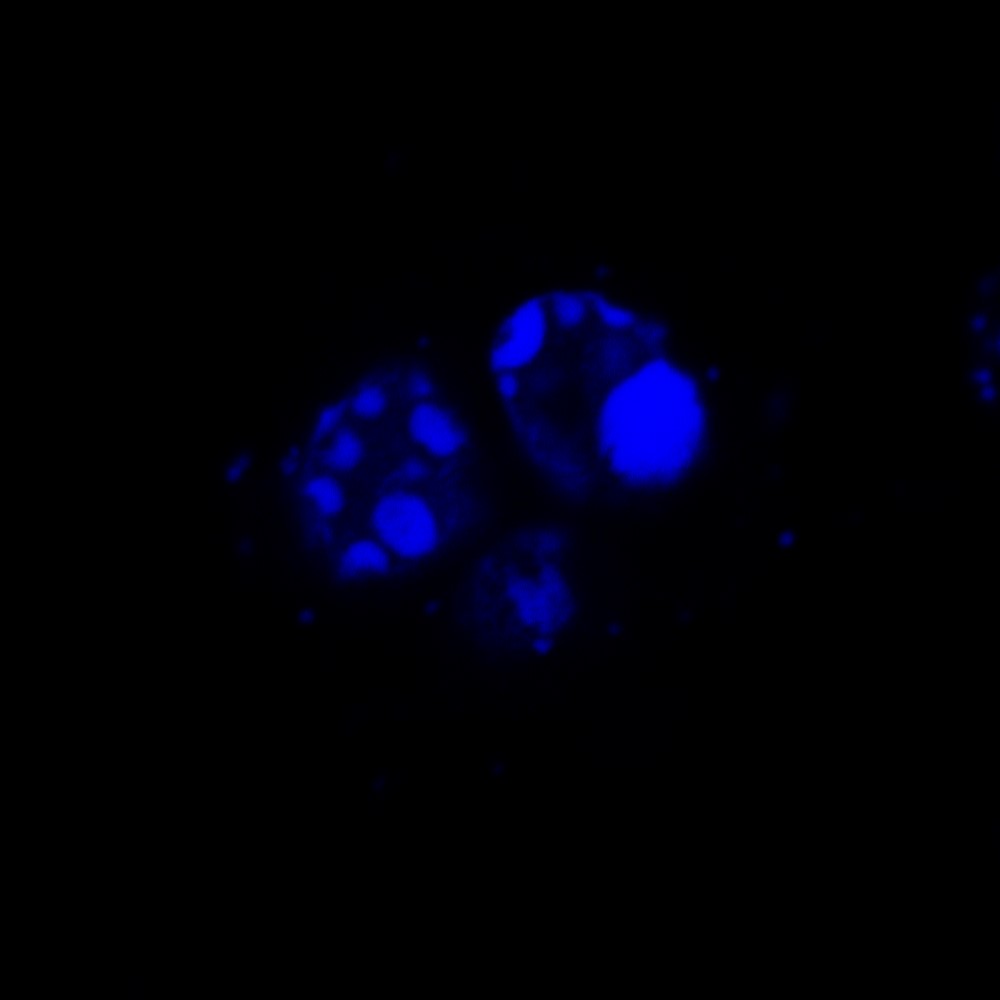

Supplement: S1 File — (ZIP) [file ppat.1012230.s002.zip › S1_File/Fig_6B/WT-Mock-DAPI.jpg]

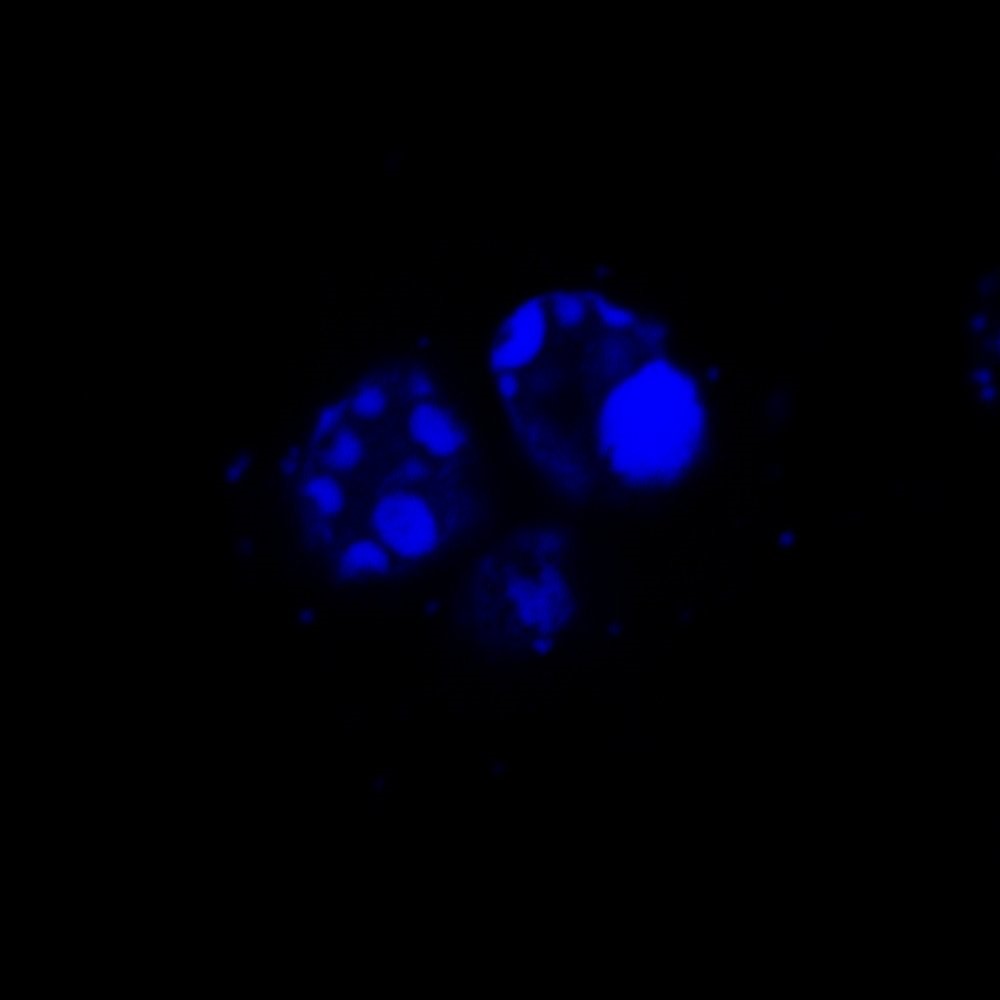

Supplement: S1 File — (ZIP) [file ppat.1012230.s002.zip › S1_File/Fig_6B/WT-Mock-merge.jpg]

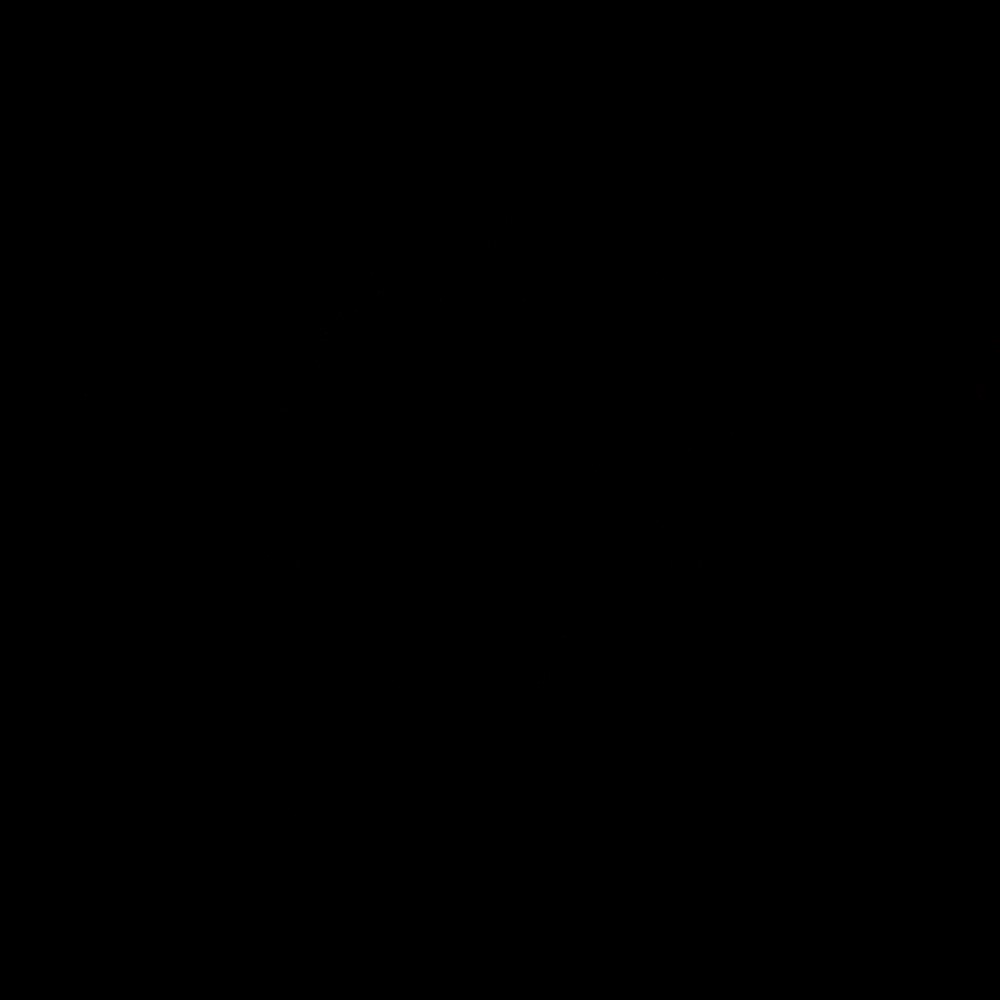

Supplement: S1 File — (ZIP) [file ppat.1012230.s002.zip › S1_File/Fig_6B/WT-Mock-NP.jpg]

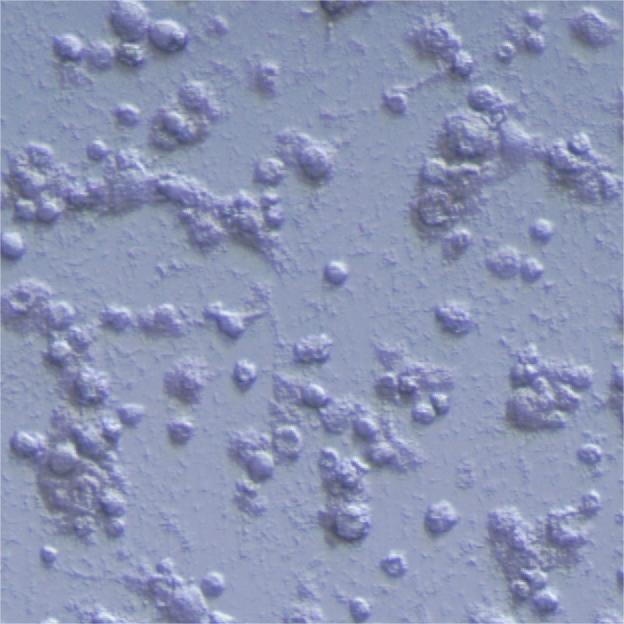

Supplement: S1 File — (ZIP) [file ppat.1012230.s002.zip › S1_File/Fig_6G/Adap KO-BF.jpg]

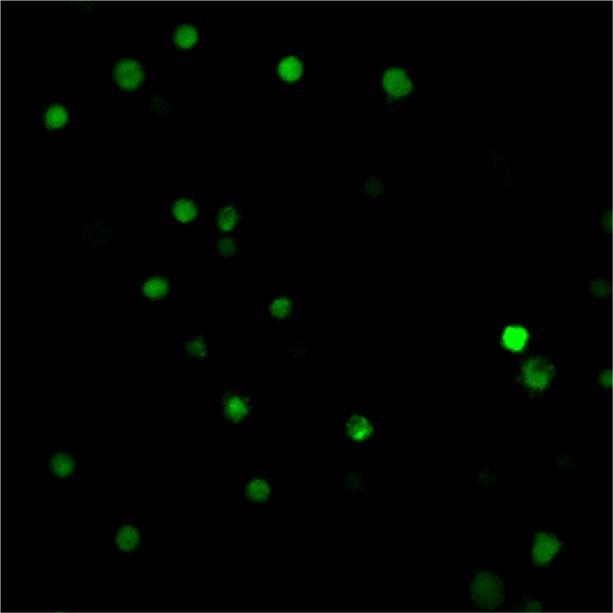

Supplement: S1 File — (ZIP) [file ppat.1012230.s002.zip › S1_File/Fig_6G/Adap KO-VSV-GFP.jpg]

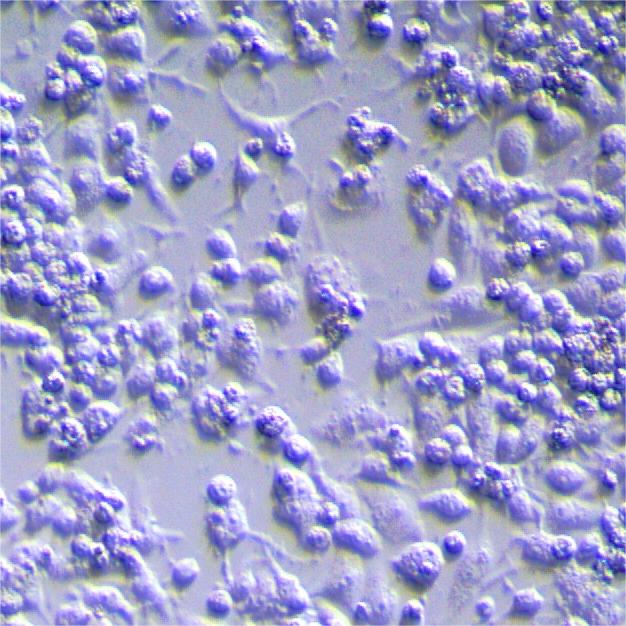

Supplement: S1 File — (ZIP) [file ppat.1012230.s002.zip › S1_File/Fig_6G/WT-BF.jpg]
