# Supplementary figures and images for: Dampening of ISGylation of RIG-I by ADAP regulates type I interferon response of macrophages to RNA virus infection (part 2 of 2)
Source: PLoS Pathog. 2024 May 22;20(5):e1012230. doi: 10.1371/journal.ppat.1012230 (PMC11111093; doi:10.1371/journal.ppat.1012230)

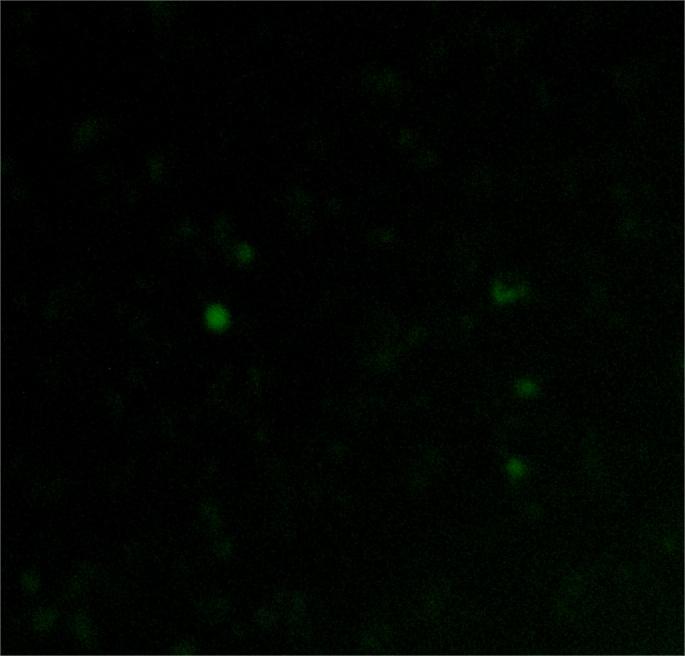

Supplement: S1 File — (ZIP) [file ppat.1012230.s002.zip › S1_File/Fig_6G/WT-VSV-GFP.jpg]

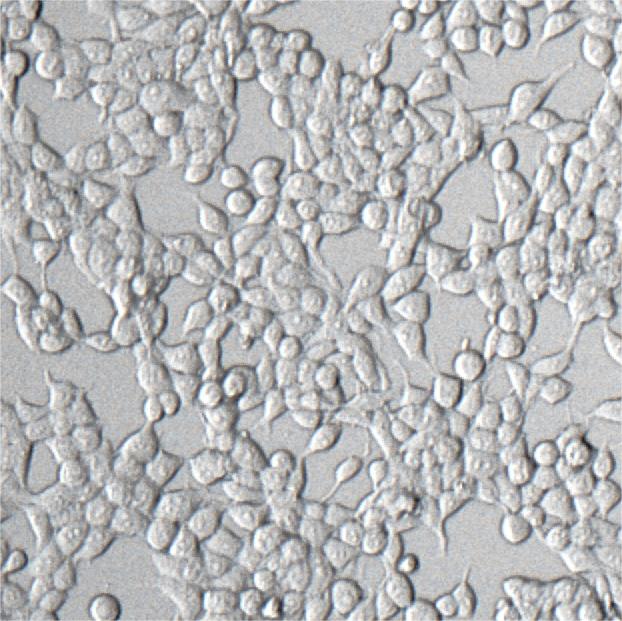

Supplement: S1 File — (ZIP) [file ppat.1012230.s002.zip › S1_File/Fig_6H/HA-ADAP-BF.jpg]

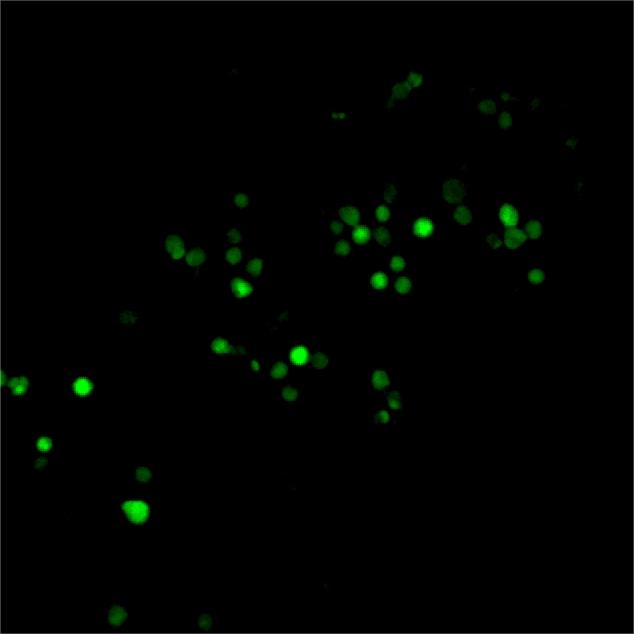

Supplement: S1 File — (ZIP) [file ppat.1012230.s002.zip › S1_File/Fig_6H/HA-ADAP-VSV-GFP.jpg]

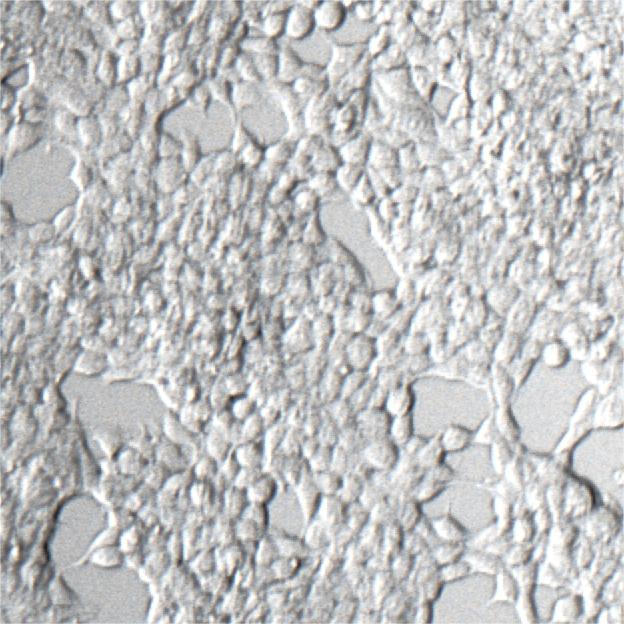

Supplement: S1 File — (ZIP) [file ppat.1012230.s002.zip › S1_File/Fig_6H/Vector-BF.jpg]

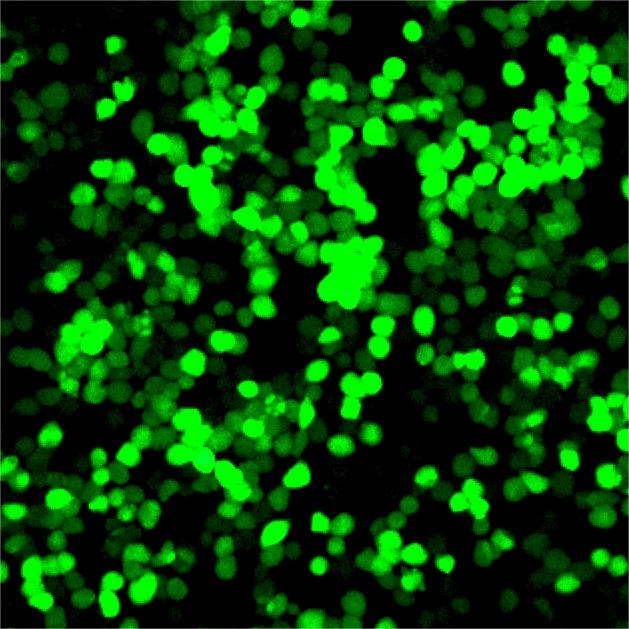

Supplement: S1 File — (ZIP) [file ppat.1012230.s002.zip › S1_File/Fig_6H/Vector-VSV-GFP.jpg]

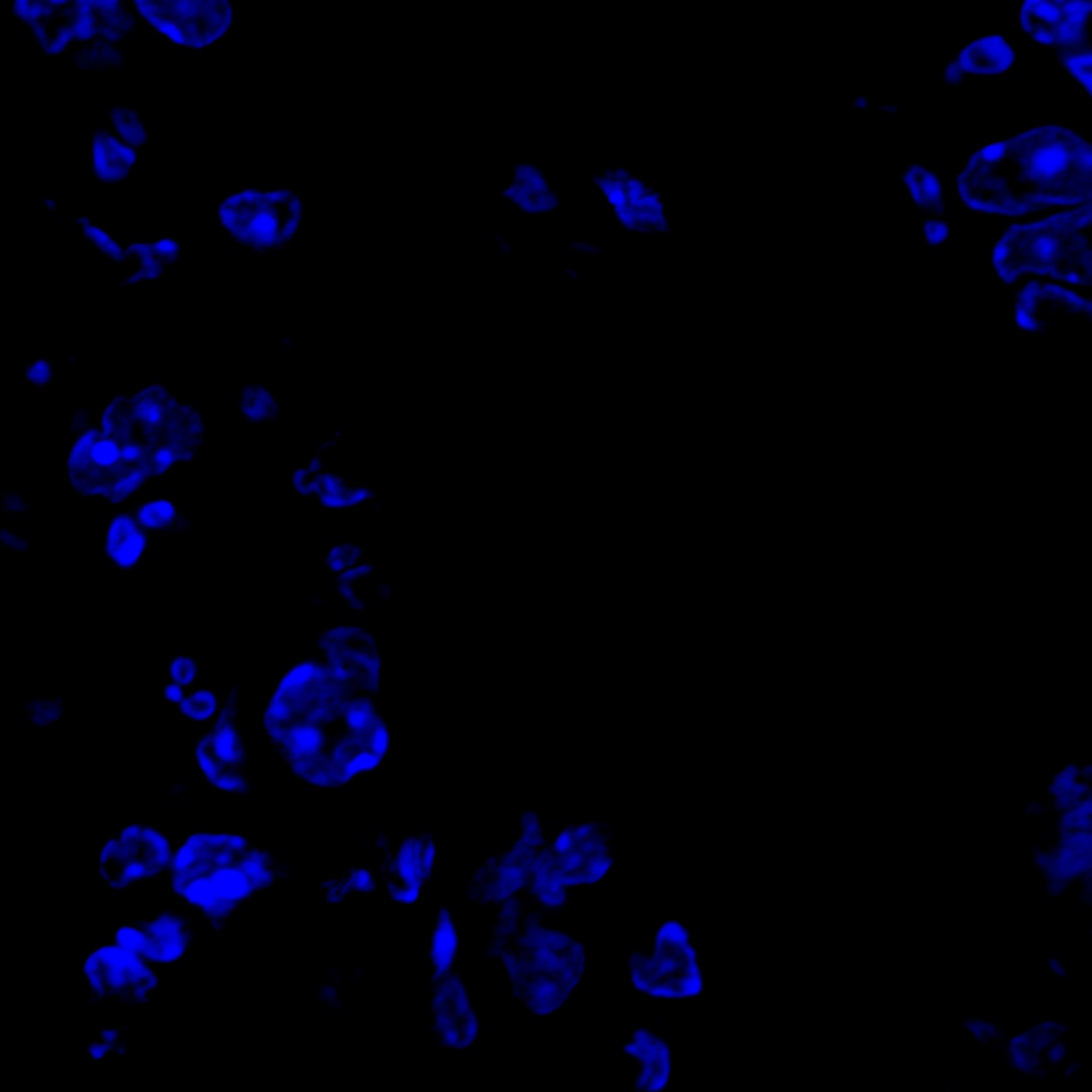

Supplement: S1 File — (ZIP) [file ppat.1012230.s002.zip › S1_File/Fig_7A/Lung/Adap KO-IAV-DAPI.jpg]

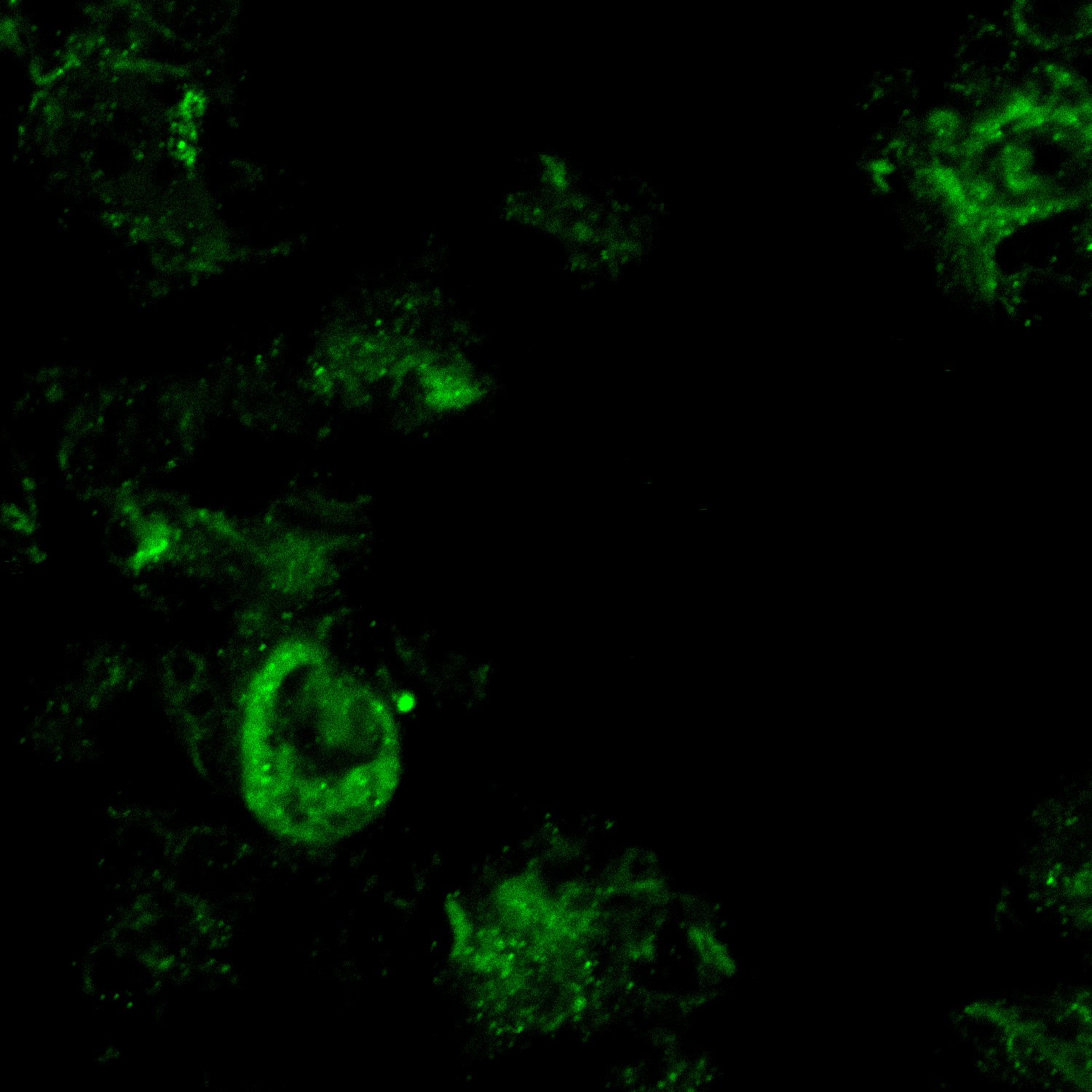

Supplement: S1 File — (ZIP) [file ppat.1012230.s002.zip › S1_File/Fig_7A/Lung/Adap KO-IAV-F480.jpg]

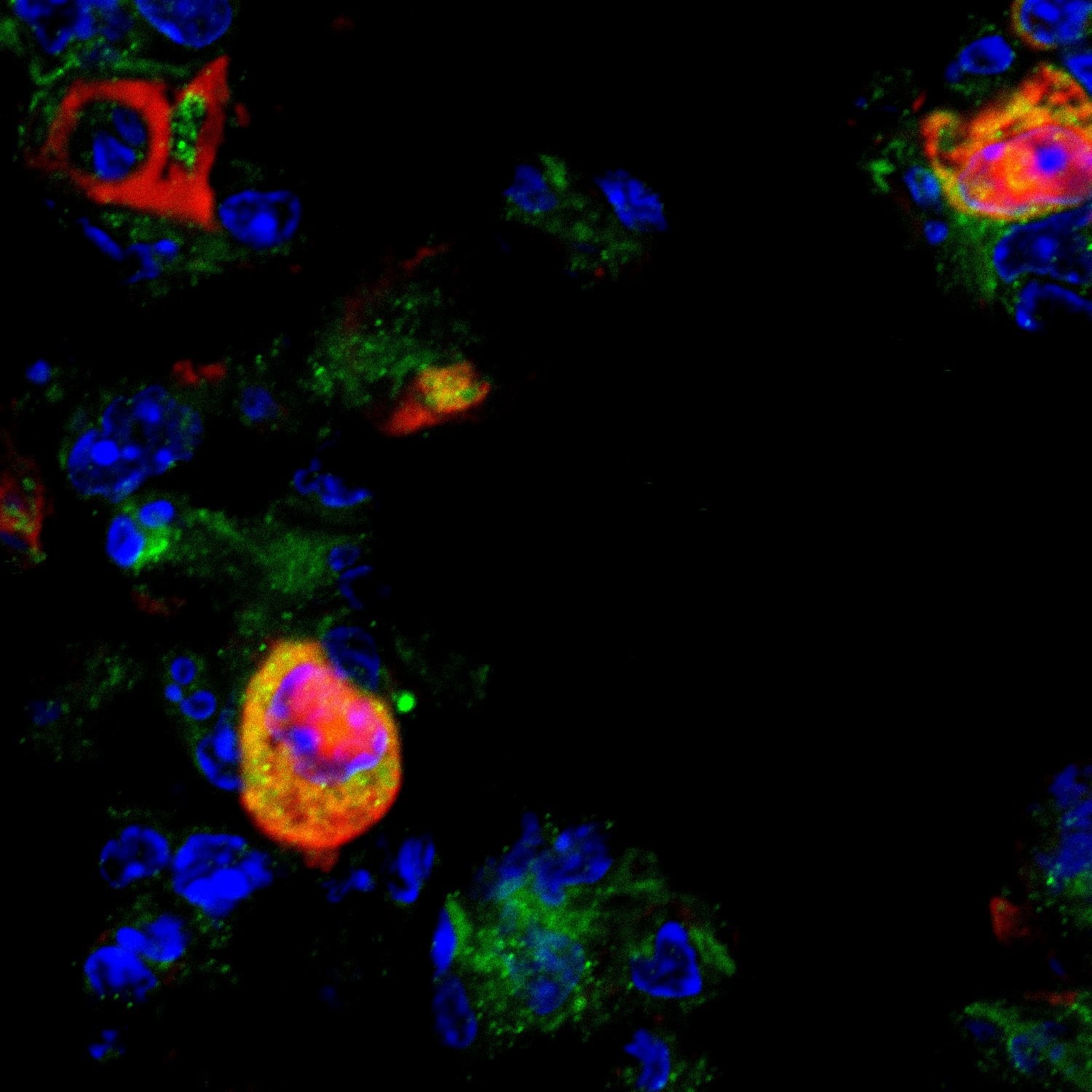

Supplement: S1 File — (ZIP) [file ppat.1012230.s002.zip › S1_File/Fig_7A/Lung/Adap KO-IAV-Merge.jpg]

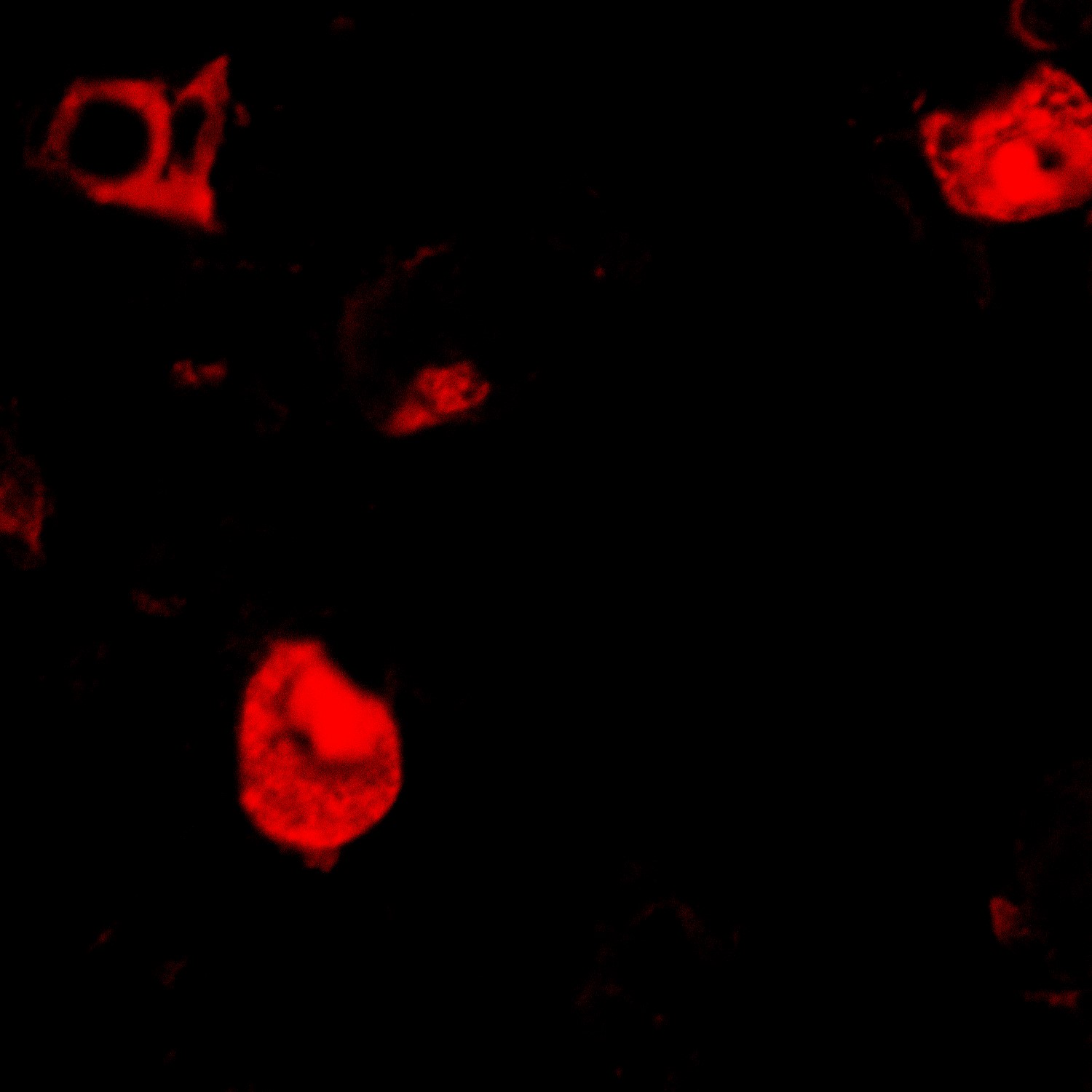

Supplement: S1 File — (ZIP) [file ppat.1012230.s002.zip › S1_File/Fig_7A/Lung/Adap KO-IAV-NP.jpg]

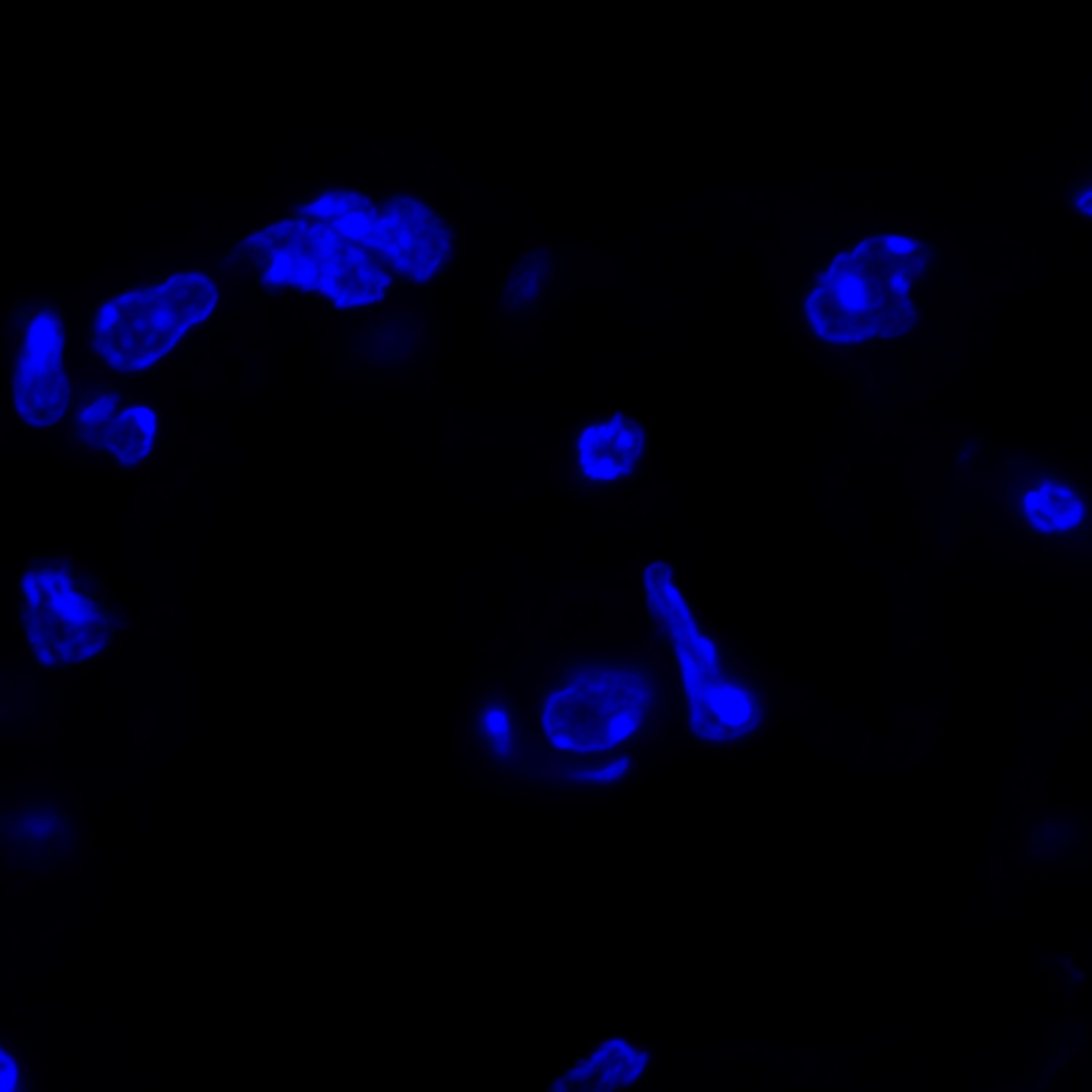

Supplement: S1 File — (ZIP) [file ppat.1012230.s002.zip › S1_File/Fig_7A/Lung/Adap KO-Mock-DAPI.jpg]

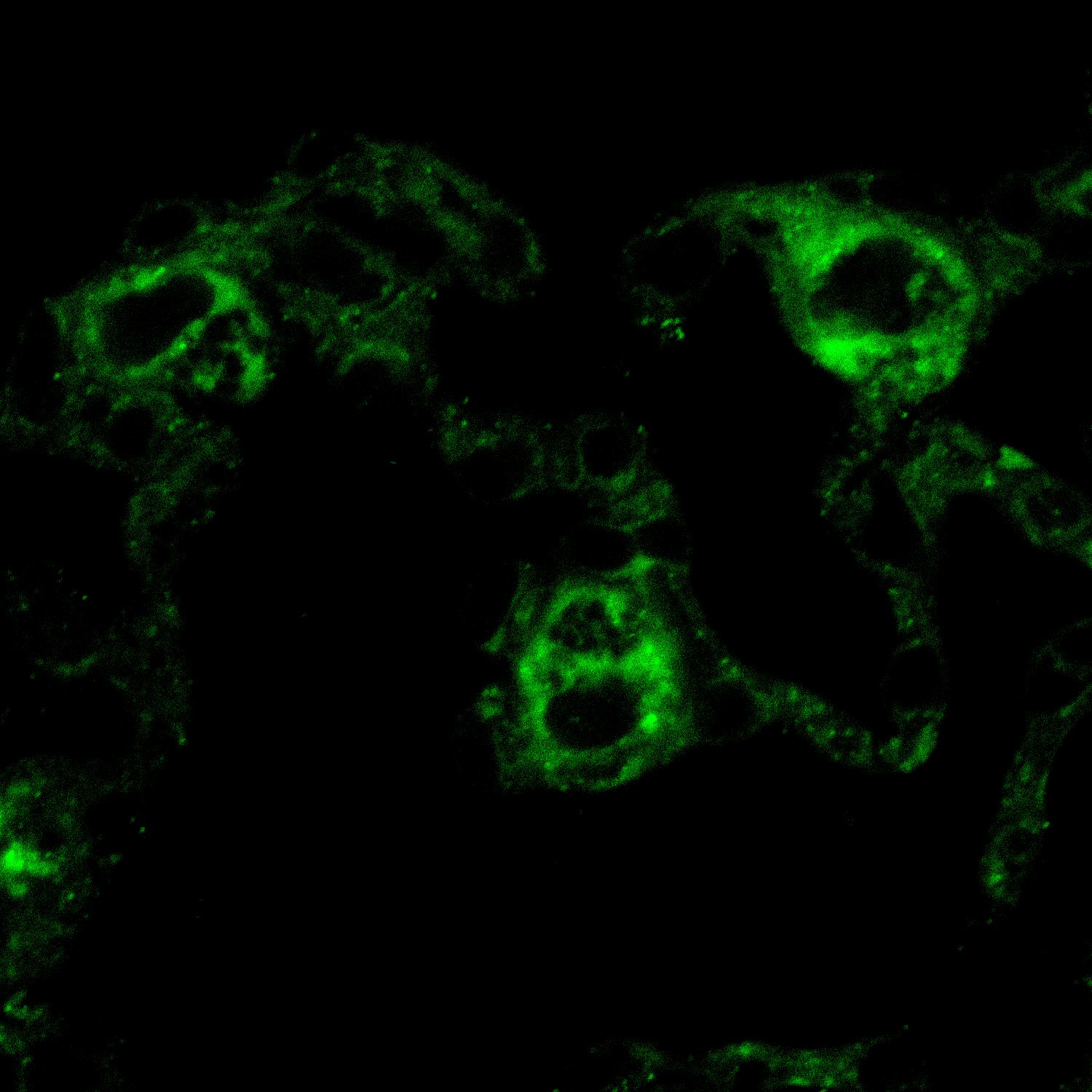

Supplement: S1 File — (ZIP) [file ppat.1012230.s002.zip › S1_File/Fig_7A/Lung/Adap KO-Mock-F480.jpg]

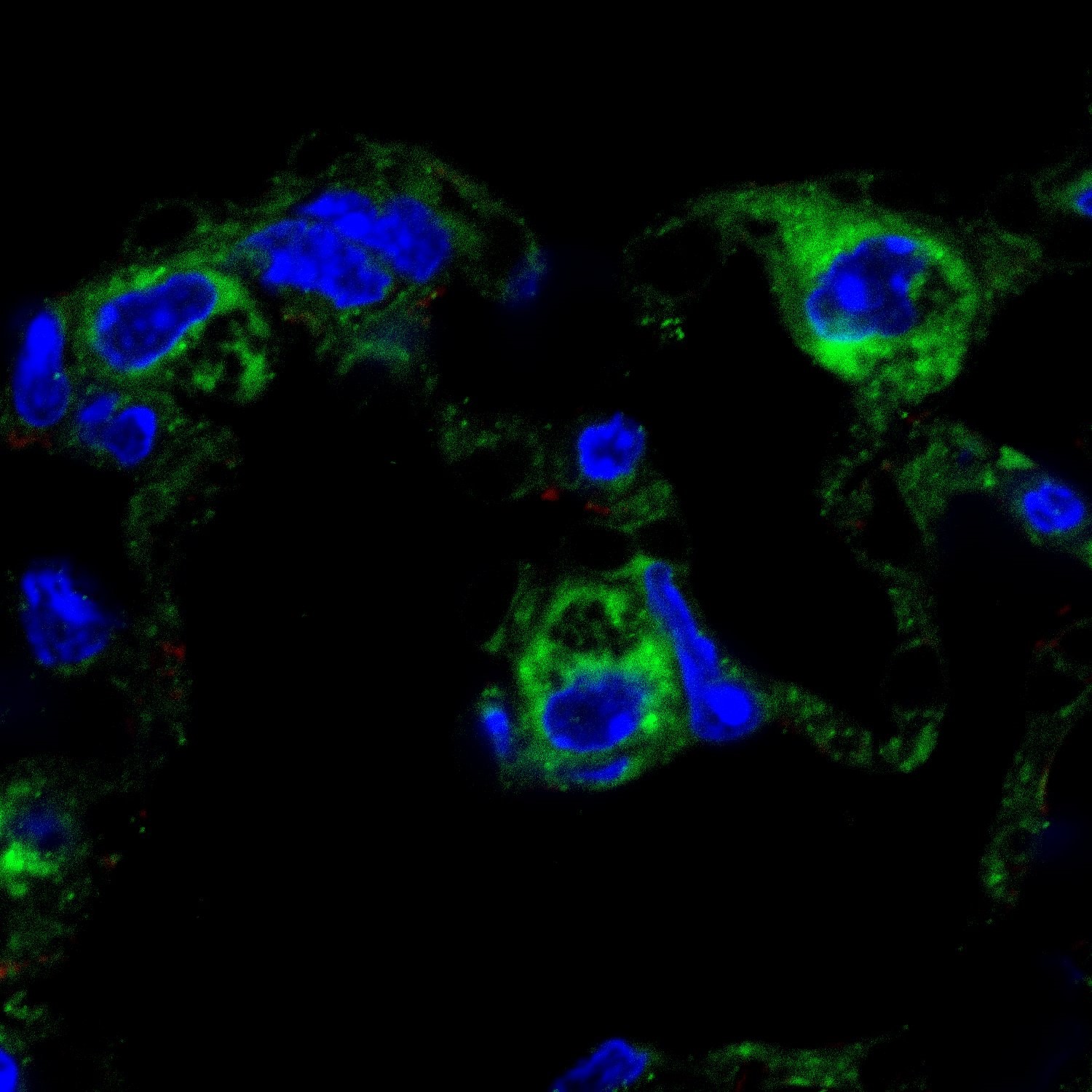

Supplement: S1 File — (ZIP) [file ppat.1012230.s002.zip › S1_File/Fig_7A/Lung/Adap KO-Mock-Merge.jpg]

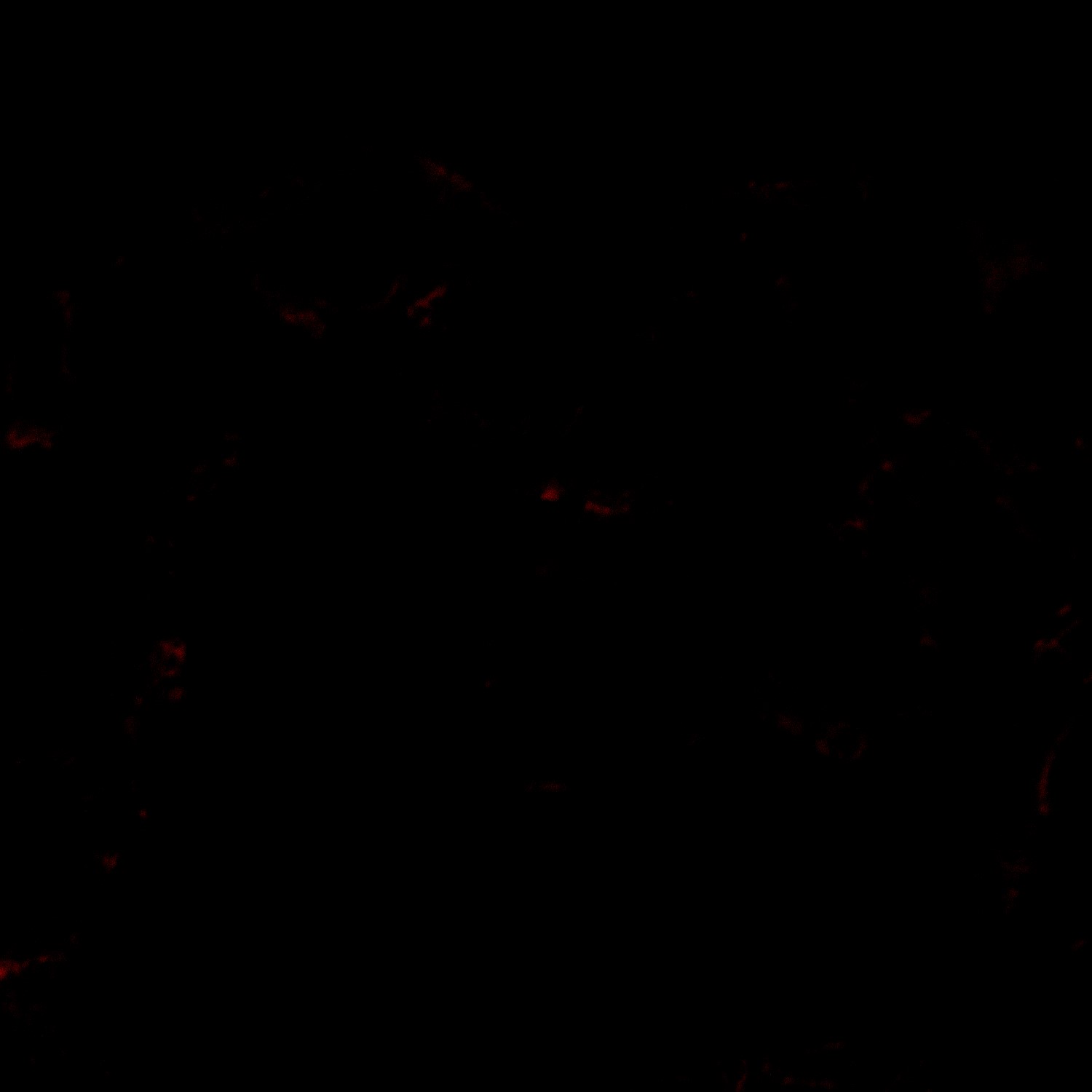

Supplement: S1 File — (ZIP) [file ppat.1012230.s002.zip › S1_File/Fig_7A/Lung/Adap KO-Mock-NP.jpg]

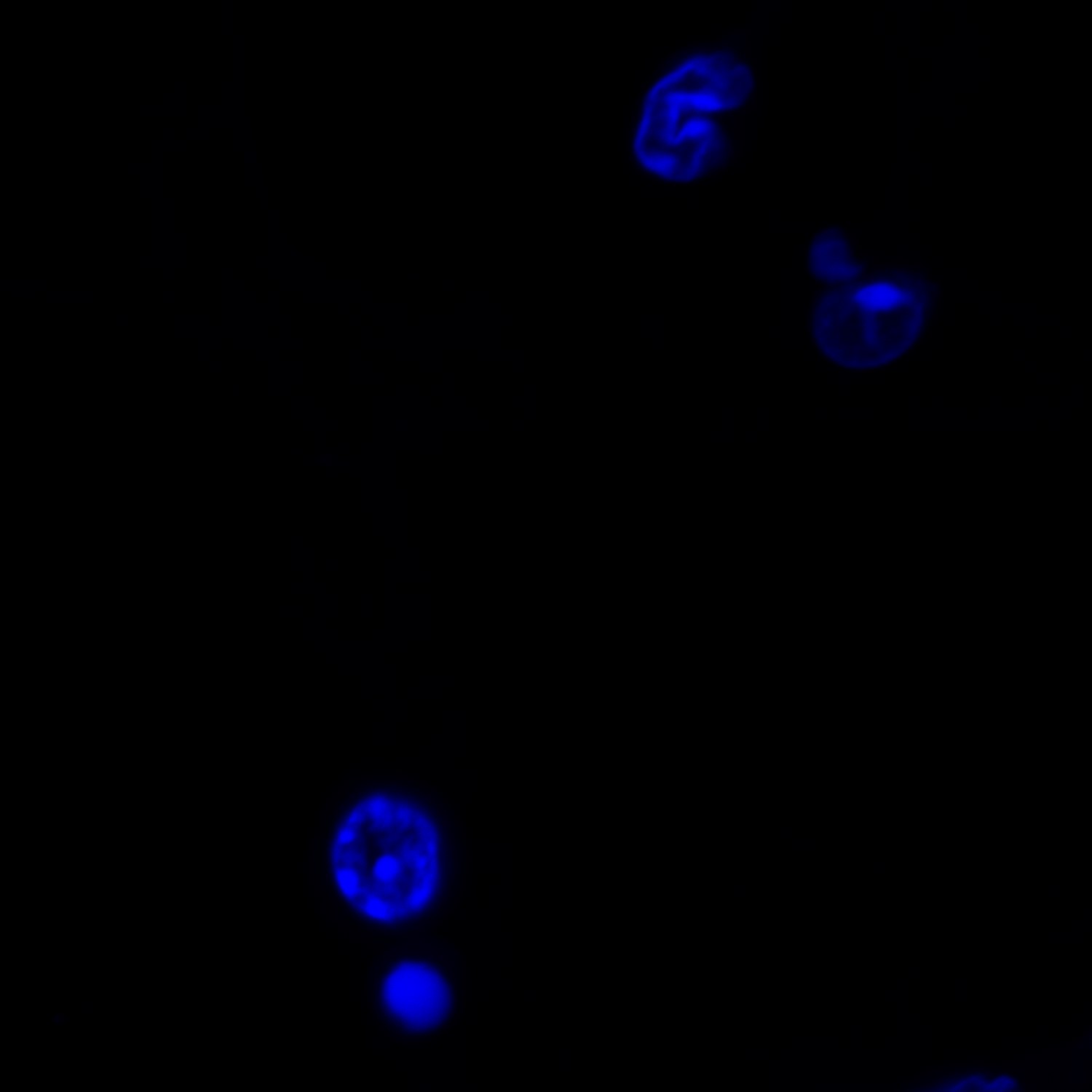

Supplement: S1 File — (ZIP) [file ppat.1012230.s002.zip › S1_File/Fig_7A/Lung/WT-IAV-DAPI.jpg]

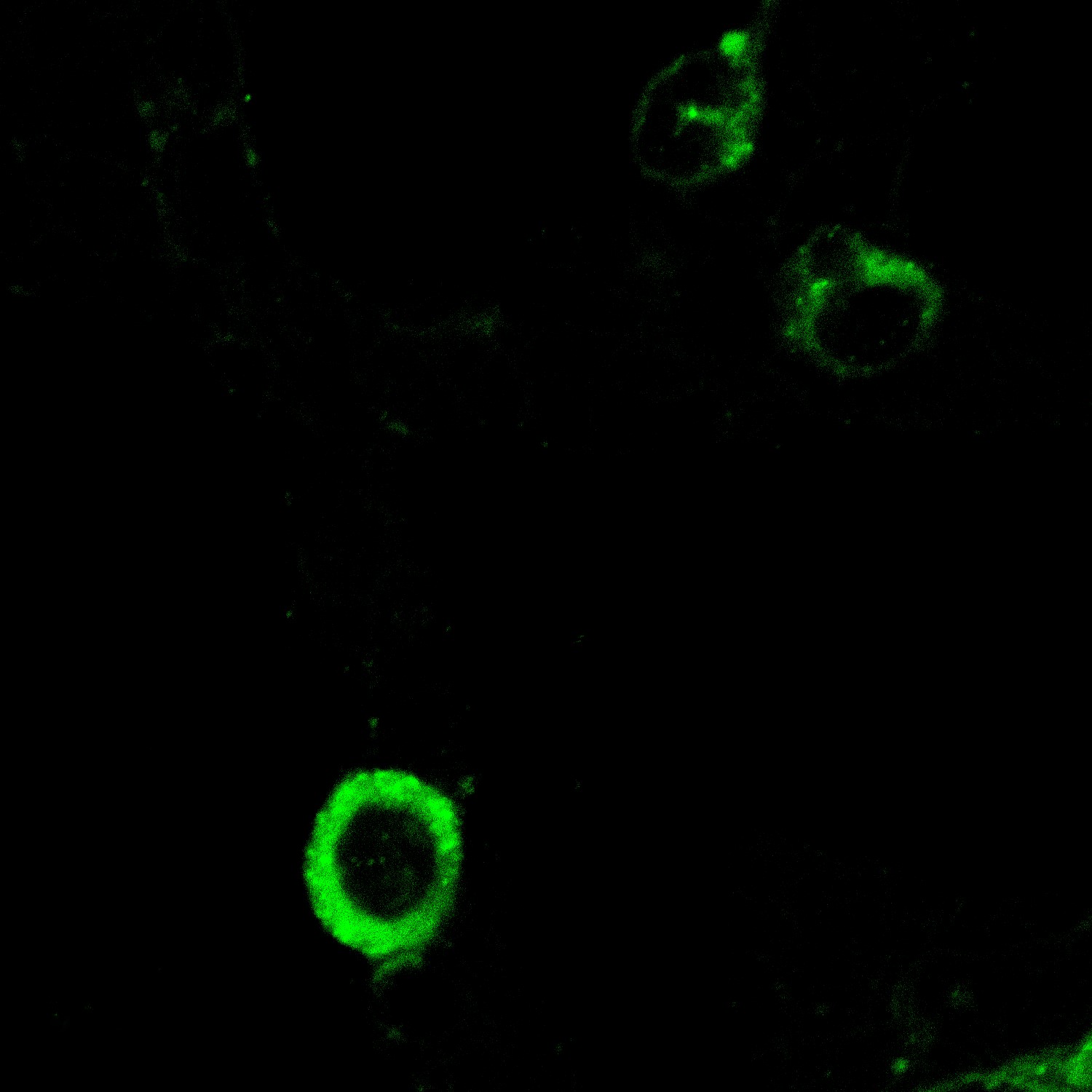

Supplement: S1 File — (ZIP) [file ppat.1012230.s002.zip › S1_File/Fig_7A/Lung/WT-IAV-F480.jpg]

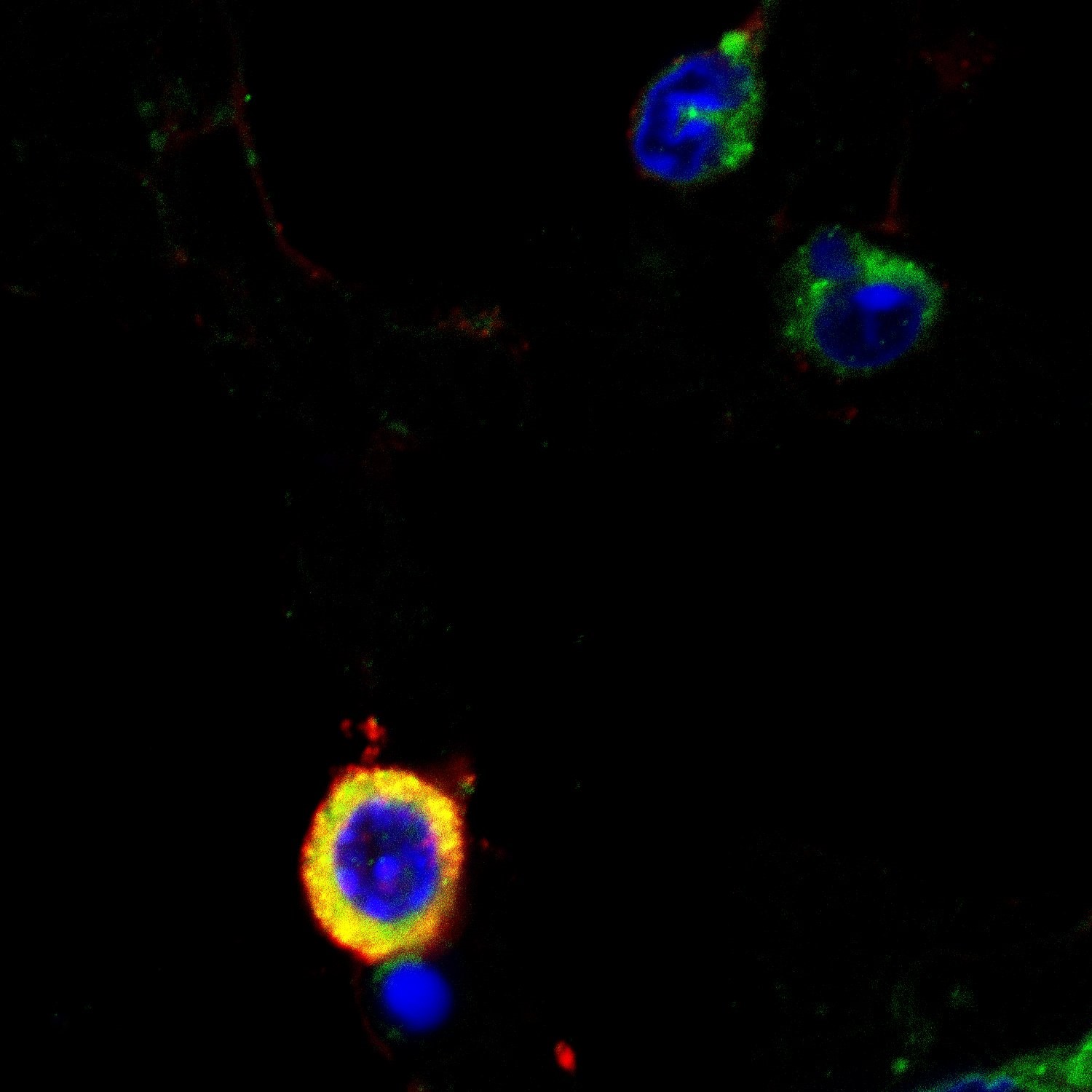

Supplement: S1 File — (ZIP) [file ppat.1012230.s002.zip › S1_File/Fig_7A/Lung/WT-IAV-Merge.jpg]

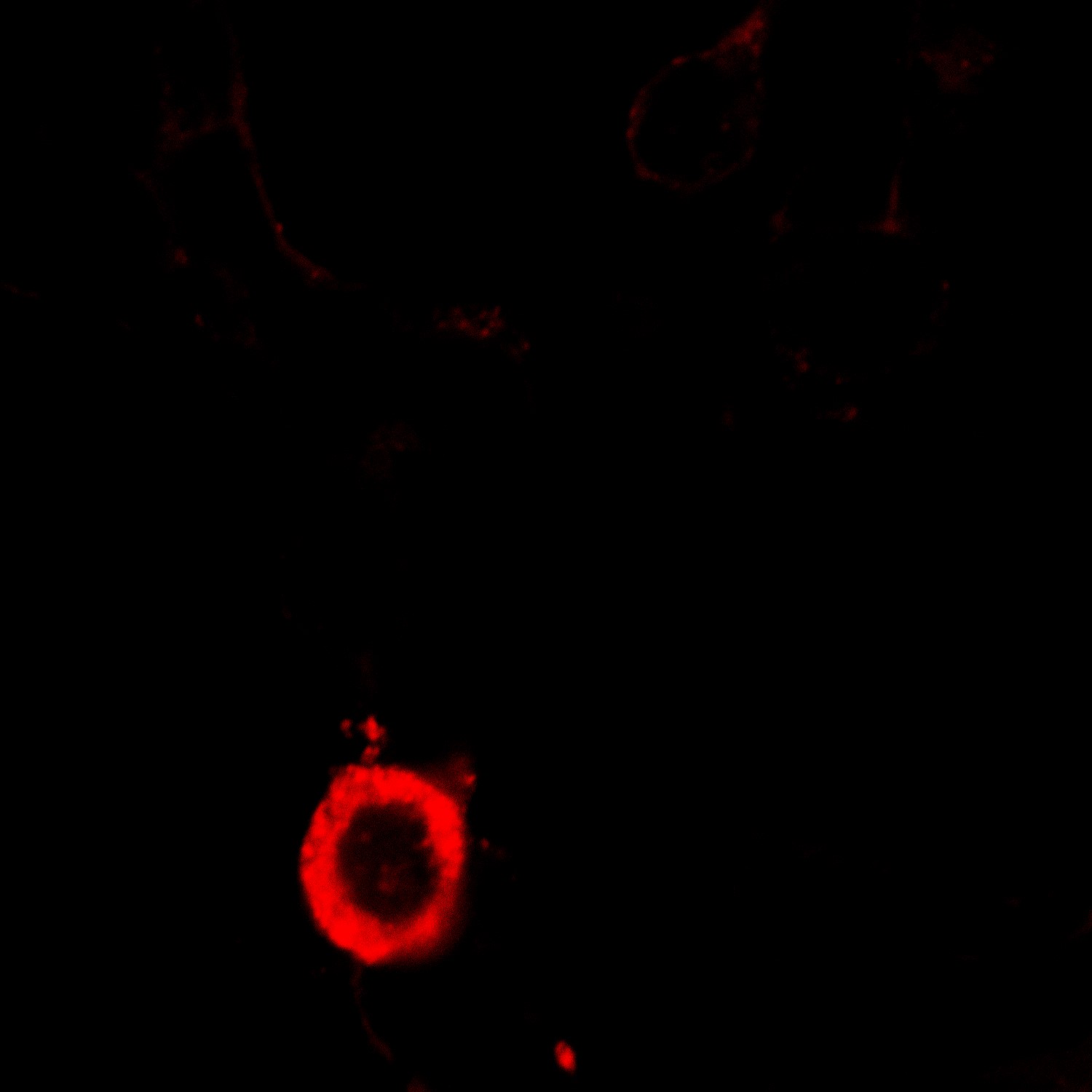

Supplement: S1 File — (ZIP) [file ppat.1012230.s002.zip › S1_File/Fig_7A/Lung/WT-IAV-NP.jpg]

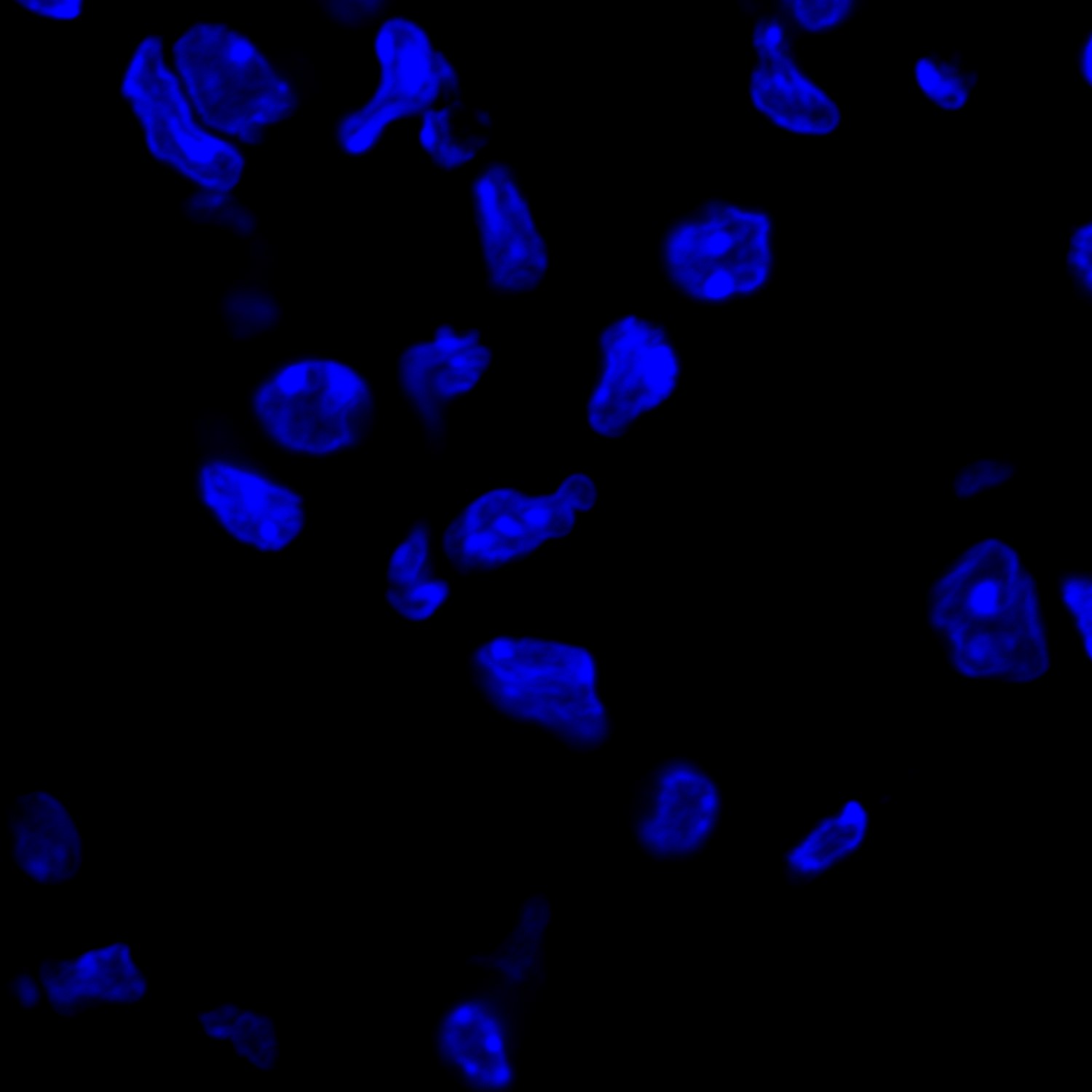

Supplement: S1 File — (ZIP) [file ppat.1012230.s002.zip › S1_File/Fig_7A/Lung/WT-Mock-DAPI.jpg]

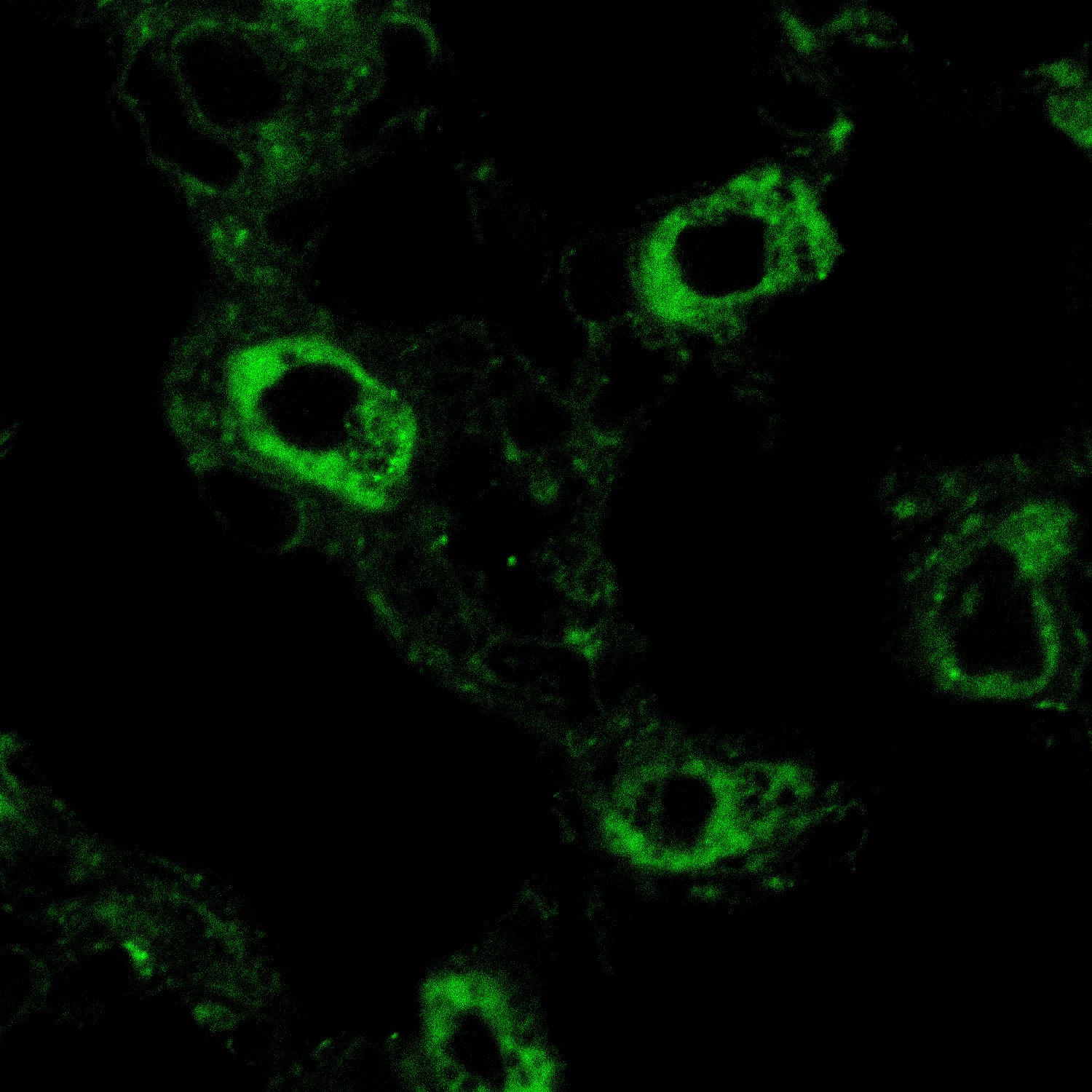

Supplement: S1 File — (ZIP) [file ppat.1012230.s002.zip › S1_File/Fig_7A/Lung/WT-Mock-F480.jpg]

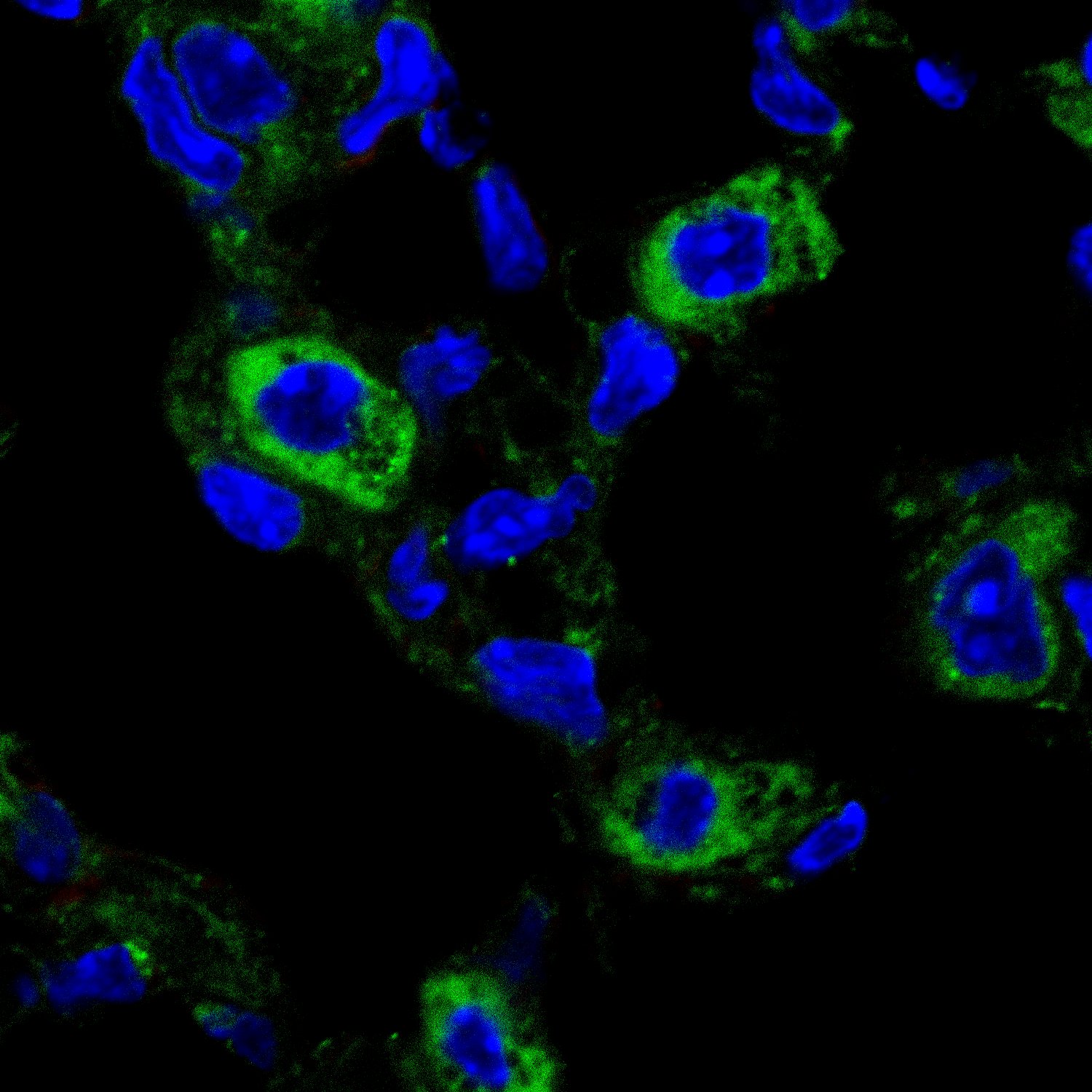

Supplement: S1 File — (ZIP) [file ppat.1012230.s002.zip › S1_File/Fig_7A/Lung/WT-Mock-Merge.jpg]

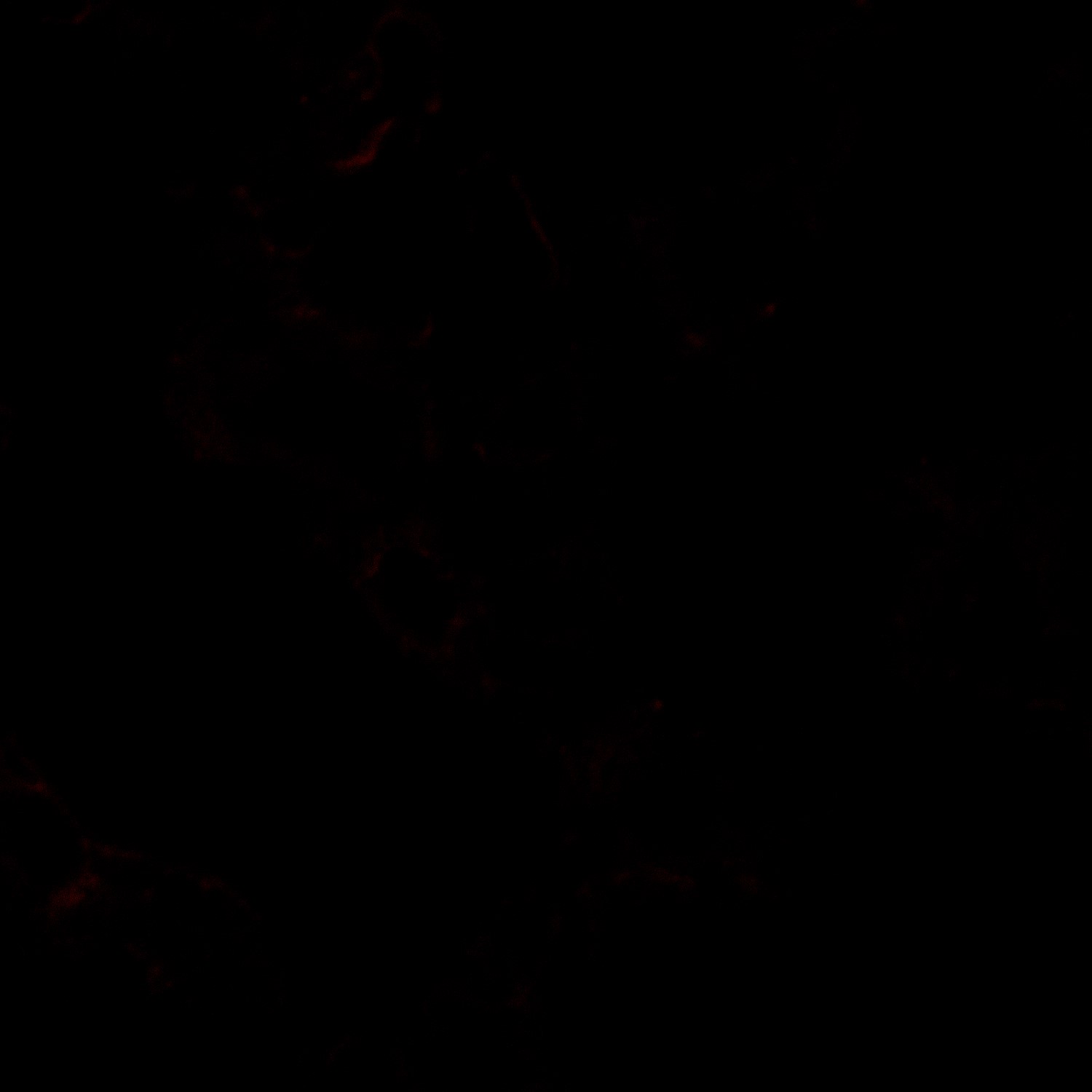

Supplement: S1 File — (ZIP) [file ppat.1012230.s002.zip › S1_File/Fig_7A/Lung/WT-Mock-NP.jpg]

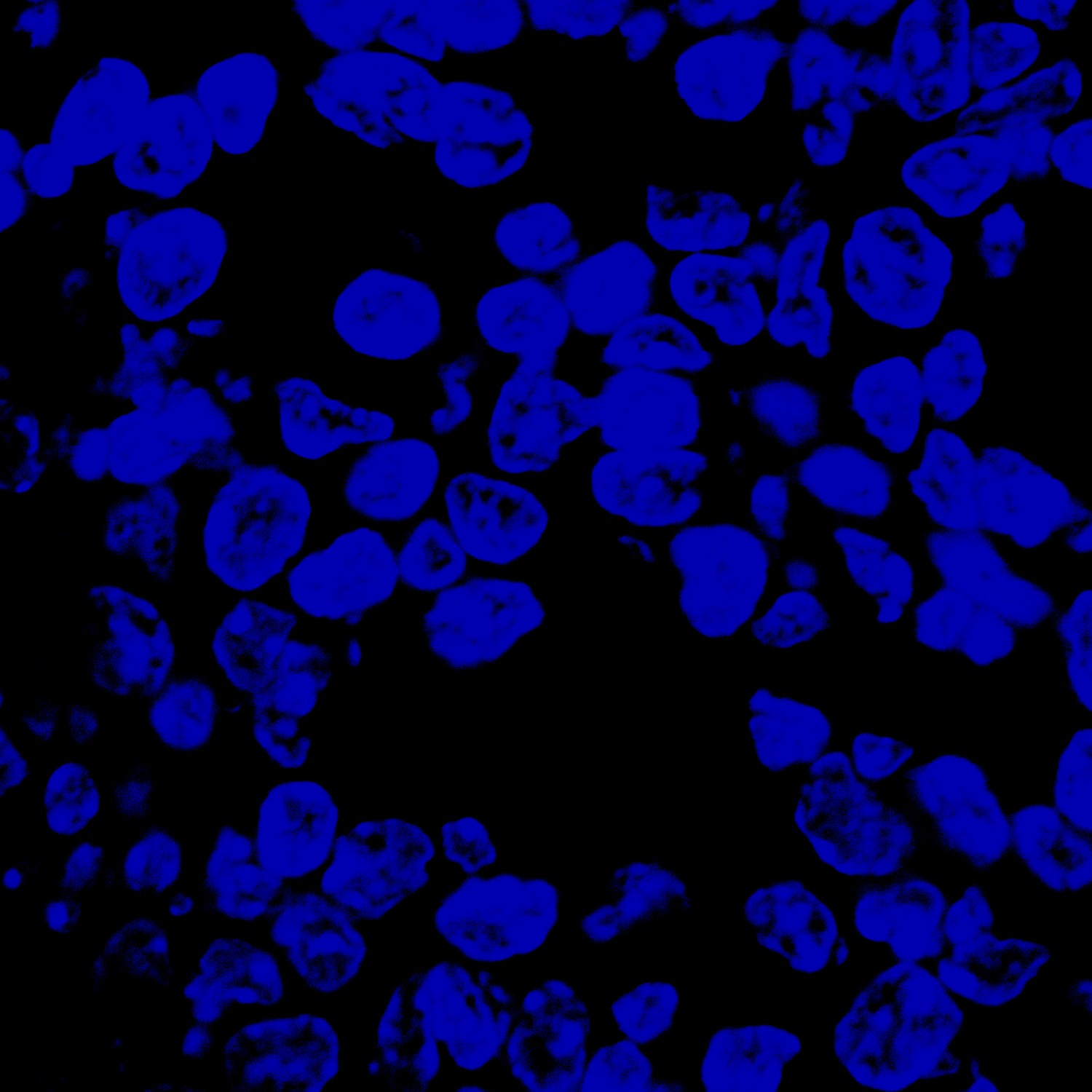

Supplement: S1 File — (ZIP) [file ppat.1012230.s002.zip › S1_File/Fig_7A/Spleen/Adap KO-IAV-DAPI.jpg]

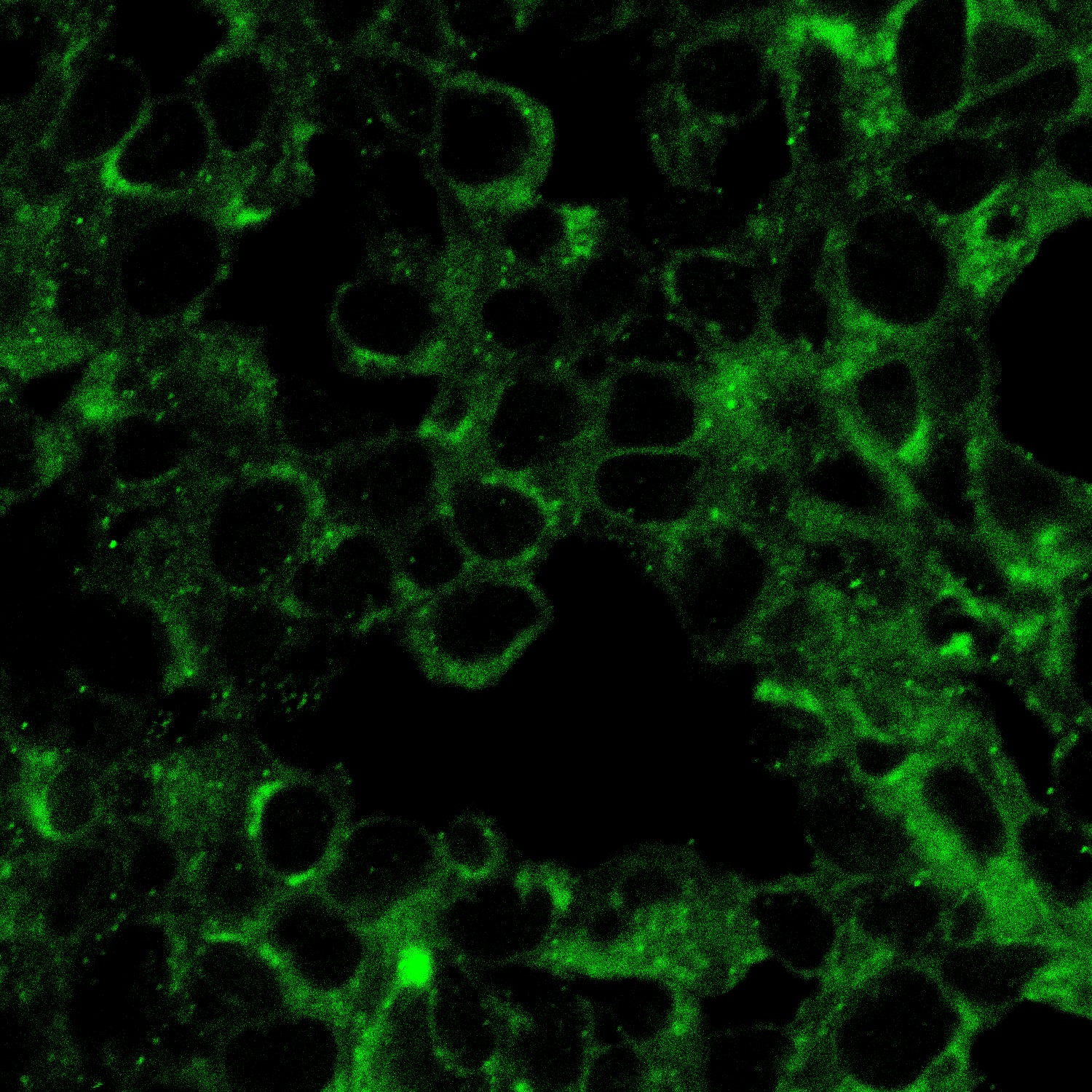

Supplement: S1 File — (ZIP) [file ppat.1012230.s002.zip › S1_File/Fig_7A/Spleen/Adap KO-IAV-F480.jpg]

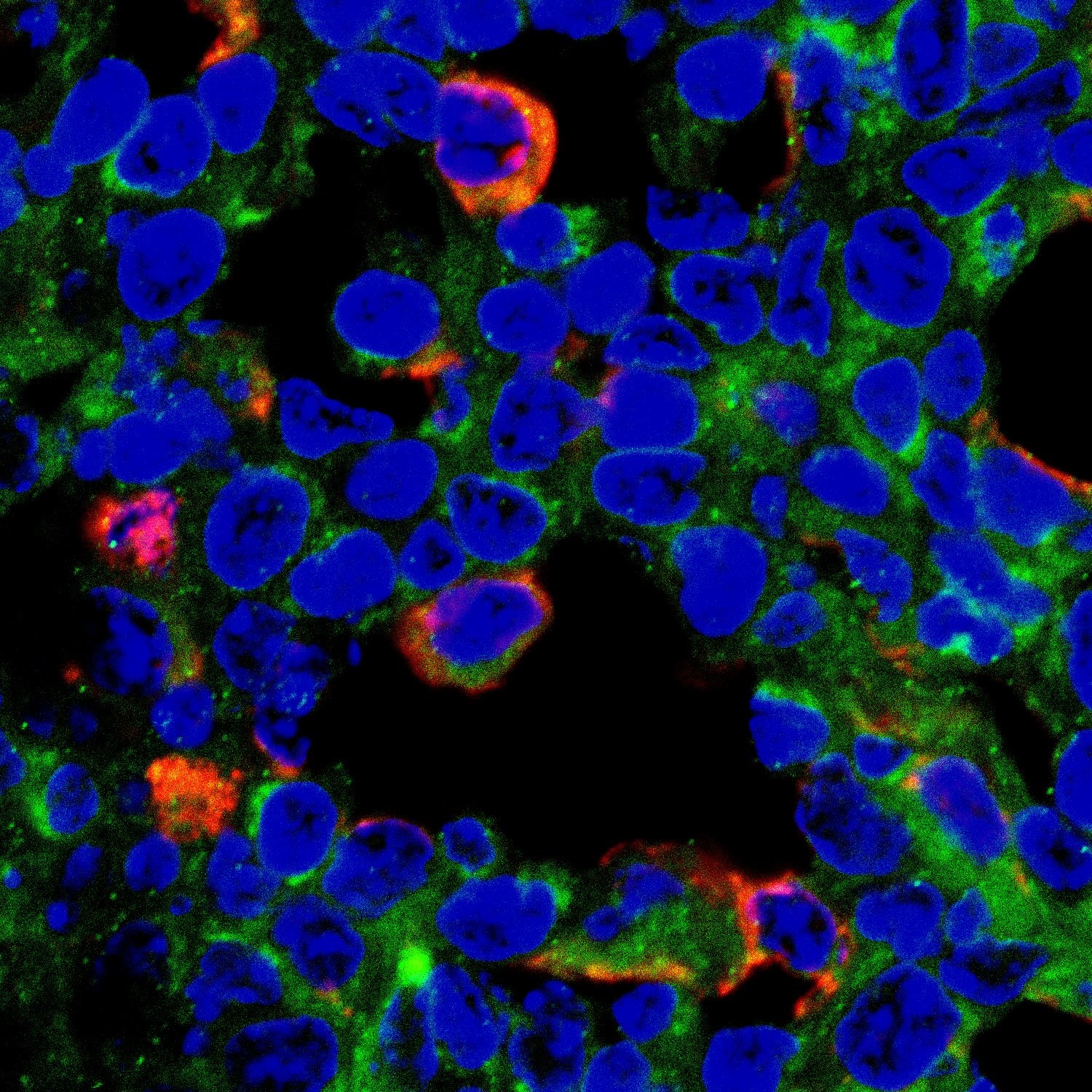

Supplement: S1 File — (ZIP) [file ppat.1012230.s002.zip › S1_File/Fig_7A/Spleen/Adap KO-IAV-Merge.jpg]

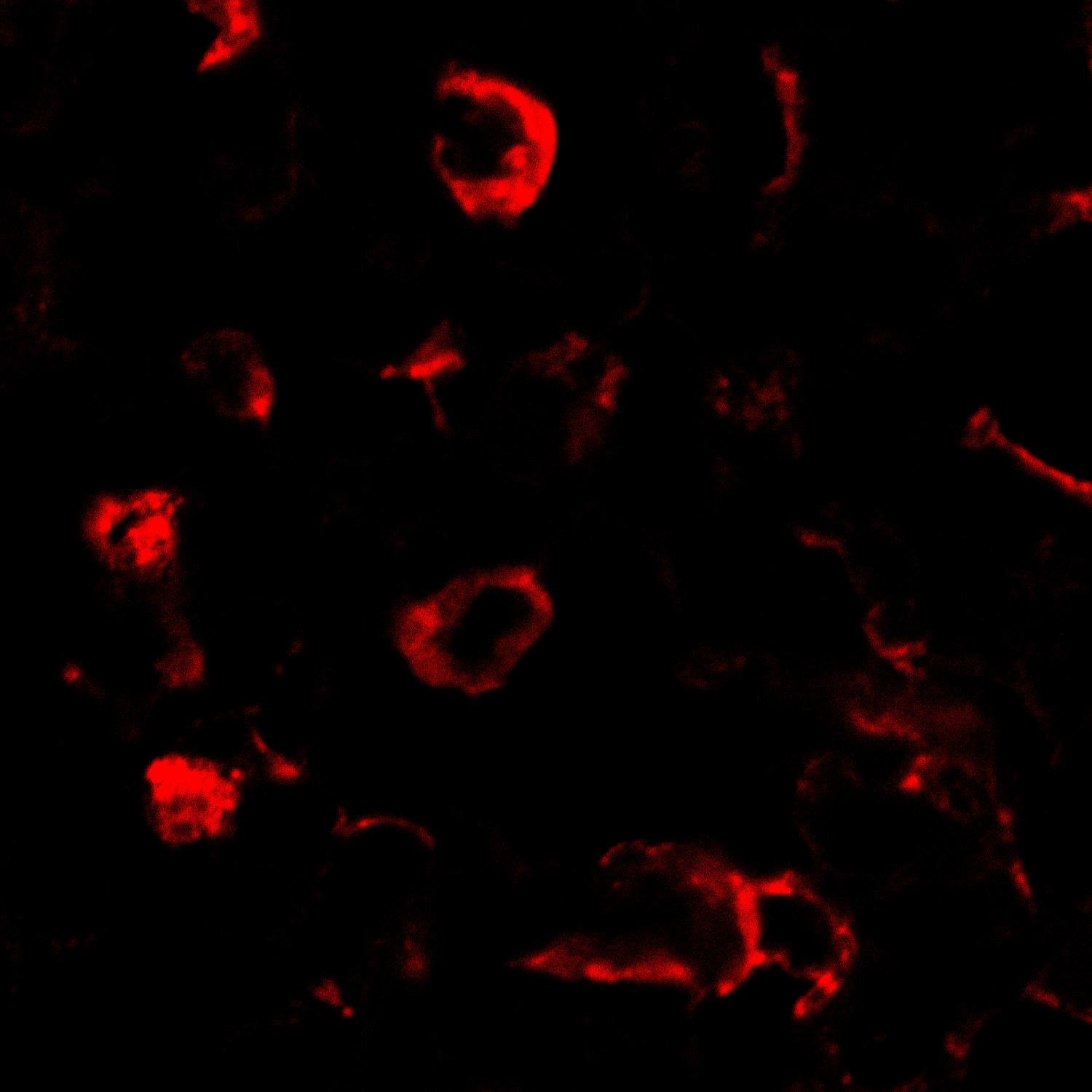

Supplement: S1 File — (ZIP) [file ppat.1012230.s002.zip › S1_File/Fig_7A/Spleen/Adap KO-IAV-NP.jpg]

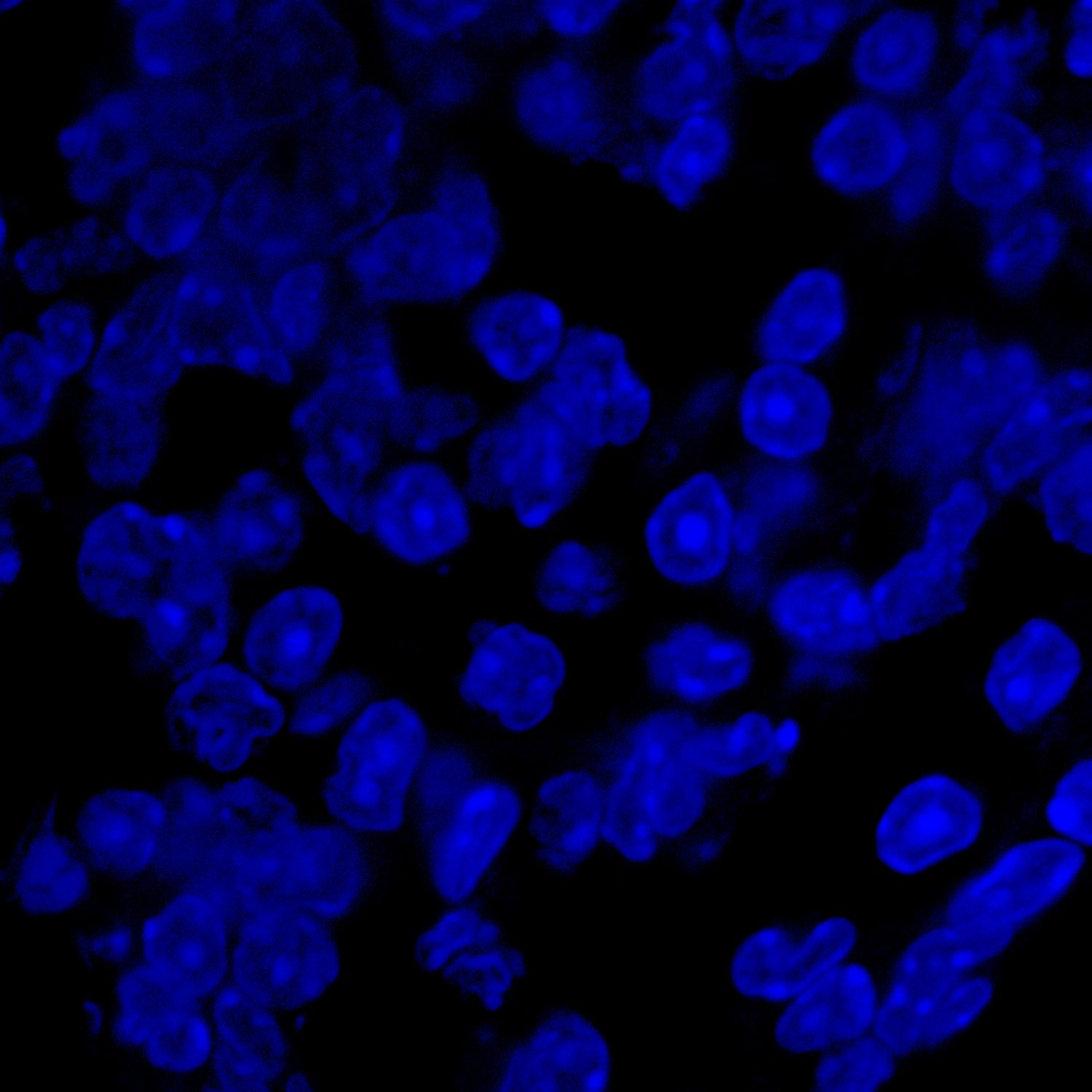

Supplement: S1 File — (ZIP) [file ppat.1012230.s002.zip › S1_File/Fig_7A/Spleen/Adap KO-Mock-DAPI.jpg]

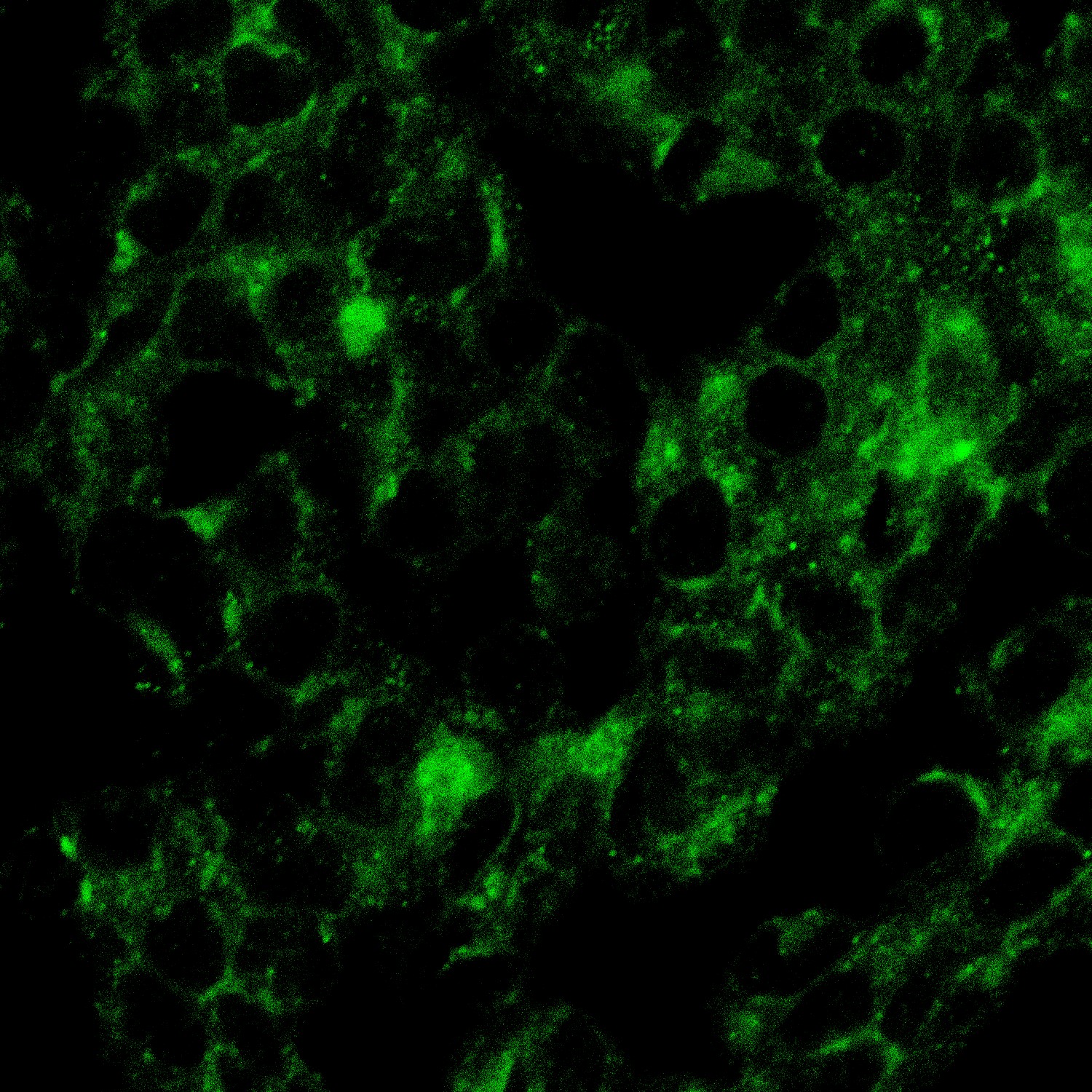

Supplement: S1 File — (ZIP) [file ppat.1012230.s002.zip › S1_File/Fig_7A/Spleen/Adap KO-Mock-F480.jpg]

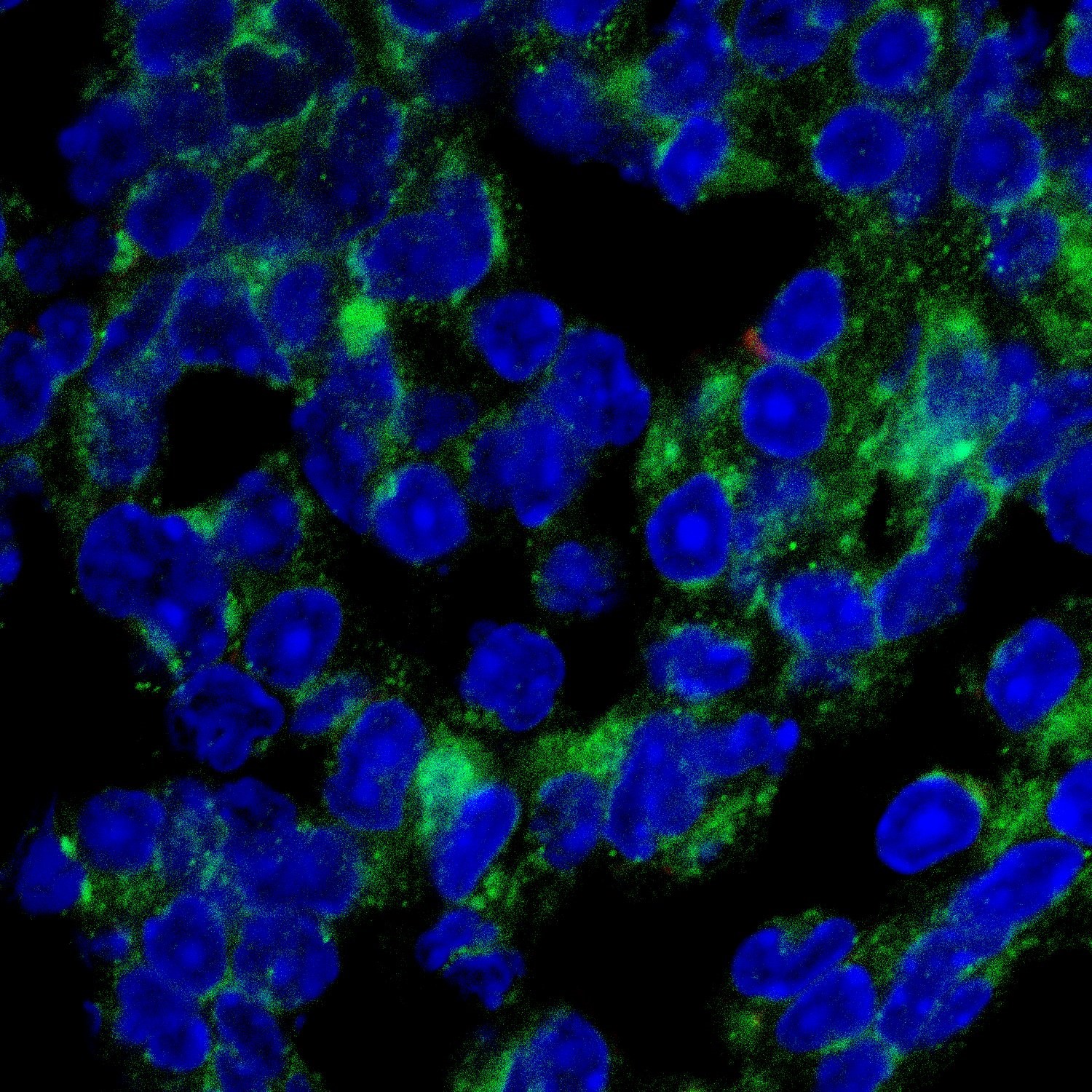

Supplement: S1 File — (ZIP) [file ppat.1012230.s002.zip › S1_File/Fig_7A/Spleen/Adap KO-Mock-Merge.jpg]

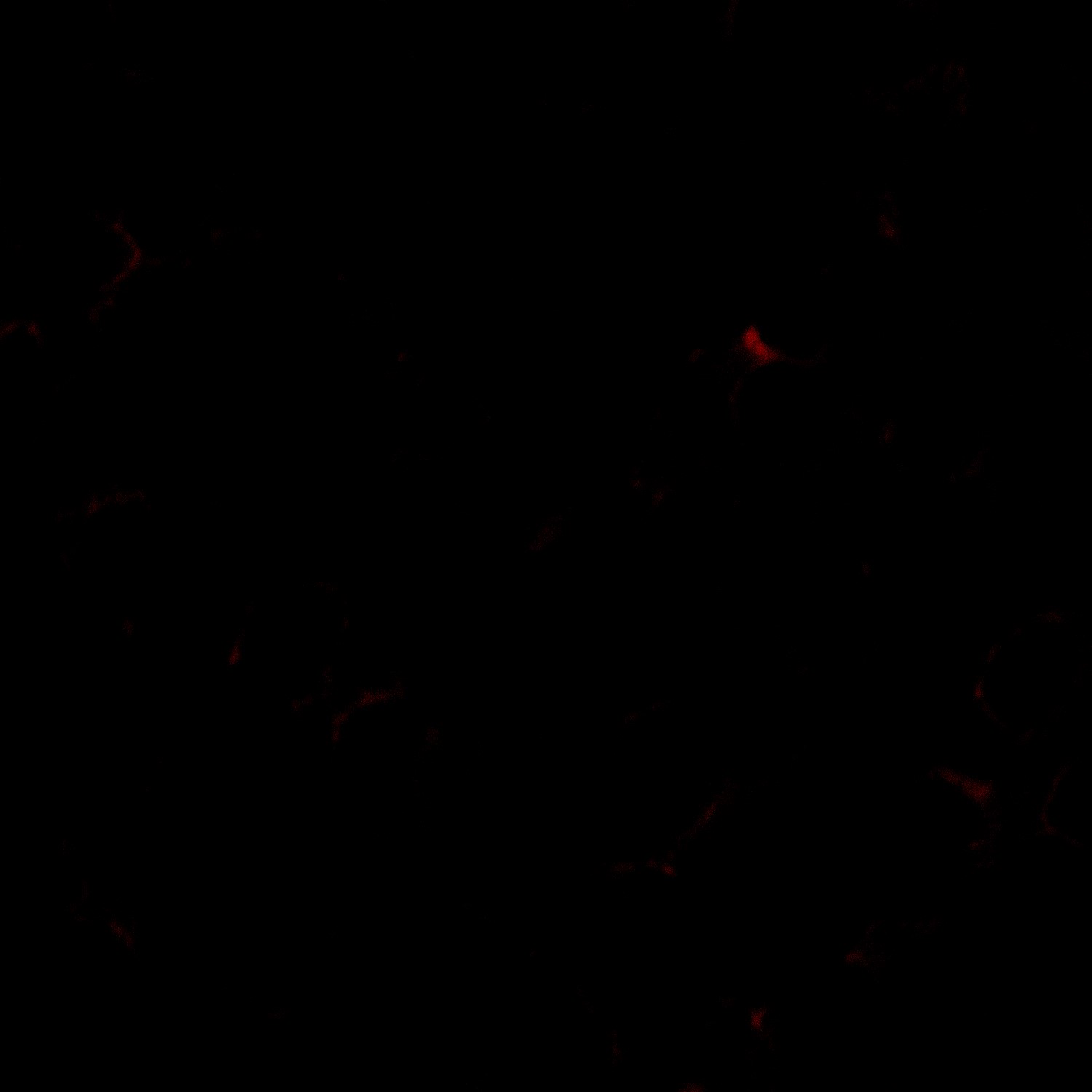

Supplement: S1 File — (ZIP) [file ppat.1012230.s002.zip › S1_File/Fig_7A/Spleen/Adap KO-Mock-NP.jpg]

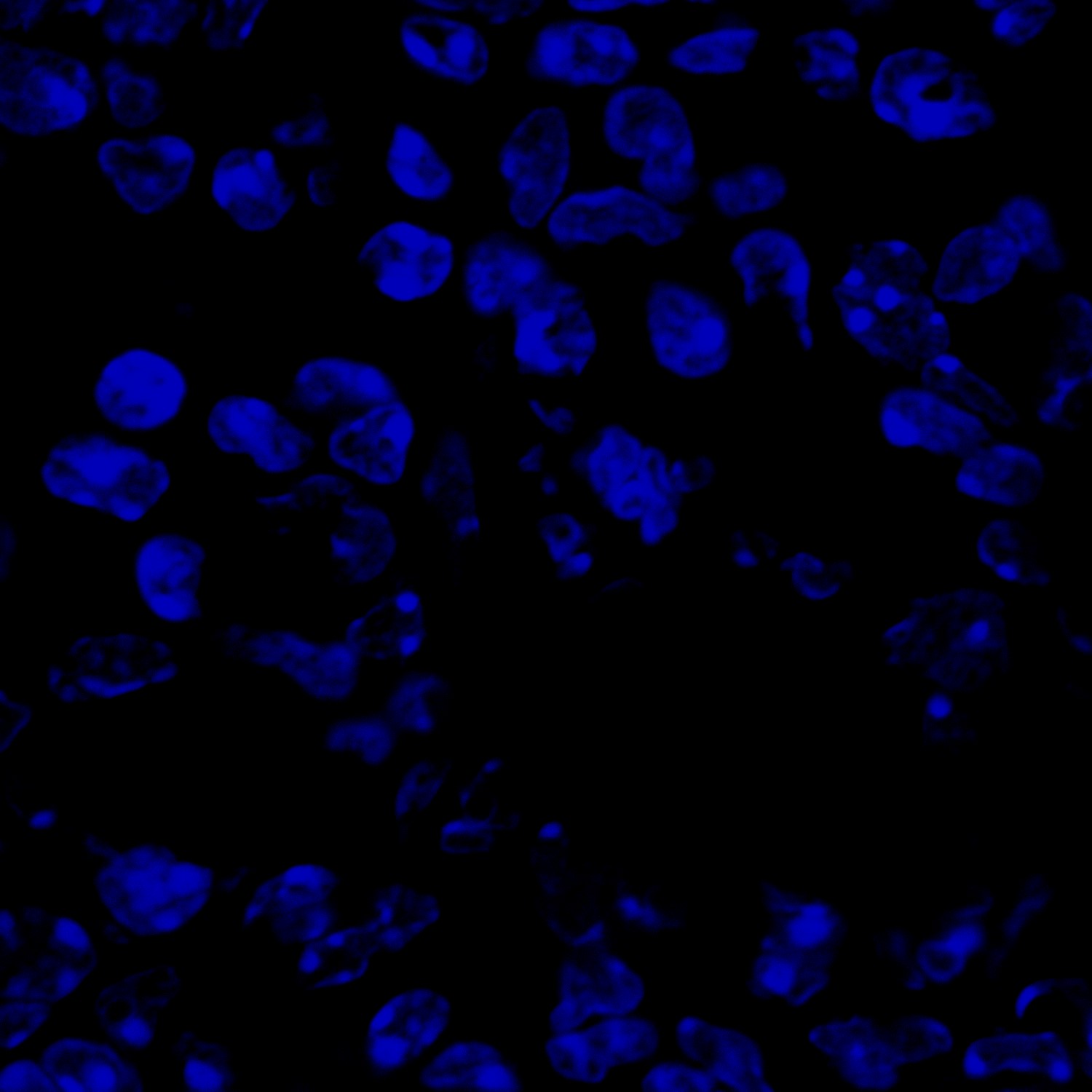

Supplement: S1 File — (ZIP) [file ppat.1012230.s002.zip › S1_File/Fig_7A/Spleen/WT-IAV-DAPI.jpg]

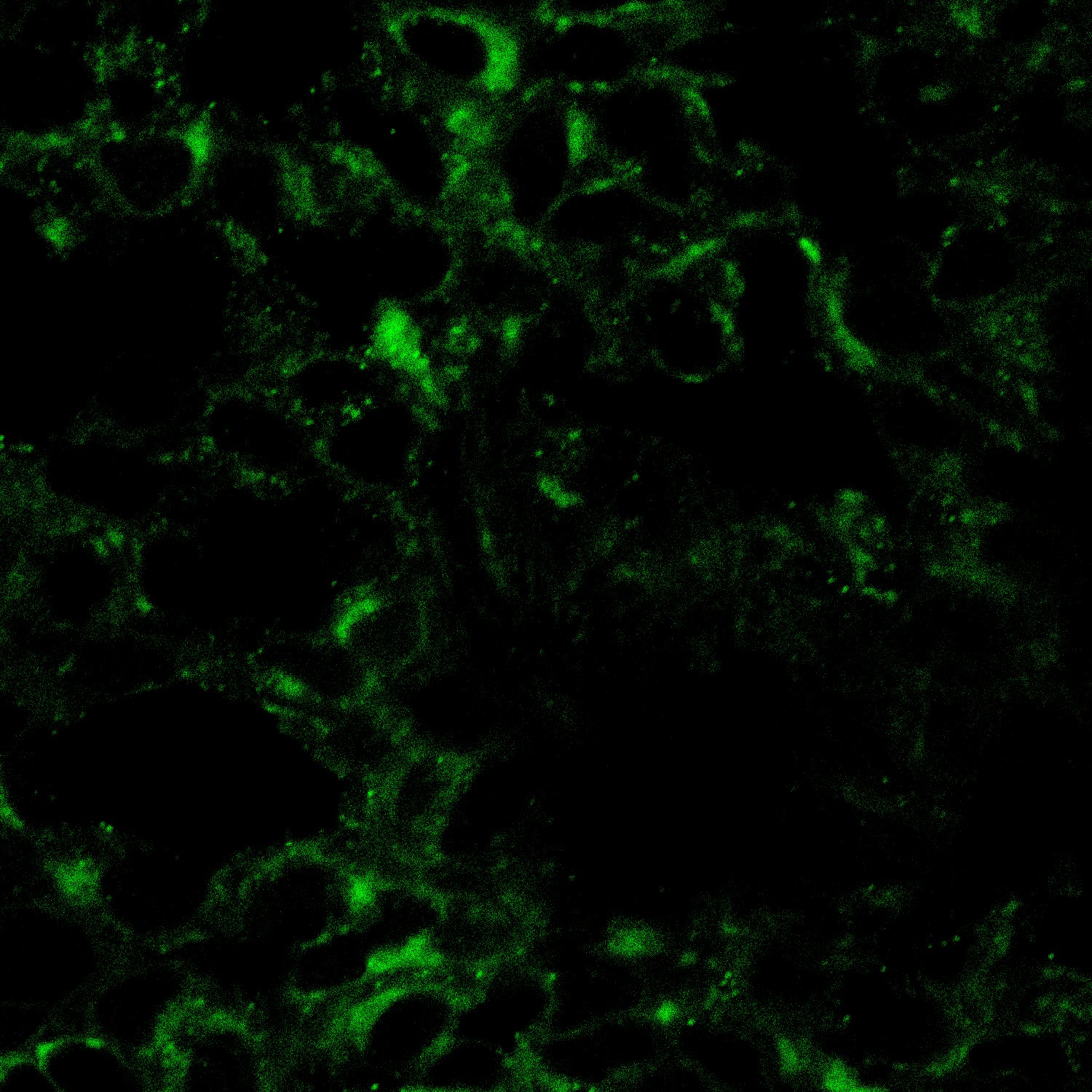

Supplement: S1 File — (ZIP) [file ppat.1012230.s002.zip › S1_File/Fig_7A/Spleen/WT-IAV-F480.jpg]

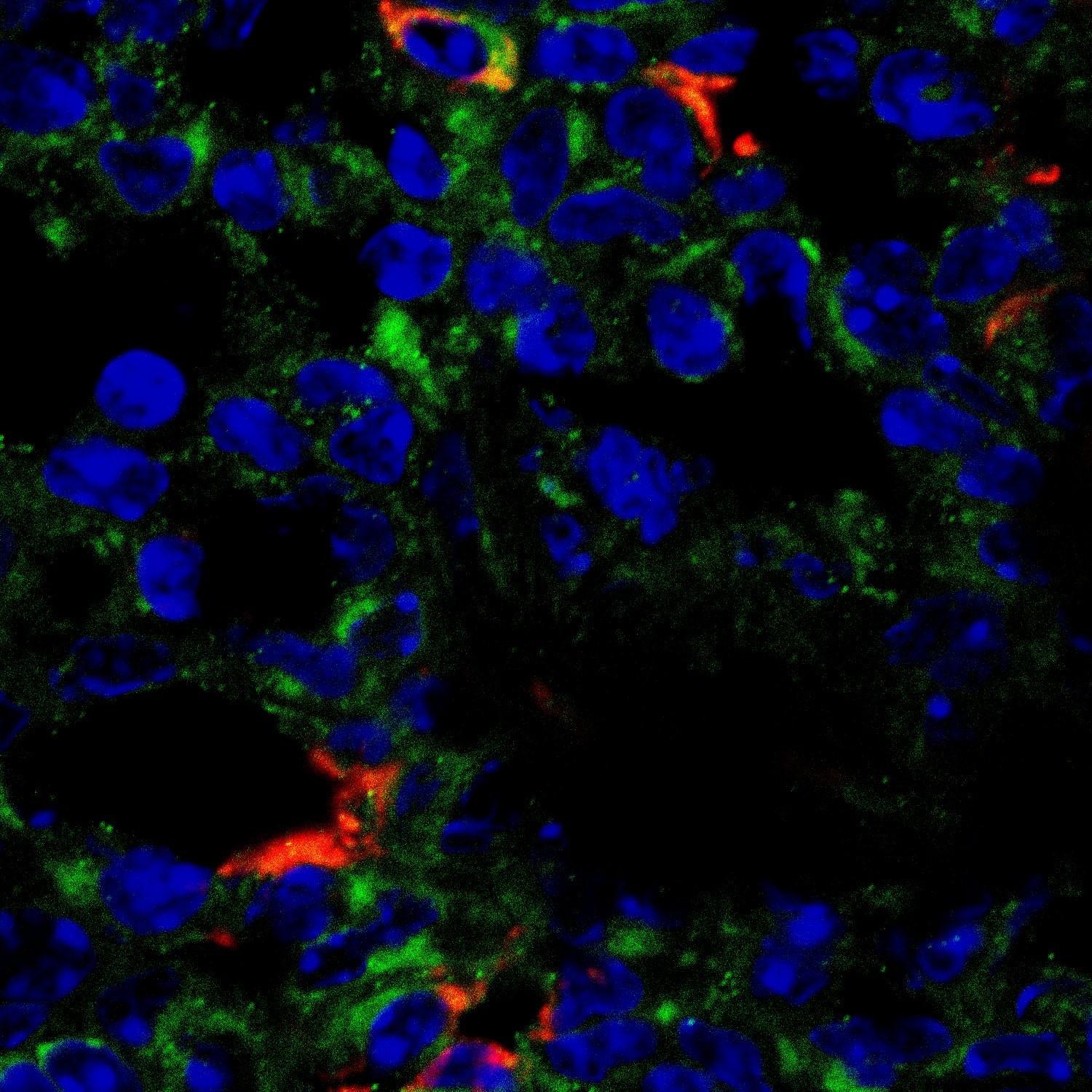

Supplement: S1 File — (ZIP) [file ppat.1012230.s002.zip › S1_File/Fig_7A/Spleen/WT-IAV-Merge.jpg]

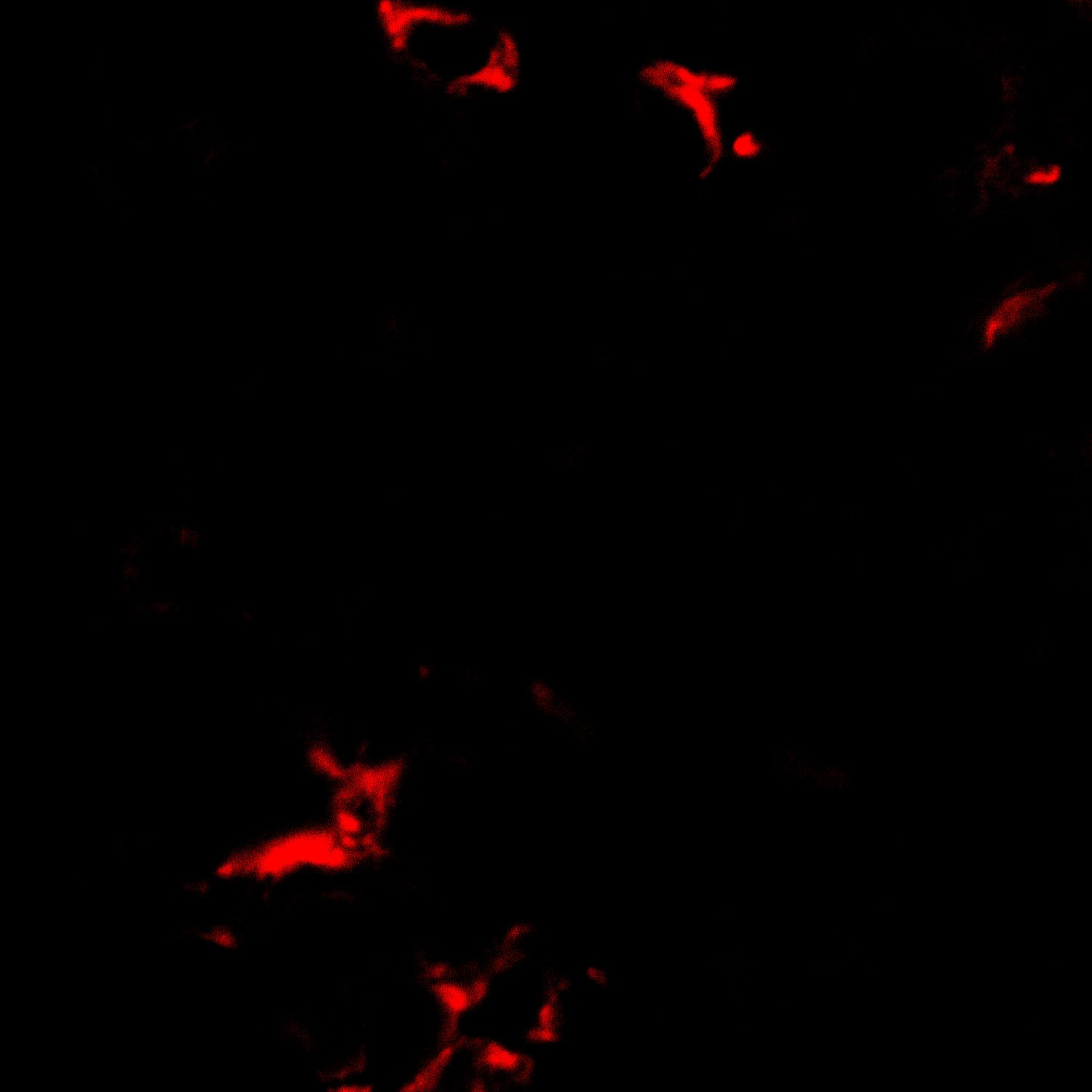

Supplement: S1 File — (ZIP) [file ppat.1012230.s002.zip › S1_File/Fig_7A/Spleen/WT-IAV-NP.jpg]

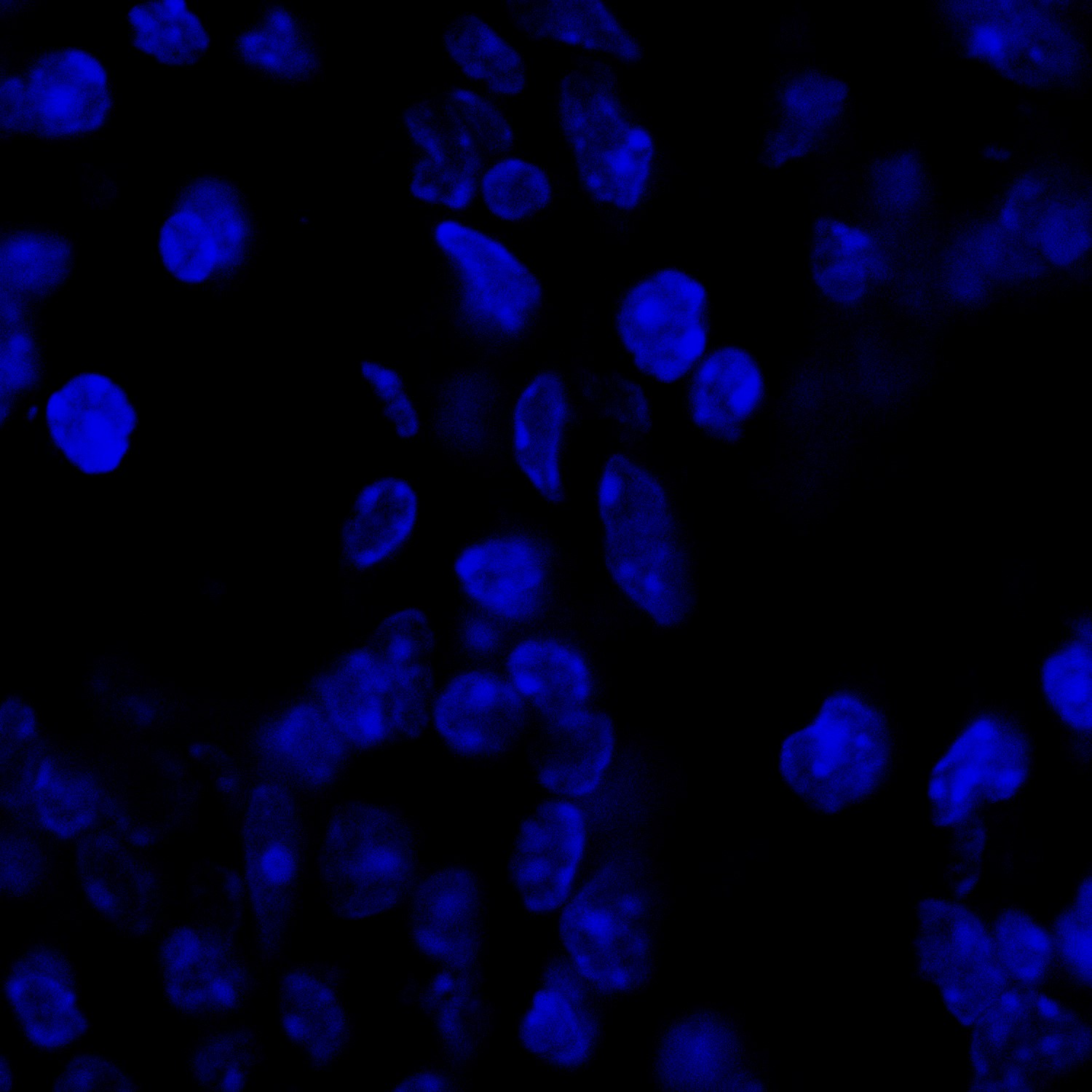

Supplement: S1 File — (ZIP) [file ppat.1012230.s002.zip › S1_File/Fig_7A/Spleen/WT-Mock-DAPI.jpg]

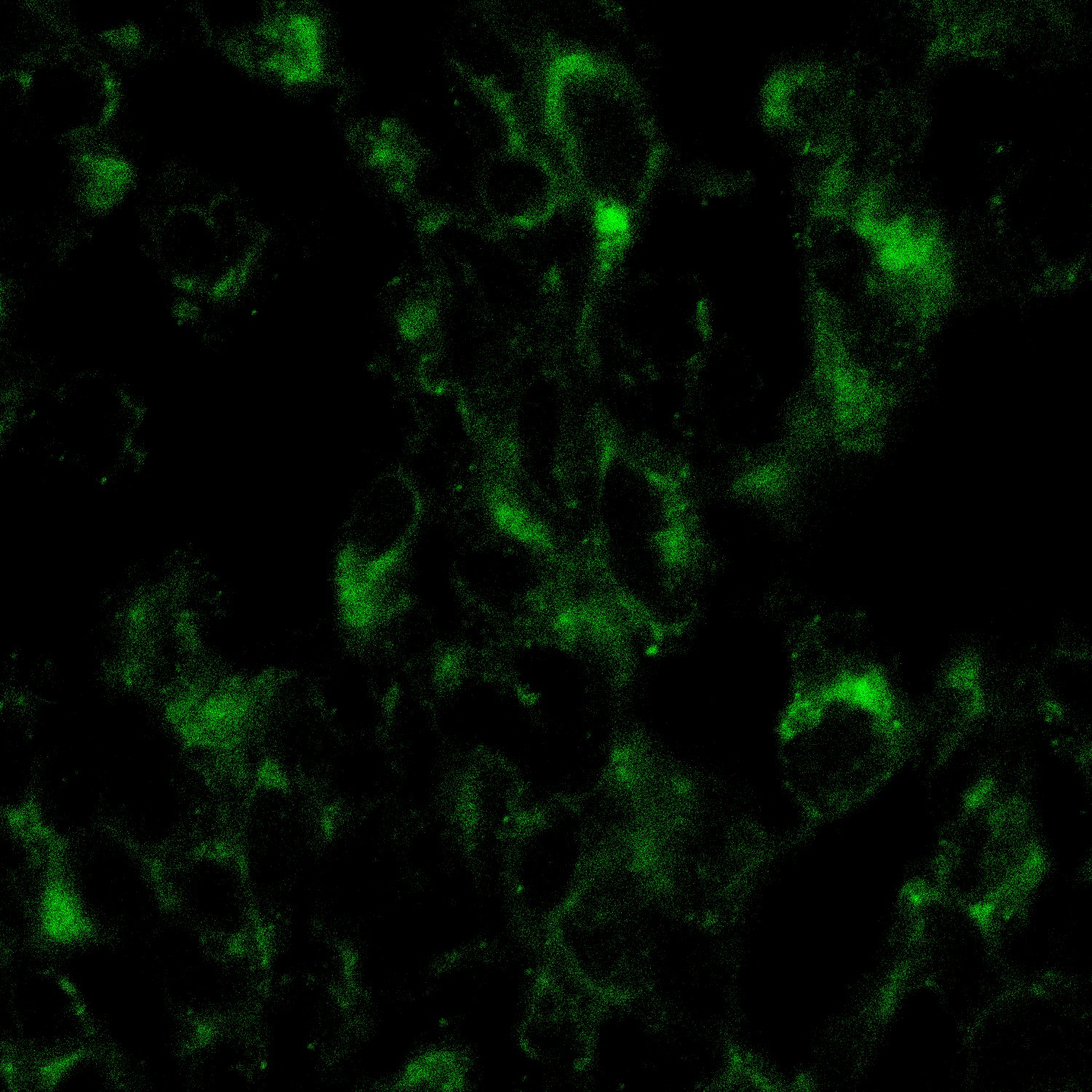

Supplement: S1 File — (ZIP) [file ppat.1012230.s002.zip › S1_File/Fig_7A/Spleen/WT-Mock-F480.jpg]

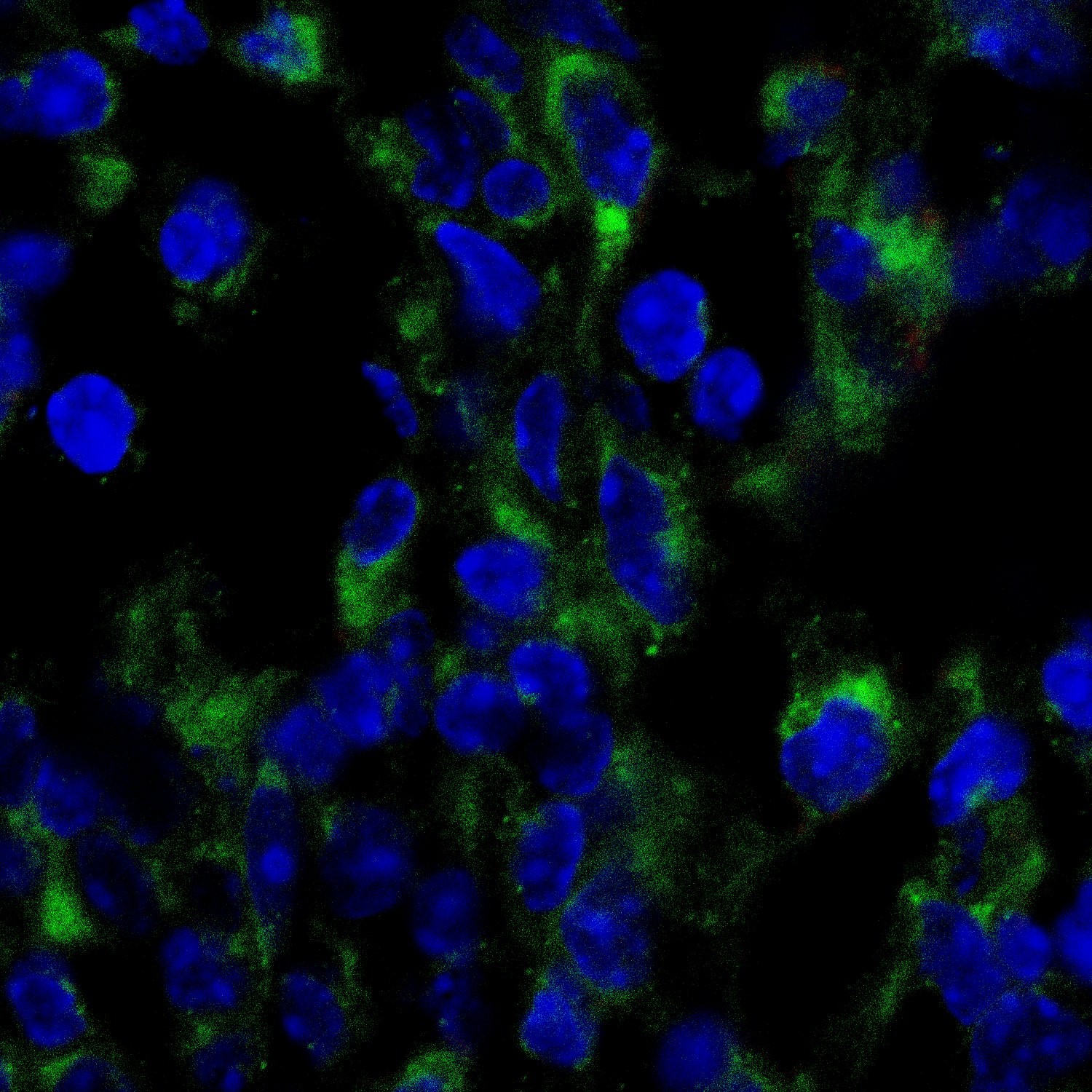

Supplement: S1 File — (ZIP) [file ppat.1012230.s002.zip › S1_File/Fig_7A/Spleen/WT-Mock-Merge.jpg]

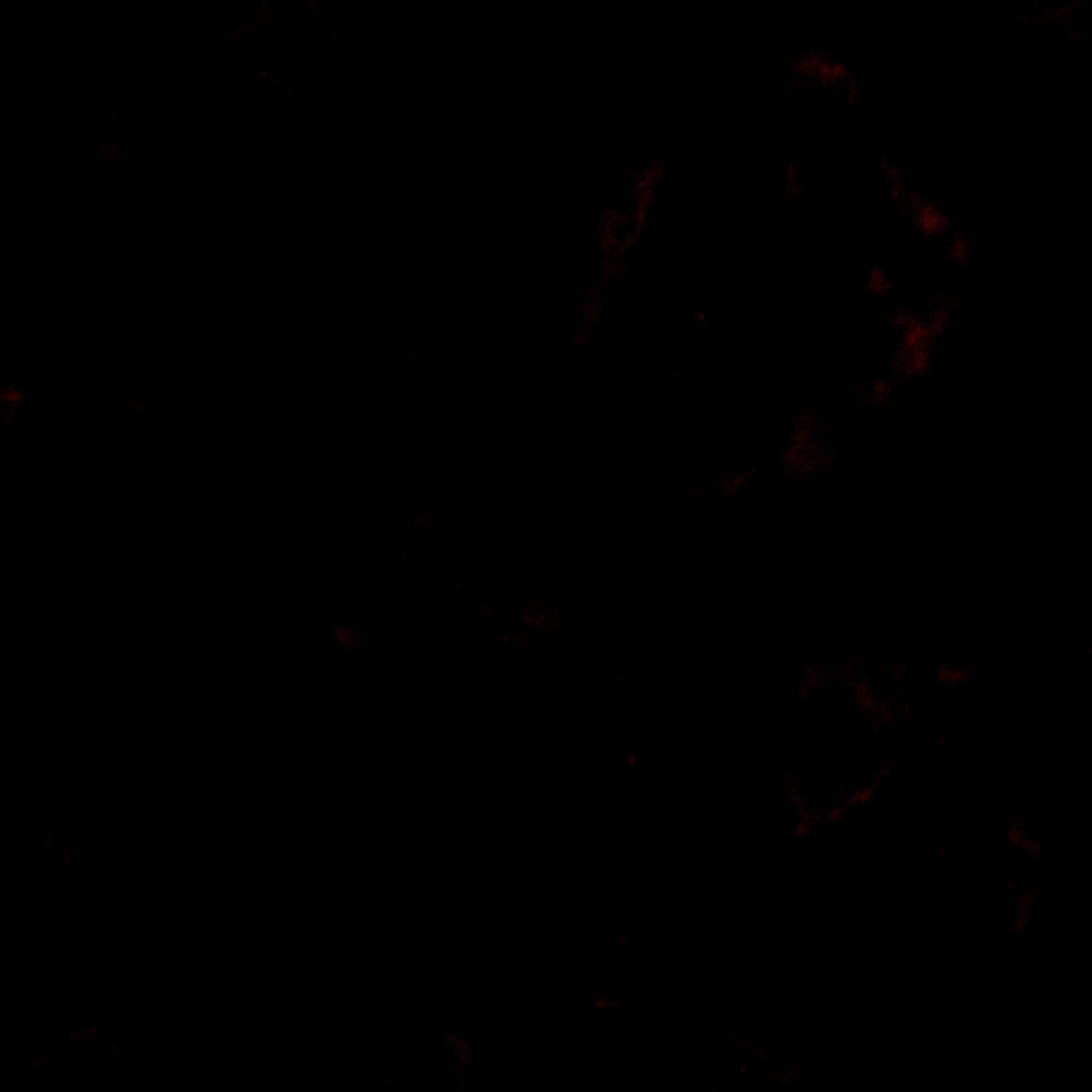

Supplement: S1 File — (ZIP) [file ppat.1012230.s002.zip › S1_File/Fig_7A/Spleen/WT-Mock-NP.jpg]

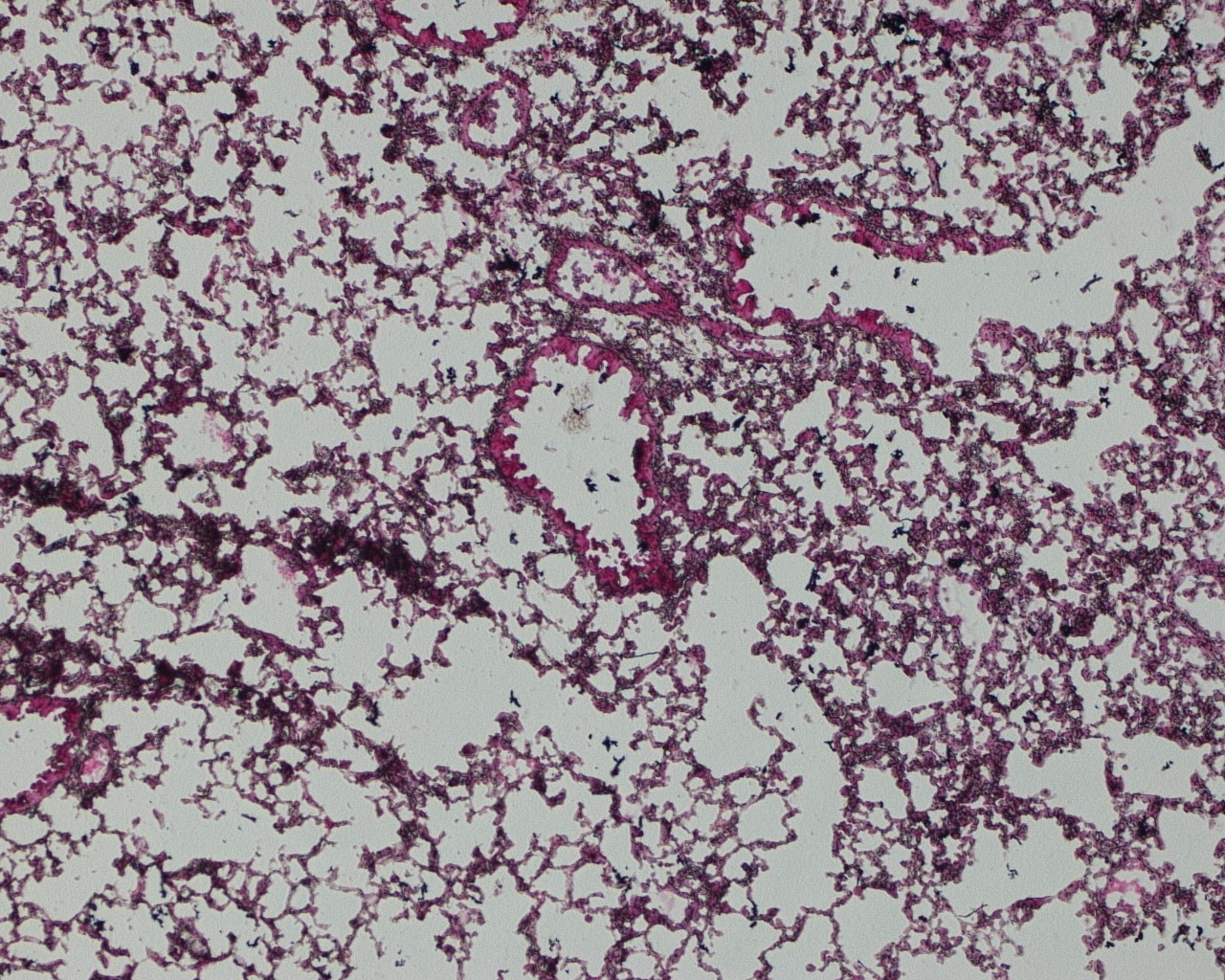

Supplement: S1 File — (ZIP) [file ppat.1012230.s002.zip › S1_File/Fig_7C/Lung/Adap KO-IAV-10X.jpg]

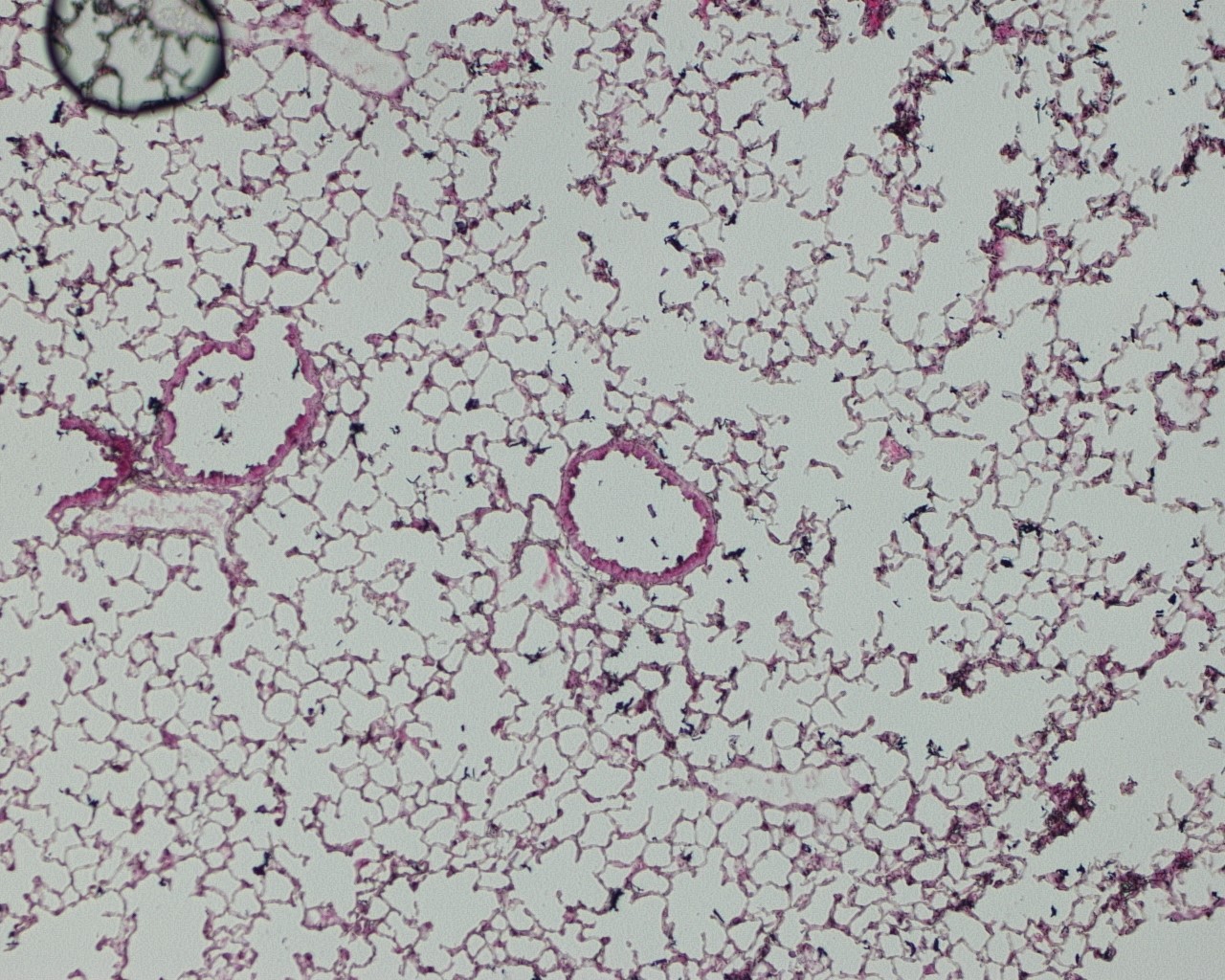

Supplement: S1 File — (ZIP) [file ppat.1012230.s002.zip › S1_File/Fig_7C/Lung/Adap KO-Mock-10X.jpg]

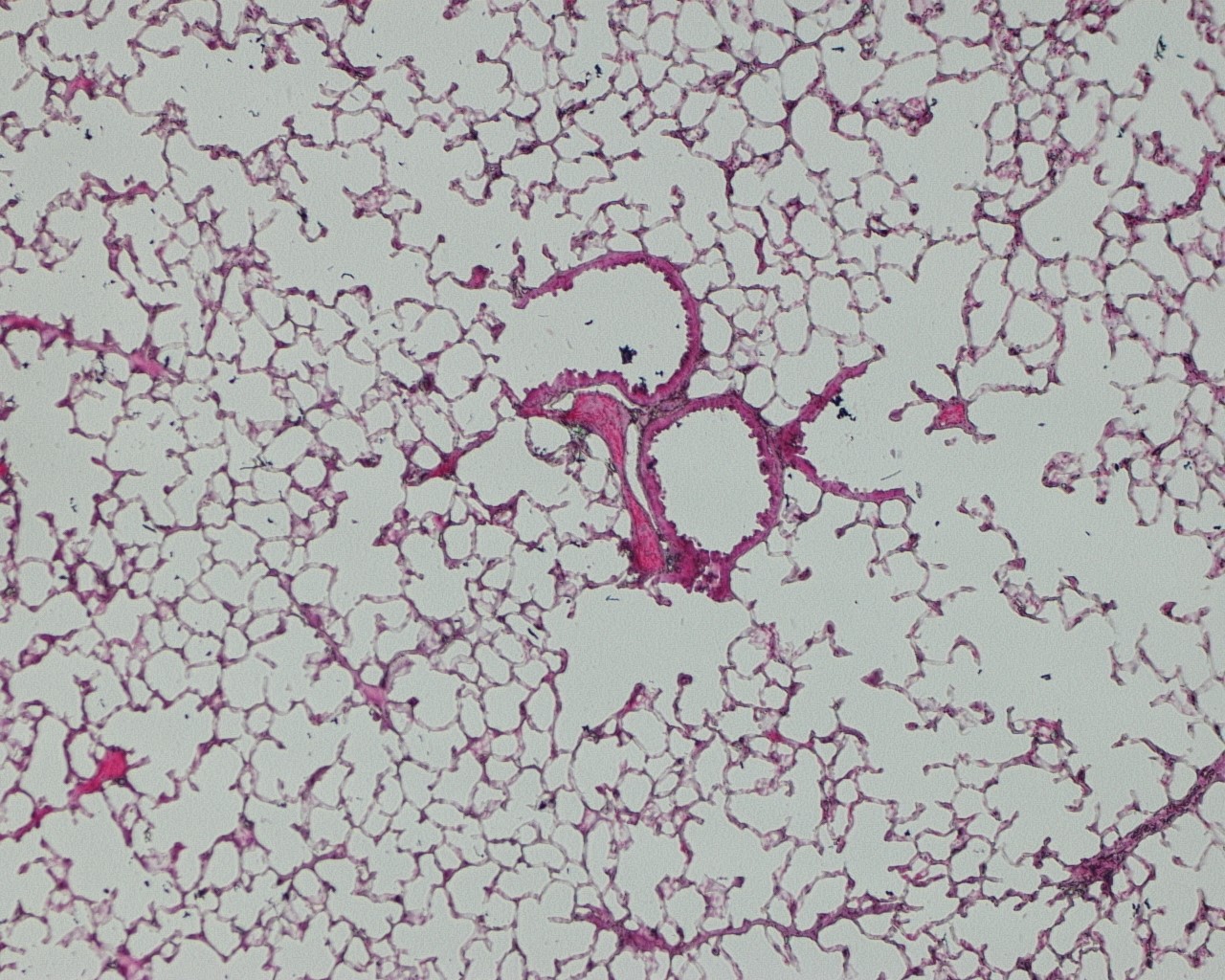

Supplement: S1 File — (ZIP) [file ppat.1012230.s002.zip › S1_File/Fig_7C/Lung/WT-IAV-10X.jpg]

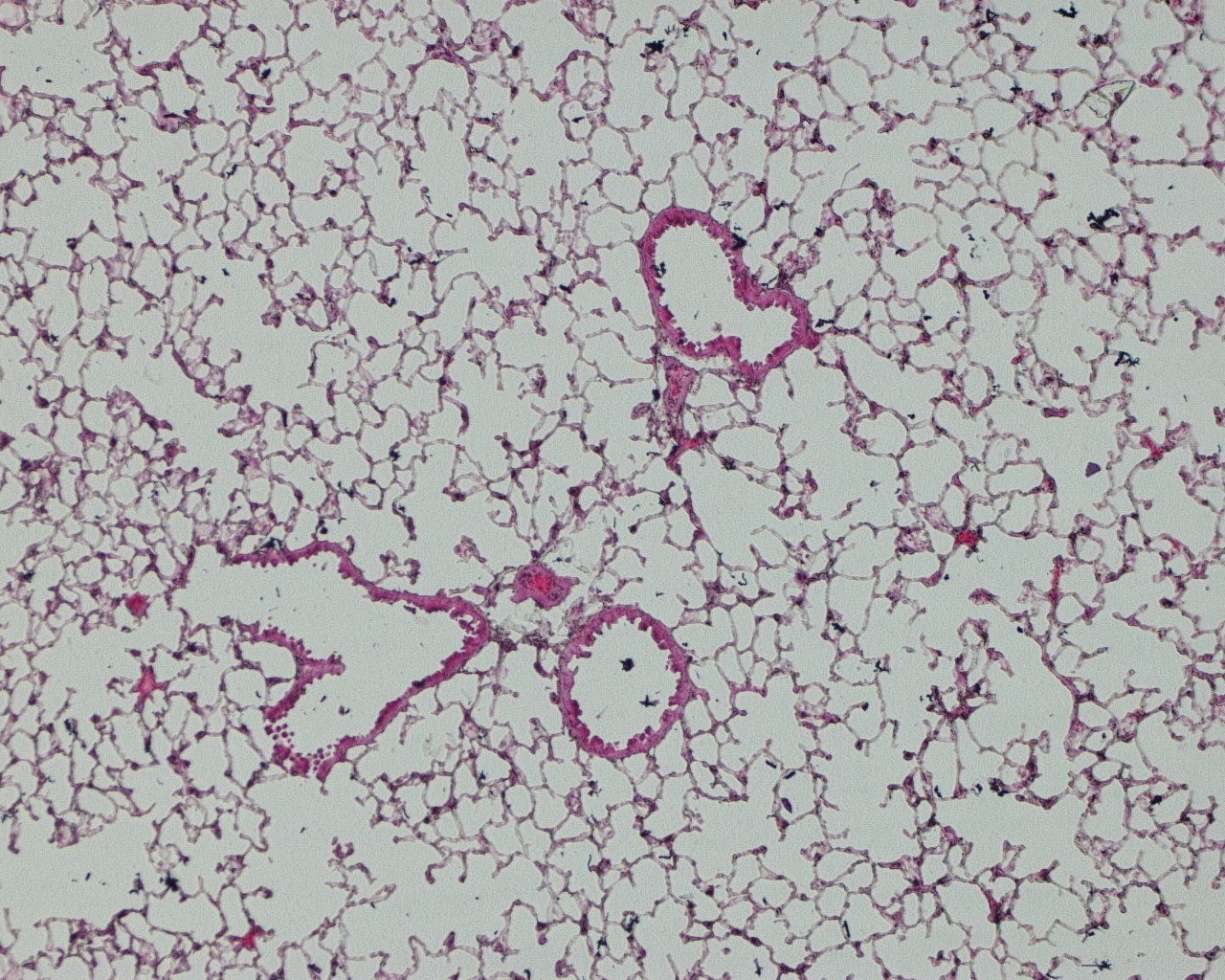

Supplement: S1 File — (ZIP) [file ppat.1012230.s002.zip › S1_File/Fig_7C/Lung/WT-Mock-10X.jpg]

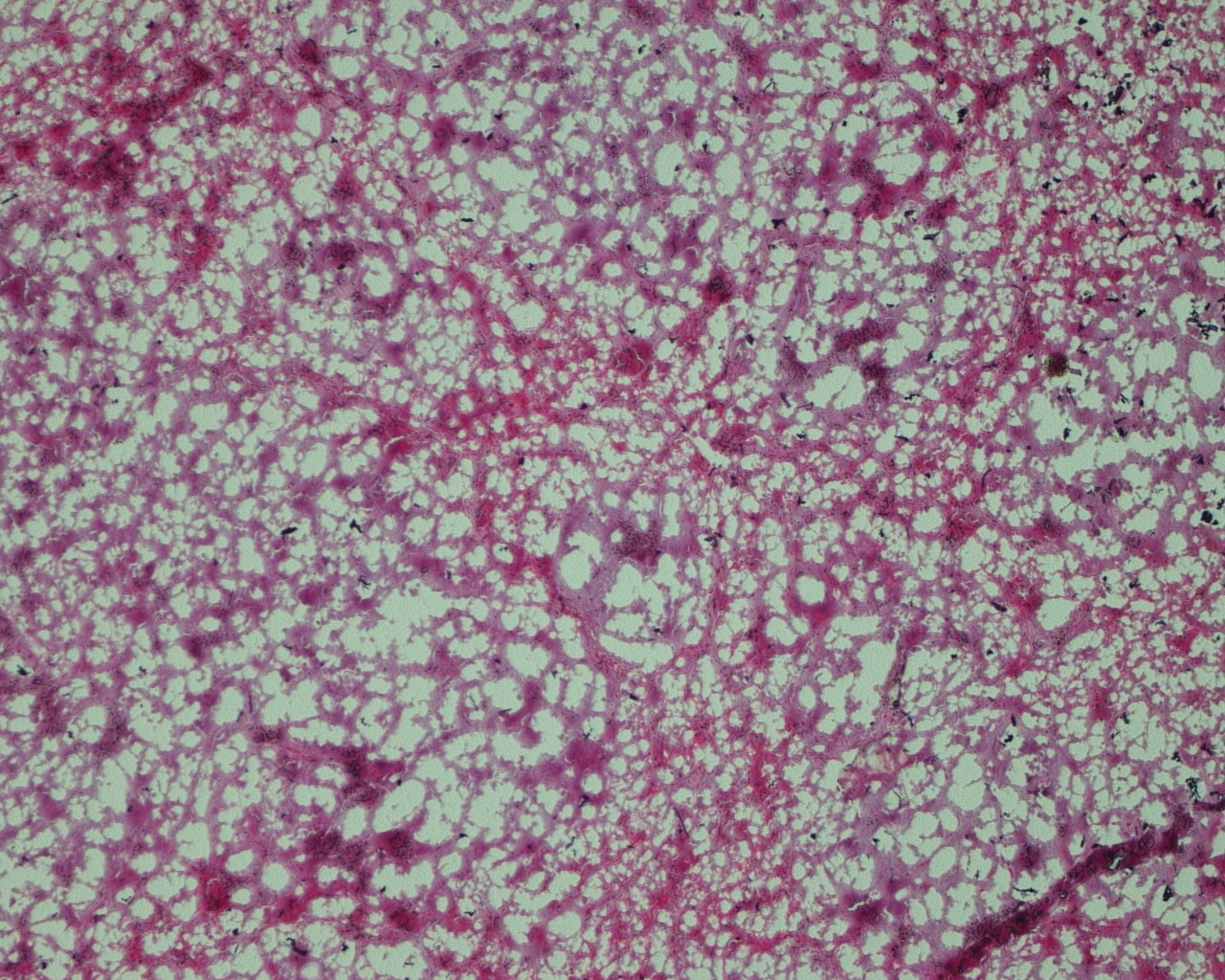

Supplement: S1 File — (ZIP) [file ppat.1012230.s002.zip › S1_File/Fig_7C/Spleen/Adap KO-IAV-10X.jpg]

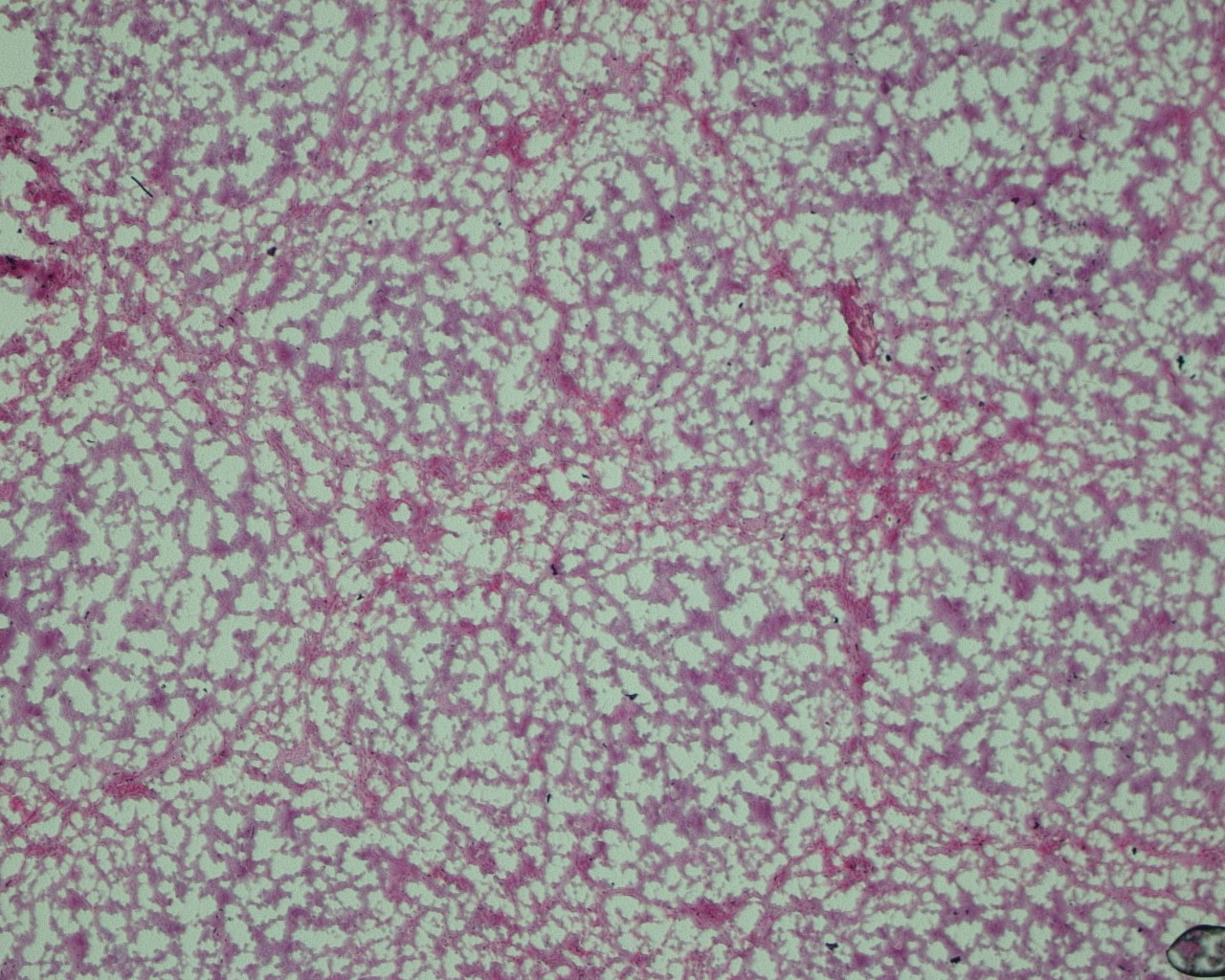

Supplement: S1 File — (ZIP) [file ppat.1012230.s002.zip › S1_File/Fig_7C/Spleen/Adap KO-Mock-10X.jpg]

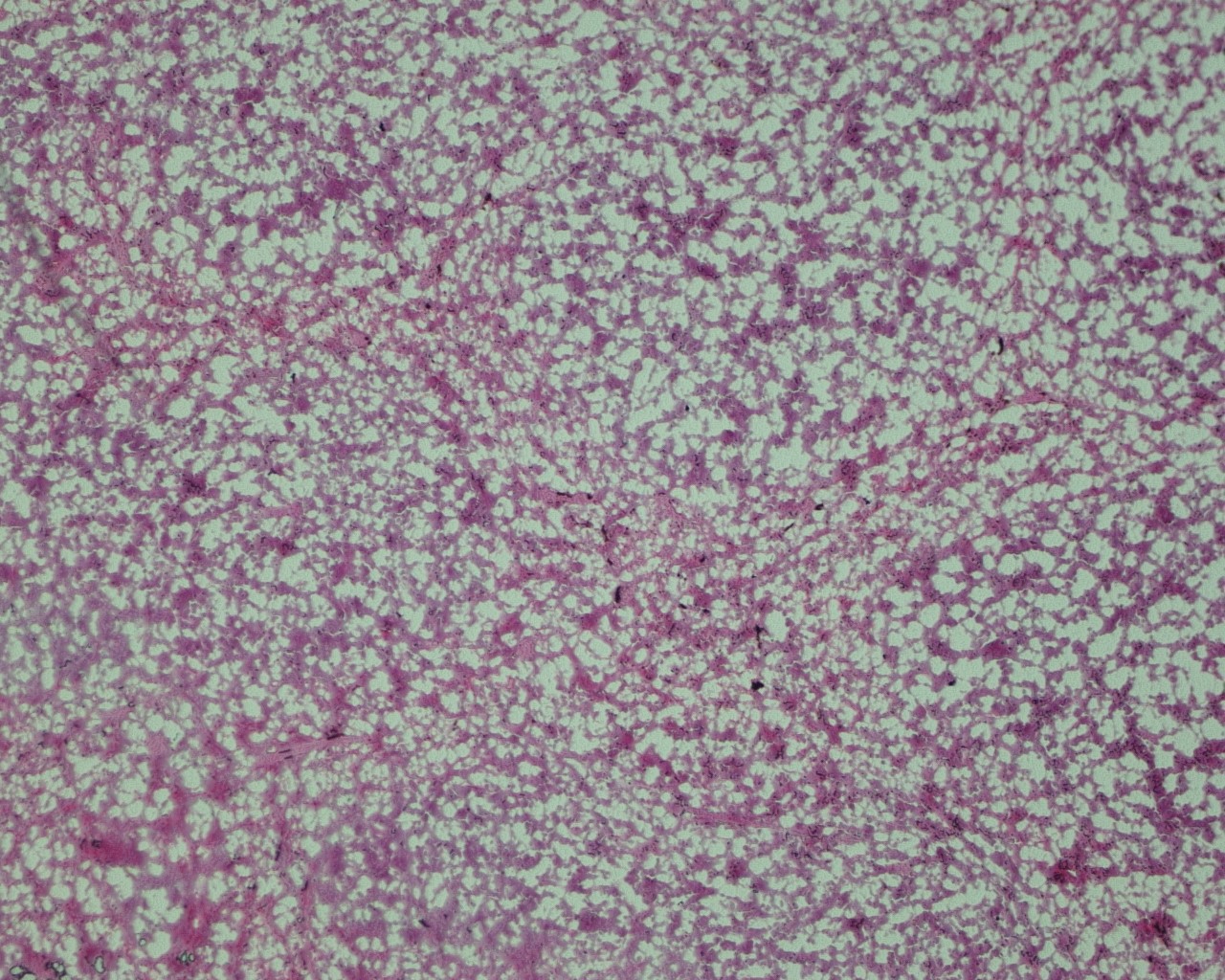

Supplement: S1 File — (ZIP) [file ppat.1012230.s002.zip › S1_File/Fig_7C/Spleen/WT-IAV-10X.jpg]

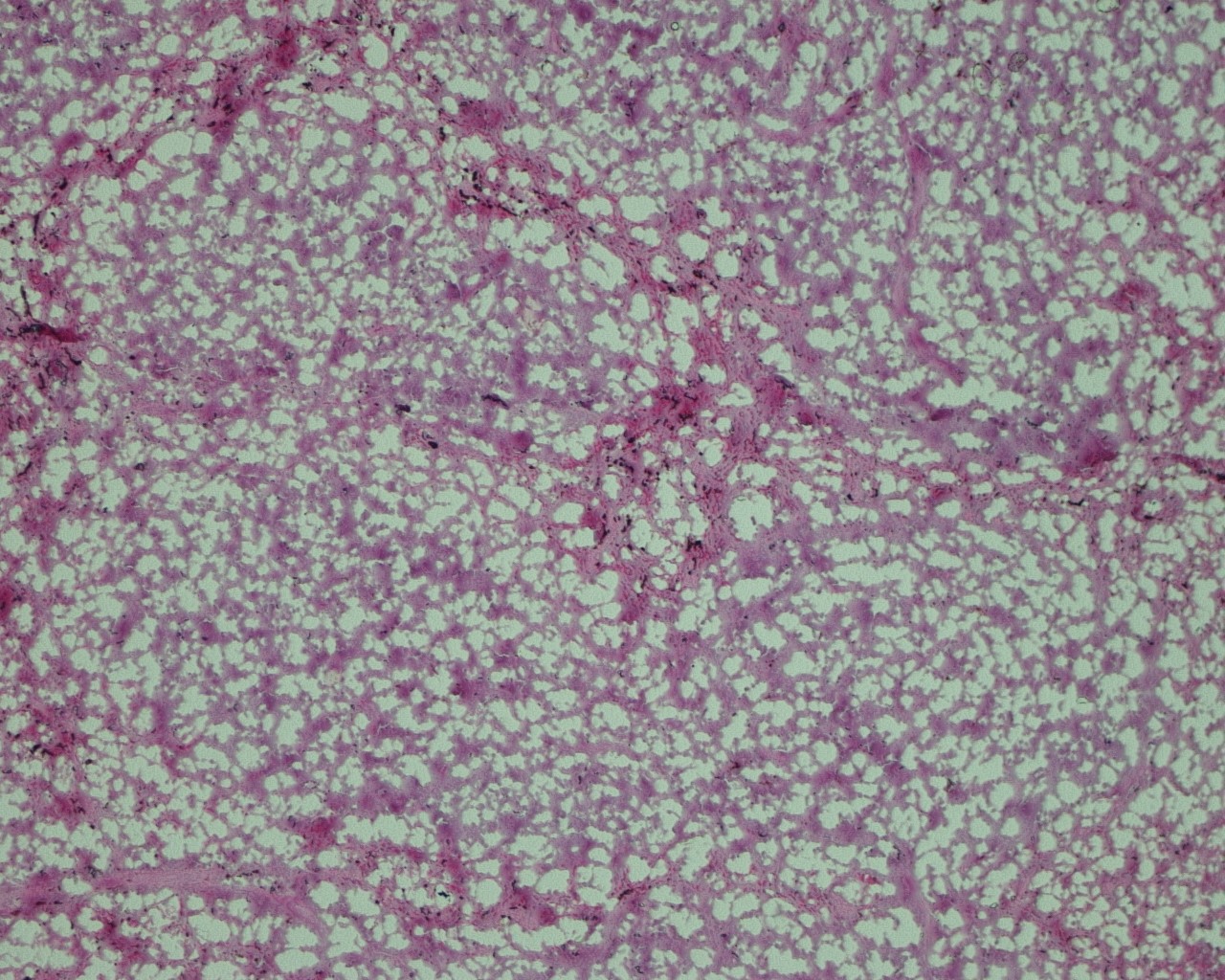

Supplement: S1 File — (ZIP) [file ppat.1012230.s002.zip › S1_File/Fig_7C/Spleen/WT-Mock-10X.jpg]

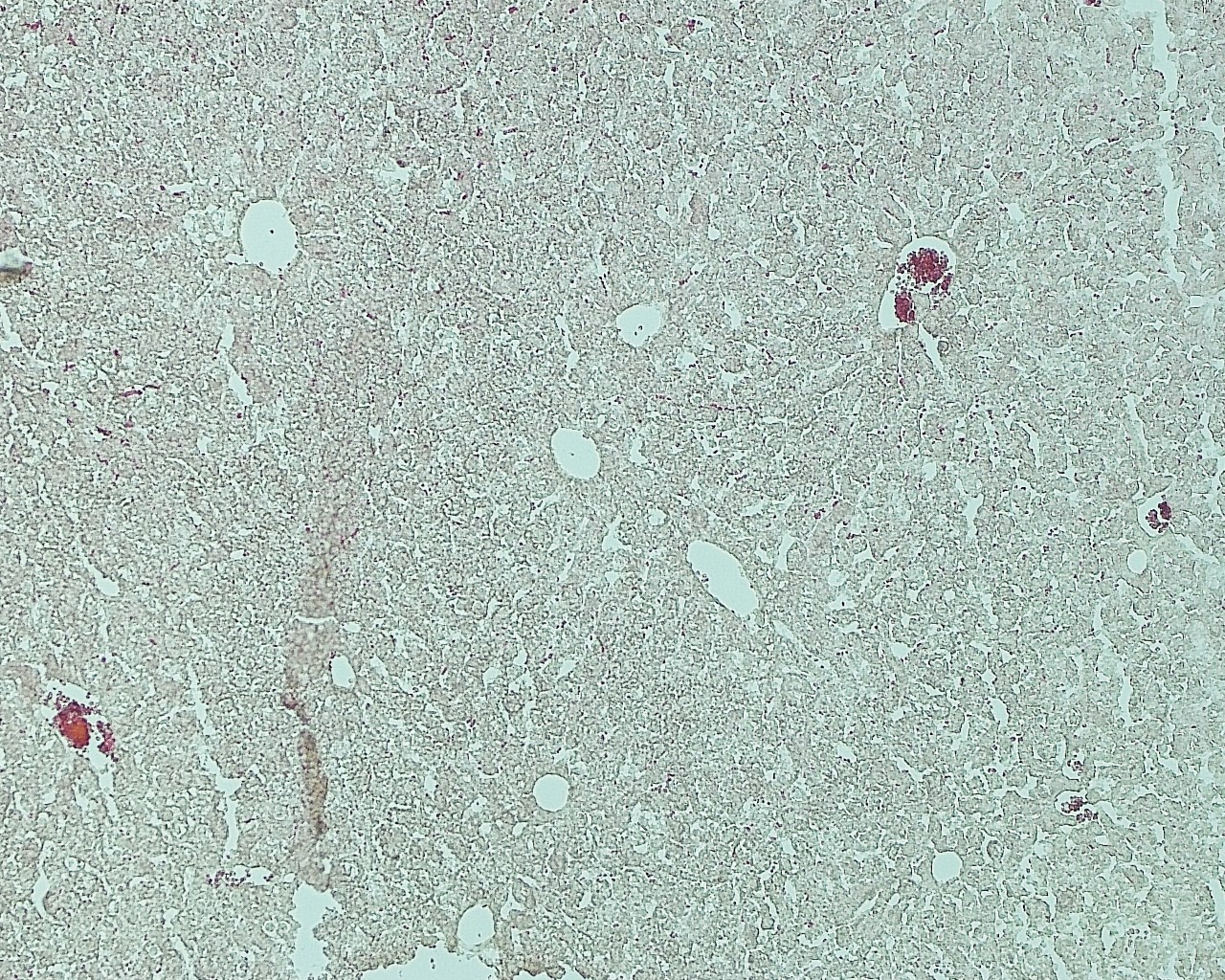

Supplement: S1 File — (ZIP) [file ppat.1012230.s002.zip › S1_File/Fig_7D/Liver/Adap KO-Mock-10X.jpg]

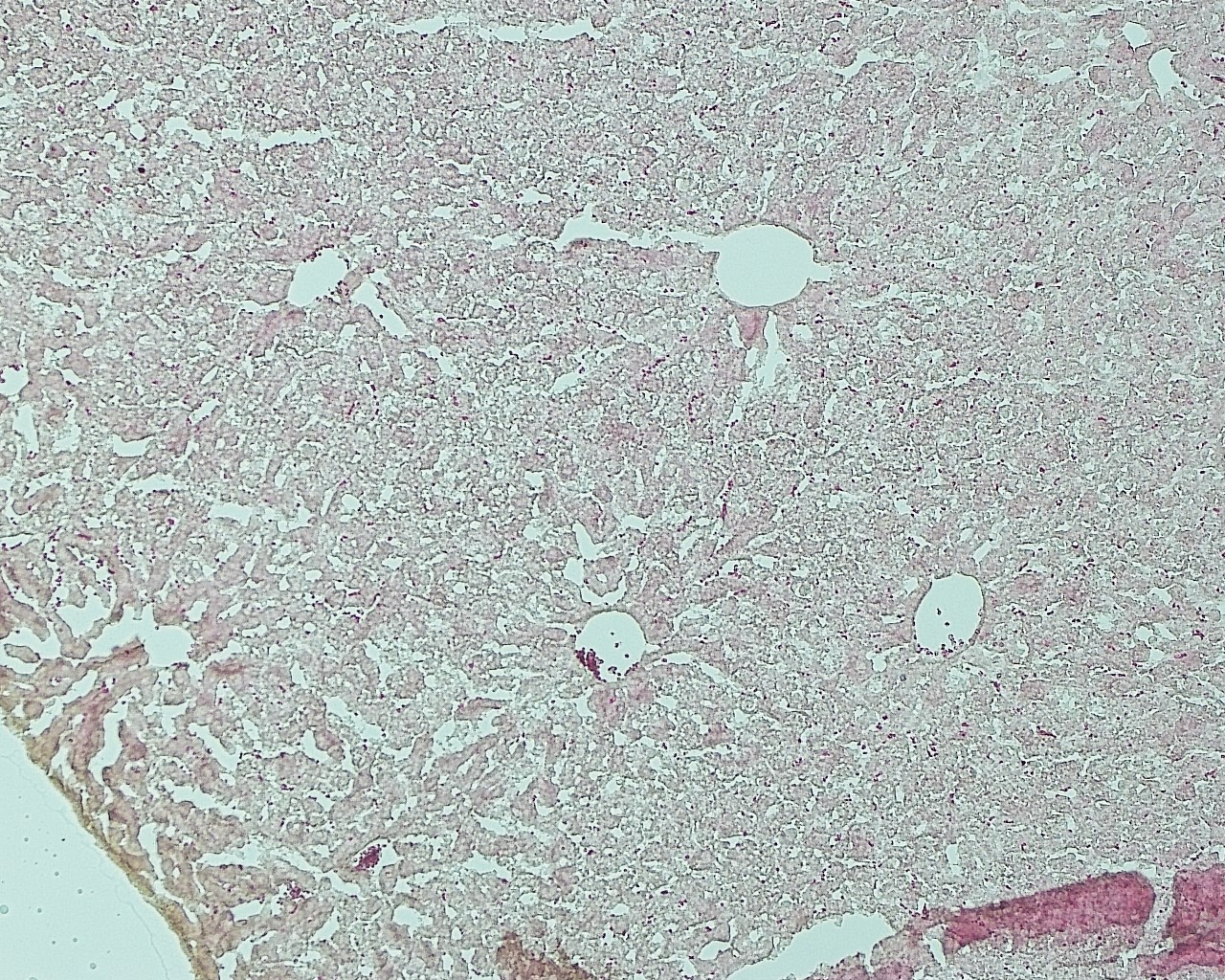

Supplement: S1 File — (ZIP) [file ppat.1012230.s002.zip › S1_File/Fig_7D/Liver/Adap KO-SeV-10X.jpg]

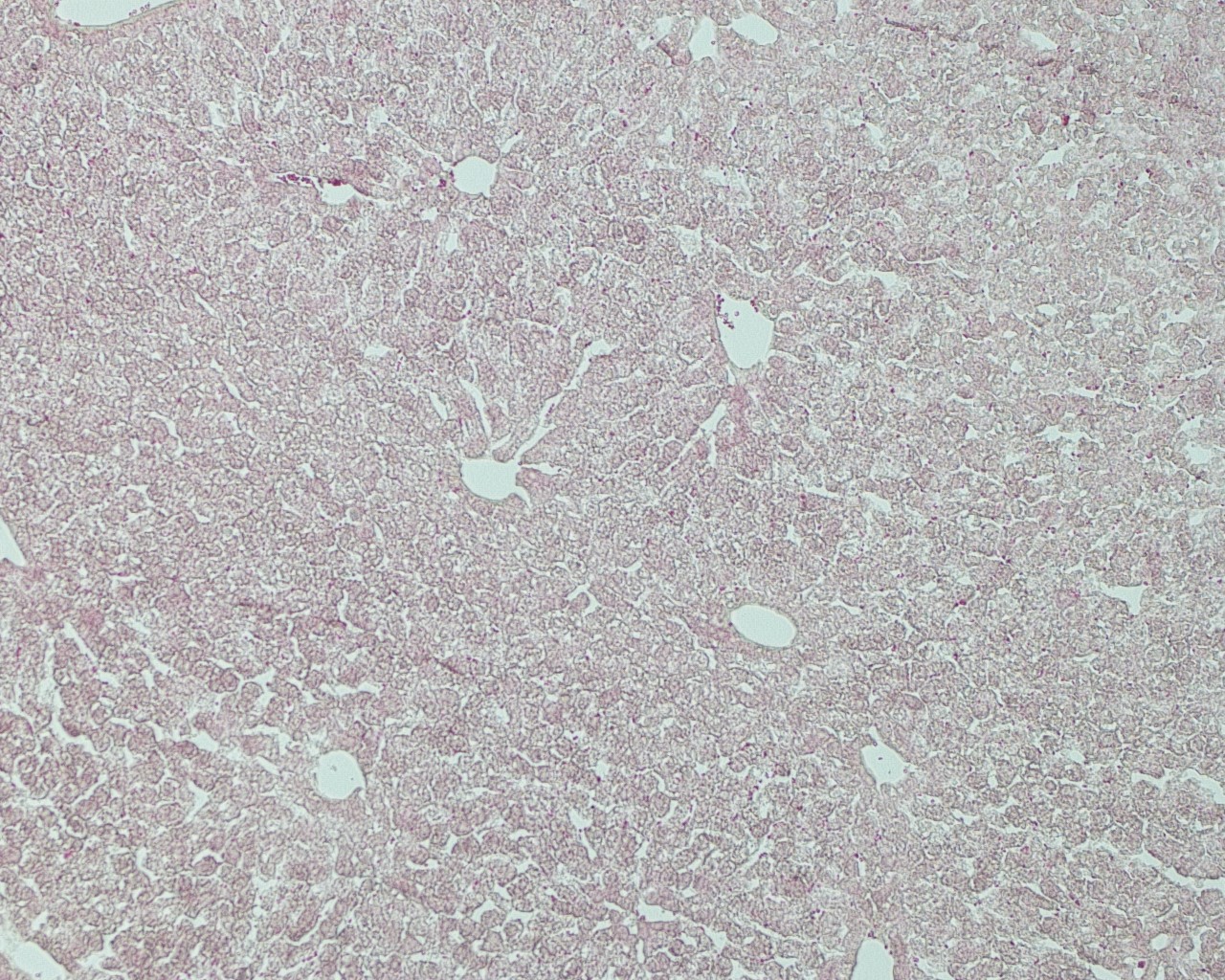

Supplement: S1 File — (ZIP) [file ppat.1012230.s002.zip › S1_File/Fig_7D/Liver/WT-Mock-10X.jpg]

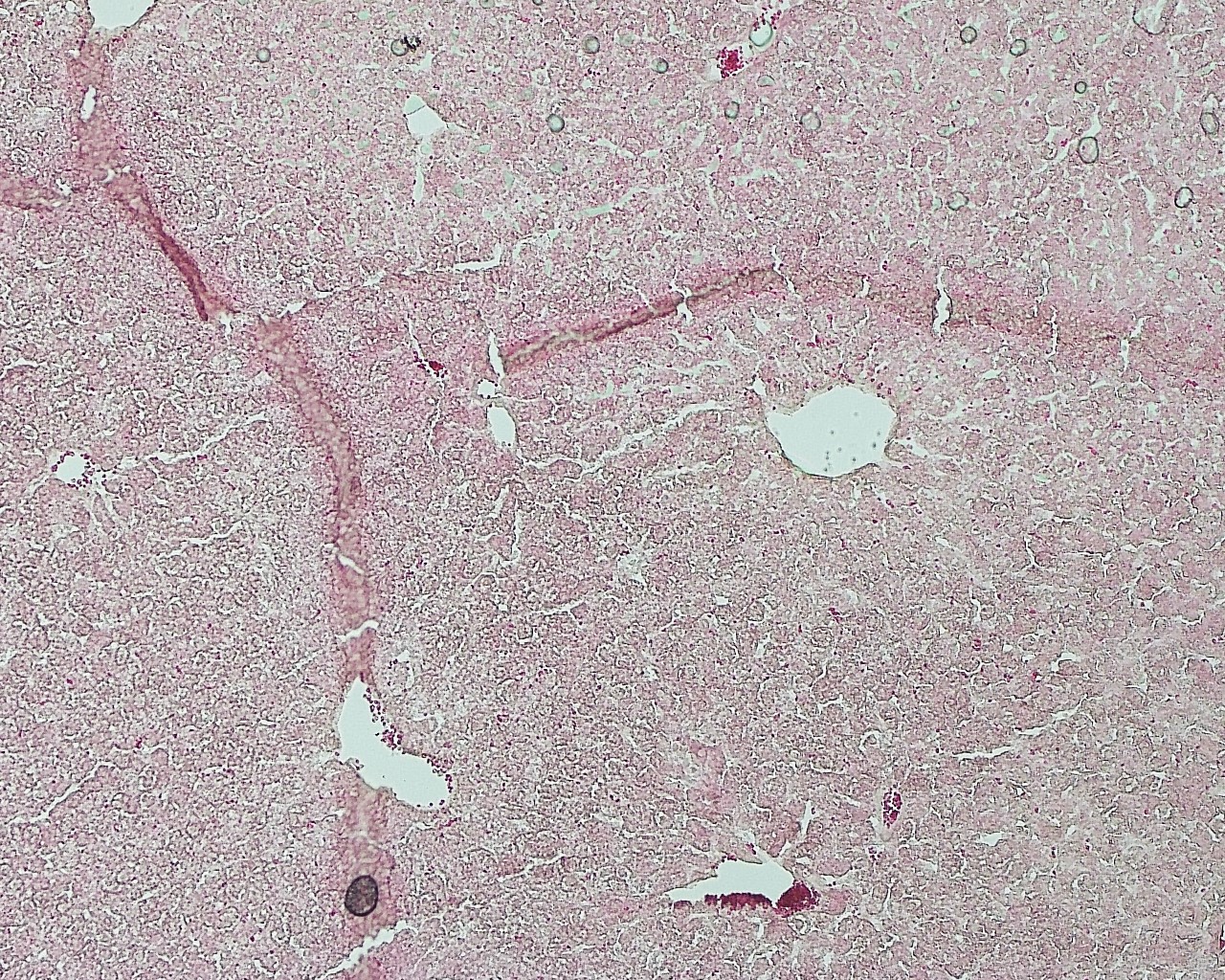

Supplement: S1 File — (ZIP) [file ppat.1012230.s002.zip › S1_File/Fig_7D/Liver/WT-SeV-10X.jpg]

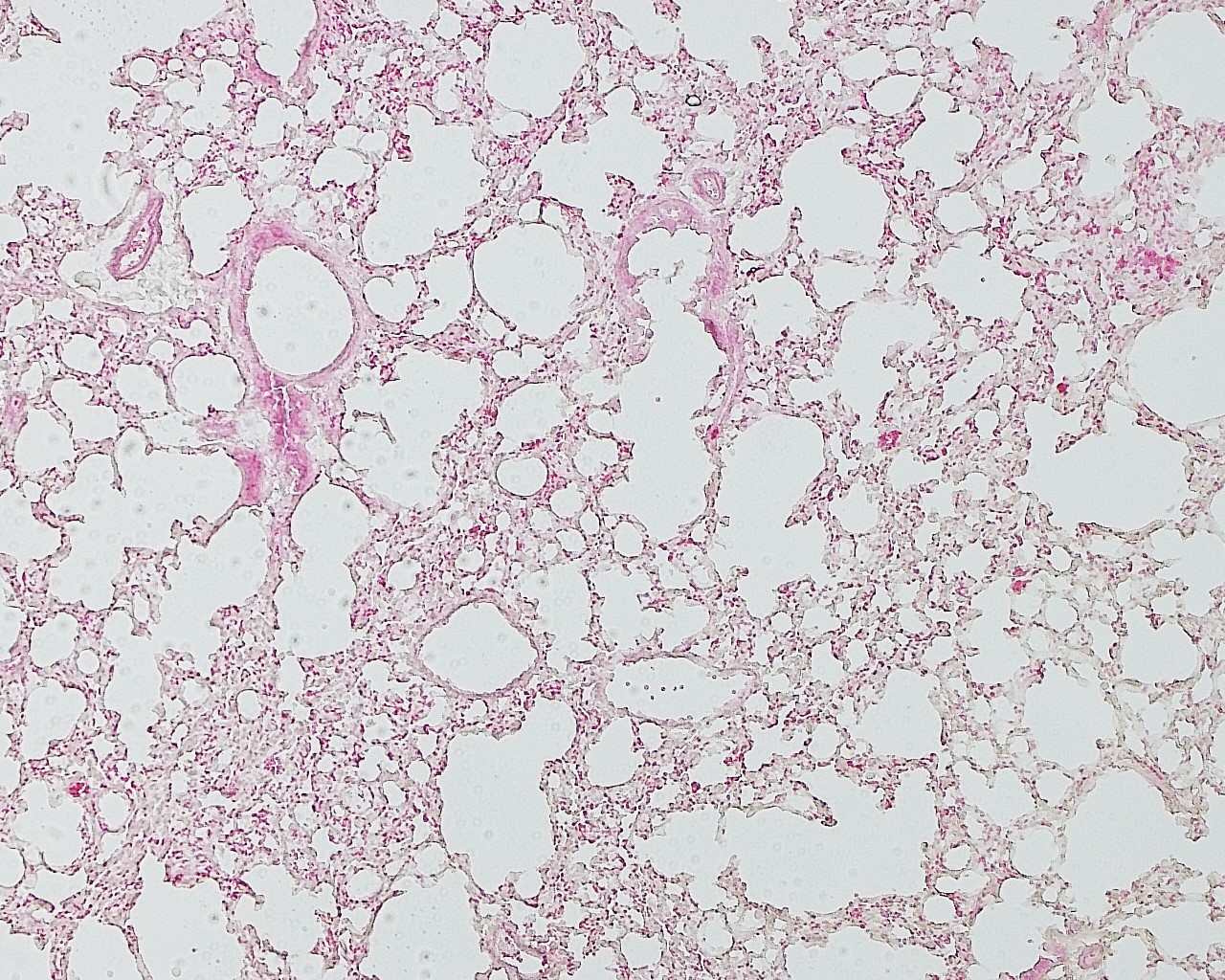

Supplement: S1 File — (ZIP) [file ppat.1012230.s002.zip › S1_File/Fig_7D/Lung/Adap KO-Mock-10X.jpg]

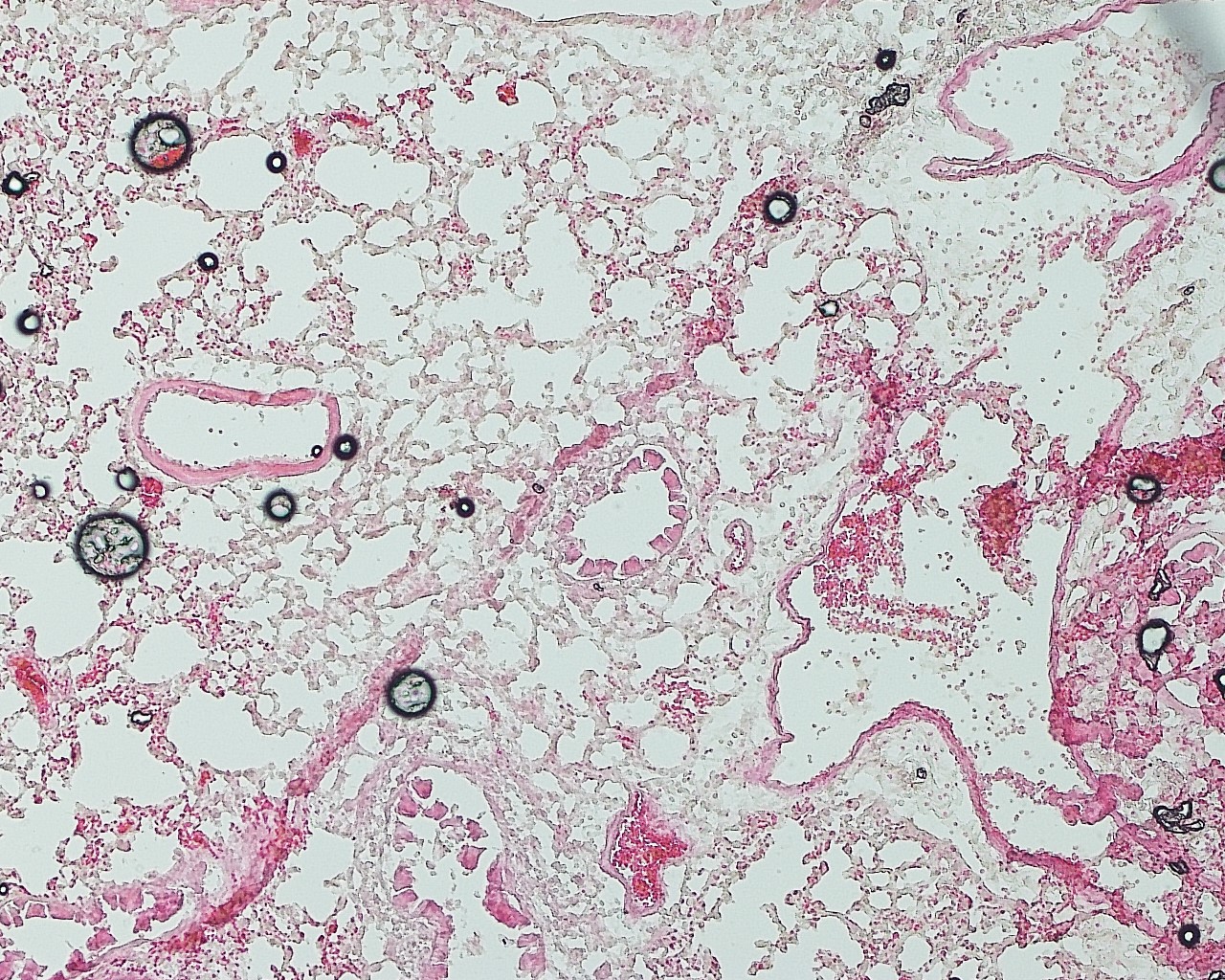

Supplement: S1 File — (ZIP) [file ppat.1012230.s002.zip › S1_File/Fig_7D/Lung/Adap KO-SeV-10X.jpg]

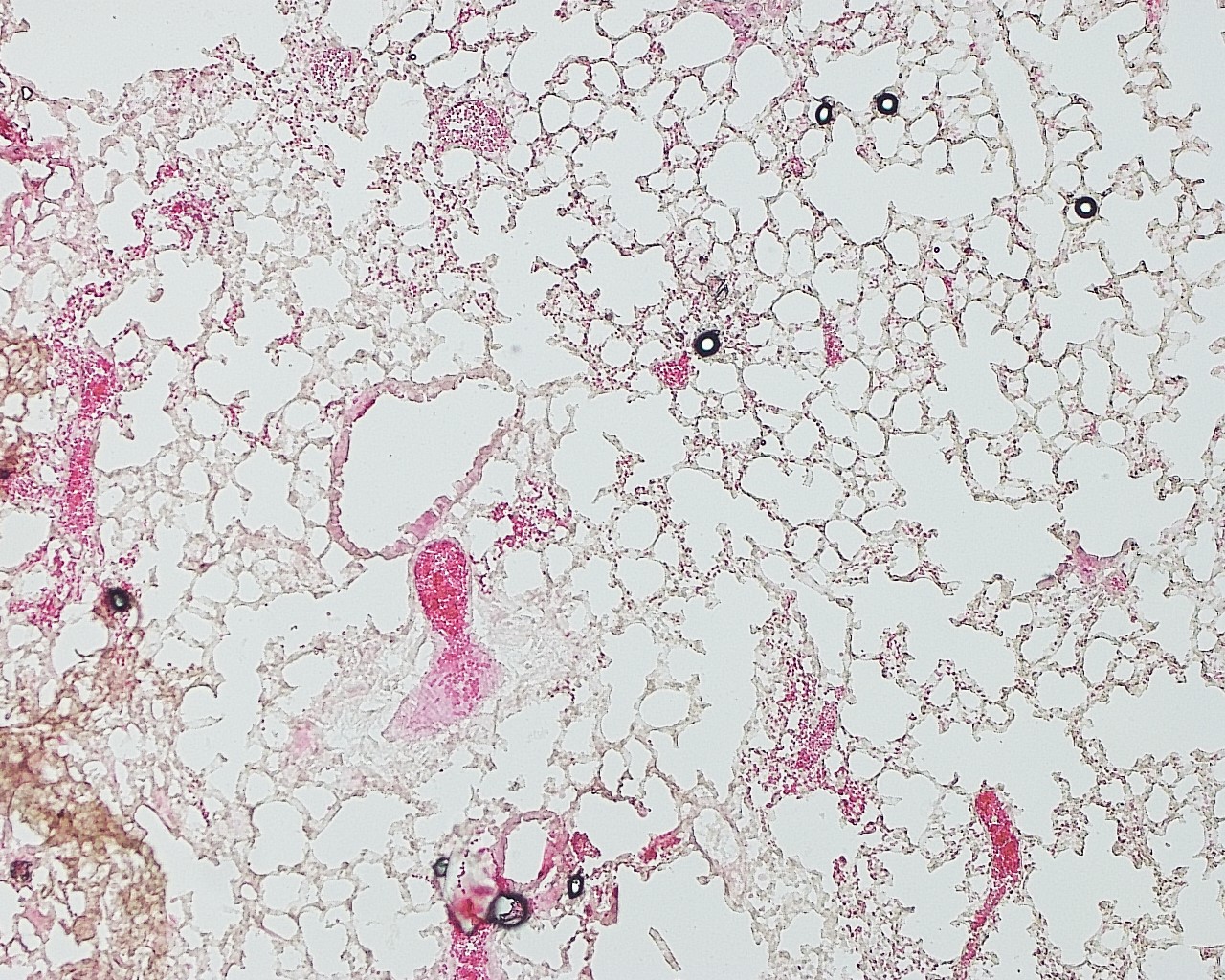

Supplement: S1 File — (ZIP) [file ppat.1012230.s002.zip › S1_File/Fig_7D/Lung/WT-Mock-10X.jpg]

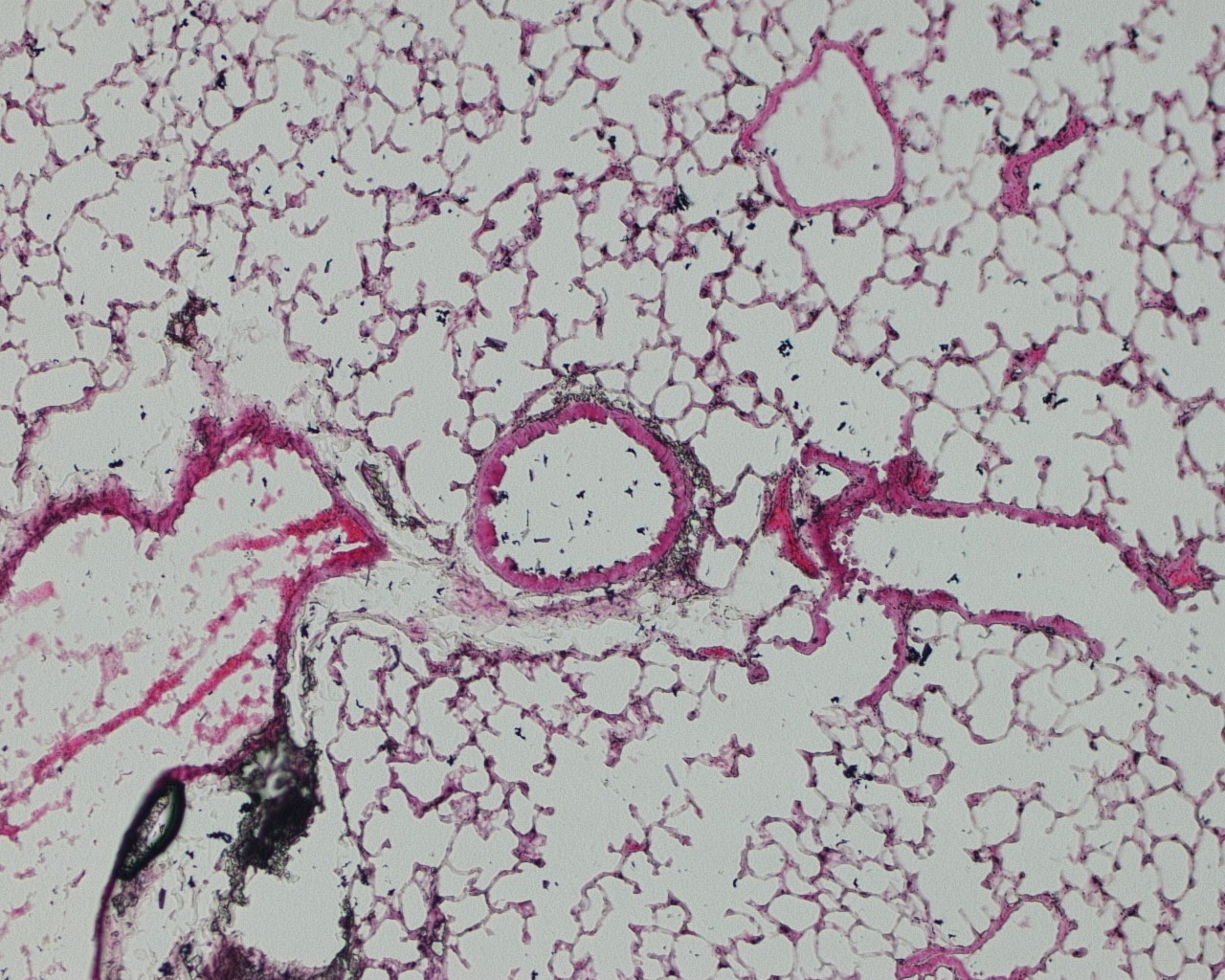

Supplement: S1 File — (ZIP) [file ppat.1012230.s002.zip › S1_File/Fig_7D/Lung/WT-SeV-10X.jpg]

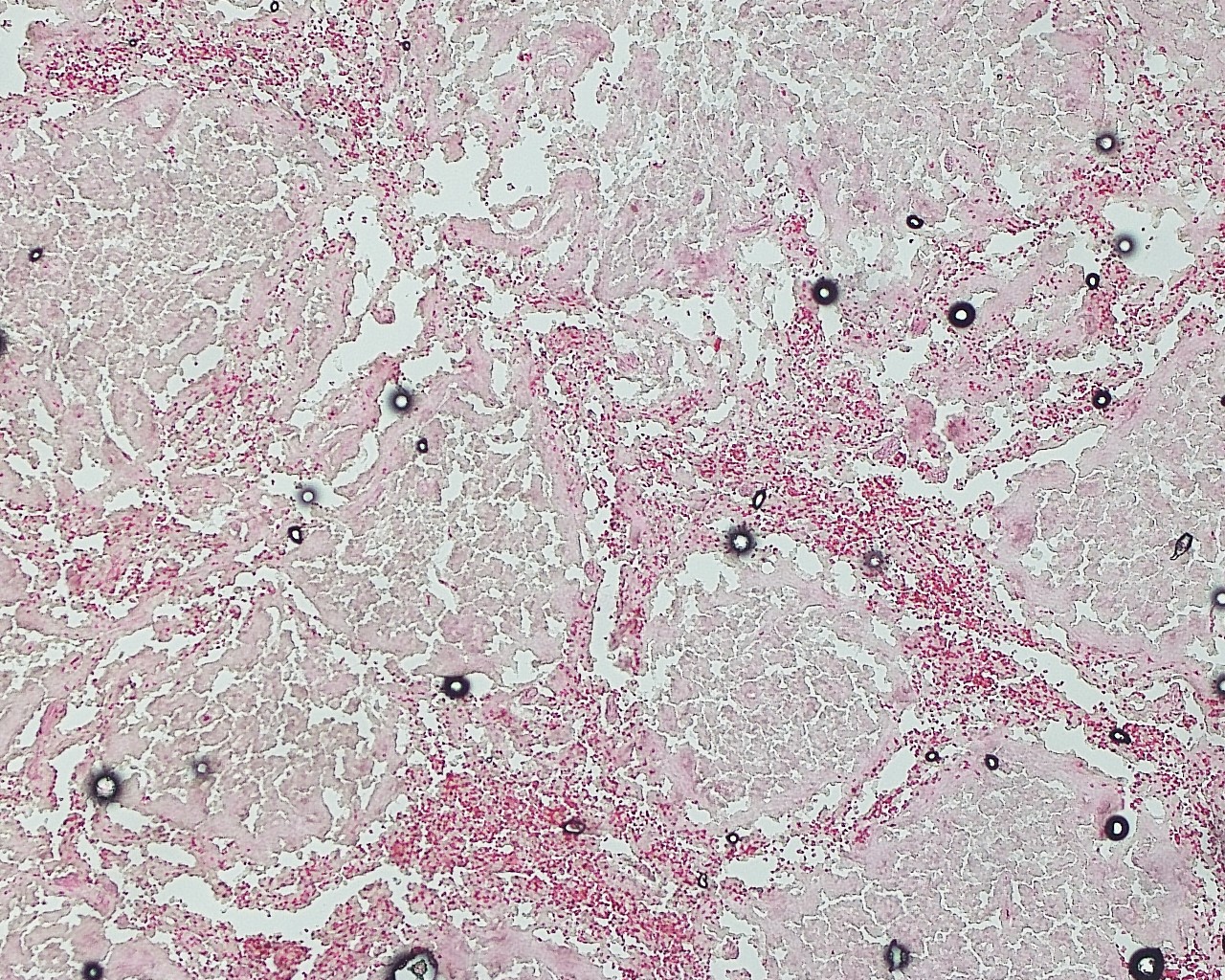

Supplement: S1 File — (ZIP) [file ppat.1012230.s002.zip › S1_File/Fig_7D/Spleen/Adap KO-Mock-10X.jpg]

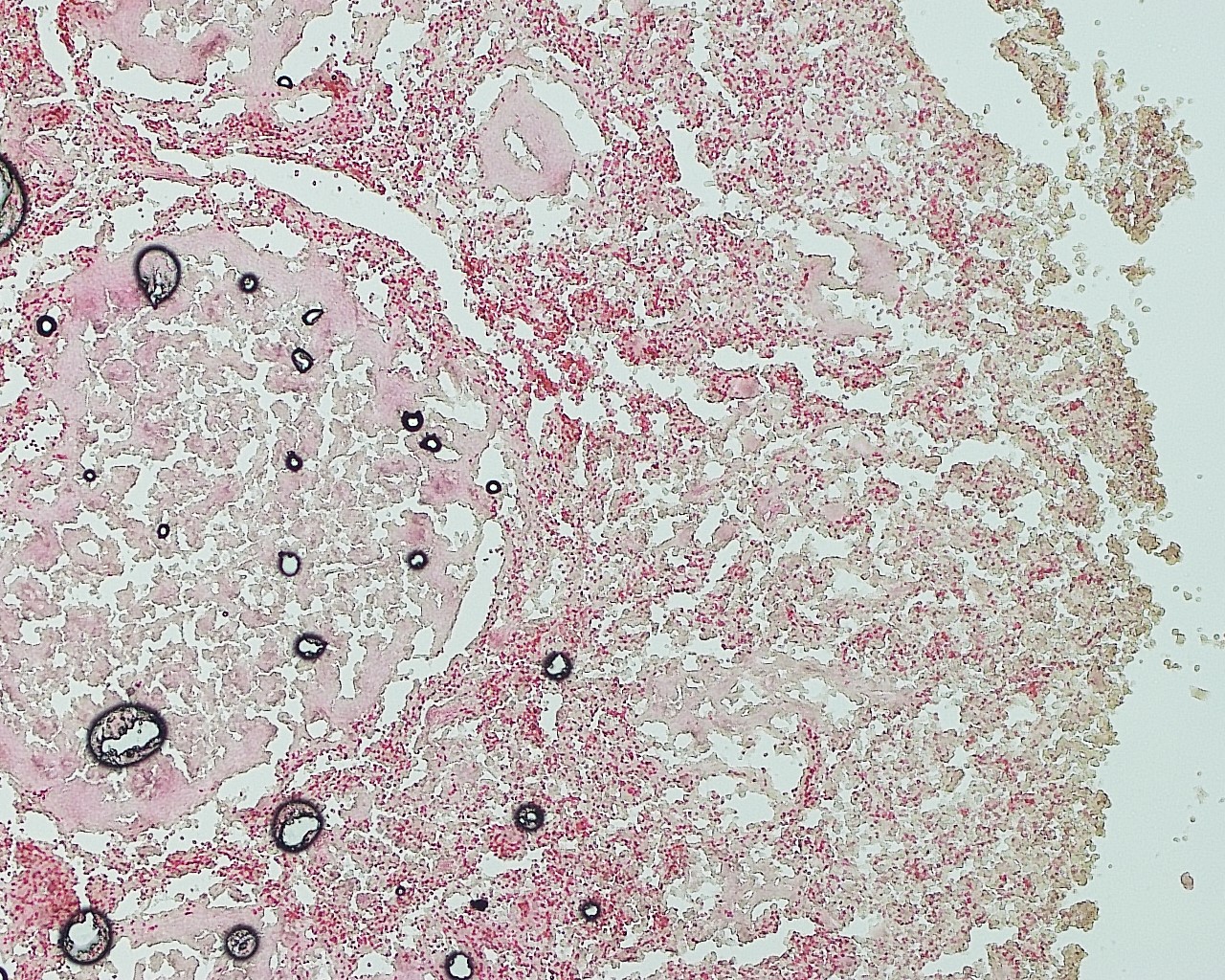

Supplement: S1 File — (ZIP) [file ppat.1012230.s002.zip › S1_File/Fig_7D/Spleen/Adap KO-SeV-10X.jpg]

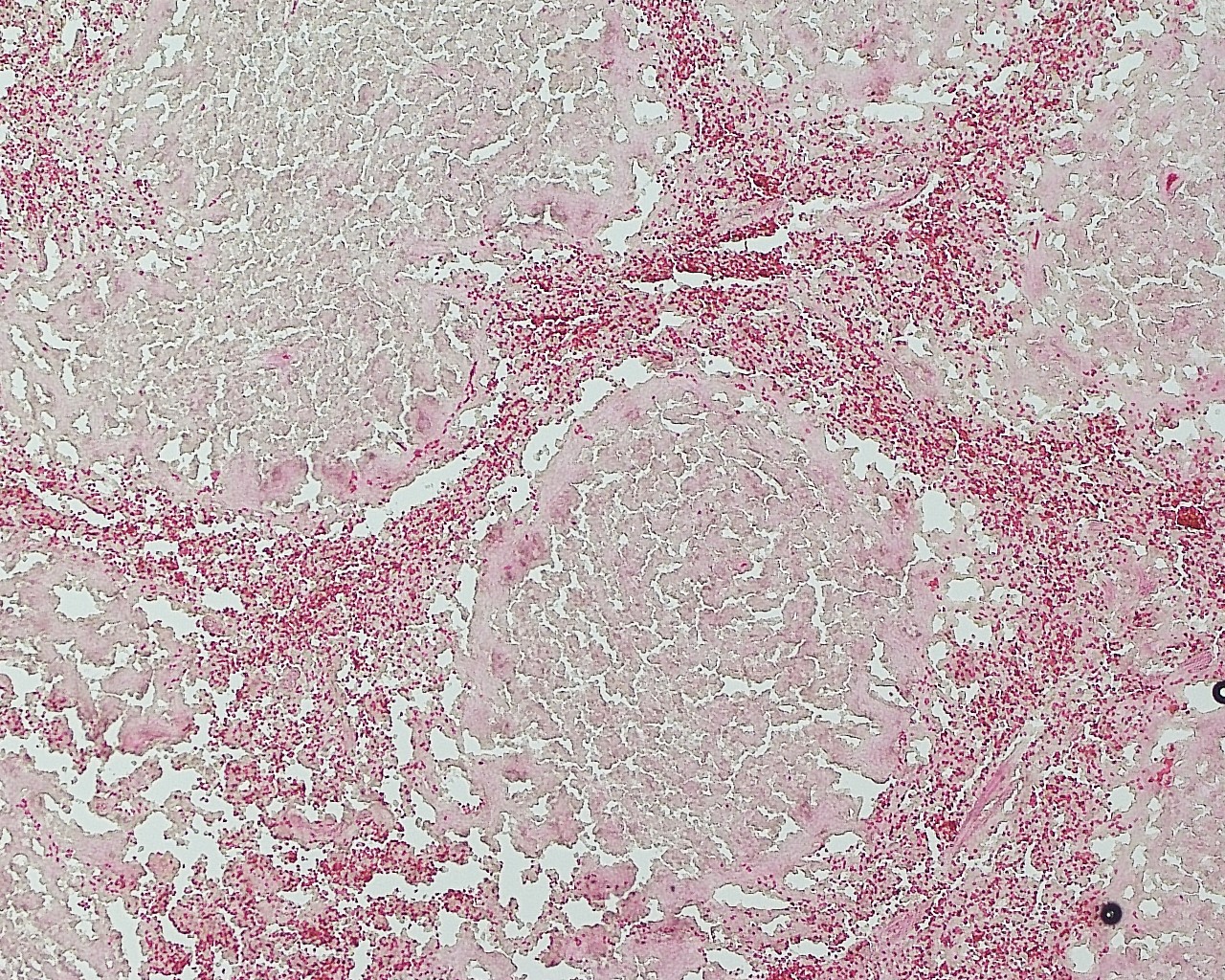

Supplement: S1 File — (ZIP) [file ppat.1012230.s002.zip › S1_File/Fig_7D/Spleen/WT-Mock-10X.jpg]

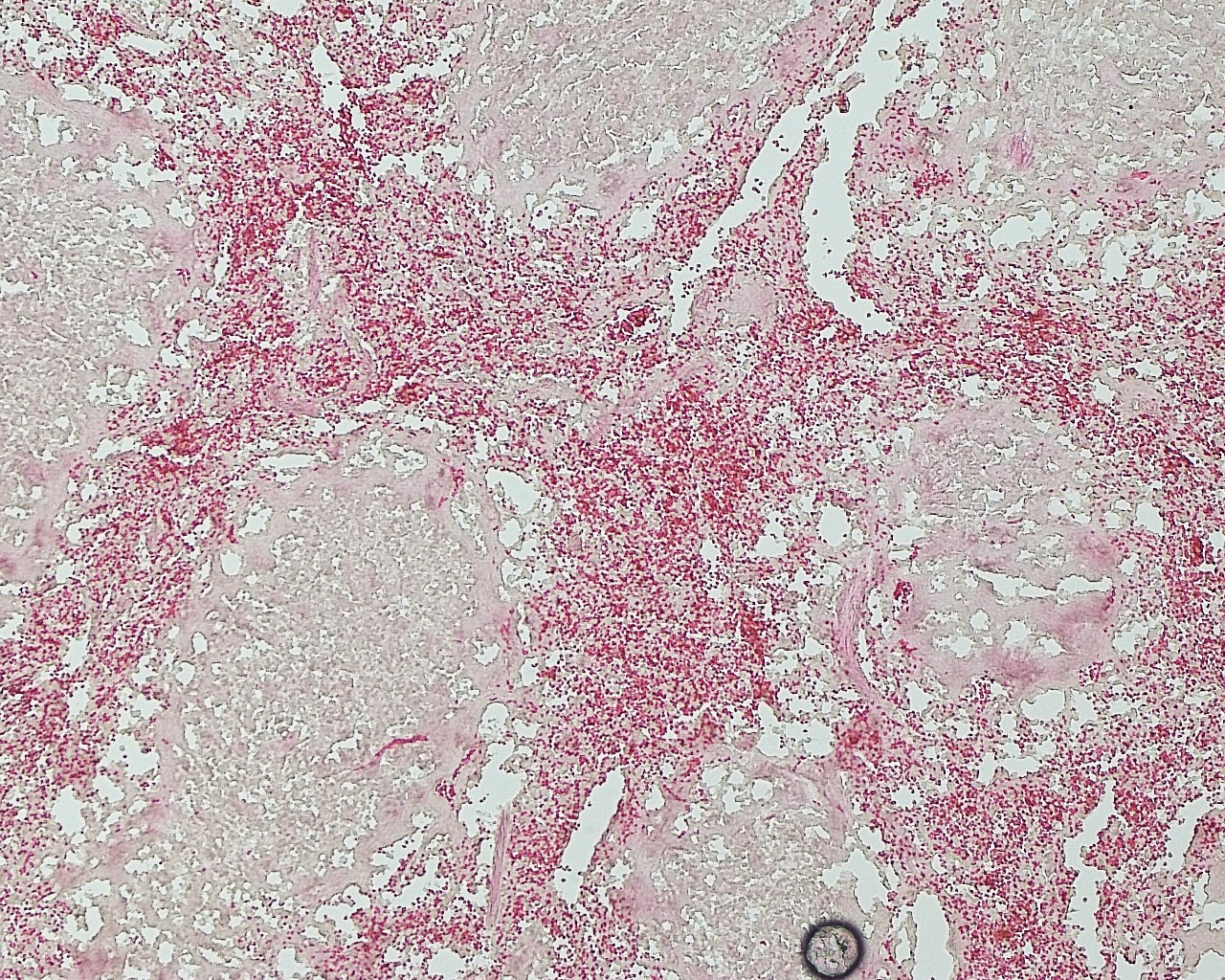

Supplement: S1 File — (ZIP) [file ppat.1012230.s002.zip › S1_File/Fig_7D/Spleen/WT-SeV-10X.jpg]
